# A Novel Hypoxia-Related Gene Signature with Strong Predicting Ability in Non-Small-Cell Lung Cancer Identified by Comprehensive Profiling

**DOI:** 10.1155/2022/8594658

**Published:** 2022-05-19

**Authors:** Huajun Yang, Zhongan Wang, Ling Gong, Guichuan Huang, Daigang Chen, Xiaoping Li, Fei Du, Jiang Lin, Xueyi Yang

**Affiliations:** ^1^Xingyi People's Hospital (Department of Respiratory and Critical Medicine, Xingyi Hospital Affiliated to Guizhou Medical University), Xingyi, Guizhou 562400, China; ^2^The First People's Hospital of Zunyi (Department of Respiratory and Critical Medicine, The Third Affiliated Hospital of Zunyi Medical University), Zunyi, Guizhou 563000, China; ^3^Life Science College, Luoyang Normal University, Luoyang, Henan 471934, China

## Abstract

**Background:**

Non-small-cell lung cancer (NSCLC) is the most common malignant tumor among males and females worldwide. Hypoxia is a typical feature of the tumor microenvironment, and it affects cancer development. Circular RNAs (circRNAs) have been reported to sponge miRNAs to regulate target gene expression and play an essential role in tumorigenesis and progression. This study is aimed at identifying whether circRNAs could be used as the diagnostic biomarkers for NSCLC.

**Methods:**

The heterogeneity of samples in this study was assessed by principal component analysis (PCA). Furthermore, the Gene Expression Omnibus (GEO) database was normalized by the affy R package. We further screened the differentially expressed genes (DEGs) and differentially expressed circular RNAs (DEcircRNAs) using the DEseq2 R package. Moreover, we analyzed the Gene Ontology (GO) annotation and Kyoto Encyclopedia of Genes and Genomes (KEGG) enrichment of DEGs using the cluster profile R package. Besides, the Gene Set Enrichment Analysis (GSEA) was used to identify the biological function of DEGs. The interaction between DEGs and the competing endogenous RNAs (ceRNA) network was detected using STRING and visualized using Cytoscape. Starbase predicted the miRNAs of target hub genes, and miRanda predicted the target miRNAs of circRNAs. The RNA-seq profiler and clinical information were downloaded from The Cancer Genome Atlas (TCGA) database. Then, the variables were assessed by the univariate and multivariate Cox proportional hazard regression models. Significant variables in the univariate Cox proportional hazard regression model were included in the multivariate Cox proportional hazard regression model to analyze the association between the variables of clinical features. Furthermore, the overall survival of variables was determined by the Kaplan-Meier survival curve, and the time-dependent receiver operating characteristic (ROC) curve analysis was used to calculate and validate the risk score in NSCLC patients. Moreover, predictive nomograms were constructed and used to predict the prognostic features between the high-risk and low-risk score groups.

**Results:**

We screened a total of 2039 DEGs, including 1293 upregulated DEGs and 746 downregulated DEGs in hypoxia-treated A549 cells. A549 cells treated with hypoxia had a total of 70 DEcircRNAs, including 21 upregulated and 49 downregulated DEcircRNAs, compared to A549 cells treated with normoxia. The upregulated genes were significantly enriched in 284 GO terms and 42 KEGG pathways, while the downregulated genes were significantly enriched in 184 GO terms and 25 KEGG pathways. Moreover, the function analysis by GSEA showed enrichment in the enzyme-linked receptor protein signaling pathway, hypoxia-inducible factor- (HIF-) 1 signaling pathway, and G protein-coupled receptor (GPCR) downstream signaling. Furthermore, six hub modules and 10 hub genes, CDC45, EXO1, PLK1, RFC4, CCNB1, CDC6, MCM10, DLGAP5, AURKA, and POLE2, were identified. The ceRNA network was constructed, and it consisted of 4 circRNAs, 14 miRNAs, and 38 mRNAs. The ROC curve was constructed and calculated. The area under the curve (AUC) value was 0.62, and the optimal threshold was 0.28. Based on the optimal threshold, the patients were divided into the high-risk score and low-risk score groups. The survival rate in the high-risk score group was lower than that in the low-risk score group. The expression of SERPINE1, STC2, and LPCAT1; clinical stage; and age of the patient were significantly correlated with the high-risk score. Moreover, nomograms were established based on the risk factors in multivariate analysis, and the median survival time, 3-year survival probability, and 5-year survival were possibly predicted according to nomograms.

**Conclusion:**

The ceRNA network associated with NSCLC was identified, and the hub genes, circRNAs, might act as the potential biomarkers for NSCLC.

## 1. Introduction

Lung cancer is the leading cause of cancer-related mortality worldwide [1]. More than 80% of lung cancer cases are diagnosed as non-small-cell lung cancer (NSCLC) [2]. According to the histopathology, there are about 40% adenocarcinomas, 25% squamous cell carcinomas, 10% lung squamous cell carcinomas, large cell carcinomas, and sarcomatoid carcinomas in NSCLC. The main manifestations of NSCLC are cough, expectoration, hemoptysis, wheezing, dyspnea, fever, and weight loss. Intrathoracic metastasis may be accompanied by chest pain, pleural effusion, hoarseness, superior vena cava obstruction syndrome, Horner syndrome, and dysphagia [3]. At present, lung cancer is mainly screened by X-ray and chest computed tomography (CT). The final diagnosis depends on exfoliative sputum cytology, bronchoscopy, video-assisted thoracoscopic surgery, lung biopsy, and surgical procedures for pathological examination [7]. As a result, most NSCLC patients are already in an advanced stage at the time of detection, which seriously affects the treatment of patients [4]. On the other hand, although surgery is advocated in the early stage, and radiotherapy, chemotherapy, targeted therapy, or combined immunotherapy are advocated in the middle and late stages, there are often some problems, such as recurrence after surgery, gene mutation, and drug resistance after chemotherapy and targeted therapy [5, 6]. Moreover, many patients cannot tolerate the toxic effects and side effects, and the 5-year survival rate is still lower than 15% [7, 8]. Therefore, it is very important to better understand the molecular mechanisms underlying NCSLC and explore the potential biomarkers for the diagnosis and treatment of NSCLC.

Hypoxia is an inherent feature of solid tumors because of the imbalance between tumor cell proliferation rates and vascular nutrient supply [9]. Increasing evidence has revealed that hypoxia plays a critical role in tumor occurrence and development, including lung cancer [10]. For example, under the hypoxic microenvironment, tumor cells can undergo epithelial-mesenchymal transition (EMT), thus increasing the degree of malignancy of the tumor [11]. Hypoxia can enhance Wnt signal activity by stabilizing *β*-catenin and changing its location in the nucleus, promoting proliferation, migration, invasion, and EMT of lung cancer cells, and inhibiting apoptosis [12]. In addition, hypoxia also activates PI3K/Akt and Wnt signaling in a HIF-2 *α-*dependent manner, thereby enhancing the resistance of lung cancer cells to chronic hypoxia-induced stress, inducing EMT of tumor cells and increasing the malignancy of tumor cells [13]. Furthermore, the expression of mir-191 is upregulated after hypoxia in NSCLC, which leads to a decrease in NFIA expression and promotes the proliferation and migration of lung cancer cells [14]. Under hypoxic conditions, autophagy enhances the antiradiation ability of NSCLC by regulating the level of reactive oxygen species (ROS), affecting the effect of radiotherapy and leading to a poor prognosis of lung cancer [15]. Hypoxia can also upregulate the level of galectin-3, thus enhancing the activity of RhoA and significantly increasing the cell migration and invasion activity [16]. In particular, recent research studies have found that hypoxia is associated with the prognosis of cancers and can act as a target for treatment [17, 18]. However, how hypoxia regulates NSCLC has not been fully elucidated. Therefore, research studies focusing on the molecular mechanism of hypoxia in the occurrence and progression may contribute to the screening of novel biomarkers for the diagnosis and treatment of NSCLC.

Circular RNAs (circRNAs) are a group of noncoding RNAs derived from precursor mRNA back-splicing, and they form a closed covalent circular structure [19]. circRNAs are abundant in humans and have many biological functions [20], including acting as “sponges” of miRNAs that regulate gene expression at the posttranscriptional level [21]. Recent studies have suggested that numerous circRNAs have been reported to play an important role in the proliferation, migration, and invasion of NSCLC cells [7]. For instance, circ_0000284 [19] and circ_0074027 [22] have been reported to promote NSCLC progression by increasing CUL4B through sponging miR-335-5p and by upregulating PD-L1 expression through sponging miR-337-3p, respectively. Upregulated circular RNA vangl1 is involved in NSCLC progression by inhibiting miR-195 and activating Bcl-2 [23]. Besides, abnormal expression of circRNAs is significantly associated with cisplatin resistance in NSCLC, which provides a new therapeutic target for reversing cisplatin resistance in NSCLC [24]. Song et al. have reported that the host genes of circRNAs identified in cisplatin-resistant NSCLC cell lines were involved in biological processes and pathways contributing to cisplatin resistance in NSCLC, indicating the therapeutic role of circRNAs in NSCLC [24]. However, to our knowledge, circRNAs associated with hypoxia in NSCLC have not yet been fully explored.

Therefore, the current study was designed to explore circRNAs in NSCLC associated with hypoxia, followed by constructing a hypoxia-related ceRNA regulatory network and investigating the potential prognostic biomarkers in NSCLC via bioinformatic analysis. Thus, the results of this study will enhance our understanding of the mechanisms underlying hypoxia-related NSCLC and provide novel insights into the diagnosis and treatment of NSCLC.

## 2. Materials and Methods

### 2.1. Data Acquisition and Processing

The circRNA expression profiles and transcriptome expression profiles were obtained from the GSE131378 dataset of the GEO database (https://www.ncbi.nlm.nih.gov/geo/). The GSE131378 dataset included 2 normoxia- and 2 hypoxia-treated A549 cells. The RNA-sequencing (RNA-seq) data and paired clinical information from 497 NSCLC samples and 49 standard samples were obtained from The Cancer Genome Atlas (TCGA, https://portal.gdc.cancer.gov/). The raw data obtained from the Gene Expression Omnibus (GEO) database was normalized using the affy R package for subsequent analysis. To eliminate the system error and verify the reliable data for the subsequent analysis, quality control (QC) of data in this study was assessed by the principal component analysis (PCA).

### 2.2. Screening of Differentially Expressed Genes (DEGs) and Differentially Expressed circRNAs (DEcircRNAs)

DEGs and DEcircRNAs between normoxia-treated A549 cells and hypoxia-treated A549 cells were screened using the DEseq2 R package with a cutoff value of |log2 (FC)| > 1 and a *p* value < 0.05 (FC, fold change).

### 2.3. Gene Ontology (GO) and Kyoto Encyclopedia of Genes and Genomes (KEGG) Pathway Enrichment Analysis

GO and KEGG enrichment analysis was performed using the clusterProfiler R package. *p* < 0.5 was used as a threshold to identify significant pathways. GO biological function analysis consisted of biological process (BP), molecular function (MF), and cellular component (CC).

### 2.4. Gene Set Enrichment Analysis (GSEA)

GSEA performed the gene function analysis of DEGs. The function pathways were assessed between the high- and low-expression groups of DEGs. A threshold |log2 (FC)| > 1 and *p* < 0.05 were used as the criteria.

### 2.5. Protein-Protein Interaction (PPI) Network Construction and Hub Gene Identification

To further explore the interaction among the DEGs and identify the hub genes, a PPI network of DEGs was constructed using the Search Tool for the Retrieval of Interaction Genes (STRING) database (http://string-db.org/). The interaction network was constructed with a threshold score > 0.7. The PPI network was analyzed and visualized using Cytoscape software. The hub modules were identified with a cutoff value degree > 5, and the hub genes were identified with a cutoff value degree > 20.

### 2.6. miRNA of Target mRNA and Target circRNA of miRNA Prediction

The miRNAs targeting hub genes were screened using the Starbase online database (http://starbase.sysu.edu.cn/) with criteria, including clipExpNum = 1, pancancerNum = 1, and program = 1. Moreover, the target circRNAs of miRNAs were identified using miRanda online software with a mirSVR score. The lower the score presented, the more reliable the binding site.

### 2.7. circRNA-miRNA-mRNA (ceRNA) Network Construction

The hypergeometric distribution test was used to assess whether target miRNAs of DEGs were enriched in the miRNA set of circRNAs. Then, the circRNA-miRNA-mRNA network was constructed and visualized using Cytoscape based on the hypergeometric distribution test; *p* < 0.05.

### 2.8. Patients and Tissue Samples

Cancer tissues and adjacent tissues of 5 patients with NSCLC who were surgically treated in the Third Affiliated Hospital of Zunyi Medical University from September 2019 to March 2020 were collected. All patients were diagnosed with NSCLC by histopathological examination and did not receive radiotherapy and chemotherapy before the operation. After collection, the specimens were frozen in liquid nitrogen for later use. The study was approved by the Ethics Committee of the Third Affiliated Hospital of Zunyi Medical University, and written informed consent was obtained from all patients or their guardians.

### 2.9. RNA Isolation and Quantitative Real-Time Polymerase Chain Reaction (qRT-PCR)

All cancer tissues and adjacent tissues were lysed with TRIzol Reagent (Life Technologies-Invitrogen, Carlsbad, CA, USA), and total RNA was isolated following the manufacturer's instructions. Then, the concentration and purity of the RNA solution were quantified using a NanoDrop 2000FC-3100 nucleic acid protein quantifier (Thermo Fisher Scientific, Waltham, MA, USA Life Real). The extracted RNA was reverse-transcribed to cDNA using the SureScript First-Strand cDNA Synthesis Kit (Genecopoeia, Guangzhou, China) prior to qRT-PCR. The qRT-PCR reaction consisted of 3 *μ*l of the reverse transcription product, 5 *μ*l of 5x BlazeTaq qPCR Mix (Genecopoeia, Guangzhou, China), and 1 *μ*l of forward and reverse primer each. PCR was performed in a Bio-Rad CFX96 Touch™ PCR detection system (Bio-Rad Laboratories, Inc., USA) under the following conditions: initial denaturation at 95°C for 1 min, followed by 40 cycles that involved incubation at 95°C for 20 s, 55°C for 20 s, and 72°C for 30 s. The forward primer of LPCAT1 was “ACCTGCCTAATTACCTTCAAAC,” and the reverse primer of LPCAT1 was “TCCGCAATACCTATCTTCTCTC.” The forward primer of SERPINE1 was “GACTCCCTTCCCCGACTCCA,” and the reverse primer of SERPINE1 was “CGGTCATTCCCAGGTTCTCT.” The forward primer of STC2 was “GTGGGGTGTGGCGTGTTT,” and the reverse primer of STC2 was “TGGGAGGCTTCTGGATGG.” The forward primer of *β*-actin was “TCCCTGGAGAAGAGCTATGA,” and the reverse primer of *β*-actin was “AGGAAGGAAGGCTGGAAAAG.” All primers were synthesized by Servicebio (Servicebio, Wuhan, China). The *β*-actin gene served as an internal control, and the relative expression of 3 hub genes was determined using the 2^-*ΔΔ*Ct^ method [25]. The experiment was performed in triplicate on independent occasions. Statistical differences in the 3 hub genes between the control and NSCLC samples were detected by paired *t*-tests, using GraphPad Prism V6 (GraphPad Software, La Jolla, CA, USA), and the level of statistical significance was tested and presented as ^∗^*p* < 0.05.

### 2.10. Statistical Analysis

The different expression of hub genes simultaneously regulated by multiple circRNAs was determined between standard samples and NSCLC samples of pathologic stages using the one-way ANOVA. Survival curves were performed using the survival R package to assess the association between variables and survival. Candidate variables with *p* < 0.05 were included in the multivariate model. Independent prognostic factors were determined by stepwise regression of multivariate Cox analysis based on the Akaike Information Criterion (AIC) value. The risk score was calculated and normalized using multivariate Cox models. The pROC-R package was then used to create the ROC curve and calculate the AUC and optimal threshold values based on the risk score. NSCLC samples were divided into a high-risk group and a low-risk group. Nomograms of the median survival time and 3-/5-year survival probability were constructed based on multivariate Cox models.

## 3. Results

### 3.1. Data Selected and Patients' Characteristics

**Figure 1:**
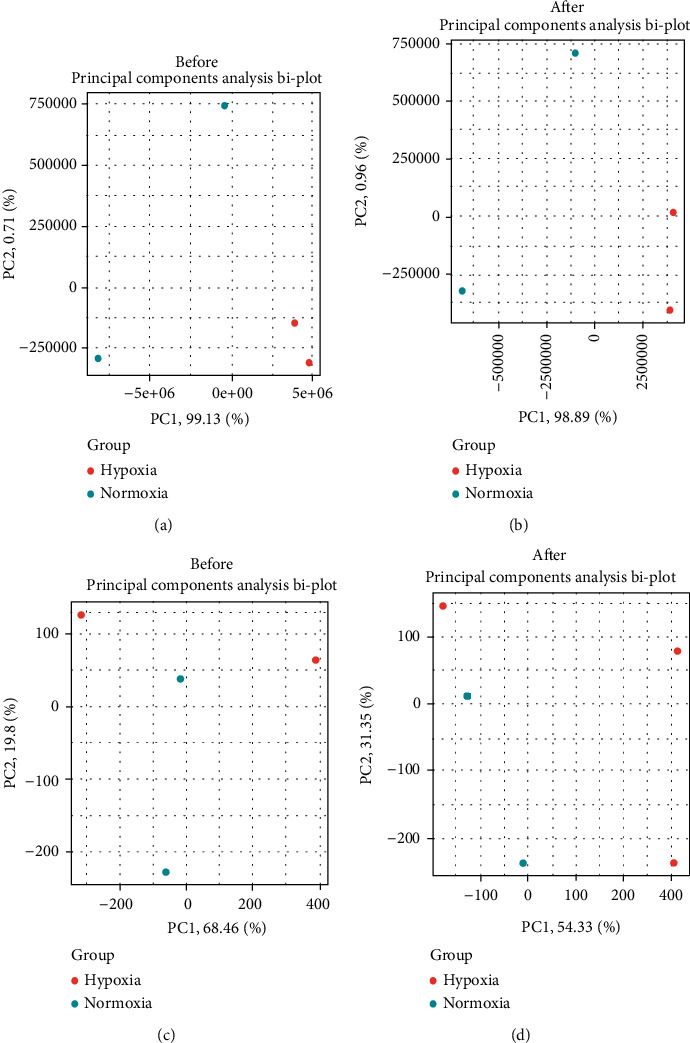
Data processing. (a, b) The principal component analysis biplot of the transcriptome expression profiler between hypoxia-treated A549 cells and normoxia-treated A549 cells. (c, d) The principal component analysis biplot of circRNA expression profiler between hypoxia-treated A549 cells and normoxia-treated A549 cells.

The circRNA expression profiles and transcriptome expression profiles were obtained from the GSE131378 dataset. To avoid the system error and verify the reliable data for the subsequent analysis, the data were assessed by PCA. The results showed that differences between groups were more remarkable than those within groups before and after data normalization (Figures 1(a)–1(d)).

### 3.2. Identification of DEGs and DEcircRNAs in NSCLC Cells

**Figure 2:**
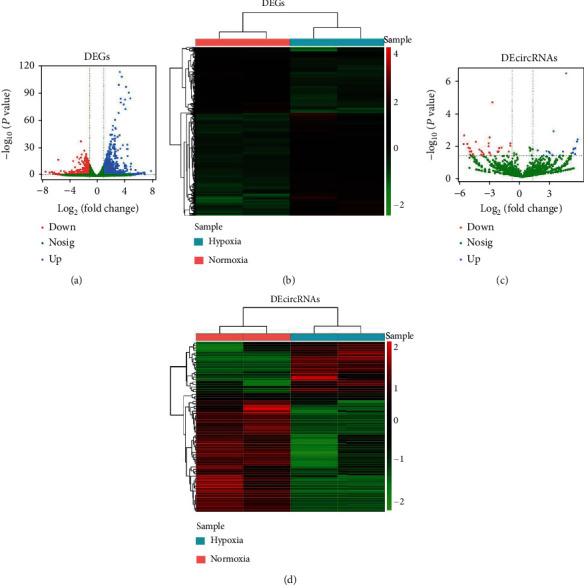
Identification of DEGs and DEcircRNAs in NSCLC cells. (a) Volcano plots of DEGs between hypoxia-treated A549 cells and normoxia-treated A549 cells. (b) Heat map of DEGs between hypoxia-treated A549 cells and normoxia-treated A549 cells. (c) Volcano plots of DEcircRNAs between hypoxia-treated A549 cells and normoxia-treated A549 cells. (d) Heat map of DEcircRNAs between hypoxia-treated A549 cells and normoxia-treated A549 cells.

A total of 2039 DEGs and 70 DEcircRNAs were screened by the DEseq2 R package using the criterion |log2 (FC)| > 1 and *p* < 0.05. The volcano plot map showed significant differences and distribution of the fold change in DEGs (Figure 2(a)) and DEcircRNAs (Figure 2(c)). The heat map illustrated that there were 1293 upregulated DEGs and 746 downregulated DEGs in hypoxia-treated A549 cells compared to normoxia-treated A549 cells (Figure 2(b), Table S1). Moreover, there were 21 upregulated DEcircRNAs and 49 downregulated DEcircRNAs in hypoxia-treated A549 cells compared to normoxia-treated A549 cells, as shown in Figure 2(d) (Table S2).

### 3.3. GO and KEGG Functional Enrichment Analyses of DEGs

**Figure 3:**
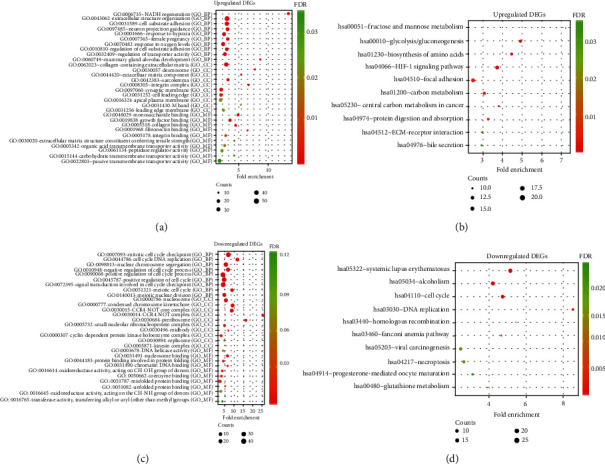
GO and KEGG functional enrichment analyses of DEGs. (a, c) The bubble plot exhibited the top 30 GO terms (top 10 BP, CC, and MF) of the upregulated/downregulated DEGs. (b, d) The bubble plot exhibited the top 10 KEGG pathways of the upregulated/downregulated DEGs.

To further explore the function of DEGs, GO enrichment, including BP term, CC term, and MF term, was analyzed. We found that 284 GO and 42 KEGG pathways were significantly enriched by the upregulated DEGs (Tables S3 and S4). Furthermore, 184 GO and 25 KEGG pathways were significantly enriched by the downregulated DEGs (Tables S5 and S6). As shown in Figure 3(a), the first 30 GO terms for upregulation of DEGs included NADH regeneration, extracellular structural tissue, cell-substrate adhesion, neuronal projection guidance, response to hypoxia, collagen-containing extracellular matrix, desmosomes, extracellular matrix components, myositis, integrin complex, monosaccharide binding, growth factor binding, collagen binding, fibronectin-binding, integrin binding, splinter cell cycle checkpoint, cell cycle DNA replication, nuclear chromosome segregation, DNA integrity checkpoint, negative regulation of cell cycle processes, nucleosome, condensed chromosome kinesis, noncore complex, ribosome, DNA helicase activity, nucleosome binding, protein binding involved in protein folding, chromatin DNA binding, oxidoreductase activity, and CH-OH group acting on the donor (Figure 3(c)). Besides, the 10 KEGG pathways of the upregulated DEGs were as follows: fructose- and mannose-rich metabolism, glycolysis/glycogenesis, biosynthesis of amniotic acid, HIF-1 signaling pathway, focal adhesion, carbon metabolism, carbon metabolism in cancer, protein digestion and absorption, ECM-receptor interaction, and bile secretion (Figure 3(b)). The top 10 KEGG pathways for the downregulated DEGs were as follows: systemic lupus erythematosus-rich, alcoholism, cell cycle, DNA replication, homologous recombination, Fanconi anemia pathway, viral carcinogenesis, necrotizing disease, progesterone-mediated oocyte maturation, and glutathione metabolism (Figure 3(d)).

### 3.4. Function Analysis of DEGs by GSEA

**Figure 4:**
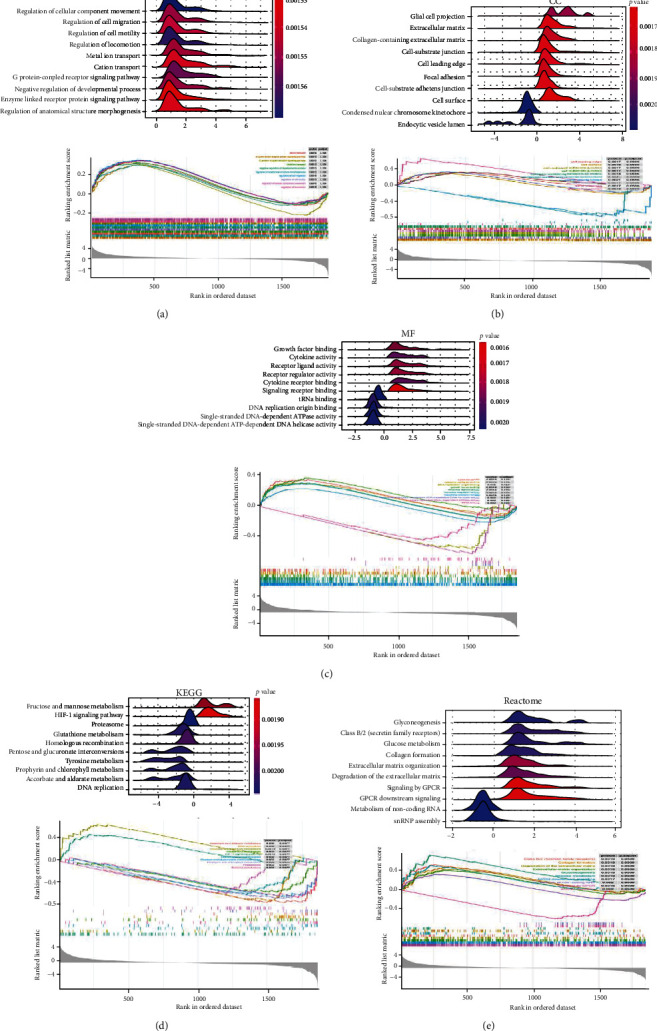
Function analysis of DEGs by GSEA. (a–c) The GSEA plots of GO terms (BP, CC, and MF) of DEGs. (d) The GSEA plots of KEGG enrichment of DEGs. (e) The GSEA plots of Reactome enrichment of DEGs.

To further investigate the function of DEGs, the DEGs were assessed by GSEA with a threshold of |log2 (FC)| > 1 and *p* < 0.05. The results of GSEA showed that the top 30 rich GO terms in the DEGs with the highest enrichment score included enzyme-linked receptor protein signaling pathway, regulation of anatomical structure morphogenesis, cation transport, metal ion transport, regulation of cell motility, cell surface, extracellular matrix, cell-substrate junction, cell-substrate adherens junction, focal adhesion, signaling receptor activity, receptor regulator activity, receptor-ligand activity, growth factor binding, and cytokine receptor binding (Figures 4(a)–4(c)). Ten KEGG pathways were enriched in the HIF-1 signaling pathway, fructose and mannose metabolism, homologous recombination, pentose and glucuronate interconversions, tyrosine metabolism, glutathione metabolism, DNA replication, ascorbate, and alternate metabolism, porphyrin, and chlorophyll metabolism, proteasome (Figure 4(d)). Moreover, the top 10 Reactome terms were enriched in signaling by GPCR, GPCR downstream signaling, extracellular matrix organization, degradation of the extracellular matrix, class B/2 (secretin family receptors), glucose metabolism, collagen formation, gluconeogenesis, snRNP assembly, and metabolism of noncoding RNA (Figure 4(e)).

### 3.5. PPI Network Construction and Hub Gene Identification

**Figure 5:**
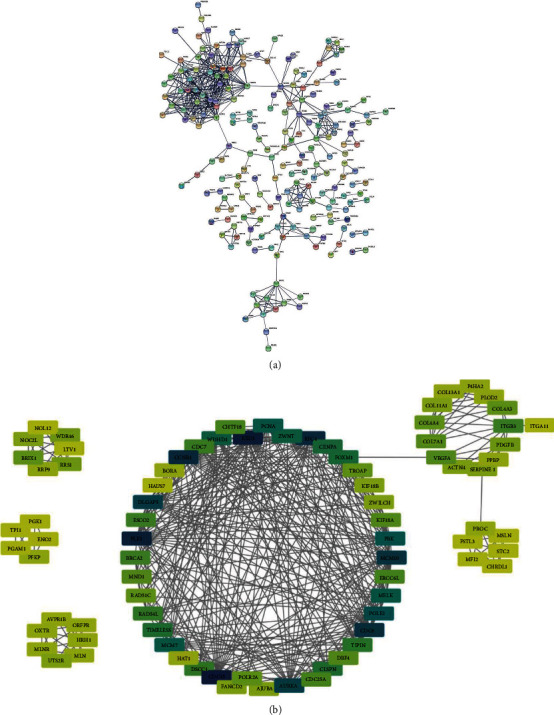
PPI network construction and hub gene identification. (a) The PPI network of 206 DEGs. (b) The hub module with a threshold connectivity degree > 5, and 10 hub genes with a threshold connectivity degree > 20.

We analyzed the interaction between 2039 DEGs and identified the hub genes by constructing a PPI network. The unconnected nodes were removed, and the PPI network included 206 nodes, and 551 edges were constructed (Figure 5(a)). The following hub genes in the PPI network with connectivity degree > 20 were identified: CDC45, EXO1, PLK1, RFC4, CCNB1, CDC6, MCM10, DLGAP5, AURKA, and POLE2. Moreover, 6 hub connective modules in the PPI network were identified with connectivity degree > 5, and 10 hub genes were located in the most extensive module of the six modules (Figure 5(b)).

### 3.6. ceRNA Network Construction

**Figure 6:**
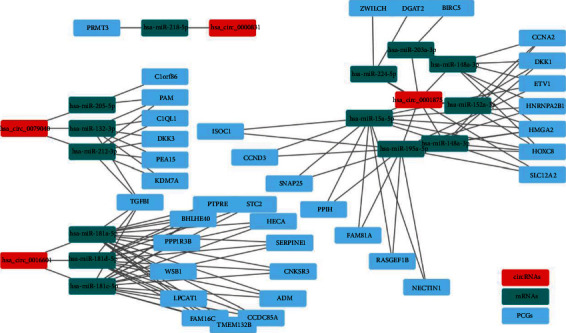
The ceRNA network construction. The ceRNA network included 4 circRNA nodes, 14 miRNA nodes, and 38 mRNA nodes. Red color represents circRNA nodes, green color represents miRNA nodes, and blue color represents mRNA nodes.

The ceRNA network was constructed to perform further synthetic analysis of the role of hub genes and circRNAs. Firstly, the miRNAs targeting the hub DEGs of 6 hub modules were predicted (Table 1). We then predicted the miRNAs targeting the DEcircRNAs (Table 2). After identifying target miRNAs of DEGs, the miRNA set of circRNAs was determined by the hypergeometric distribution test. The ceRNA network was constructed based on the intersection of hub DEGs and circRNAs, including 4 circRNA nodes, 14 miRNA nodes, and 38 mRNA nodes in hypoxia-treated A549 cells (Figure 6).

### 3.7. Correlation between Hub Genes and Clinicopathological Characteristics

**Figure 7:**
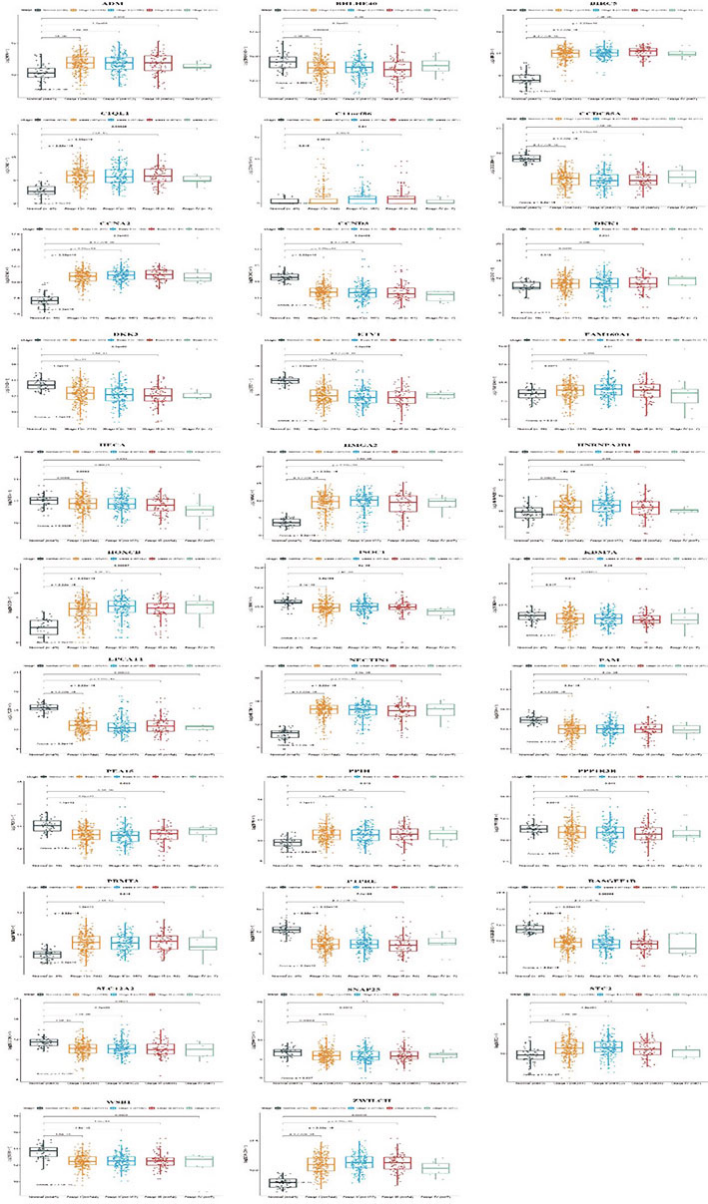
Correlation between hub genes and clinicopathological characteristics. The association between hub gene expression levels and clinical stage.

**Figure 8:**
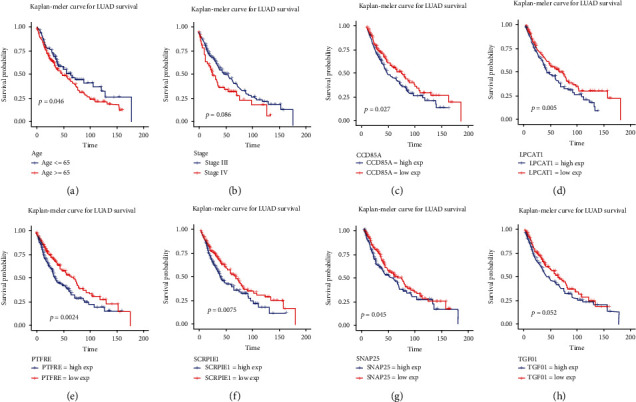
Overall survival analysis of the hub genes, age, and clinical stage in NSCLC patients.

We further tested the expression of hub DEGs between standard samples and NSCLC samples from TCGA database. The results showed that the expression of ADM, BIRC5, C1QL1, CCNA2, DKK1, FAM160A1, HMGA2, HNRNPA2B1, HOXCB, NECTIN1, PPIH, PRMT3, STC2, and ZWILCH was significantly increased in NSCLC samples compared to standard samples (Figure 7). Inversely, the expression of BHLHE40, C11orf86, CCDC85A, CCND3, DKK3, ETV1, HECA, ISOC1, KDM7A, LPCAT1, PAM, PEA15, PPP1R3B, PTPRE, RASGEF1B, SLC12A2, SNAP25, and WSB1 was strongly decreased in NSCLC samples compared to normal samples (Figure 7). Furthermore, other hub DEGs showed no significant difference between NSCLC samples and normal samples (Supplementary Figure 1). Furthermore, the correlation between hub DEGs and NSCLC patients was analyzed, and the results indicated that high expression of CCDC85A, LPCAT1, PTPRE, SERPINE1, SNAP25, and TCFB1 was correlated with more prolonged survival (Figures 8(c)–8(h)). Besides, clinical features, including age and pathological stages, significantly affected the survival. Patients aged less than 65 years showed more prolonged survival, and patients diagnosed in the I/II stage showed more prolonged survival than those diagnosed in the III/IV stage (Figures 8(a) and 8(b)). However, gender and other hub expression did not significantly affect NSCLC (Supplementary Figure 2).

### 3.8. Nomogram Construction and Validation

**Figure 9:**
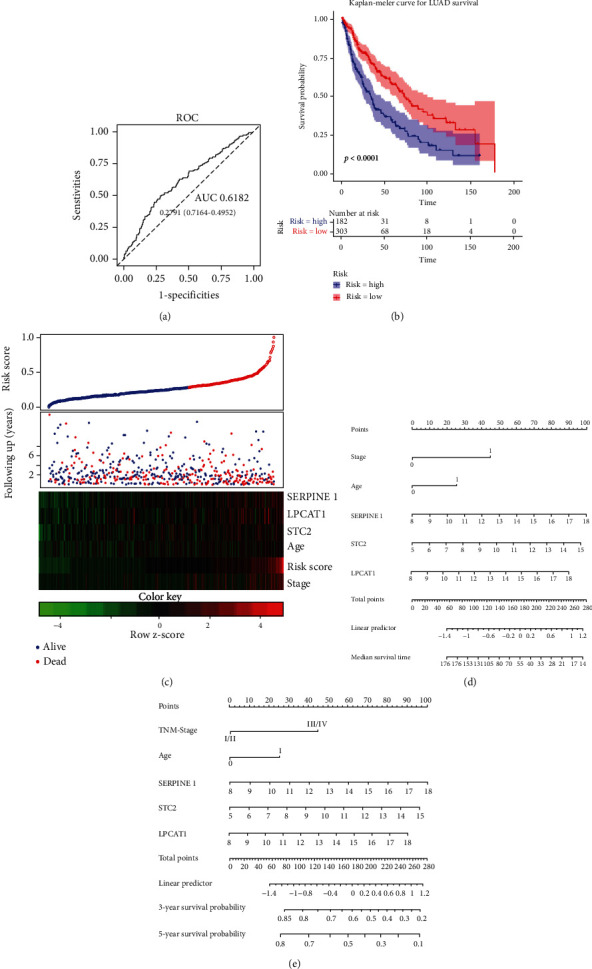
Nomogram construction and validation. (a) The ROC curve for risk prediction. (b) Survival curve for the high-risk score and low-risk score groups. (c) Top figure: the risk score curve. Middle figure: the survival status map. Bottom figure: the heat map of prognostic risk factors (SERPINE1, LPCAT1, STC2, clinical stage, age, and risk score) levels. (d) Nomogram for the median survival time prediction of NSCLC patients. (e) Nomogram for the 3-year and 5-year survival probability prediction in NSCLC patients.

Candidate variables with a value < 0.1 on univariate analysis were included in the multivariable model (Table 3). An ROC curve was drawn using the pROC R package; the AUC value was 0.62, and the optimal threshold was 0.28 (Figure 9(a)). NSCLC samples were grouped into a high-risk group and a low-risk group based on the risk score. Moreover, the survival rate showed a significant difference between the high-risk and low-risk groups, and the high-risk group revealed shorter 5-year survival rates (Figure 9(b)). NSCLC patients were divided into high-risk score and low-risk score groups based on the median risk score, and the number of NSCLC patients with high-risk scores was less than the number of NSCLC patients with low-risk scores (Figure 9(c), top figure). The survival status map showed that the survival rate in the high-risk and low-risk groups declined with time, and most patients in the high-risk score group showed short survival of less than 5 years (Figure 9(c), central figure). Moreover, the heat map indicated that high expression levels of SERPINE1, STC2, and LPCAT1, clinical stage, and age of patients were significantly associated with high-risk scores (Figure 9(c), bottom figure). Moreover, prognostic nomograms were established based on the risk factors in multivariate analysis, and the median survival time, 3-year survival probability, and 5-year survival probability were predicted according to the nomograms (Figure 9(e)).

### 3.9. Validation of the Expression of 3 Hub Genes in NSCLC Patients

**Figure 10:**
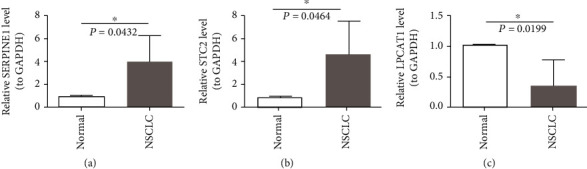
Altering expression of 3 hub mRNAs in NSCLC. qRT-PCR analysis of (a) SERPINE1, (b) STC2, and (c) LPCAT1 expression in 5 pairs of NSCLC samples, ^∗^*p* < 0.05, data are shown as the mean ± SD.

We further validated the expression of the 3 above-mentioned hub genes in clinical tissues using qRT-PCR. High expression of SERPINE1 and STC2 in lung cancer tissues of NSCLC patients (*n* = 5) and low expression of LPCAT1 in the NSCLC group were confirmed at the mRNA level compared to those in the normal lung tissues (*n* = 5) (Figure 10).

## 4. Discussion

Hypoxia is a typical feature of NSCLC. The molecular mechanisms involved in NSCLC have not yet been completely clarified. In this study, we identified circRNAs using the expression profiling of hypoxia-treated NSCLC cells and constructed the ceRNA regulatory network to further reveal the role of hypoxia in NSCLC. Additionally, we explored the potential hypoxia-related biomarkers for predicting the prognosis of NSCLC patients.

On comparing the expression profiling between hypoxia-treated and nontreated NSCLC cells, we found 2039 DEGs. To investigate the biological functions of these DEGs, we performed GO, KEGG, and GSEA analyses. We found that the upregulated DEGs were significantly enriched in response to hypoxia and the HIF-1 signaling pathway, demonstrating that hypoxia did play an important role in NSCLC. Also, the metastasis-associated protein 2 promotes the metastasis of NSCLC by regulating the ERK/AKT and vascular endothelial growth factor (VEGF) signaling pathways [26]. By regulating the miR-29c/vascular endothelial growth factor (VEGF) signaling pathway, PVT1 promotes angiogenesis in non-small-cell lung cancer (NSCLC) [27]. Thus, these hypoxia-related DEGs may also regulate the progression of NSCLC via the ERK/AKT and VEGF pathways.

Furthermore, we explored the interactions of these DEGs and identified ten hub genes, including LOXL2, STC2, SERPINE1, SLC2A3, PFKFB4, EGLN3, NDRG1, MT1X, PGK1, and ADM. LOXL2 is associated with the progression of lung adenocarcinoma [28] and poor prognosis of NSCLC [29]. Mir-504 can also inhibit the proliferation and invasion of NSCLC cells by targeting LOXL2 [30]. Stc2/Jun/Axl signal activation can mediate the acquired resistance of lung cancer patients to EGFR tyrosine kinase inhibitors [31]. Serpine1 is upregulated in mesenchymal lung cancer cells and promotes cell invasion [32]. Abnormal serpine1 DNA methylation participates in EMT of ovarian cancer induced by carboplatin [33]. Also, serpine1, as an oncogene of gastric adenocarcinoma, promotes tumor cell proliferation, migration, and invasion by regulating EMT [34]. Slc2a3 promotes glycolysis and provides energy for gastric cancer cell proliferation [35]. Pfkfb4 is a biomarker for predicting the poor prognosis of gastric cancer patients [36]. It can promote lung adenocarcinoma progression through phosphorylation and activation of transcription coactivator SRC-2 [37]. Egln2 DNA methylation and expression interact with HIF1A to affect the survival of early NSCLC [38]. Increased expression of NDRG1 in NSCLC is associated with advanced T stage and inadequate angiogenesis [39]. Mtlx can promote the migration and invasion of spc-a-1sci and PC-9 lung cancer cell lines [40]. GBP1 promotes erlotinib resistance in NSCLC through the PGK1-activated EMT signaling pathway [41]. Rab11-FIP2 inhibits the growth of NSCLC by regulating the ubiquitination of PGK1 [42]. Thus, these essential genes can affect the development of NSCLC by affecting the proliferation, migration, invasion, and drug resistance of cancer cells and then affect the survival and prognosis of NSCLC patients.

Meanwhile, we identified XX DEcircRNAs between hypoxia-treated and nontreated NSCLC cells and constructed a ceRNA regulatory network, including 4 circRNAs, 14 miRNA, and 38 mRNAs. These four circRNAs may act as endogenous sponges of the corresponding miRNAs to regulate the expressions of corresponding mRNAs to further affect NSCLC. Moreover, we found that CCDC85A, LPCAT1, PTPRE, SERPINE1, SNAP25, and TCFB1 in the ceRNA network were significantly correlated with NSCLC patients' survival on univariate Cox analysis. To further obtain more robust biomarkers for predicting the prognosis of NSCLC patients, we performed multivariate Cox analysis and found that SERPINE1, STC2, and LPCAT1 were independent risk factors for NSCLC. Serpine1 is the most closely related gene with invasion, which can be used to evaluate the importance in the invasion and metastasis of NSCLC [43]. Stc2 can be used as an independent prognostic factor to predict the overall survival rate of NSCLC patients [44]. The acquired resistance of lung cancer patients to EGFR tyrosine kinase inhibitors is mediated by reactivation of the stc2/Jun/Axl signal [31]. Lpcat1 promotes brain metastasis of lung adenocarcinoma by upregulating the PI3K/Akt/myc pathway [45].

Finally, we constructed a related risk score that divided NSCLC patients into high- and low-risk groups to accurately predict their clinical outcomes based on the features of SERPINE1, STC2, and LPCAT1.

In conclusion, for the first time, we constructed the ceRNA regulatory network in NSCLC associated with hypoxia and identified SERPINE1, STC2, and LPCAT1 as potential prognostic biomarkers for NSCLC. Further fundamental *in vitro* and *in vivo* experiments are needed to provide more solid evidence for our study. Our findings increase our knowledge of the molecular mechanisms involved in hypoxia-related NSCLC and guide the discovery of new therapies for NSCLC.

## Figures and Tables

**Table 1:** The top 10 upstream miRNAs targeted with hub DEGs of 6 hub modules were predicted using Starbase online software.

Gene symbol	Ensembl gene ID	miRNA targets
LOXL2	ENSG00000134013	hsa-miR-29c-3p, hsa-miR-29b-3p, hsa-miR-26a-5p, hsa-miR-1297, hsa-miR-26b-5p, hsa-miR-425-5p, hsa-miR-29a-3p
STC2	ENSG00000113739	hsa-miR-488-3p, hsa-miR-181b-5p, hsa-miR-181a-5p, hsa-miR-613, hsa-miR-376c-3p, hsa-miR-410-3p, hsa-miR-140-5p, hsa-miR-1-3p, hsa-miR-24-3p, hsa-miR-181c-5p, hsa-miR-181d-5p, hsa-miR-206, hsa-miR-204-5p, hsa-miR-542-3p
SERPINE1	ENSG00000106366	hsa-miR-30e-5p, hsa-miR-30c-5p, hsa-miR-199a-5p, hsa-miR-181b-5p, hsa-miR-181a-5p, hsa-miR-148b-3p, hsa-miR-152-3p, hsa-miR-181c-5p, hsa-miR-181d-5p, hsa-miR-30a-5p, hsa-miR-148a-3p, hsa-miR-30b-5p, hsa-miR-30d-5p, hsa-miR-224-5p
SLC2A3	ENSG00000059804	hsa-miR-92b-3p, hsa-miR-181b-5p, hsa-miR-181a-5p, hsa-miR-194-5p, hsa-miR-107, hsa-miR-146b-5p, hsa-miR-148b-3p, hsa-miR-26a-5p, hsa-miR-16-5p, hsa-miR-15a-5p, hsa-miR-1297, hsa-miR-92a-3p, hsa-miR-203a-3p, hsa-miR-195-5p, hsa-miR-497-5p, hsa-miR-152-3p, hsa-miR-301a-3p, hsa-miR-181c-5p, hsa-miR-181d-5p, hsa-miR-4262, hsa-miR-216a-5p, hsa-miR-26b-5p, hsa-miR-103a-3p, hsa-miR-15b-5p, hsa-miR-367-3p, hsa-miR-146a-5p, hsa-miR-148a-3p, hsa-miR-25-3p, hsa-miR-182-5p, hsa-miR-32-5p, hsa-miR-363-3p, hsa-miR-542-3p, hsa-miR-424-5p
PFKFB4	ENSG00000114268	hsa-miR-186-5p, hsa-miR-137, hsa-miR-92b-3p, hsa-miR-214-3p, hsa-miR-16-5p, hsa-miR-15a-5p, hsa-miR-92a-3p, hsa-miR-195-5p, hsa-miR-497-5p, hsa-miR-23a-3p, hsa-miR-128-3p, hsa-miR-15b-5p, hsa-miR-367-3p, hsa-miR-25-3p, hsa-miR-23b-3p, hsa-miR-32-5p, hsa-miR-363-3p, hsa-miR-424-5p
EGLN3	ENSG00000129521	hsa-miR-9-5p, hsa-miR-202-3p, hsa-miR-130a-3p, hsa-miR-17-5p, hsa-miR-20a-5p, hsa-miR-454-3p, hsa-miR-301a-3p, hsa-miR-519d-3p, hsa-miR-301b-3p, hsa-miR-130b-3p, hsa-miR-218-5p, hsa-miR-93-5p, hsa-miR-106b-5p, hsa-miR-873-5p, hsa-miR-20b-5p, hsa-miR-106a-5p
NDRG1	ENSG00000104419	hsa-miR-9-5p, hsa-miR-133a-3p, hsa-miR-449a, hsa-miR-449b-5p, hsa-miR-1271-5p, hsa-miR-133b, hsa-miR-182-5p, hsa-miR-96-5p
MT1X	ENSG00000187193	hsa-miR-376a-3p, hsa-miR-376b-3p
PGK1	ENSG00000102144	hsa-miR-19a-3p, hsa-miR-19b-3p, hsa-miR-217, hsa-miR-143-3p, hsa-miR-96-5p, hsa-miR-873-5p, hsa-miR-4770
ADM	ENSG00000102144	hsa-miR-92b-3p, hsa-miR-181b-5p, hsa-miR-181a-5p, hsa-miR-26a-5p, hsa-miR-1297, hsa-miR-92a-3p, hsa-miR-495-3p, hsa-miR-539-5p, hsa-miR-410-3p, hsa-miR-365a-3p, hsa-miR-144-3p, hsa-miR-181c-5p, hsa-miR-181d-5p, hsa-miR-4262, hsa-miR-26b-5p, hsa-miR-367-3p, hsa-miR-4465, hsa-miR-25-3p, hsa-miR-32-5p, hsa-miR-363-3p

**Table 2:** The target miRNA of circRNA and mirSVR using miRanda online software.

Seq1	Seq2	Tot. score	Tot. energy	Max. score	Max. energy	Strand	Len1	Len2	Positions	CircID
hsa-let-7a-5p	chr3_27424643_27493989	153	-15.03	153	-15.03	13	22	3158	1150	NA
hsa-let-7a-5p	chr3_27453132_27465643	153	-15.03	153	-15.03	14	22	616	432	hsa_circ_0002901
hsa-let-7a-5p	chr3_27453132_27493989	153	-15.03	153	-15.03	15	22	1334	1150	hsa_circ_0064622
hsa-let-7a-5p	chr3_142188178_142279296	156	-22.80	156	-22.80	69	22	5203	2344	NA
hsa-let-7a-5p	chr3_195605123_195615477	161	-24.17	161	-24.17	102	22	1274	833	hsa_circ_0001377
hsa-let-7a-5p	chr12_69210591_69218431	154	-18.99	154	-18.99	140	22	349	301	hsa_circ_0027491
hsa-let-7a-5p	chr12_88542064_88570096	154	-17.14	154	-17.14	147	22	1241	648	hsa_circ_0027689
hsa-let-7a-5p	chr12_109046047_109048186	162	-23.27	162	-23.27	162	22	251	146	hsa_circ_0000437
hsa-let-7a-5p	chr12_116434280_116435035	152	-16.58	152	-16.58	168	22	427	301	hsa_circ_0002748
hsa-let-7a-5p	chr1_24840803_24841057	159	-19.05	159	-19.05	294	22	254	181	hsa_circ_0003553

**Table 3:** Univariate and multivariate analyses of the overall survival in TCGA dataset.

Row names	Single coefficient	HR (95% CI for HR)	Wald test	Single *z*	Single *p* value	Multicoefficient	HR	Multi *z*	Multi *p* value
ADM	-0.0026	1 (0.91-1.1)	0	-0.054	0.96	NA	NA	NA	NA
Age	0.31	1.4 (1-1.9)	4.2	2	0.042	0.3	1.4 (1-1.8)	2	0.051
BHLHE40	0.22	1.3 (1.1-1.5)	8.5	2.9	0.0035	NA	NA	NA	NA
BIRC5	-0.012	0.99 (0.86-1.1)	0.03	-0.18	0.85	NA	NA	NA	NA
C11orf86	0.043	1 (0.97-1.1)	1.3	1.1	0.26	NA	NA	NA	NA
C1QL1	-0.029	0.97 (0.91-1)	0.7	-0.84	0.4	NA	NA	NA	NA
CCDC85A	0.088	1.1 (1-1.2)	4.2	2	0.041	NA	NA	NA	NA
CCNA2	-0.019	0.98 (0.85-1.1)	0.07	-0.26	0.79	NA	NA	NA	NA
CCND3	0.12	1.1 (0.97-1.3)	2.5	1.6	0.12	NA	NA	NA	NA
CNKSR3	-0.015	0.98 (0.86-1.1)	0.05	-0.21	0.83	NA	NA	NA	NA
DGAT2	0.096	1.1 (1-1.2)	3.9	2	0.049	NA	NA	NA	NA
DKK1	0.0076	1 (0.95-1.1)	0.06	0.25	0.8	NA	NA	NA	NA
DKK3	-0.0016	1 (0.89-1.1)	0	-0.027	0.98	NA	NA	NA	NA
ETV1	0.031	1 (0.94-1.1)	0.46	0.68	0.5	NA	NA	NA	NA
FAM160A1	0.058	1.1 (0.94-1.2)	0.95	0.98	0.33	NA	NA	NA	NA
FAM81A	0.05	1.1 (0.95-1.2)	0.94	0.97	0.33	NA	NA	NA	NA
HECA	0.13	1.1 (0.96-1.4)	2.2	1.5	0.14	NA	NA	NA	NA
HMGA2	-0.0095	0.99 (0.94-1)	0.14	-0.38	0.7	NA	NA	NA	NA
HNRNPA2B1	0.089	1.1 (0.88-1.4)	0.67	0.82	0.41	NA	NA	NA	NA
HOXC8	0.0016	1 (0.94-1.1)	0	0.05	0.96	NA	NA	NA	NA
ISOC1	0.041	1 (0.88-1.2)	0.23	0.48	0.63	NA	NA	NA	NA
KDM7A	0.063	1.1 (0.91-1.3)	0.59	0.77	0.44	NA	NA	NA	NA
LPCAT1	0.15	1.2 (1-1.3)	8.3	2.9	0.004	0.11	1.1 (0.99-1.2)	1.9	0.062
NECTIN1	-0.02	0.98 (0.89-1.1)	0.16	-0.4	0.69	NA	NA	NA	NA
PAM	0.1	1.1 (0.95-1.3)	1.7	1.3	0.19	NA	NA	NA	NA
PEA15	0.17	1.2 (0.99-1.4)	3.4	1.8	0.065	NA	NA	NA	NA
PPIH	0.053	1.1 (0.88-1.3)	0.34	0.58	0.56	NA	NA	NA	NA
PPP1R3B	0.04	1 (0.91-1.2)	0.35	0.59	0.56	NA	NA	NA	NA
PRMT3	0.068	1.1 (0.91-1.3)	0.63	0.8	0.43	NA	NA	NA	NA
PTPRE	0.17	1.2 (1-1.4)	5.9	2.4	0.015	NA	NA	NA	NA
RASGEF1B	0.094	1.1 (0.97-1.2)	2.3	1.5	0.13	NA	NA	NA	NA
SERPINE1	0.15	1.2 (1.1-1.3)	11	3.4	8e-04	0.12	1.1 (1-1.2)	2.5	0.012
Sex	-0.19	0.83 (0.6-1.1)	1.3	-1.2	0.25	NA	NA	NA	NA
SLC12A2	0.0078	1 (0.89-1.1)	0.02	0.13	0.9	NA	NA	NA	NA
SNAP25	0.069	1.1 (0.99-1.2)	3.1	1.8	0.078	NA	NA	NA	NA
Stage	0.3	1.3 (1.1-1.7)	6.6	2.6	0.01	0.38	1.5 (1.2-1.8)	3.2	0.0015
Status	21	1*e* + 09 (0-Inf)	0	0.012	0.99	NA	NA	NA	NA
STC2	0.098	1.1 (1-1.2)	4.7	2.2	0.03	0.11	1.1 (1-1.2)	2.4	0.014
TGFBI	0.071	1.1 (0.98-1.2)	2.4	1.6	0.12	NA	NA	NA	NA
TMEM132B	-0.016	0.98 (0.91-1.1)	0.19	-0.43	0.66	NA	NA	NA	NA
WSB1	0.13	1.1 (0.97-1.3)	2.4	1.5	0.12	NA	NA	NA	NA
ZWILCH	-0.034	0.97 (0.81-1.2)	0.14	-0.37	0.71	NA	NA	NA	NA

## Data Availability

The data are obtained from GEO and TCGA databases.

## Supplementary Materials

Supplementary Materials Supplementary Figure S1: the expressions ofCNKSR3, DGAT2, FAMB1A, SERPINE1, TGFB1, and TMEM132B in the ceRNA network showed no significant difference between NSCLC and normal samples. Supplementary Figure S2: no significant difference of survival between groups divided by gender or expression of ADM, BHLHE40, BIRC5, C1QL1, C11orf86, CCNA2, CCND3, CNKSR3, DKK1, DKK3, DGAT2, ETV1, FAM81A, FAM160A1, HECA, HMGA2, HOXC8, ISOC1, KDM7A, NECTIN1, HNRNPA2B1, PAM, PEA15, PPIH, PPP1R3B, RASGEF1B, SLC12A2, ZWILCH, WSB1, TMEM132B, or STC2. Supplementary Table S1: identification of 1293 upregulated DEGs and 746 downregulated DEGs in hypoxia-treated A549 cells compared to normoxia-treated A549 cells displayed in the heat map. Supplementary Table S2: 21 upregulated DEcircRNAs and 49 downregulated DEcircRNAs identified in hypoxia-treated A549 cells compared to normoxia-treated A549 cells. Supplementary Table S3 and S4: upregulated DEGs were significantly enriched into 284 GO terms and 42 KEGG pathways. Supplementary Table S5 and S6: downregulated DEGs were significantly enriched into 184 GO terms and 25 KEGG pathways.
