# Supplementary material for: A Novel Hypoxia-Related Gene Signature with Strong Predicting Ability in Non-Small-Cell Lung Cancer Identified by Comprehensive Profiling
Source: Int J Genomics. 2022 May 19;2022:8594658. doi: 10.1155/2022/8594658 (PMC9135579; doi:10.1155/2022/8594658)
Supplement: Supplementary Materials — Supplementary Figure S1: the expressions ofCNKSR3, DGAT2, FAMB1A, SERPINE1, TGFB1, and TMEM132B in the ceRNA network showed no significant difference between NSCLC and normal samples. Supplementary Figure S2: no significant difference of survival between groups divided by gender or expression of ADM, BHLHE40, BIRC5, C1QL1, C11orf86, CCNA2, CCND3, CNKSR3, DKK1, DKK3, DGAT2, ETV1, FAM81A, FAM160A1, HECA, HMGA2, HOXC8, ISOC1, KDM7A, NECTIN1, HNRNPA2B1, PAM, PEA15, PPIH, PPP1R3B, RASGEF1B, SLC12A2, ZWILCH, WSB1, TMEM132B, or STC2. Supplementary Table S1: identification of 1293 upregulated DEGs and 746 downregulated DEGs in hypoxia-treated A549 cells compared to normoxia-treated A549 cells displayed in the heat map. Supplementary Table S2: 21 upregulated DEcircRNAs and 49 downregulated DEcircRNAs identified in hypoxia-treated A549 cells compared to normoxia-treated A549 cells. Supplementary Table S3 and S4: upregulated DEGs were significantly enriched into 284 GO terms and 42 KEGG pathways. Supplementary Table S5 and S6: downregulated DEGs were significantly enriched into 184 GO terms and 25 KEGG pathways. [file 8594658.f1.zip › Supplementary 1_DEmRNA_sig.pdf]

Supplementary Table S1. Identification of 1,293 up-regulated DEGs and 746 downregulated DEGs in hypoxia treated A549 cells compared to normoxia treated A549 cells displayed in the heatmap.

GeneID	baseMean	logFC	lfcSE	stat	PValue	FDR	Gene.symb
LOXL2	24775.91	3.320493	0.145638	22.79956	4.63E-115	8.50E-111	LOXL2
STC2	6074.721	3.583185	0.160982	22.2583	9.37E-110	8.60E-106	STC2
SERPINE1	24937.55	3.139819	0.147257	21.32204	7.09E-101	4.33E-97	SERPINE1
CCN5	3714.777	4.177057	0.198131	21.08235	1.16E-98	5.30E-95	CCN5
ANGPTL4	6691.107	4.564611	0.223936	20.38353	2.34E-92	8.59E-89	ANGPTL4
PPFIA4	1058.473	4.779374	0.243255	19.64757	6.07E-86	1.85E-82	PPFIA4
LOC10537	1662.816	3.41019	0.173949	19.60452	1.41E-85	3.71E-82	LOC10537
SLC2A3	25696.85	4.0488	0.207795	19.4846	1.48E-84	3.40E-81	SLC2A3
PFKFB4	2880.826	3.424532	0.179196	19.11052	2.06E-81	4.21E-78	PFKFB4
EGLN3	9037.686	3.848216	0.204071	18.85727	2.56E-79	4.70E-76	EGLN3
NDRG1	25375.33	4.099861	0.225095	18.21389	4.00E-74	6.68E-71	NDRG1
MT1X	3006.54	3.110014	0.175977	17.67288	6.78E-70	1.04E-66	MT1X
APOL2	3423.689	2.619098	0.157276	16.65283	2.89E-62	4.07E-59	APOL2
PGK1	158111.8	2.311064	0.146844	15.73824	8.27E-56	1.08E-52	PGK1
MEGF6	2575.486	2.647033	0.171967	15.39272	1.83E-53	2.24E-50	MEGF6
ADM	6747.317	2.742203	0.179701	15.2598	1.42E-52	1.62E-49	ADM
SLC2A1	21660.73	2.242961	0.148801	15.07361	2.42E-51	2.61E-48	SLC2A1
ENO2	6822.095	2.196999	0.153898	14.27571	3.10E-46	3.16E-43	ENO2
BNIP3L	21806.05	1.989857	0.13978	14.23567	5.50E-46	5.31E-43	BNIP3L
PLOD2	51085.58	2.041994	0.143486	14.23134	5.85E-46	5.37E-43	PLOD2
TGFBI	113315	1.954804	0.137588	14.20769	8.21E-46	7.17E-43	TGFBI
BNIP3	5133.186	2.105345	0.148322	14.1944	9.92E-46	8.27E-43	BNIP3
ITGB3	790.7029	3.267696	0.230966	14.14794	1.92E-45	1.53E-42	ITGB3
PLIN2	9789.308	2.553945	0.181917	14.03905	8.99E-45	6.87E-42	PLIN2
TMEM45A	1527.62	2.990699	0.21562	13.87025	9.59E-44	7.04E-41	TMEM45A
SLC16A3	8606.951	2.631786	0.193875	13.57463	5.66E-42	3.99E-39	SLC16A3
VEGFA	5955.596	2.20957	0.164282	13.44982	3.09E-41	2.10E-38	VEGFA
JUN	3525.184	2.435191	0.181371	13.42661	4.22E-41	2.77E-38	JUN
PDK1	2897.68	2.589484	0.197525	13.10963	2.90E-39	1.83E-36	PDK1
ALDH3A1	77183.06	-2.26016	0.173669	-13.0142	1.02E-38	6.21E-36	ALDH3A1
PAM	11857.29	2.030591	0.156561	12.96995	1.81E-38	1.07E-35	PAM
DAPK1	36192.27	1.887651	0.151331	12.47368	1.04E-35	5.96E-33	DAPK1
BARX1	892.0392	2.486441	0.199406	12.46927	1.10E-35	6.10E-33	BARX1
LBH	9347.089	2.044856	0.164536	12.42802	1.84E-35	9.93E-33	LBH
PDGFB	758.2332	2.594425	0.20936	12.39216	2.88E-35	1.51E-32	PDGFB
CXCR4	357.8494	4.37332	0.35449	12.33692	5.73E-35	2.92E-32	CXCR4
SPAG4	883.996	2.376228	0.193364	12.28886	1.04E-34	5.15E-32	SPAG4
LOC10050	542.0282	3.723495	0.304132	12.24304	1.83E-34	8.84E-32	LOC10050
FAM162A	3861.851	1.955859	0.160743	12.16762	4.62E-34	2.17E-31	FAM162A
COL12A1	6207.396	2.481088	0.206993	11.98635	4.19E-33	1.92E-30	COL12A1
DDIT4	30533.34	1.799259	0.151685	11.86179	1.87E-32	8.36E-30	DDIT4
COL4A3	1853.957	2.475968	0.208859	11.85474	2.03E-32	8.88E-30	COL4A3
GAL3ST1	4082.784	2.2909	0.193511	11.83861	2.46E-32	1.05E-29	GAL3ST1
TAGLN	626.3533	2.524685	0.214091	11.79257	4.26E-32	1.78E-29	TAGLN
CDCP1	4153.322	1.829234	0.156652	11.67709	1.67E-31	6.80E-29	CDCP1
TNS1	2126.137	2.296099	0.197026	11.65382	2.19E-31	8.75E-29	TNS1
NKAIN4	644.0005	2.391183	0.207615	11.5174	1.08E-30	4.21E-28	NKAIN4
HK2	764.7874	3.369796	0.292975	11.502	1.29E-30	4.93E-28	HK2
PDLIM7	2856.334	1.823663	0.159371	11.44289	2.55E-30	9.55E-28	PDLIM7
UPK1A	247.8753	4.303675	0.376388	11.43414	2.82E-30	1.04E-27	UPK1A
LOC10537	2574.014	1.902545	0.166468	11.42888	3.00E-30	1.08E-27	LOC10537
ABCG1	4012.749	2.659579	0.232865	11.42113	3.28E-30	1.16E-27	ABCG1
CCN4	259.1165	4.431404	0.388976	11.39248	4.56E-30	1.58E-27	CCN4
STC1	5103.924	2.560237	0.226559	11.30053	1.30E-29	4.43E-27	STC1
FER1L4	1435.249	1.988497	0.176473	11.26799	1.89E-29	6.30E-27	FER1L4
SBK2	494.6632	3.031226	0.272228	11.13488	8.49E-29	2.78E-26	SBK2
INSL4	5507.154	-1.76174	0.159358	-11.0552	2.07E-28	6.66E-26	INSL4

KLF7	5210.613	1.643188	0.150041	10.95159	6.53E-28	2.06E-25	KLF7
SEC14L4	3155.408	1.714396	0.156892	10.92721	8.54E-28	2.66E-25	SEC14L4
P4HA2	7232.649	1.596467	0.146138	10.92441	8.81E-28	2.69E-25	P4HA2
PPP1R3G	412.6681	2.759437	0.253738	10.87515	1.51E-27	4.55E-25	PPP1R3G
TMEM141	5149.813	1.870953	0.172358	10.85503	1.89E-27	5.58E-25	TMEM141
SORCS2	568.3603	2.622662	0.243507	10.77039	4.75E-27	1.38E-24	SORCS2
PDLIM2	2103.031	1.943683	0.181279	10.72204	8.02E-27	2.30E-24	PDLIM2
PPP1R3C	4381.805	2.108603	0.198203	10.63862	1.97E-26	5.56E-24	PPP1R3C
MXI1	2914.864	2.00037	0.188291	10.62383	2.31E-26	6.42E-24	MXI1
SAMD4A	1597.512	1.80692	0.171387	10.54291	5.48E-26	1.50E-23	SAMD4A
COL7A1	20659.76	1.650272	0.15788	10.45273	1.42E-25	3.84E-23	COL7A1
KDM3A	6308.777	1.627035	0.156213	10.41547	2.11E-25	5.53E-23	KDM3A
P4HA1	11903.21	1.582636	0.151952	10.41536	2.11E-25	5.53E-23	P4HA1
C8orf58	1037.65	2.557632	0.246761	10.36481	3.58E-25	9.26E-23	C8orf58
FSTL3	7214.055	1.496706	0.145521	10.28516	8.22E-25	2.09E-22	FSTL3
AK4	3033.541	1.830298	0.178216	10.27014	9.61E-25	2.41E-22	AK4
COL4A4	2726.618	1.825384	0.177776	10.26786	9.84E-25	2.44E-22	COL4A4
PCNA	16010.97	-1.50789	0.147182	-10.245	1.25E-24	3.05E-22	PCNA
TMEM37	2418.23	2.040218	0.199182	10.24299	1.27E-24	3.07E-22	TMEM37
LOXL4	997.984	1.916143	0.188146	10.18432	2.33E-24	5.55E-22	LOXL4
DKK1	22604.19	-2.14486	0.211362	-10.1478	3.39E-24	7.97E-22	DKK1
C14orf132	4174.164	1.563646	0.154506	10.12027	4.49E-24	1.04E-21	C14orf132
PFKFB3	46812.08	1.404112	0.138942	10.10577	5.21E-24	1.19E-21	PFKFB3
HILPDA	9850.353	1.511679	0.149888	10.08536	6.41E-24	1.45E-21	HILPDA
TGFB1I1	1668.001	1.76606	0.17528	10.07566	7.08E-24	1.58E-21	TGFB1I1
MELTF	2916.707	1.583311	0.157322	10.06414	7.96E-24	1.76E-21	MELTF
FAM160A1	493.7464	2.590072	0.257644	10.0529	8.92E-24	1.95E-21	FAM160A1
PFKP	31714.54	1.401897	0.139539	10.04661	9.51E-24	2.05E-21	PFKP
HRH1	1384.934	1.767504	0.178606	9.896121	4.33E-23	9.23E-21	HRH1
F3	829.3261	2.800666	0.284215	9.854034	6.58E-23	1.39E-20	F3
ODC1	4674.474	-1.52007	0.154944	-9.81043	1.02E-22	2.12E-20	ODC1
AXL	17261.46	1.454767	0.14859	9.790506	1.24E-22	2.55E-20	AXL
GBP2	932.7675	2.444362	0.250454	9.759723	1.68E-22	3.42E-20	GBP2
IDH2	6721.176	1.491809	0.152873	9.758511	1.70E-22	3.42E-20	IDH2
CACNA1B	129.4313	4.413218	0.452961	9.743048	1.98E-22	3.94E-20	CACNA1B
MCM10	3422.694	-1.55817	0.161202	-9.66599	4.21E-22	8.29E-20	MCM10
EGFR	34177.49	1.880951	0.195082	9.641836	5.32E-22	1.04E-19	EGFR
ARRDC3	18233.05	1.825278	0.189928	9.610383	7.23E-22	1.40E-19	ARRDC3
IGFBP5	634.5183	2.655307	0.276376	9.607578	7.43E-22	1.42E-19	IGFBP5
APOL1	844.9006	2.740384	0.285457	9.599987	8.00E-22	1.51E-19	APOL1
PLEKHA2	4148.7	1.485966	0.155676	9.545251	1.36E-21	2.54E-19	PLEKHA2
TRNL1	807.9321	-2.76714	0.291962	-9.47775	2.60E-21	4.81E-19	TRNL1
RIMKLA	1471.538	1.812005	0.19219	9.428179	4.17E-21	7.65E-19	RIMKLA
SLC9A2	1626.069	-1.60973	0.171104	-9.40791	5.06E-21	9.19E-19	SLC9A2
CCDC34	2137.255	-1.56602	0.166805	-9.38835	6.09E-21	1.10E-18	CCDC34
ALPK2	12139.18	1.51546	0.161556	9.380374	6.57E-21	1.17E-18	ALPK2
TP63	1031.048	-2.24781	0.240311	-9.35375	8.46E-21	1.49E-18	TP63
QSOX1	7749.702	1.520392	0.163718	9.28664	1.59E-20	2.78E-18	QSOX1
AREG	1580.203	-1.66198	0.179461	-9.26097	2.03E-20	3.51E-18	AREG
LNCSRLR	221.2022	2.951837	0.319	9.253396	2.17E-20	3.73E-18	LNCSRLR
RNR1	2061397	-1.42579	0.154116	-9.25144	2.21E-20	3.76E-18	RNR1
HIF1A-AS2	217.8746	2.872972	0.311651	9.218553	3.01E-20	5.07E-18	HIF1A-AS2
C4orf3	3874.084	1.413288	0.153411	9.212448	3.19E-20	5.32E-18	C4orf3
INHA	248.3697	3.524216	0.38296	9.202568	3.49E-20	5.78E-18	INHA
FUT11	3609.494	1.47636	0.161062	9.16642	4.89E-20	8.01E-18	FUT11
CPLX2	63904.71	-1.69889	0.185756	-9.1458	5.92E-20	9.61E-18	CPLX2
RRS1	1547.911	-1.57244	0.171956	-9.14441	6.00E-20	9.65E-18	RRS1
CRABP2	255.1057	2.609722	0.286241	9.117217	7.71E-20	1.23E-17	CRABP2

UACA	13691.4	1.40303	0.154416	9.08605	1.03E-19	1.62E-17	UACA
TOB1	5418.192	-1.32298	0.146177	-9.05054	1.42E-19	2.23E-17	TOB1
TRNT	362.6988	-2.46858	0.273765	-9.01715	1.93E-19	3.00E-17	TRNT
TIPARP	6360.975	1.31045	0.1456	9.000312	2.25E-19	3.47E-17	TIPARP
GPI	82111.08	1.627989	0.181398	8.974672	2.84E-19	4.34E-17	GPI
HAVCR1	28439.33	1.396642	0.155977	8.954137	3.42E-19	5.19E-17	HAVCR1
COL5A1	8429.99	1.756378	0.196936	8.918516	4.73E-19	7.10E-17	COL5A1
DEGS2	573.6515	2.103657	0.236258	8.904063	5.38E-19	8.03E-17	DEGS2
INSIG2	1637.809	1.477907	0.166127	8.896256	5.78E-19	8.54E-17	INSIG2
SYNGR3	975.2611	1.957999	0.220327	8.886803	6.29E-19	9.23E-17	SYNGR3
SYT12	958.7405	2.140864	0.240977	8.884116	6.44E-19	9.38E-17	SYT12
GRB10	8782.779	1.313897	0.14836	8.85614	8.28E-19	1.20E-16	GRB10
CLSPN	2735.052	-1.42297	0.16093	-8.84219	9.39E-19	1.35E-16	CLSPN
JCAD	2632.566	1.422074	0.160931	8.836529	9.87E-19	1.40E-16	JCAD
SLC6A6	7287.442	1.408463	0.159956	8.805291	1.31E-18	1.84E-16	SLC6A6
EFNA3	448.0329	2.061824	0.234418	8.795516	1.42E-18	1.99E-16	EFNA3
NR5A2	9286.979	1.273314	0.145091	8.775984	1.69E-18	2.35E-16	NR5A2
DDC	103.8189	-5.4986	0.629482	-8.73512	2.43E-18	3.33E-16	DDC
LAMB3	2884.349	1.879627	0.215166	8.735705	2.42E-18	3.33E-16	LAMB3
PHGDH	2306.451	-1.67142	0.192113	-8.70019	3.31E-18	4.50E-16	PHGDH
TPI1	81939.22	1.265825	0.145522	8.698518	3.36E-18	4.53E-16	TPI1
WTIP	1930.225	1.394198	0.161402	8.638064	5.72E-18	7.65E-16	WTIP
BACH1	5886.279	1.386182	0.160663	8.627894	6.25E-18	8.31E-16	BACH1
SDK1	647.7176	1.882202	0.218675	8.607314	7.48E-18	9.87E-16	SDK1
H2BC14	2816.344	-1.56704	0.182199	-8.60066	7.93E-18	1.04E-15	H2BC14
HNRNPA2	29768.81	-1.23936	0.14451	-8.57631	9.80E-18	1.27E-15	HNRNPA2
STEAP3	3550.763	1.742187	0.204076	8.536964	1.38E-17	1.78E-15	STEAP3
PPL	2187.324	1.517647	0.178059	8.523282	1.55E-17	1.99E-15	PPL
SDF2L1	676.7422	-1.7226	0.202158	-8.52105	1.58E-17	2.01E-15	SDF2L1
TCP11L2	3256.05	1.360779	0.15973	8.519256	1.61E-17	2.03E-15	TCP11L2
VLDLR	3626.573	1.650439	0.193982	8.508217	1.77E-17	2.22E-15	VLDLR
DDIAS	1328.207	-1.64648	0.193574	-8.50569	1.81E-17	2.25E-15	DDIAS
DUSP1	12701.46	1.779825	0.210322	8.462387	2.62E-17	3.25E-15	DUSP1
LAMB2	15025.22	1.316253	0.155838	8.446301	3.01E-17	3.70E-15	LAMB2
TSPAN13	2395.32	-1.62844	0.19283	-8.44498	3.04E-17	3.72E-15	TSPAN13
FCGBP	309.7773	-3.35433	0.398065	-8.42659	3.56E-17	4.32E-15	FCGBP
AMBP	360.8446	-2.10842	0.250421	-8.41952	3.78E-17	4.56E-15	AMBP
TNFRSF12/	3970.03	1.634566	0.194216	8.416219	3.89E-17	4.66E-15	TNFRSF12/
PXDN	2574.038	1.485595	0.176699	8.407506	4.19E-17	4.99E-15	PXDN
GBE1	8110.134	1.206233	0.143644	8.3974	4.56E-17	5.40E-15	GBE1
BLM	2936.667	-1.4962	0.178539	-8.38024	5.28E-17	6.21E-15	BLM
PBK	4514.509	-1.38864	0.165727	-8.37907	5.33E-17	6.23E-15	PBK
MAFK	5597.512	1.5658	0.186906	8.377494	5.41E-17	6.28E-15	MAFK
CHAF1B	1478.058	-1.47461	0.176092	-8.37411	5.56E-17	6.42E-15	CHAF1B
PLA2G4A	17119.06	-1.18757	0.14257	-8.32971	8.10E-17	9.29E-15	PLA2G4A
GYS1	5253.57	1.401523	0.168313	8.326885	8.30E-17	9.46E-15	GYS1
MAP7	10280.34	-1.22991	0.148222	-8.29774	1.06E-16	1.20E-14	MAP7
ATP1B1	48415.51	1.385523	0.167167	8.288272	1.15E-16	1.29E-14	ATP1B1
PTPRB	752.2163	2.245407	0.271028	8.284779	1.18E-16	1.32E-14	PTPRB
LZTS3	1378.787	1.519827	0.185354	8.19958	2.41E-16	2.68E-14	LZTS3
EXO1	2674.157	-1.32738	0.161905	-8.19849	2.43E-16	2.69E-14	EXO1
ITGA5	7382.052	1.178154	0.144012	8.18096	2.82E-16	3.09E-14	ITGA5
PGAM1	5976.19	1.209779	0.147931	8.178003	2.89E-16	3.15E-14	PGAM1
CITED2	2138.845	1.870114	0.228895	8.170196	3.08E-16	3.34E-14	CITED2
ANO6	27806.53	1.593086	0.195058	8.167251	3.15E-16	3.40E-14	ANO6
PSD4	3415.849	1.930475	0.236493	8.162932	3.27E-16	3.51E-14	PSD4
MIR210HG	245.7136	2.838988	0.347925	8.15977	3.36E-16	3.58E-14	MIR210HG
DPYSL4	1409.684	1.50951	0.185263	8.147925	3.70E-16	3.93E-14	DPYSL4

TMEM158	226.7455	2.808563	0.344849	8.144331	3.81E-16	4.02E-14	TMEM158
ITGA11	900.4365	1.617539	0.199195	8.120384	4.65E-16	4.87E-14	ITGA11
GPR146	247.687	2.409282	0.297951	8.086158	6.16E-16	6.42E-14	GPR146
ANKZF1	3563.79	1.431566	0.177113	8.082763	6.33E-16	6.56E-14	ANKZF1
UNC13A	1300.806	-1.84667	0.229487	-8.04698	8.49E-16	8.74E-14	UNC13A
SLC25A29	4465.5	1.564797	0.194912	8.028208	9.89E-16	1.01E-13	SLC25A29
PCED1B	6401.912	1.297986	0.161811	8.021603	1.04E-15	1.06E-13	PCED1B
SDAD1P1	515.4594	1.794668	0.223896	8.01563	1.10E-15	1.11E-13	SDAD1P1
GPATCH4	1354.145	-1.39115	0.173982	-7.99592	1.29E-15	1.30E-13	GPATCH4
ARRB2	3354.08	-1.47834	0.185486	-7.9701	1.59E-15	1.59E-13	ARRB2
TNNT1	5296.87	1.228591	0.15436	7.959249	1.73E-15	1.73E-13	TNNT1
AJUBA	9335.307	1.351174	0.169803	7.957311	1.76E-15	1.74E-13	AJUBA
APP	63741.6	1.240437	0.155935	7.954845	1.79E-15	1.77E-13	APP
OPTN	4867.632	1.379002	0.173613	7.942956	1.97E-15	1.94E-13	OPTN
PAK3	435.1821	-1.95678	0.246491	-7.93854	2.05E-15	2.00E-13	PAK3
PRKAR1B	2997.482	1.540204	0.194222	7.930118	2.19E-15	2.12E-13	PRKAR1B
ACOX2	230.5939	-2.55448	0.322486	-7.9212	2.35E-15	2.27E-13	ACOX2
PLK1	3608.211	-1.201	0.151765	-7.91356	2.50E-15	2.40E-13	PLK1
H2AC17	6744.167	-1.27103	0.160731	-7.90781	2.62E-15	2.50E-13	H2AC17
TIMELESS	6562.097	-1.18804	0.150261	-7.90652	2.65E-15	2.52E-13	TIMELESS
DNAH11	399.4468	2.205559	0.279204	7.899455	2.80E-15	2.65E-13	DNAH11
NEDD4	33629.43	1.59475	0.202083	7.891574	2.98E-15	2.81E-13	NEDD4
MIR193BH	416.8106	2.000402	0.253778	7.882481	3.21E-15	3.00E-13	MIR193BH
DHRS13	1009.772	1.509961	0.191718	7.87596	3.38E-15	3.15E-13	DHRS13
NOL3	2518.018	1.447214	0.183905	7.869334	3.57E-15	3.30E-13	NOL3
AURKA	6230.956	-1.16882	0.14856	-7.86764	3.61E-15	3.33E-13	AURKA
KDM7A	2130.712	1.368404	0.17472	7.831974	4.80E-15	4.40E-13	KDM7A
NOTUM	144.2882	3.844736	0.492549	7.805796	5.91E-15	5.40E-13	NOTUM
LDHAP4	658.0394	1.665027	0.213569	7.796217	6.38E-15	5.79E-13	LDHAP4
ELOVL6	1843.568	-1.27087	0.163408	-7.77727	7.41E-15	6.68E-13	ELOVL6
LOC10272	1879.016	1.606705	0.206597	7.776994	7.43E-15	6.68E-13	LOC10272
DNAJB2	2951.852	1.5589	0.200845	7.761704	8.38E-15	7.50E-13	DNAJB2
KYNU	15613.66	-1.1757	0.151595	-7.75553	8.80E-15	7.83E-13	KYNU
FDXR	2606.961	-1.43495	0.185262	-7.7455	9.52E-15	8.44E-13	FDXR
PKM	390483.4	1.175584	0.151995	7.734358	1.04E-14	9.16E-13	PKM
LOC10798	204.2107	2.422409	0.313329	7.731198	1.07E-14	9.35E-13	LOC10798
FOXM1	3803.194	-1.16757	0.151261	-7.71894	1.17E-14	1.02E-12	FOXM1
ZWILCH	3228.038	-1.42167	0.184306	-7.7136	1.22E-14	1.06E-12	ZWILCH
NCL	22630.52	-1.31996	0.171216	-7.7093	1.27E-14	1.09E-12	NCL
PTAFR	213.4321	2.750564	0.356802	7.708927	1.27E-14	1.09E-12	PTAFR
SLC12A2	39315.31	-1.29085	0.167684	-7.69809	1.38E-14	1.18E-12	SLC12A2
FAT3	1104.458	1.561137	0.203883	7.657023	1.90E-14	1.62E-12	FAT3
PADI2	1360.754	1.883892	0.246433	7.64463	2.10E-14	1.78E-12	PADI2
STEAP1	2622.537	-1.33092	0.174229	-7.6389	2.19E-14	1.85E-12	STEAP1
COL11A1	3688.447	1.151672	0.151162	7.618791	2.56E-14	2.15E-12	COL11A1
RHOB	6187.171	1.109525	0.145652	7.617641	2.58E-14	2.16E-12	RHOB
SLC29A4	6751.254	1.532685	0.201263	7.615338	2.63E-14	2.19E-12	SLC29A4
MAFF	454.9996	2.010201	0.264354	7.60419	2.87E-14	2.38E-12	MAFF
H2AC21	5251.623	-1.14927	0.151375	-7.59221	3.14E-14	2.59E-12	H2AC21
HCFC1R1	4837.451	1.177521	0.155096	7.592193	3.15E-14	2.59E-12	HCFC1R1
CCDC183	397.382	2.104622	0.277285	7.590117	3.20E-14	2.62E-12	CCDC183
CAVIN1	34035.44	1.327301	0.174982	7.585359	3.32E-14	2.70E-12	CAVIN1
ERRFI1	16806.91	1.719421	0.226705	7.5844	3.34E-14	2.71E-12	ERRFI1
SEMA5B	82.93721	4.903747	0.64761	7.572074	3.67E-14	2.97E-12	SEMA5B
GABRA5	5837.13	-1.29283	0.170999	-7.56042	4.02E-14	3.23E-12	GABRA5
H2BC6	2888.339	-1.26712	0.16764	-7.55855	4.08E-14	3.26E-12	H2BC6
ANPEP	892.6242	-1.51392	0.200633	-7.54576	4.50E-14	3.58E-12	ANPEP
POLA2	2016.584	-1.26838	0.168103	-7.5453	4.51E-14	3.58E-12	POLA2

CACNG4	750.4431	1.463919	0.194076	7.543019	4.59E-14	3.63E-12	CACNG4
STK17A	1872.502	1.302478	0.172773	7.538669	4.75E-14	3.74E-12	STK17A
WDHD1	3837.209	-1.29176	0.171539	-7.53037	5.06E-14	3.97E-12	WDHD1
CDC20	3627.879	-1.19032	0.158249	-7.5218	5.40E-14	4.21E-12	CDC20
HNRNPH3	2420.764	-1.30542	0.173561	-7.52139	5.42E-14	4.21E-12	HNRNPH3
KDM4B	1786.903	1.265733	0.168316	7.519992	5.48E-14	4.24E-12	KDM4B
H4C1	2936.915	-1.27086	0.169011	-7.51941	5.50E-14	4.24E-12	H4C1
BACE2	3353.61	1.271156	0.169125	7.516076	5.64E-14	4.33E-12	BACE2
CALCOCO	4543.574	1.175586	0.156495	7.511985	5.82E-14	4.45E-12	CALCOCO
GABARAPI	3538.815	1.178723	0.157128	7.501692	6.30E-14	4.79E-12	GABARAPL
ST6GAL2	5075.002	-1.27561	0.170336	-7.48882	6.95E-14	5.27E-12	ST6GAL2
TIPIN	1390.145	-1.3174	0.175972	-7.48642	7.08E-14	5.34E-12	TIPIN
OBSL1	3281.814	1.157778	0.154692	7.484424	7.19E-14	5.40E-12	OBSL1
GJB3	232.2447	2.955456	0.395227	7.47787	7.55E-14	5.66E-12	GJB3
ZWINT	4813.971	-1.11454	0.149132	-7.47356	7.81E-14	5.82E-12	ZWINT
POLR3G	670.2773	-1.8178	0.243494	-7.46547	8.30E-14	6.16E-12	POLR3G
ESCO2	3041.614	-1.36259	0.182545	-7.4644	8.37E-14	6.19E-12	ESCO2
EPHB2	4830.264	1.145152	0.153458	7.462335	8.50E-14	6.26E-12	EPHB2
LIMS2	131.8515	2.96802	0.397823	7.460661	8.61E-14	6.32E-12	LIMS2
FAM13A	4153.069	1.364587	0.183039	7.455174	8.97E-14	6.56E-12	FAM13A
FLNA	203295.7	1.053121	0.141332	7.451414	9.23E-14	6.72E-12	FLNA
FSCN2	1073.634	1.518438	0.203803	7.450525	9.30E-14	6.74E-12	FSCN2
B4GALNT2	180.1686	2.476601	0.332734	7.443192	9.83E-14	7.10E-12	B4GALNT2
NPR1	421.5368	1.847417	0.248785	7.425757	1.12E-13	8.07E-12	NPR1
BIRC5	6225.562	-1.13546	0.152997	-7.42147	1.16E-13	8.30E-12	BIRC5
LOC10192	526.366	2.276448	0.307522	7.402549	1.34E-13	9.53E-12	LOC10192
PDE3A	3203.979	-1.52814	0.206464	-7.40146	1.35E-13	9.56E-12	PDE3A
WSB1	7260.835	1.146043	0.154846	7.401188	1.35E-13	9.56E-12	WSB1
ARHGEF37	1051.468	1.447769	0.195669	7.399075	1.37E-13	9.66E-12	ARHGEF37
ATP10A	1986.117	1.251417	0.169138	7.398799	1.37E-13	9.66E-12	ATP10A
DBF4	2160.732	-1.41323	0.191073	-7.39628	1.40E-13	9.80E-12	DBF4
H1-5	4898.842	-1.44411	0.195315	-7.39377	1.43E-13	9.95E-12	H1-5
RFC3	2740.132	-1.36088	0.184092	-7.3924	1.44E-13	1.00E-11	RFC3
SGO2	2362.995	-1.40414	0.19009	-7.3867	1.51E-13	1.04E-11	SGO2
GLIPR1	3217.373	1.731434	0.234792	7.374338	1.65E-13	1.14E-11	GLIPR1
ORAI3	2652.399	1.339624	0.181694	7.372973	1.67E-13	1.15E-11	ORAI3
CAV1	18542.61	1.100801	0.149441	7.366137	1.76E-13	1.20E-11	CAV1
G6PD	56208.56	-1.06106	0.144278	-7.35429	1.92E-13	1.31E-11	G6PD
GAPDH	437567.3	1.076988	0.146556	7.348659	2.00E-13	1.36E-11	GAPDH
SMAD7	1655.99	1.219366	0.165981	7.346419	2.04E-13	1.38E-11	SMAD7
OAS1	1878.433	-1.3088	0.178353	-7.33822	2.16E-13	1.46E-11	OAS1
FAM219A	1553.633	1.272596	0.173498	7.334948	2.22E-13	1.49E-11	FAM219A
SLC26A9	282.0974	-2.36144	0.322028	-7.33303	2.25E-13	1.51E-11	SLC26A9
FBXL21P	404.9703	2.199603	0.300478	7.320344	2.47E-13	1.65E-11	FBXL21P
NOP16	1353.151	-1.24865	0.171203	-7.29337	3.02E-13	2.01E-11	NOP16
H2AC13	6767.713	-1.26256	0.173392	-7.2815	3.30E-13	2.19E-11	H2AC13
GPAT3	1844.496	-1.31368	0.180778	-7.26681	3.68E-13	2.43E-11	GPAT3
FARSB	3373.111	-1.12068	0.154375	-7.25951	3.88E-13	2.55E-11	FARSB
H2BC17	5919.95	-1.11806	0.154595	-7.23219	4.75E-13	3.11E-11	H2BC17
BHLHE40	17288	1.158054	0.160138	7.231606	4.77E-13	3.12E-11	BHLHE40
LOC10272	128.652	4.025312	0.556897	7.22811	4.90E-13	3.19E-11	LOC10272
H2AC12	8442.699	-1.11469	0.154275	-7.22533	5.00E-13	3.24E-11	H2AC12
MCM2	4767.537	-1.10044	0.152402	-7.22063	5.17E-13	3.34E-11	MCM2
AIFM1	3893.076	-1.0963	0.151896	-7.21738	5.30E-13	3.41E-11	AIFM1
TTK	3600.477	-1.2107	0.167918	-7.21005	5.59E-13	3.59E-11	TTK
CDCA5	2654.498	-1.22413	0.170129	-7.1953	6.23E-13	3.98E-11	CDCA5
EDN2	179.8875	2.996968	0.417731	7.174403	7.26E-13	4.63E-11	EDN2
MCM7	15062.97	-1.00887	0.140731	-7.1688	7.57E-13	4.80E-11	MCM7

E2F8	574.895	-1.55287	0.216896	-7.15951	8.10E-13	5.12E-11	E2F8
CARHSP1	6170.449	1.187592	0.166134	7.148376	8.78E-13	5.53E-11	CARHSP1
H3C11	1852.643	-1.42148	0.198931	-7.14558	8.96E-13	5.63E-11	H3C11
VIM	57978.19	1.169403	0.163871	7.136134	9.60E-13	6.01E-11	VIM
HSP90AA1	97387.17	-1.02756	0.144262	-7.12287	1.06E-12	6.59E-11	HSP90AA1
ASB4	654.1903	-1.57934	0.221797	-7.12065	1.07E-12	6.68E-11	ASB4
BUB1B	4072.756	-1.07472	0.151	-7.11732	1.10E-12	6.82E-11	BUB1B
MCCC1	3547.913	1.198772	0.168537	7.112793	1.14E-12	7.02E-11	MCCC1
BRCA1	7162.482	-1.1612	0.163308	-7.11048	1.16E-12	7.12E-11	BRCA1
FANK1	659.4938	1.585632	0.223627	7.090526	1.34E-12	8.20E-11	FANK1
LINC01671	71.59721	4.135347	0.584224	7.078357	1.46E-12	8.92E-11	LINC01671
EIF5B	3695.819	-1.24672	0.176761	-7.05318	1.75E-12	1.07E-10	EIF5B
SKA3	1217.221	-1.48496	0.210768	-7.04546	1.85E-12	1.12E-10	SKA3
TYMS	856.0327	-1.4843	0.210811	-7.04091	1.91E-12	1.16E-10	TYMS
NCF2	216.7255	2.182335	0.310021	7.039322	1.93E-12	1.17E-10	NCF2
PPP2R5B	1173.368	1.40713	0.199925	7.038301	1.95E-12	1.17E-10	PPP2R5B
PEA15	3418.396	1.095225	0.155626	7.03754	1.96E-12	1.17E-10	PEA15
SLC38A2	76463.16	1.128265	0.160465	7.031202	2.05E-12	1.22E-10	SLC38A2
LOC10537	282.2371	2.045103	0.290949	7.029078	2.08E-12	1.24E-10	LOC10537
YARS1	5466.924	-1.0631	0.151463	-7.01885	2.24E-12	1.33E-10	YARS1
FUS	3542.661	-1.22343	0.174707	-7.00276	2.51E-12	1.48E-10	FUS
HOXD10	274.261	2.200749	0.314831	6.990264	2.74E-12	1.62E-10	HOXD10
H3C12	4194.847	-1.16027	0.166042	-6.98778	2.79E-12	1.64E-10	H3C12
HPCAL1	2745.573	1.092189	0.156315	6.987094	2.81E-12	1.64E-10	HPCAL1
UCN2	146.5825	2.814688	0.402967	6.984918	2.85E-12	1.66E-10	UCN2
SPAG5	8797.66	-1.05044	0.150454	-6.98176	2.92E-12	1.70E-10	SPAG5
KRT81	62755.35	-1.02038	0.146166	-6.98096	2.93E-12	1.70E-10	KRT81
NR4A1	1212.013	-1.69735	0.243296	-6.9765	3.03E-12	1.75E-10	NR4A1
H2BC3	2791.852	-1.0927	0.15674	-6.9714	3.14E-12	1.81E-10	H2BC3
MCM6	4183.803	-1.1844	0.16991	-6.97076	3.15E-12	1.81E-10	MCM6
CCNB1	9561.733	-1.05089	0.150822	-6.96774	3.22E-12	1.85E-10	CCNB1
SFXN3	3635.693	1.176475	0.168932	6.964205	3.30E-12	1.89E-10	SFXN3
KIF11	9131.146	-1.21077	0.173891	-6.96283	3.33E-12	1.90E-10	KIF11
HEG1	2328.938	1.360139	0.195632	6.952523	3.59E-12	2.04E-10	HEG1
CDC6	5035.967	-1.10497	0.158982	-6.95027	3.65E-12	2.06E-10	CDC6
NOC2L	4193.644	-1.05176	0.151608	-6.93736	3.99E-12	2.25E-10	NOC2L
LAMC2	2677.339	1.094246	0.157803	6.934259	4.08E-12	2.30E-10	LAMC2
GINS2	1986.254	-1.19089	0.17181	-6.93145	4.17E-12	2.34E-10	GINS2
C15orf48	312.3234	2.676627	0.386241	6.929946	4.21E-12	2.35E-10	C15orf48
FAS	1166.302	-1.35215	0.195128	-6.92956	4.22E-12	2.35E-10	FAS
DEPDC1	3292.878	-1.31499	0.189812	-6.92784	4.27E-12	2.38E-10	DEPDC1
NOLC1	9913.846	-1.05418	0.152189	-6.92674	4.31E-12	2.39E-10	NOLC1
GPR153	336.0654	1.736807	0.250884	6.922751	4.43E-12	2.44E-10	GPR153
SLAMF7	115.8557	3.138205	0.453329	6.922581	4.43E-12	2.44E-10	SLAMF7
GSN	10642.22	1.201192	0.173533	6.921968	4.45E-12	2.45E-10	GSN
MT2A	2958.889	1.365973	0.197429	6.918825	4.55E-12	2.49E-10	MT2A
DTL	3993.548	-1.19019	0.172271	-6.90883	4.89E-12	2.67E-10	DTL
NOP58	4558.364	-1.02401	0.148253	-6.90719	4.94E-12	2.69E-10	NOP58
PFKL	7269.657	1.046864	0.151855	6.893846	5.43E-12	2.95E-10	PFKL
ATP5MC1	4480.168	-1.05238	0.152766	-6.88888	5.62E-12	3.04E-10	ATP5MC1
CPNE4	659.5928	-1.59636	0.231734	-6.88877	5.63E-12	3.04E-10	CPNE4
SNRPF	2206.782	-1.10165	0.160301	-6.8724	6.31E-12	3.40E-10	SNRPF
DNAJB11	4449.59	-1.03515	0.150651	-6.87118	6.37E-12	3.41E-10	DNAJB11
ABCA3	5093.788	1.111049	0.161707	6.870741	6.39E-12	3.42E-10	ABCA3
NPM3	1259.95	-1.26617	0.184445	-6.86474	6.66E-12	3.55E-10	NPM3
TRIP13	2822.626	-1.09389	0.159457	-6.86012	6.88E-12	3.66E-10	TRIP13
H2BC18	5474.212	-1.21591	0.177303	-6.8578	6.99E-12	3.71E-10	H2BC18
TBX3	3194.177	-1.12876	0.164713	-6.85291	7.24E-12	3.82E-10	TBX3

HOXD11	193.9856	2.157208	0.314829	6.852007	7.28E-12	3.84E-10	HOXD11
PDE9A	663.3778	1.463969	0.213671	6.851516	7.31E-12	3.84E-10	PDE9A
CXCL2	530.7882	-1.621	0.236715	-6.8479	7.49E-12	3.93E-10	CXCL2
SYTL2	3284.891	1.525504	0.222791	6.84725	7.53E-12	3.93E-10	SYTL2
GCLM	9068.832	-1.15788	0.169148	-6.84541	7.63E-12	3.97E-10	GCLM
LNCAROD	715.0574	-1.65672	0.242195	-6.84045	7.89E-12	4.10E-10	LNCAROD
ANP32B	3603.105	-1.31891	0.192948	-6.83556	8.17E-12	4.23E-10	ANP32B
NYAP1	442.5304	2.08902	0.305662	6.834422	8.23E-12	4.25E-10	NYAP1
BRIX1	3064.934	-1.08583	0.159003	-6.829	8.55E-12	4.39E-10	BRIX1
CENPE	4565.084	-1.20843	0.176965	-6.82866	8.57E-12	4.39E-10	CENPE
H2BC15	2953.901	-1.34585	0.197065	-6.82945	8.52E-12	4.39E-10	H2BC15
GFPT2	2415.147	1.099834	0.161134	6.825569	8.76E-12	4.47E-10	GFPT2
EFNA1	1704.267	1.302362	0.190903	6.82212	8.97E-12	4.57E-10	EFNA1
TMPO	16662.57	-1.03527	0.151883	-6.8162	9.35E-12	4.75E-10	TMPO
DIO2	1326.943	-1.45039	0.21281	-6.8154	9.40E-12	4.76E-10	DIO2
HAUS7	1110.36	-1.25384	0.184057	-6.81226	9.61E-12	4.85E-10	HAUS7
MYL6	53304.82	1.025953	0.150889	6.799398	1.05E-11	5.29E-10	MYL6
KCNMB4	210.2494	2.335669	0.34375	6.794667	1.09E-11	5.46E-10	KCNMB4
CDCA7	2357.066	-1.41751	0.208741	-6.79077	1.12E-11	5.59E-10	CDCA7
MCM4	12621.68	-1.01628	0.149894	-6.77998	1.20E-11	6.01E-10	MCM4
SH3PXD2A	1878.924	-1.23669	0.182528	-6.77532	1.24E-11	6.19E-10	SH3PXD2A
CPS1	8670.934	-1.50604	0.22237	-6.7727	1.26E-11	6.28E-10	CPS1
DAPK3	2137.649	1.185729	0.175213	6.767342	1.31E-11	6.50E-10	DAPK3
ENO1	197530.7	1.063573	0.157309	6.761039	1.37E-11	6.76E-10	ENO1
JUNB	5230.607	1.024434	0.151732	6.751608	1.46E-11	7.19E-10	JUNB
SLCO1C1	704.8179	1.840952	0.272835	6.74748	1.50E-11	7.38E-10	SLCO1C1
LRP1	8819.885	1.397007	0.207297	6.739156	1.59E-11	7.79E-10	LRP1
TOR3A	2286.217	-1.07421	0.159457	-6.7367	1.62E-11	7.90E-10	TOR3A
UPK1A-AS	60.91475	4.196214	0.623268	6.732599	1.67E-11	8.09E-10	UPK1A-AS
WDR76	2030.305	-1.10232	0.163798	-6.7298	1.70E-11	8.22E-10	WDR76
SH3D21	219.8602	2.084213	0.309823	6.7271	1.73E-11	8.35E-10	SH3D21
AFAP1L2	3666.754	1.048647	0.156012	6.721571	1.80E-11	8.65E-10	AFAP1L2
HTRA1	4898.428	1.125109	0.167418	6.720344	1.81E-11	8.71E-10	HTRA1
MYOF	51947.33	1.046195	0.155724	6.718276	1.84E-11	8.81E-10	MYOF
C11orf86	2551.861	3.986034	0.593567	6.715391	1.88E-11	8.96E-10	C11orf86
GABRE	5064.186	1.22105	0.181939	6.711311	1.93E-11	9.19E-10	GABRE
SHCBP1	3424.586	-1.21242	0.18067	-6.71068	1.94E-11	9.19E-10	SHCBP1
XBP1	11805.75	-1.27225	0.189588	-6.71059	1.94E-11	9.19E-10	XBP1
NCAPH	2479.998	-1.06042	0.158155	-6.70494	2.01E-11	9.52E-10	NCAPH
UNC5D	593.6268	-1.44051	0.214922	-6.70245	2.05E-11	9.66E-10	UNC5D
HMGA2	1745.706	-1.30069	0.194164	-6.69895	2.10E-11	9.87E-10	HMGA2
DLGAP5	5863.958	-1.01956	0.152351	-6.69214	2.20E-11	1.03E-09	DLGAP5
XRCC2	1732.482	-1.29998	0.194285	-6.6911	2.21E-11	1.04E-09	XRCC2
HNRNPA3	3642.763	-1.2392	0.185249	-6.6894	2.24E-11	1.05E-09	HNRNPA3
CLTRN	168.8351	3.030592	0.453129	6.688138	2.26E-11	1.05E-09	CLTRN
LINC02582	3742.479	1.052208	0.157317	6.688458	2.26E-11	1.05E-09	LINC02582
CDC45	1343.814	-1.14773	0.171905	-6.67654	2.45E-11	1.13E-09	CDC45
ALDOA	631.6394	1.788796	0.268063	6.673041	2.51E-11	1.16E-09	ALDOA
DHFR	2163.345	-1.11925	0.167861	-6.66774	2.60E-11	1.20E-09	DHFR
H2BC13	6238.604	-1.15168	0.173057	-6.6549	2.84E-11	1.30E-09	H2BC13
DSCAML1	296.9512	1.850111	0.278162	6.651209	2.91E-11	1.33E-09	DSCAML1
KIF18A	4759.183	-1.13654	0.171286	-6.63535	3.24E-11	1.48E-09	KIF18A
PRMT3	2280.862	-1.07128	0.161723	-6.62417	3.49E-11	1.59E-09	PRMT3
TARDBP	3836.948	-1.08749	0.164792	-6.59917	4.13E-11	1.88E-09	TARDBP
TUBA1A	14017.96	1.066382	0.161596	6.59906	4.14E-11	1.88E-09	TUBA1A
DOCK2	236.3237	2.218098	0.336351	6.594602	4.26E-11	1.93E-09	DOCK2
PERP	28462.48	1.188769	0.180329	6.592221	4.33E-11	1.96E-09	PERP
H4C9	1817.044	-1.21262	0.184327	-6.57862	4.75E-11	2.14E-09	H4C9

H2AX	2677.425	-1.12223	0.170671	-6.57539	4.85E-11	2.18E-09	H2AX
ANP32E	7478.647	-1.19578	0.182017	-6.56961	5.04E-11	2.25E-09	ANP32E
POLE2	852.1354	-1.31388	0.199983	-6.56994	5.03E-11	2.25E-09	POLE2
LINC0096:	1606.491	1.160766	0.176739	6.567685	5.11E-11	2.28E-09	LINC0096:
PAG1	159.7155	2.833962	0.431703	6.564618	5.22E-11	2.32E-09	PAG1
MTHFD2	6124.623	-1.1463	0.174647	-6.56351	5.26E-11	2.33E-09	MTHFD2
UBE2C	3025.446	-1.05732	0.161387	-6.55143	5.70E-11	2.52E-09	UBE2C
LTV1	2399.085	-1.059	0.161658	-6.55089	5.72E-11	2.52E-09	LTV1
FANCD2	3799.183	-1.06796	0.1631	-6.54789	5.84E-11	2.57E-09	FANCD2
PLA2R1	2463.938	1.427991	0.218322	6.540758	6.12E-11	2.69E-09	PLA2R1
MND1	498.042	-1.50734	0.230491	-6.53969	6.16E-11	2.70E-09	MND1
ANKRD13/	3212.638	1.062059	0.162438	6.538257	6.22E-11	2.71E-09	ANKRD13/
NEK2	3216.453	-1.06786	0.163493	-6.53155	6.51E-11	2.83E-09	NEK2
CAVIN2-A	264.2133	1.809642	0.277241	6.527328	6.70E-11	2.90E-09	CAVIN2-A
KIF22	4081.122	-1.01739	0.155874	-6.52702	6.71E-11	2.90E-09	KIF22
TNIP1	8210.538	1.137384	0.174331	6.524272	6.83E-11	2.95E-09	TNIP1
NEK6	3379.949	1.205111	0.184936	6.516366	7.20E-11	3.10E-09	NEK6
LINC0158:	77.72412	4.813324	0.738784	6.515199	7.26E-11	3.12E-09	LINC0158:
GLP2R	4302.783	1.276372	0.196027	6.511204	7.46E-11	3.19E-09	GLP2R
HSPA4L	3443.048	-1.05856	0.163144	-6.48851	8.67E-11	3.70E-09	HSPA4L
SNX33	5507.013	1.041418	0.160534	6.487228	8.74E-11	3.72E-09	SNX33
MDGA1	532.3775	1.475	0.227586	6.481071	9.11E-11	3.87E-09	MDGA1
SHC2	351.9867	2.080036	0.321002	6.479832	9.18E-11	3.89E-09	SHC2
ABCG2	6089.403	-1.15757	0.178828	-6.47309	9.60E-11	4.06E-09	ABCG2
FAT4	1582.7	1.428719	0.220875	6.468458	9.90E-11	4.17E-09	FAT4
KIF18B	2623.817	-1.02775	0.158931	-6.46663	1.00E-10	4.22E-09	KIF18B
SGO1	1098.548	-1.36486	0.211088	-6.46585	1.01E-10	4.23E-09	SGO1
PTPRE	508.607	1.514809	0.234402	6.462452	1.03E-10	4.31E-09	PTPRE
SNORC	1029.816	1.39744	0.21628	6.461267	1.04E-10	4.34E-09	SNORC
SHB	4418.663	1.344747	0.20818	6.459543	1.05E-10	4.38E-09	SHB
BUB1	3934.691	-1.05089	0.163006	-6.44696	1.14E-10	4.74E-09	BUB1
FLJ16779	154.6152	2.201399	0.341467	6.446894	1.14E-10	4.74E-09	FLJ16779
MTHFD1L	4199.594	1.028798	0.159764	6.439467	1.20E-10	4.96E-09	MTHFD1L
KIF4A	3843.759	-1.04444	0.162468	-6.42863	1.29E-10	5.32E-09	KIF4A
CTSL	20375.93	-1.03847	0.161552	-6.42807	1.29E-10	5.33E-09	CTSL
RHBDF1	1965.33	1.236841	0.192455	6.426638	1.30E-10	5.34E-09	RHBDF1
TSC22D2	2199.8	1.059849	0.165039	6.42181	1.35E-10	5.49E-09	TSC22D2
MPI	5345.585	1.011111	0.157558	6.417379	1.39E-10	5.63E-09	MPI
DSC2	1749.848	1.224997	0.190934	6.415822	1.40E-10	5.67E-09	DSC2
SNAP25	2087.673	-1.3511	0.210596	-6.4156	1.40E-10	5.67E-09	SNAP25
SRRM3	661.0418	1.366412	0.213111	6.411731	1.44E-10	5.80E-09	SRRM3
GDPD5	1111.177	1.213942	0.18936	6.410749	1.45E-10	5.82E-09	GDPD5
CEBPD	4954.625	1.198485	0.187071	6.406596	1.49E-10	5.97E-09	CEBPD
GACAT2	402.5586	1.555741	0.242991	6.402475	1.53E-10	6.12E-09	GACAT2
R3HDM1	2073.067	-1.03745	0.162044	-6.40228	1.53E-10	6.12E-09	R3HDM1
H3C3	4533.144	-1.0349	0.161681	-6.40088	1.54E-10	6.16E-09	H3C3
ASRGL1	827.3881	-1.2516	0.195568	-6.39982	1.56E-10	6.19E-09	ASRGL1
CAMK2N1	4330.55	1.089555	0.170423	6.393233	1.62E-10	6.43E-09	CAMK2N1
IL11	848.617	1.476207	0.231653	6.372495	1.86E-10	7.35E-09	IL11
INCENP	4877.456	-1.01296	0.159058	-6.36846	1.91E-10	7.53E-09	INCENP
SRM	1149.395	-1.12943	0.177402	-6.3665	1.93E-10	7.61E-09	SRM
AMPD3	579.8637	1.41051	0.221577	6.365767	1.94E-10	7.63E-09	AMPD3
OBSCN	3186.81	1.081997	0.169985	6.365248	1.95E-10	7.64E-09	OBSCN
ENPP1	1078	-1.13256	0.178349	-6.35024	2.15E-10	8.39E-09	ENPP1
CENPK	1934.889	-1.19608	0.188383	-6.34922	2.16E-10	8.43E-09	CENPK
HMGN2	2952.663	-1.10593	0.17428	-6.34572	2.21E-10	8.59E-09	HMGN2
SUSD2	1116.669	-1.27372	0.201108	-6.33349	2.40E-10	9.27E-09	SUSD2
S100A3	1051.571	1.657951	0.262277	6.321374	2.59E-10	9.99E-09	S100A3

STS	1831.538	1.311495	0.207587	6.317809	2.65E-10	1.02E-08	STS
FAM81A	1168.3	-1.37815	0.218225	-6.3153	2.70E-10	1.03E-08	FAM81A
MYZAP	169.1954	2.630216	0.416486	6.315256	2.70E-10	1.03E-08	MYZAP
ANXA3	8590.21	1.050759	0.166455	6.312559	2.74E-10	1.05E-08	ANXA3
ORC1	1689.237	-1.08744	0.172457	-6.30556	2.87E-10	1.09E-08	ORC1
AMOTL2	9230.565	1.150717	0.182536	6.304069	2.90E-10	1.10E-08	AMOTL2
SCARA5	1752.352	-1.31904	0.209297	-6.30223	2.93E-10	1.11E-08	SCARA5
PDK4	6752.453	-3.07978	0.489373	-6.29332	3.11E-10	1.17E-08	PDK4
PPP1R13L	576.3037	1.304592	0.207606	6.283981	3.30E-10	1.24E-08	PPP1R13L
CREBRF	1874.26	1.143127	0.182081	6.278108	3.43E-10	1.29E-08	CREBRF
SYNC	553.0388	1.37113	0.218543	6.273954	3.52E-10	1.32E-08	SYNC
DSCC1	844.7914	-1.37665	0.219441	-6.27343	3.53E-10	1.32E-08	DSCC1
ADGRF4	689.0402	1.372685	0.218858	6.272027	3.56E-10	1.33E-08	ADGRF4
RAB20	348.0214	1.76373	0.281453	6.26652	3.69E-10	1.37E-08	RAB20
CDA	662.8974	1.639013	0.261674	6.263572	3.76E-10	1.40E-08	CDA
HYKK	624.4903	-1.29649	0.207115	-6.25978	3.86E-10	1.43E-08	HYKK
SCTR	190.6484	2.316473	0.370929	6.245051	4.24E-10	1.56E-08	SCTR
H2AC14	2235.681	-1.33059	0.213164	-6.24209	4.32E-10	1.58E-08	H2AC14
DCTPP1	1776.429	-1.03	0.165081	-6.23939	4.39E-10	1.61E-08	DCTPP1
CHRNA5	1656.086	-1.08116	0.173329	-6.23761	4.44E-10	1.62E-08	CHRNA5
RBFOX3	847.3221	-1.38856	0.222702	-6.23506	4.52E-10	1.65E-08	RBFOX3
IGFL2-AS1	3915.009	1.426394	0.228806	6.234083	4.54E-10	1.65E-08	IGFL2-AS1
ZNF772	664.1858	1.257293	0.2017	6.233489	4.56E-10	1.66E-08	ZNF772
MAD2L1	2991.904	-1.14608	0.184239	-6.22059	4.95E-10	1.79E-08	MAD2L1
CENPA	1513.293	-1.07713	0.17321	-6.21863	5.02E-10	1.81E-08	CENPA
PPIH	1297.909	-1.07892	0.173809	-6.20748	5.38E-10	1.93E-08	PPIH
HMBS	945.6806	-1.19205	0.192101	-6.20531	5.46E-10	1.96E-08	HMBS
DPYSL2	41693.77	1.114709	0.179807	6.199469	5.67E-10	2.03E-08	DPYSL2
CSRNP1	1799.37	1.017405	0.164468	6.186044	6.17E-10	2.20E-08	CSRNP1
CCND3	6178.738	-1.11396	0.181127	-6.15014	7.74E-10	2.75E-08	CCND3
ITGB4	14667.42	1.202233	0.19551	6.149215	7.79E-10	2.76E-08	ITGB4
H2AC15	1510.471	-1.16942	0.190455	-6.1401	8.25E-10	2.91E-08	H2AC15
SORD	1082.753	-1.12665	0.183582	-6.13706	8.41E-10	2.97E-08	SORD
PRELID2	1735.581	1.00513	0.163806	6.136097	8.46E-10	2.97E-08	PRELID2
PRIM1	1839.002	-1.07787	0.175693	-6.13499	8.52E-10	2.99E-08	PRIM1
LRFN4	3028.569	1.143027	0.186329	6.134462	8.54E-10	2.99E-08	LRFN4
ADGRG2	1056.358	1.108089	0.180735	6.131032	8.73E-10	3.04E-08	ADGRG2
GAR1	323.9557	-1.65253	0.269619	-6.12915	8.83E-10	3.07E-08	GAR1
BFSP1	1300.547	1.508191	0.246466	6.119265	9.40E-10	3.26E-08	BFSP1
NUF2	2038.865	-1.0436	0.17059	-6.11755	9.50E-10	3.29E-08	NUF2
C11orf80	1213.269	1.078804	0.176359	6.117107	9.53E-10	3.29E-08	C11orf80
CSDC2	191.0103	1.986394	0.324865	6.114514	9.69E-10	3.33E-08	CSDC2
FAM72B	193.7979	-1.98542	0.324761	-6.11347	9.75E-10	3.35E-08	FAM72B
NEIL3	1736.591	-1.1524	0.188802	-6.10374	1.04E-09	3.54E-08	NEIL3
UNC13D	995.816	1.140801	0.187224	6.093231	1.11E-09	3.75E-08	UNC13D
H2BC10	3416.201	-1.07792	0.177074	-6.08737	1.15E-09	3.87E-08	H2BC10
SMC4	13330.52	-1.04682	0.17209	-6.08299	1.18E-09	3.96E-08	SMC4
PROC	266.6436	1.872434	0.308015	6.079027	1.21E-09	4.05E-08	PROC
WDR62	2219.402	-1.16121	0.191049	-6.07806	1.22E-09	4.06E-08	WDR62
SERPINA3	277.1552	-2.01208	0.331266	-6.0739	1.25E-09	4.16E-08	SERPINA3
SLC39A13	2430.281	1.038265	0.171318	6.06044	1.36E-09	4.50E-08	SLC39A13
TOP2A	55196.86	-1.02146	0.168599	-6.05853	1.37E-09	4.55E-08	TOP2A
ABCA5	1756.649	1.020579	0.168591	6.053574	1.42E-09	4.67E-08	ABCA5
TNFAIP8	4008.884	1.2284	0.203004	6.051124	1.44E-09	4.73E-08	TNFAIP8
RAD51C	2156.03	-1.03916	0.171807	-6.04845	1.46E-09	4.80E-08	RAD51C
GMNN	1515.915	-1.05591	0.174618	-6.04699	1.48E-09	4.83E-08	GMNN
HMMR	4275.544	-1.15817	0.191905	-6.03508	1.59E-09	5.19E-08	HMMR
NUDT1	1182.339	-1.06833	0.177279	-6.02623	1.68E-09	5.47E-08	NUDT1

LOC10012	732.7732	1.257688	0.208743	6.025058	1.69E-09	5.50E-08	LOC10012
TACC2	6324.12	-1.00669	0.16709	-6.02483	1.69E-09	5.50E-08	TACC2
C1S	5249.214	1.408883	0.233881	6.023922	1.70E-09	5.51E-08	C1S
RRAGD	1129.683	1.114205	0.18496	6.024029	1.70E-09	5.51E-08	RRAGD
PARP2	1466.188	-1.13348	0.188189	-6.0231	1.71E-09	5.53E-08	PARP2
PGM2L1	7469.623	1.145865	0.190259	6.022642	1.72E-09	5.53E-08	PGM2L1
ADAMTS1	4138.307	1.005108	0.167075	6.015918	1.79E-09	5.76E-08	ADAMTS1(
SCARB1	9854.833	1.074295	0.178604	6.014967	1.80E-09	5.77E-08	SCARB1
LXN	4724.435	-1.17511	0.195529	-6.0099	1.86E-09	5.91E-08	LXN
RTL8B	815.4422	1.18327	0.196887	6.0099	1.86E-09	5.91E-08	RTL8B
KCNV1	172.1525	-1.96782	0.327748	-6.00407	1.92E-09	6.12E-08	KCNV1
PCLAF	3139.728	-1.06533	0.17749	-6.0022	1.95E-09	6.18E-08	PCLAF
EGFR-AS1	210.9414	1.885096	0.31428	5.998142	2.00E-09	6.32E-08	EGFR-AS1
GUCY2D	98.42048	2.887361	0.481514	5.996417	2.02E-09	6.38E-08	GUCY2D
RMI1	618.3736	-1.42998	0.238512	-5.99543	2.03E-09	6.41E-08	RMI1
ERCC6L	2334.724	-1.0008	0.166987	-5.9933	2.06E-09	6.48E-08	ERCC6L
SASS6	1350.3	-1.19651	0.199781	-5.9891	2.11E-09	6.63E-08	SASS6
C1QL1	562.2253	1.35372	0.226094	5.987419	2.13E-09	6.68E-08	C1QL1
JPH2	552.6923	1.315162	0.219932	5.979853	2.23E-09	6.95E-08	JPH2
CCNA2	3778.92	-1.0089	0.168972	-5.9708	2.36E-09	7.33E-08	CCNA2
TIMM21	1272.807	-1.04135	0.174416	-5.97048	2.37E-09	7.33E-08	TIMM21
CCNG2	1620.207	1.113069	0.186564	5.966142	2.43E-09	7.50E-08	CCNG2
LANCL1	5223.934	-1.13235	0.189966	-5.9608	2.51E-09	7.74E-08	LANCL1
PLXNA3	1016.095	1.312136	0.220177	5.959471	2.53E-09	7.76E-08	PLXNA3
PAQR6	987.1089	1.12517	0.188889	5.956768	2.57E-09	7.86E-08	PAQR6
KIFC2	2467.558	1.030576	0.17306	5.95502	2.60E-09	7.94E-08	KIFC2
TBC1D30	212.5628	-1.92058	0.322737	-5.95092	2.67E-09	8.12E-08	TBC1D30
FBN1	3582.576	1.106235	0.185917	5.950162	2.68E-09	8.15E-08	FBN1
CDC25A	830.9759	-1.14812	0.193136	-5.94463	2.77E-09	8.39E-08	CDC25A
H2AC4	5192.391	-1.00929	0.169785	-5.94451	2.77E-09	8.39E-08	H2AC4
CHAMP1	1224.55	-1.17944	0.198557	-5.94008	2.85E-09	8.61E-08	CHAMP1
CDK1	6688.572	-1.04612	0.17626	-5.93508	2.94E-09	8.86E-08	CDK1
TIMD4	597.9508	1.375181	0.231947	5.928869	3.05E-09	9.19E-08	TIMD4
ISOC1	1130.998	-1.05938	0.179412	-5.90473	3.53E-09	1.06E-07	ISOC1
L2HGDH	1134.277	-1.04991	0.177947	-5.90011	3.63E-09	1.08E-07	L2HGDH
PTPRN	46.40344	4.18583	0.709491	5.899766	3.64E-09	1.08E-07	PTPRN
HECA	987.6407	1.09074	0.184983	5.89643	3.71E-09	1.10E-07	HECA
TMEM74B	686.7871	1.337414	0.226873	5.895001	3.75E-09	1.11E-07	TMEM74B
NDC80	993.0585	-1.19095	0.202043	-5.89454	3.76E-09	1.11E-07	NDC80
NAP1L2	363.1697	-1.50974	0.256159	-5.89377	3.77E-09	1.11E-07	NAP1L2
MTFR2	529.4314	-1.39277	0.236411	-5.89133	3.83E-09	1.13E-07	MTFR2
EME1	1292.343	-1.25185	0.212733	-5.88461	3.99E-09	1.17E-07	EME1
ATAD5	2898.592	-1.01676	0.172931	-5.87954	4.11E-09	1.21E-07	ATAD5
PARP3	1063.148	1.119538	0.19045	5.878389	4.14E-09	1.21E-07	PARP3
CMBL	791.7022	-1.18973	0.202478	-5.87586	4.21E-09	1.23E-07	CMBL
SNTA1	1050.395	1.091987	0.185889	5.874409	4.24E-09	1.24E-07	SNTA1
RRP9	1035.623	-1.06985	0.182368	-5.86643	4.45E-09	1.29E-07	RRP9
BRCA2	2831.608	-1.04144	0.177572	-5.86492	4.49E-09	1.30E-07	BRCA2
LOC10272	320.1943	1.589408	0.271199	5.860669	4.61E-09	1.33E-07	LOC10272
ALDOC	2751.473	4.02415	0.687801	5.850744	4.89E-09	1.41E-07	ALDOC
LOC10012	3253.16	1.295828	0.221531	5.84941	4.93E-09	1.42E-07	LOC10012
RERG	529.9734	1.841726	0.315174	5.843511	5.11E-09	1.46E-07	RERG
MXRA7	3793.248	1.011559	0.173246	5.838873	5.26E-09	1.50E-07	MXRA7
ARHGAP4	257.5898	1.64944	0.282555	5.837598	5.30E-09	1.51E-07	ARHGAP4
HELLS	8609.316	-1.04042	0.17823	-5.83752	5.30E-09	1.51E-07	HELLS
PCDHAC2	1031.78	-1.41162	0.241853	-5.83669	5.32E-09	1.52E-07	PCDHAC2
RNR2	10491200	-1.10368	0.189322	-5.82967	5.55E-09	1.58E-07	RNR2
GAB2	1786.628	1.116445	0.191787	5.821269	5.84E-09	1.65E-07	GAB2

BOK	873.3419	1.232034	0.211875	5.814901	6.07E-09	1.71E-07	BOK
LOC72965	252.8356	-1.72263	0.296502	-5.80982	6.25E-09	1.76E-07	LOC72965
DENND2A	223.6163	2.030397	0.349929	5.802317	6.54E-09	1.82E-07	DENND2A
RAD51AP1	1579.233	-1.09934	0.189458	-5.80253	6.53E-09	1.82E-07	RAD51AP1
RAB27B	9991.486	-1.15473	0.199115	-5.7993	6.66E-09	1.85E-07	RAB27B
CYP4F11	778.2566	-1.17018	0.201959	-5.79414	6.87E-09	1.90E-07	CYP4F11
TRNV	165.0426	-1.97116	0.340362	-5.79136	6.98E-09	1.93E-07	TRNV
CEP83	1392.069	-1.04285	0.18011	-5.79008	7.04E-09	1.94E-07	CEP83
IFI30	562.7508	-1.25031	0.216313	-5.7801	7.47E-09	2.05E-07	IFI30
ETV1	1001.811	-1.19237	0.206316	-5.77935	7.50E-09	2.06E-07	ETV1
PRRG4	751.9975	1.65027	0.285787	5.774468	7.72E-09	2.11E-07	PRRG4
PARM1	1097.77	-1.3317	0.230804	-5.76982	7.94E-09	2.16E-07	PARM1
HOMER3	1702.745	1.311367	0.227385	5.767159	8.06E-09	2.19E-07	HOMER3
RHOV	764.1679	-1.50289	0.260809	-5.76243	8.29E-09	2.24E-07	RHOV
MAF	109.0948	3.008059	0.522295	5.759316	8.45E-09	2.28E-07	MAF
ARRDC3-A	432.9701	1.564735	0.272058	5.751481	8.85E-09	2.37E-07	ARRDC3-A
RFC4	2082.753	-1.014	0.176341	-5.75019	8.91E-09	2.39E-07	RFC4
SDSL	473.8499	-1.36777	0.237879	-5.74985	8.93E-09	2.39E-07	SDSL
VAV1	711.283	1.182447	0.205728	5.747612	9.05E-09	2.42E-07	VAV1
FST	728.5467	-1.68191	0.292669	-5.7468	9.09E-09	2.42E-07	FST
HIF1A-AS3	196.4705	1.844615	0.321459	5.738255	9.57E-09	2.54E-07	HIF1A-AS3
SAP30-DT	112.2366	2.465913	0.429829	5.736964	9.64E-09	2.56E-07	SAP30-DT
TMEM45B	187.7916	2.108364	0.367614	5.735266	9.74E-09	2.58E-07	TMEM45B
PSAT1	8811.81	-1.28246	0.223683	-5.73339	9.84E-09	2.60E-07	PSAT1
FAM98B	749.7963	-1.28911	0.224903	-5.73184	9.93E-09	2.62E-07	FAM98B
H4C13	1735.362	-1.14304	0.199631	-5.72575	1.03E-08	2.71E-07	H4C13
NUDT18	1468.29	1.061667	0.185435	5.725274	1.03E-08	2.71E-07	NUDT18
PXDC1	1169.869	1.164755	0.203462	5.724685	1.04E-08	2.71E-07	PXDC1
TNFAIP6	151.2514	2.044835	0.357183	5.724896	1.03E-08	2.71E-07	TNFAIP6
H3C10	6447.47	-1.0429	0.182371	-5.71858	1.07E-08	2.80E-07	H3C10
RAD54L	660.6173	-1.16246	0.203275	-5.71866	1.07E-08	2.80E-07	RAD54L
RNF24	907.3849	1.179389	0.206349	5.715493	1.09E-08	2.84E-07	RNF24
SFRP1	5542.72	1.002215	0.175346	5.715631	1.09E-08	2.84E-07	SFRP1
CAVIN3	385.8213	1.512123	0.264742	5.71169	1.12E-08	2.90E-07	CAVIN3
ATP6V0A4	174.3398	2.681835	0.469658	5.710187	1.13E-08	2.92E-07	ATP6V0A4
CRISPLD2	165.6892	2.057911	0.360438	5.709478	1.13E-08	2.93E-07	CRISPLD2
COQ3	451.8755	-1.33045	0.233347	-5.7016	1.19E-08	3.06E-07	COQ3
ALPK3	245.2408	1.684909	0.295916	5.693868	1.24E-08	3.18E-07	ALPK3
TPM1-AS	1248.328	1.260218	0.221393	5.692212	1.25E-08	3.20E-07	TPM1-AS
PLCXD3	3083.073	1.124967	0.197665	5.691271	1.26E-08	3.21E-07	PLCXD3
CTU2	719.5571	-1.14399	0.201743	-5.67055	1.42E-08	3.61E-07	CTU2
ZFP36L2	1752.271	-1.15204	0.203189	-5.66981	1.43E-08	3.62E-07	ZFP36L2
MARS1	10446.72	-1.00644	0.177716	-5.6632	1.49E-08	3.75E-07	MARS1
ANXA8	274.6691	1.631831	0.288356	5.659091	1.52E-08	3.83E-07	ANXA8
HSD3B7	2745.185	1.249149	0.220745	5.658782	1.52E-08	3.83E-07	HSD3B7
ANXA8L1	642.5153	1.360933	0.240553	5.657528	1.54E-08	3.85E-07	ANXA8L1
ELF3-AS1	714.0608	1.152534	0.203886	5.652823	1.58E-08	3.94E-07	ELF3-AS1
C4orf47	104.138	2.340829	0.414146	5.652176	1.58E-08	3.95E-07	C4orf47
SEC31B	1138.699	1.006394	0.178184	5.648061	1.62E-08	4.04E-07	SEC31B
ITPR1	2755.691	1.003074	0.17768	5.645395	1.65E-08	4.08E-07	ITPR1
LOC10028	374.3783	1.389553	0.246416	5.639052	1.71E-08	4.21E-07	LOC10028
RAB6B	330.8292	1.490996	0.264401	5.63915	1.71E-08	4.21E-07	RAB6B
SLC45A4	3063.152	-1.09742	0.194752	-5.63495	1.75E-08	4.31E-07	SLC45A4
NRP2	22294.8	1.077542	0.191837	5.616967	1.94E-08	4.75E-07	NRP2
DNA2	2963.044	-1.03367	0.184641	-5.59829	2.16E-08	5.24E-07	DNA2
MAP1LC3A	448.3348	1.30872	0.233811	5.59735	2.18E-08	5.25E-07	MAP1LC3A
LINC-PINT	3019.47	1.297927	0.232417	5.584466	2.34E-08	5.62E-07	LINC-PINT
FAM72A	235.1846	-1.90003	0.340346	-5.58263	2.37E-08	5.67E-07	FAM72A

MOXD1	379.559	1.371196	0.245743	5.57979	2.41E-08	5.75E-07	MOXD1
TENT5A	3309.198	-1.33825	0.239929	-5.57768	2.44E-08	5.81E-07	TENT5A
OXTR	578.9692	1.197215	0.214675	5.576863	2.45E-08	5.83E-07	OXTR
LOC10537	585.4099	1.228469	0.220514	5.570921	2.53E-08	6.02E-07	LOC10537
CDC7	1212.954	-1.16023	0.208293	-5.57017	2.54E-08	6.04E-07	CDC7
TSPAN2	120.9428	2.172127	0.392363	5.536012	3.09E-08	7.28E-07	TSPAN2
LIN28B	1009.122	-1.21544	0.219649	-5.53355	3.14E-08	7.36E-07	LIN28B
MMS22L	1608.463	-1.01808	0.184164	-5.52812	3.24E-08	7.57E-07	MMS22L
OLFML3	1016.691	1.002864	0.181446	5.527054	3.26E-08	7.61E-07	OLFML3
PCYOX1L	616.6205	-1.4955	0.270674	-5.52511	3.29E-08	7.67E-07	PCYOX1L
ADARB1	403.5991	1.425801	0.258604	5.513458	3.52E-08	8.16E-07	ADARB1
AGMAT	764.0143	-1.17474	0.213163	-5.51102	3.57E-08	8.25E-07	AGMAT
FUNDC2	4291.743	1.01304	0.183919	5.508081	3.63E-08	8.38E-07	FUNDC2
RASGRP1	412.7765	1.305899	0.237461	5.499416	3.81E-08	8.75E-07	RASGRP1
ID4	200.6381	-1.69712	0.309161	-5.48944	4.03E-08	9.22E-07	ID4
ADPRS	1468.238	1.031951	0.188058	5.487412	4.08E-08	9.30E-07	ADPRS
SLCO1B7	1120.992	1.037705	0.189439	5.47779	4.31E-08	9.76E-07	SLCO1B7
PEAR1	169.4941	1.897027	0.346834	5.469548	4.51E-08	1.02E-06	PEAR1
HSPB8	1675.041	1.078098	0.19717	5.46785	4.56E-08	1.02E-06	HSPB8
SYDE1	1717.724	1.039574	0.190205	5.46556	4.61E-08	1.04E-06	SYDE1
MYL9	154.4942	1.855219	0.339939	5.457509	4.83E-08	1.08E-06	MYL9
LOC11226	325.7045	1.361017	0.249478	5.455451	4.88E-08	1.09E-06	LOC11226
HSPE1	1847.45	-1.00381	0.184681	-5.43536	5.47E-08	1.21E-06	HSPE1
LDLRAD4	899.8226	1.163208	0.214363	5.42636	5.75E-08	1.26E-06	LDLRAD4
RAPGEF4-	36.10763	4.862087	0.898303	5.412525	6.21E-08	1.36E-06	RAPGEF4-/
RBM20	578.1051	-1.26111	0.232983	-5.41289	6.20E-08	1.36E-06	RBM20
DIAPH3	1779.556	-1.07772	0.199241	-5.40915	6.33E-08	1.38E-06	DIAPH3
PINK1	1403.097	1.398791	0.258792	5.405074	6.48E-08	1.41E-06	PINK1
SNORA2C	354.3664	1.437093	0.26599	5.402812	6.56E-08	1.43E-06	SNORA2C
USP13	1552.281	-1.07198	0.198637	-5.39671	6.79E-08	1.47E-06	USP13
ZNF304	696.1999	1.070089	0.198321	5.395742	6.82E-08	1.48E-06	ZNF304
CEACAM6	33681.43	-1.04587	0.194026	-5.39036	7.03E-08	1.52E-06	CEACAM6
MKI67	21847.78	-1.20616	0.224021	-5.38411	7.28E-08	1.57E-06	MKI67
GPRC5B	1920.067	1.027012	0.190758	5.383841	7.29E-08	1.57E-06	GPRC5B
LINC00842	843.2395	1.070547	0.198995	5.379782	7.46E-08	1.59E-06	LINC00842
CXCL3	331.0382	-1.36122	0.253089	-5.37844	7.51E-08	1.60E-06	CXCL3
MOSPD1	2297.798	1.042581	0.194059	5.372498	7.77E-08	1.65E-06	MOSPD1
PFDN2	788.2083	-1.05025	0.195722	-5.36601	8.05E-08	1.71E-06	PFDN2
MBNL2	5656.528	1.089176	0.203003	5.365331	8.08E-08	1.72E-06	MBNL2
NIPAL4	512.5523	1.266017	0.236313	5.357367	8.44E-08	1.78E-06	NIPAL4
CA9	6798.304	4.856696	0.906993	5.354725	8.57E-08	1.80E-06	CA9
LUCAT1	1066.458	1.124346	0.210039	5.353043	8.65E-08	1.82E-06	LUCAT1
TRIB3	6809.33	1.241672	0.232084	5.350104	8.79E-08	1.84E-06	TRIB3
ANGPT1	602.4506	-1.32566	0.247852	-5.34859	8.86E-08	1.85E-06	ANGPT1
NOL12	864.5235	-1.00429	0.187767	-5.3486	8.86E-08	1.85E-06	NOL12
TMEM86A	108.3376	2.228171	0.416617	5.348246	8.88E-08	1.85E-06	TMEM86A
MANCR	151.5083	2.174795	0.406783	5.34633	8.98E-08	1.87E-06	MANCR
LOC10272	591.4047	1.119187	0.209455	5.343326	9.13E-08	1.89E-06	LOC10272
TEX15	133.6064	-2.02201	0.378817	-5.33769	9.41E-08	1.95E-06	TEX15
LOC10798	368.14	1.416674	0.265795	5.329956	9.82E-08	2.03E-06	LOC10798
NPBWR1	344.4787	1.533246	0.288944	5.306379	1.12E-07	2.30E-06	NPBWR1
LOC10050	582.1328	1.308126	0.246739	5.301652	1.15E-07	2.35E-06	LOC10050
MAP1A	442.7327	-1.4398	0.271597	-5.30122	1.15E-07	2.36E-06	MAP1A
PLEKHG6	474.805	-1.17391	0.221536	-5.29898	1.16E-07	2.38E-06	PLEKHG6
LINC0017:	170.589	-2.31604	0.438516	-5.28155	1.28E-07	2.60E-06	LINC0017:
NCALD	1802.116	-1.25479	0.237816	-5.27632	1.32E-07	2.66E-06	NCALD
NDRG4	2599.24	1.115865	0.211664	5.271876	1.35E-07	2.71E-06	NDRG4
NOX1	175.0216	-2.39611	0.454524	-5.2717	1.35E-07	2.71E-06	NOX1

NQO2	1099.443	-1.04417	0.198095	-5.27105	1.36E-07	2.71E-06	NQO2
RAB11FIP1	909.6818	1.000534	0.189859	5.26988	1.37E-07	2.73E-06	RAB11FIP1
LOX	321.4006	1.775123	0.336864	5.269558	1.37E-07	2.73E-06	LOX
DUSP5	3001.504	1.17465	0.22294	5.268899	1.37E-07	2.74E-06	DUSP5
TOP1	2637.544	-1.11392	0.21143	-5.26849	1.38E-07	2.74E-06	TOP1
SERINC2	1837.402	1.015381	0.192757	5.267683	1.38E-07	2.75E-06	SERINC2
APOBEC3E	288.4221	-1.39145	0.264369	-5.26329	1.41E-07	2.81E-06	APOBEC3E
CENPH	1201.476	-1.15448	0.21953	-5.25887	1.45E-07	2.88E-06	CENPH
MCAM	930.5403	1.114453	0.21202	5.256346	1.47E-07	2.91E-06	MCAM
RETREG1	932.6048	1.045257	0.198852	5.25645	1.47E-07	2.91E-06	RETREG1
CHAC2	549.4694	-1.46034	0.277849	-5.25585	1.47E-07	2.92E-06	CHAC2
NPAS3	36.40163	4.345207	0.826835	5.255229	1.48E-07	2.92E-06	NPAS3
ZNF185	2001.282	1.005855	0.19218	5.233931	1.66E-07	3.24E-06	ZNF185
PC	4056.709	1.120119	0.214043	5.233152	1.67E-07	3.26E-06	PC
RAP1GAP	3402.779	-1.22453	0.234023	-5.23252	1.67E-07	3.26E-06	RAP1GAP
ADAM12	1775.2	1.115552	0.213338	5.229034	1.70E-07	3.32E-06	ADAM12
H2AC16	767.6123	-1.26028	0.241329	-5.22224	1.77E-07	3.43E-06	H2AC16
NATD1	807.2502	1.180791	0.226156	5.22113	1.78E-07	3.45E-06	NATD1
EVA1B	510.2235	1.47936	0.284101	5.207163	1.92E-07	3.70E-06	EVA1B
CENPM	657.8181	-1.05195	0.20222	-5.20201	1.97E-07	3.79E-06	CENPM
DPY19L2P	407.0011	1.244682	0.239327	5.20075	1.98E-07	3.81E-06	DPY19L2P
REPS2	834.0735	1.027844	0.197662	5.20001	1.99E-07	3.82E-06	REPS2
KSR2	487.7172	1.325484	0.255485	5.188119	2.12E-07	4.05E-06	KSR2
SCN5A	116.1721	2.430731	0.468565	5.187602	2.13E-07	4.06E-06	SCN5A
C4orf46	1149.868	-1.07232	0.20672	-5.18731	2.13E-07	4.06E-06	C4orf46
ELMO1	930.8176	-1.03016	0.198761	-5.18294	2.18E-07	4.15E-06	ELMO1
TIMM8A	595.3817	-1.11233	0.214656	-5.18192	2.20E-07	4.17E-06	TIMM8A
TRNM	125.2908	-2.72161	0.525272	-5.18134	2.20E-07	4.18E-06	TRNM
DYSF	86.7613	2.408377	0.464914	5.180262	2.22E-07	4.20E-06	DYSF
KCNF1	152.0689	-1.81795	0.350943	-5.1802	2.22E-07	4.20E-06	KCNF1
LINC0260(	277.8936	1.503924	0.290376	5.179222	2.23E-07	4.21E-06	LINC0260(
LOC10798	845.7663	1.082321	0.209136	5.17521	2.28E-07	4.30E-06	LOC10798
GALNTL6	109.0098	-2.75564	0.533486	-5.16534	2.40E-07	4.52E-06	GALNTL6
ITGB3BP	1358.456	-1.06002	0.205367	-5.16157	2.45E-07	4.60E-06	ITGB3BP
ALDH1L2	794.1936	-1.17451	0.227596	-5.16048	2.46E-07	4.62E-06	ALDH1L2
HIF1A	33080.98	-1.13021	0.219034	-5.15996	2.47E-07	4.63E-06	HIF1A
HMGN2P4	893.4476	-1.26314	0.245221	-5.15103	2.59E-07	4.83E-06	HMGN2P4
AUNIP	469.7604	-1.17254	0.227693	-5.14966	2.61E-07	4.86E-06	AUNIP
CPE	1214.546	1.03828	0.201903	5.142464	2.71E-07	5.03E-06	CPE
SPDEF	2473.903	-1.23385	0.239939	-5.14234	2.71E-07	5.03E-06	SPDEF
EFNB3	473.3623	1.326881	0.258393	5.135125	2.82E-07	5.20E-06	EFNB3
TMEM47	2211.52	1.041592	0.202894	5.133681	2.84E-07	5.24E-06	TMEM47
PID1	417.9184	1.265285	0.246525	5.13249	2.86E-07	5.26E-06	PID1
ISM2	49.37119	3.339707	0.651393	5.127021	2.94E-07	5.39E-06	ISM2
LOC10192	239.7873	1.71975	0.335924	5.119464	3.06E-07	5.59E-06	LOC10192
DARS-AS1	70.52448	2.582679	0.504923	5.114992	3.14E-07	5.71E-06	DARS-AS1
CAND1.11	81.81268	2.336833	0.458628	5.095273	3.48E-07	6.31E-06	CAND1.11
DOCK4	3318.348	1.108446	0.218128	5.081635	3.74E-07	6.74E-06	DOCK4
NFATC2	294.0123	-1.43123	0.281975	-5.07574	3.86E-07	6.94E-06	NFATC2
CNKSR3	1819.418	1.016428	0.200444	5.070894	3.96E-07	7.09E-06	CNKSR3
EFEMP2	206.8397	1.498263	0.295754	5.065915	4.06E-07	7.24E-06	EFEMP2
HCN2	931.0078	1.028926	0.203512	5.055855	4.28E-07	7.62E-06	HCN2
CENPU	1395.346	-1.06856	0.211503	-5.05222	4.37E-07	7.74E-06	CENPU
LOC10537	35.20307	5.539134	1.097061	5.049065	4.44E-07	7.85E-06	LOC10537
MSC	782.1262	1.013222	0.201239	5.034923	4.78E-07	8.41E-06	MSC
HAUS8	543.343	-1.12862	0.22433	-5.03107	4.88E-07	8.54E-06	HAUS8
SOX2	1025.254	-1.1932	0.237575	-5.0224	5.10E-07	8.91E-06	SOX2
SLCO1A2	269.4856	1.41282	0.281353	5.021522	5.13E-07	8.94E-06	SLCO1A2

NECTIN1	804.1215	-1.00806	0.200917	-5.01729	5.24E-07	9.10E-06	NECTIN1
YPEL3	405.9485	1.201672	0.239656	5.014142	5.33E-07	9.24E-06	YPEL3
WNT11	110.5073	2.271549	0.453218	5.012039	5.39E-07	9.34E-06	WNT11
ANK1	2530.182	1.232058	0.246121	5.005913	5.56E-07	9.61E-06	ANK1
KCNJ2	757.064	-1.40346	0.280444	-5.00442	5.60E-07	9.68E-06	KCNJ2
LOC10537	321.3512	1.292796	0.258377	5.003515	5.63E-07	9.71E-06	LOC10537
LINC00482	298.1097	1.30109	0.260049	5.00326	5.64E-07	9.72E-06	LINC00482
BORA	616.663	-1.06962	0.213797	-5.00296	5.65E-07	9.72E-06	BORA
GPR155	318.0777	1.261808	0.252481	4.997643	5.80E-07	9.97E-06	GPR155
SNHG9	193.963	-1.67317	0.334833	-4.99702	5.82E-07	9.99E-06	SNHG9
DKK3	2141.877	1.011634	0.202528	4.995037	5.88E-07	1.01E-05	DKK3
CHRD	82.67452	2.409229	0.482376	4.994509	5.90E-07	1.01E-05	CHRD
LOC10537	64.94977	2.555645	0.51287	4.983024	6.26E-07	1.07E-05	LOC10537
CDKN3	1719.938	-1.03568	0.208481	-4.96774	6.77E-07	1.15E-05	CDKN3
PLGRKT	799.1175	-1.01304	0.204103	-4.96336	6.93E-07	1.17E-05	PLGRKT
SESN3	935.223	-1.01622	0.204739	-4.96349	6.92E-07	1.17E-05	SESN3
IQCN	240.0752	1.41496	0.285158	4.962013	6.98E-07	1.18E-05	IQCN
C9orf152	396.7584	-1.52606	0.307742	-4.9589	7.09E-07	1.19E-05	C9orf152
LRRCC1	455.7212	-1.14559	0.231198	-4.95501	7.23E-07	1.22E-05	LRRCC1
VSTM2L	461.2682	1.491897	0.3011	4.954815	7.24E-07	1.22E-05	VSTM2L
PRKCQ-AS	477.7768	-1.11639	0.225349	-4.95405	7.27E-07	1.22E-05	PRKCQ-AS
LOC10536	137.9949	1.884777	0.380545	4.952831	7.31E-07	1.23E-05	LOC10536
POLR3K	586.7458	-1.11073	0.224552	-4.94641	7.56E-07	1.26E-05	POLR3K
ADCY9	3399.952	1.107881	0.22401	4.945673	7.59E-07	1.27E-05	ADCY9
CPP	262.169	1.642027	0.332098	4.944407	7.64E-07	1.27E-05	CPP
CDH3	86.76263	2.171962	0.439329	4.943812	7.66E-07	1.28E-05	CDH3
FXYD2	100.0568	2.584501	0.522994	4.941739	7.74E-07	1.29E-05	FXYD2
RASGRP3	322.0882	1.406496	0.284764	4.939167	7.85E-07	1.30E-05	RASGRP3
SDC4P	38.45439	3.745088	0.758584	4.936944	7.94E-07	1.31E-05	SDC4P
IGFBP1	2801.194	3.42087	0.69365	4.931695	8.15E-07	1.35E-05	IGFBP1
KRT222	364.8806	-1.23507	0.251258	-4.91557	8.85E-07	1.45E-05	KRT222
ENPEP	253.0009	-1.52917	0.311702	-4.90586	9.30E-07	1.52E-05	ENPEP
RPL17P50	106.4411	1.980883	0.40404	4.902693	9.45E-07	1.54E-05	RPL17P50
CCDC85A	565.0468	1.030995	0.210383	4.900558	9.56E-07	1.55E-05	CCDC85A
FXYD6	193.3348	1.890381	0.38639	4.892421	9.96E-07	1.61E-05	FXYD6
ARX	220.224	-1.7873	0.365426	-4.891	1.00E-06	1.62E-05	ARX
MTBP	938.8518	-1.03878	0.212443	-4.88969	1.01E-06	1.63E-05	MTBP
EDIL3	433.498	1.352334	0.277158	4.879292	1.06E-06	1.72E-05	EDIL3
NUS1	1033.094	-1.02877	0.210993	-4.87588	1.08E-06	1.74E-05	NUS1
GSTM3	883.3282	1.15067	0.236125	4.873145	1.10E-06	1.77E-05	GSTM3
PDZD4	300.3167	1.293787	0.266532	4.854145	1.21E-06	1.92E-05	PDZD4
PDZK1IP1	224.8522	1.835051	0.378134	4.85291	1.22E-06	1.93E-05	PDZK1IP1
ADGRG5	72.9314	-2.55157	0.525855	-4.85223	1.22E-06	1.93E-05	ADGRG5
LYPD1	426.1754	1.332895	0.274727	4.851703	1.22E-06	1.94E-05	LYPD1
MYH14	600.2984	-1.04957	0.216742	-4.84249	1.28E-06	2.02E-05	MYH14
CCDC183-	219.0123	1.521152	0.314289	4.839979	1.30E-06	2.04E-05	CCDC183-
PPARGC1E	406.38	-1.12562	0.232737	-4.83643	1.32E-06	2.07E-05	PPARGC1E
P2RX5	158.776	-1.60365	0.331615	-4.83588	1.33E-06	2.08E-05	P2RX5
LINC01116	359.9447	1.228387	0.254671	4.823425	1.41E-06	2.20E-05	LINC01116
CDH9	36.27761	-3.58514	0.744861	-4.81317	1.49E-06	2.30E-05	CDH9
KCTD16	96.82702	2.252659	0.468739	4.805783	1.54E-06	2.37E-05	KCTD16
ZSWIM5	803.2299	1.044059	0.217259	4.805587	1.54E-06	2.37E-05	ZSWIM5
FSTL4	651.0191	-1.09708	0.228662	-4.79783	1.60E-06	2.45E-05	FSTL4
TRNL2	72.42823	-2.49957	0.521099	-4.79672	1.61E-06	2.46E-05	TRNL2
TLR1	538.9233	-1.25653	0.262064	-4.79473	1.63E-06	2.48E-05	TLR1
LOC10013	252.3946	1.41829	0.295952	4.792297	1.65E-06	2.51E-05	LOC10013
RNF26	1257.477	-1.01801	0.212749	-4.78503	1.71E-06	2.59E-05	RNF26
WDR63	67.5603	-2.68357	0.560915	-4.78427	1.72E-06	2.60E-05	WDR63

CCDC184	466.7962	1.121169	0.234511	4.780875	1.75E-06	2.64E-05	CCDC184
LINC00672	180.2833	1.638991	0.343001	4.778387	1.77E-06	2.67E-05	LINC00672
STAT5A	596.8022	1.021438	0.213776	4.77807	1.77E-06	2.67E-05	STAT5A
SSPOP	446.4441	1.298004	0.271809	4.775422	1.79E-06	2.69E-05	SSPOP
MNS1	344.6563	-1.51559	0.317588	-4.77218	1.82E-06	2.73E-05	MNS1
DPY19L2	732.4104	1.04272	0.218758	4.766538	1.87E-06	2.80E-05	DPY19L2
SLC16A14	213.3924	-1.5974	0.335794	-4.75708	1.96E-06	2.92E-05	SLC16A14
PAQR9	352.4408	1.154361	0.243136	4.747797	2.06E-06	3.05E-05	PAQR9
PCSK1N	771.8213	1.201848	0.253144	4.747682	2.06E-06	3.05E-05	PCSK1N
C5orf34	736.2349	-1.06435	0.224306	-4.7451	2.08E-06	3.08E-05	C5orf34
MAP3K15	1031.784	1.010603	0.213066	4.743148	2.10E-06	3.11E-05	MAP3K15
WDR4	653.0394	-1.02418	0.215993	-4.74172	2.12E-06	3.13E-05	WDR4
SNORA64	770.3717	1.097934	0.231694	4.738727	2.15E-06	3.17E-05	SNORA64
KDM7A-D	178.0533	1.575332	0.33248	4.738132	2.16E-06	3.17E-05	KDM7A-D
LINC02086	525.5202	1.062208	0.224287	4.735923	2.18E-06	3.20E-05	LINC02086
ZNF470	688.1782	1.011025	0.214003	4.724359	2.31E-06	3.37E-05	ZNF470
STAC	648.1678	1.050108	0.222314	4.723542	2.32E-06	3.38E-05	STAC
ARSI	480.1037	1.334596	0.283061	4.714866	2.42E-06	3.51E-05	ARSI
TRNR	65.43701	-2.51257	0.53317	-4.71252	2.45E-06	3.54E-05	TRNR
LINC01433	142.6394	-1.64073	0.348889	-4.70274	2.57E-06	3.70E-05	LINC01433
FNDC4	127.147	1.912945	0.40829	4.685258	2.80E-06	3.99E-05	FNDC4
ITGA6-AS1	41.91226	3.207855	0.684879	4.68383	2.82E-06	4.01E-05	ITGA6-AS1
ADAT2	473.7176	-1.09629	0.234168	-4.68166	2.85E-06	4.05E-05	ADAT2
ADRA2C	546.5078	1.199431	0.25624	4.680891	2.86E-06	4.05E-05	ADRA2C
LOC10537	606.9743	-1.10114	0.235236	-4.68101	2.85E-06	4.05E-05	LOC10537
NCMAP	479.8707	-1.26594	0.271084	-4.66992	3.01E-06	4.25E-05	NCMAP
EVA1A	593.1993	1.133978	0.242937	4.667789	3.04E-06	4.29E-05	EVA1A
RAD51	487.4161	-1.06621	0.228631	-4.66347	3.11E-06	4.37E-05	RAD51
PCDHGC3	254.1243	-1.39646	0.299787	-4.65818	3.19E-06	4.47E-05	PCDHGC3
UNC13C	268.3155	1.521078	0.326713	4.655702	3.23E-06	4.52E-05	UNC13C
LOC11226	82.33494	2.304785	0.495078	4.655398	3.23E-06	4.52E-05	LOC11226
SNORA46	95.35544	1.956273	0.420466	4.65263	3.28E-06	4.57E-05	SNORA46
CRISP3	77.5395	-2.87939	0.619057	-4.65125	3.30E-06	4.60E-05	CRISP3
KIAA0513	1093.754	1.011104	0.217836	4.641577	3.46E-06	4.81E-05	KIAA0513
SNORD94	519.6973	1.043109	0.225104	4.633905	3.59E-06	4.97E-05	SNORD94
CSPG4	181.4687	1.537074	0.331746	4.633294	3.60E-06	4.98E-05	CSPG4
TMEM105	201.0026	1.402027	0.30274	4.631129	3.64E-06	5.02E-05	TMEM105
LOC11226	316.3556	1.208899	0.261108	4.629888	3.66E-06	5.04E-05	LOC11226
LINC00893	325.6946	1.250029	0.270035	4.629134	3.67E-06	5.06E-05	LINC00893
LOC10537	55.29967	-2.62469	0.567203	-4.62743	3.70E-06	5.08E-05	LOC10537
SPEG	291.0158	1.329639	0.287379	4.626779	3.71E-06	5.09E-05	SPEG
DGAT2	139.1613	-1.78152	0.385862	-4.617	3.89E-06	5.31E-05	DGAT2
RPIA	463.5685	-1.04003	0.225587	-4.61032	4.02E-06	5.47E-05	RPIA
PCDHGB5	235.2163	1.296132	0.281643	4.602043	4.18E-06	5.66E-05	PCDHGB5
LOC10537	344.5668	1.124219	0.244384	4.600217	4.22E-06	5.70E-05	LOC10537
NEXMIF	46.22915	2.872898	0.625704	4.591469	4.40E-06	5.92E-05	NEXMIF
LOC10537	43.93687	4.268427	0.930213	4.588655	4.46E-06	5.99E-05	LOC10537
LOC10798	18.92319	7.72492	1.68502	4.584467	4.55E-06	6.09E-05	LOC10798
ACTR3B	375.7517	-1.11233	0.242694	-4.58325	4.58E-06	6.12E-05	ACTR3B
RNA5SP35	27.01284	-5.14438	1.122682	-4.58222	4.60E-06	6.15E-05	RNA5SP35
LOC10537	63.33194	-2.50329	0.547184	-4.57486	4.77E-06	6.35E-05	LOC10537
GRHL3	351.6538	-1.56925	0.34322	-4.57214	4.83E-06	6.43E-05	GRHL3
LOC10537	41.33355	3.090102	0.676376	4.568613	4.91E-06	6.53E-05	LOC10537
CLEC2B	359.992	1.18519	0.259525	4.566765	4.95E-06	6.56E-05	CLEC2B
PCK1	64.12644	2.60782	0.571387	4.564017	5.02E-06	6.62E-05	PCK1
KCNK15	104.4024	2.029358	0.444883	4.561556	5.08E-06	6.69E-05	KCNK15
EPHB3	134.3958	-1.67894	0.368588	-4.55506	5.24E-06	6.89E-05	EPHB3
LOC10798	117.7766	1.965346	0.432378	4.545433	5.48E-06	7.18E-05	LOC10798

LOC10798	62.50159	2.37411	0.522434	4.544327	5.51E-06	7.22E-05	LOC10798
NPY2R	316.3836	-1.36832	0.301128	-4.54397	5.52E-06	7.22E-05	NPY2R
ZNF542P	349.0156	1.132681	0.249651	4.537056	5.70E-06	7.43E-05	ZNF542P
IL31RA	383.467	1.195416	0.26379	4.531702	5.85E-06	7.61E-05	IL31RA
BTG2	1098.825	-1.14871	0.253497	-4.53145	5.86E-06	7.61E-05	BTG2
CPQ	316.2929	1.203746	0.265985	4.525619	6.02E-06	7.80E-05	CPQ
CREG2	311.2811	1.345732	0.297359	4.525614	6.02E-06	7.80E-05	CREG2
DCLK1	54.6345	-2.9978	0.663095	-4.52092	6.16E-06	7.96E-05	DCLK1
LOC10192	78.86229	2.055812	0.457519	4.493391	7.01E-06	8.96E-05	LOC10192
SORBS1	201.4644	-1.40868	0.313595	-4.49202	7.06E-06	9.01E-05	SORBS1
C18orf54	474.5807	-1.24558	0.277479	-4.4889	7.16E-06	9.12E-05	C18orf54
LOC10537	393.5049	1.247888	0.278096	4.487249	7.21E-06	9.18E-05	LOC10537
SLC9A3-A	234.3367	1.273255	0.283867	4.485389	7.28E-06	9.24E-05	SLC9A3-A
GTF2IP7	254.6401	1.258038	0.280493	4.485094	7.29E-06	9.25E-05	GTF2IP7
OAF	408.0641	-1.08957	0.243088	-4.48219	7.39E-06	9.37E-05	OAF
RRM2	3868.41	-1.07294	0.239647	-4.47716	7.56E-06	9.58E-05	RRM2
SYCE1L	250.4885	1.309816	0.293172	4.467734	7.91E-06	9.97E-05	SYCE1L
PAQR8	592.664	1.157317	0.259221	4.464603	8.02E-06	0.000100985	PAQR8
OR51E1	26.08467	-4.67072	1.046742	-4.46215	8.11E-06	0.000101938	OR51E1
ADRA1D	239.0012	-1.4501	0.324995	-4.46193	8.12E-06	0.000101973	ADRA1D
MSTO1	349.6886	-1.08025	0.242117	-4.46169	8.13E-06	0.000102019	MSTO1
SLC45A1	444.028	1.221834	0.27415	4.456811	8.32E-06	0.000104082	SLC45A1
WFDC21P	721.9223	4.047182	0.90897	4.452493	8.49E-06	0.000105963	WFDC21P
RELA-DT	557.2209	1.096537	0.246693	4.444945	8.79E-06	0.000109416	RELA-DT
MZT1	1022.084	-1.07837	0.244058	-4.41849	9.94E-06	0.000122028	MZT1
BARX1-DT	39.54536	2.923582	0.662531	4.412747	1.02E-05	0.000124808	BARX1-DT
SLC6A8	499.9609	1.251827	0.284027	4.407417	1.05E-05	0.000127408	SLC6A8
SUSD4	240.9992	1.280002	0.292392	4.37769	1.20E-05	0.000144833	SUSD4
WNT9A	239.0327	1.270276	0.290238	4.376662	1.21E-05	0.000145421	WNT9A
LOC10536	35.37763	3.360142	0.767816	4.376233	1.21E-05	0.000145612	LOC10536
LOC10798	391.0429	1.111742	0.25455	4.367476	1.26E-05	0.000151274	LOC10798
ARHGAP3	699.0946	1.148284	0.263476	4.358206	1.31E-05	0.000157308	ARHGAP3
SIDT1	571.0751	-1.12957	0.259394	-4.35466	1.33E-05	0.000159561	SIDT1
SNORA80	287.2425	1.578814	0.363033	4.34895	1.37E-05	0.000163348	SNORA80
LOC10192	182.3868	1.578294	0.363394	4.343203	1.40E-05	0.000167138	LOC10192
TIAM1	146.3191	-1.4981	0.345361	-4.33779	1.44E-05	0.00017064	TIAM1
TIMP4	1515.418	1.02466	0.236412	4.334207	1.46E-05	0.000173221	TIMP4
HOXD9	60.88665	2.23795	0.516923	4.329364	1.50E-05	0.000176731	HOXD9
DNAH3	237.6187	1.368289	0.316327	4.325552	1.52E-05	0.000179354	DNAH3
SPTB	1259.656	1.018115	0.235417	4.32474	1.53E-05	0.000179784	SPTB
ALOX5AP	49.78621	2.700645	0.625394	4.318308	1.57E-05	0.000184158	ALOX5AP
SLC22A4	376.0616	1.021455	0.236801	4.313553	1.61E-05	0.000187925	SLC22A4
OSBPL6	225.0866	-1.2998	0.301394	-4.31263	1.61E-05	0.000188591	OSBPL6
AMY2B	132.7052	1.535996	0.356302	4.310934	1.63E-05	0.000189682	AMY2B
LOC10537	41.04448	3.078749	0.714258	4.310415	1.63E-05	0.000189886	LOC10537
SUCNR1	107.8414	-1.81666	0.421681	-4.30813	1.65E-05	0.000191496	SUCNR1
RIBC2	259.1766	-1.16198	0.270039	-4.30299	1.69E-05	0.000195124	RIBC2
PTK6	297.3054	1.233826	0.287896	4.285664	1.82E-05	0.000209913	PTK6
FAM72C	91.57278	-1.98557	0.463356	-4.28518	1.83E-05	0.000210234	FAM72C
GRIN3B	200.247	1.322241	0.309042	4.278522	1.88E-05	0.000216083	GRIN3B
MPV17L2	404.8502	-1.26116	0.295431	-4.26887	1.96E-05	0.00022403	MPV17L2
CLEC2D	311.1156	1.087356	0.254838	4.266857	1.98E-05	0.000225853	CLEC2D
FAM13A-A	86.55511	1.88837	0.443322	4.259593	2.05E-05	0.000232309	FAM13A-A
CYP26A1	152.4336	1.704702	0.400264	4.258945	2.05E-05	0.00023284	CYP26A1
HOXC8	124.1793	-1.56714	0.368144	-4.25688	2.07E-05	0.000234275	HOXC8
KRT83	434.112	-1.04068	0.244592	-4.25476	2.09E-05	0.000236074	KRT83
VXN	42.49727	2.97527	0.699916	4.250893	2.13E-05	0.000239888	VXN
ADAMTS9	36.09477	3.808281	0.896976	4.245687	2.18E-05	0.000244177	ADAMTS9

SLC2A5	32.00311	3.308187	0.779996	4.241284	2.22E-05	0.000248865	SLC2A5
C19orf71	278.1846	1.152538	0.272189	4.234335	2.29E-05	0.000254974	C19orf71
BMP2KL	81.12462	1.88972	0.446331	4.233902	2.30E-05	0.000255311	BMP2KL
DACT2	533.0515	-1.15053	0.271888	-4.23162	2.32E-05	0.00025776	DACT2
SRPX2	578.0658	1.377076	0.326004	4.224103	2.40E-05	0.000265325	SRPX2
LOC10537	363.631	1.013986	0.240143	4.222431	2.42E-05	0.000266461	LOC10537
FAM43B	244.3946	1.693044	0.401426	4.217572	2.47E-05	0.000271716	FAM43B
NIM1K	58.84441	2.247149	0.533758	4.210052	2.55E-05	0.00028008	NIM1K
TUBB4A	331.9762	-1.20302	0.286558	-4.19818	2.69E-05	0.000293772	TUBB4A
LOC10537	86.31013	-1.86362	0.444064	-4.19673	2.71E-05	0.000295474	LOC10537
LOC10537	88.8737	2.067698	0.49331	4.191476	2.77E-05	0.000301686	LOC10537
LOC10537	65.14816	2.176002	0.519184	4.191196	2.77E-05	0.00030188	LOC10537
LOC64284	254.0242	-1.17557	0.280765	-4.18702	2.83E-05	0.000306937	LOC64284
LOC11226	311.5733	1.163098	0.277799	4.186828	2.83E-05	0.000307016	LOC11226
TMEM132	33.0486	3.147889	0.75223	4.184743	2.85E-05	0.000308935	TMEM132
RNF138P1	275.4608	1.508559	0.360555	4.183987	2.86E-05	0.000309781	RNF138P1
LOC10537	75.83402	2.059796	0.492929	4.178683	2.93E-05	0.000315975	LOC10537
TF	1418.008	2.243742	0.537389	4.175266	2.98E-05	0.000320381	TF
DRAIC	96.55613	-1.83225	0.439008	-4.17361	3.00E-05	0.000322148	DRAIC
LOC11226	104.2386	1.810011	0.434096	4.169611	3.05E-05	0.000326899	LOC11226
RGS9	85.12755	1.867238	0.448487	4.163419	3.14E-05	0.000335112	RGS9
ITGB8	1256.017	1.066958	0.2564	4.161308	3.16E-05	0.000337832	ITGB8
HSPA1B	172.8107	-1.87073	0.450114	-4.15613	3.24E-05	0.000344173	HSPA1B
GPR160	474.6748	1.002219	0.241644	4.1475	3.36E-05	0.00035596	GPR160
ADH1C	253.3267	-3.91352	0.944684	-4.14268	3.43E-05	0.000362685	ADH1C
LMNTD2	137.9786	1.467745	0.354458	4.140812	3.46E-05	0.000364606	LMNTD2
ATL1	373.5775	1.038853	0.250982	4.139157	3.49E-05	0.000366614	ATL1
EBF1	292.9218	-1.19553	0.288926	-4.13785	3.51E-05	0.000368237	EBF1
GSN-AS1	180.8342	1.281347	0.311135	4.118306	3.82E-05	0.000397985	GSN-AS1
FBXL8	313.8504	1.056712	0.256673	4.11696	3.84E-05	0.000399633	FBXL8
KLHL13	179.5187	-1.34526	0.327668	-4.10555	4.03E-05	0.000417282	KLHL13
VASH2	356.5074	-1.12121	0.273331	-4.10203	4.10E-05	0.000423434	VASH2
LINC0270	166.3916	1.316012	0.321394	4.094703	4.23E-05	0.000434827	LINC0270
PRODH2	114.622	-1.68638	0.411869	-4.09445	4.23E-05	0.000434827	PRODH2
LOC10537	120.8727	1.596665	0.390362	4.090212	4.31E-05	0.000441375	LOC10537
HMGB3P3	27.42664	-3.4802	0.85146	-4.08733	4.36E-05	0.000445891	HMGB3P3
A2M	51.75372	2.358833	0.577276	4.086144	4.39E-05	0.00044735	A2M
ARID5A	248.831	1.302208	0.318699	4.08601	4.39E-05	0.00044735	ARID5A
SNX29	1662.428	1.070289	0.262137	4.082943	4.45E-05	0.000451632	SNX29
EIF4EP1	91.52723	-1.8784	0.460124	-4.08239	4.46E-05	0.000452455	EIF4EP1
PZP	34.29059	3.452054	0.845919	4.080832	4.49E-05	0.000454748	PZP
KBTBD8	86.77599	-1.96347	0.482241	-4.07156	4.67E-05	0.000470894	KBTBD8
MSLN	109.4156	1.601115	0.393758	4.066244	4.78E-05	0.000480704	MSLN
SALL1	180.2661	-1.43526	0.353112	-4.06459	4.81E-05	0.000483585	SALL1
HMGN5	113.515	-1.60932	0.397058	-4.05311	5.05E-05	0.000506574	HMGN5
CCAT1	195.3795	-1.66062	0.410285	-4.04747	5.18E-05	0.000517229	CCAT1
RNU5D-1	262.2013	-1.23761	0.305801	-4.04711	5.19E-05	0.00051774	RNU5D-1
LINC0150	312.0393	-1.03112	0.255021	-4.04329	5.27E-05	0.000525402	LINC01504
LOC10537	115.0987	1.544226	0.382017	4.042295	5.29E-05	0.000527066	LOC10537
TIMP3	69.71347	2.976529	0.736586	4.040978	5.32E-05	0.000529459	TIMP3
SNORA48	28.0181	3.604062	0.893753	4.032503	5.52E-05	0.000547145	SNORA48
FAM72D	86.75168	-1.88608	0.468207	-4.02831	5.62E-05	0.000555505	FAM72D
PSORS1C	34.40452	2.97741	0.739805	4.024589	5.71E-05	0.000563439	PSORS1C3
LOC10537	94.06165	1.78032	0.442965	4.019095	5.84E-05	0.000573957	LOC10537
KIAA1755	59.47621	2.113182	0.526814	4.011249	6.04E-05	0.000591789	KIAA1755
BCAT1	2405.356	-2.21631	0.552612	-4.0106	6.06E-05	0.000593096	BCAT1
TTPA	77.55114	-1.87912	0.470348	-3.99517	6.46E-05	0.00062939	TTPA
AJM1	283.8881	1.068724	0.267559	3.994348	6.49E-05	0.000631243	AJM1

LINC0023٩	75.46473	1.871567	0.469201	3.988839	6.64E-05	0.000643691	LINC0023٩
SALRNA2	38.19255	2.757955	0.692534	3.982409	6.82E-05	0.00065823	SALRNA2
IGFBP3	45880.51	2.623554	0.66054	3.971833	7.13E-05	0.000683839	IGFBP3
RHCG	291.1147	1.064777	0.268262	3.969166	7.21E-05	0.000689016	RHCG
LOC28460	169.6168	-1.34371	0.339145	-3.96206	7.43E-05	0.000707656	LOC28460
CERS1	90.53051	1.856254	0.469059	3.957401	7.58E-05	0.000720833	CERS1
TRIM46	328.4685	1.053719	0.266336	3.956349	7.61E-05	0.000723639	TRIM46
OSER1-DT	244.1288	1.097048	0.277433	3.954276	7.68E-05	0.000728922	OSER1-DT
HHLA2	36.5827	2.946466	0.745333	3.95322	7.71E-05	0.000731277	HHLA2
LOC10537	21.5219	-6.39431	1.619015	-3.9495	7.83E-05	0.000740804	LOC10537
WNT1	27.75074	3.223975	0.818078	3.940913	8.12E-05	0.000765086	WNT1
FAM229A	97.22433	1.617514	0.411144	3.934179	8.35E-05	0.000782832	FAM229A
KCNJ6	138.4267	-1.39621	0.355152	-3.93131	8.45E-05	0.000791417	KCNJ6
PCDH1	333.4617	1.113103	0.283209	3.93032	8.48E-05	0.000793881	PCDH1
LPCAT1	124.3518	1.547128	0.394345	3.923281	8.74E-05	0.00081495	LPCAT1
KCNG1	227.7548	-1.13169	0.289065	-3.915	9.04E-05	0.000839595	KCNG1
MKLN1-AS	496.1004	1.147138	0.293357	3.910387	9.21E-05	0.00085406	MKLN1-AS
PRKG1	54.40834	-2.31163	0.591226	-3.90989	9.23E-05	0.000854962	PRKG1
SEMA3F	319.8291	-1.17972	0.301756	-3.9095	9.25E-05	0.000855899	SEMA3F
SNORA8	351.8165	1.042485	0.266676	3.909184	9.26E-05	0.000856594	SNORA8
EPPK1	75.70539	2.563497	0.656126	3.907016	9.34E-05	0.000862571	EPPK1
TRNW	50.66072	-2.24091	0.574054	-3.90366	9.47E-05	0.000873297	TRNW
PITPNM3	474.9088	1.053805	0.270291	3.898774	9.67E-05	0.000890668	PITPNM3
ABCA8	235.8834	-1.59128	0.408428	-3.8961	9.78E-05	0.00089875	ABCA8
ZBTB46	333.8055	1.004705	0.258333	3.889189	0.00010058	0.000921036	ZBTB46
LOC10798	37.68692	-2.62701	0.675519	-3.88887	0.00010071	0.000921772	LOC10798
FGFR2	326.3321	-1.28849	0.331818	-3.88312	0.000103127	0.000942475	FGFR2
LOC10050	39.61764	2.523987	0.650958	3.877345	0.000105603	0.000959843	LOC10050
ARG2	167.9991	1.492255	0.384973	3.876259	0.000106075	0.000963656	ARG2
C5orf64	30.20495	-3.2331	0.835371	-3.87026	0.000108719	0.000984754	C5orf64
CCL28	118.9823	1.607911	0.415747	3.867519	0.000109948	0.000992946	CCL28
SYNGR4	37.87566	-2.63664	0.682829	-3.86135	0.000112762	0.00101635	SYNGR4
RAPGEF4	263.8033	1.06312	0.275482	3.859124	0.000113794	0.001024146	RAPGEF4
SSTR5-AS1	43.30746	-2.72666	0.706822	-3.85764	0.000114488	0.001028879	SSTR5-AS1
GPR141	66.89876	-1.91983	0.497807	-3.85659	0.000114981	0.001032299	GPR141
EXOC3L2	89.88052	1.665139	0.432153	3.853124	0.00011662	0.001044969	EXOC3L2
EDNRA	261.6214	-1.28963	0.33608	-3.83729	0.000124402	0.001108734	EDNRA
GNRH1	277.2848	1.008614	0.263067	3.834061	0.000126045	0.001122289	GNRH1
GTF2IRD2	113.1281	1.452589	0.379168	3.830989	0.000127629	0.001134193	GTF2IRD2
EMILIN3	70.77378	2.400371	0.627403	3.825883	0.000130304	0.001154609	EMILIN3
PLAC4	184.616	1.235158	0.322967	3.824415	0.000131083	0.001160387	PLAC4
PACSIN1	104.2307	-1.92995	0.505624	-3.81697	0.0001351	0.001187348	PACSIN1
LOC10192	148.4131	1.338116	0.350686	3.81571	0.000135792	0.001192292	LOC10192
LOC10537	36.06209	2.663478	0.698559	3.812817	0.000137392	0.001205186	LOC10537
MAP6	273.4267	1.169653	0.306884	3.81138	0.000138193	0.001211633	MAP6
TMEM38A	161.1036	1.246417	0.327395	3.807073	0.000140621	0.001230571	TMEM38A
BAIAP2L2	365.4738	-1.23223	0.323826	-3.80521	0.000141684	0.001238734	BAIAP2L2
TRNI	21.33767	-3.6962	0.971986	-3.80273	0.000143112	0.001249981	TRNI
FAM153B	44.9339	2.45727	0.647415	3.795509	0.000147341	0.001280437	FAM153B
LOC10537	246.3249	1.049261	0.276451	3.795465	0.000147367	0.001280437	LOC10537
TMPRSS3	43.46202	2.393505	0.631206	3.791955	0.000149466	0.001295609	TMPRSS3
FBXO2	1286.304	2.267415	0.597999	3.791672	0.000149636	0.001296473	FBXO2
DIRAS3	158.5209	-1.31217	0.347095	-3.78045	0.000156548	0.001347443	DIRAS3
PLXNC1	250.4453	-1.20307	0.318737	-3.77449	0.000160333	0.001376151	PLXNC1
LOC11226	253.0393	-1.09878	0.291186	-3.77348	0.000160988	0.001379833	LOC11226
EEF1A1P12	106.687	1.489147	0.394775	3.772138	0.000161855	0.001384674	EEF1A1P12
LOC10537	67.32938	1.83726	0.487052	3.772201	0.000161814	0.001384674	LOC10537
LINC02736	92.26347	1.569961	0.416912	3.765685	0.000166093	0.001417625	LINC02736

ADGRF3	116.1286	1.525366	0.406171	3.755476	0.000173012	0.001472572	ADGRF3
LOC10537	66.74116	1.913023	0.509922	3.751598	0.000175711	0.001492081	LOC10537
PRSS27	148.2385	1.284469	0.343116	3.743543	0.000181444	0.001535782	PRSS27
LECT2	25.73769	3.269803	0.873511	3.743288	0.000181627	0.001536424	LECT2
LOC11226	84.05266	1.639507	0.438278	3.74079	0.000183443	0.001546993	LOC11226
ITGA4	292.049	1.18572	0.317171	3.73843	0.000185173	0.001560865	ITGA4
F2RL2	257.7777	-1.18137	0.316019	-3.7383	0.00018527	0.001560965	F2RL2
EFCAB10	120.3085	-1.41849	0.379537	-3.73743	0.000185913	0.001562075	EFCAB10
KLRC2	250.3746	1.177684	0.315092	3.737585	0.000185796	0.001562075	KLRC2
MEIOB	224.3404	1.267156	0.338998	3.737947	0.000185529	0.001562075	MEIOB
KLF17	54.38228	2.071585	0.555553	3.728868	0.000192342	0.001608723	KLF17
TUBB2B	236.0861	1.11102	0.299113	3.714385	0.000203698	0.00169527	TUBB2B
FAM167B	148.4881	1.470547	0.396264	3.711032	0.000206416	0.001715492	FAM167B
SNORA71I	229.3508	1.158094	0.312068	3.711032	0.000206416	0.001715492	SNORA71I
SECTM1	88.94558	1.689	0.455201	3.710452	0.000206889	0.001718642	SECTM1
RAB3IL1	257.0942	-1.02023	0.275336	-3.70541	0.000211046	0.001747631	RAB3IL1
ZSCAN18	256.6639	1.003377	0.271066	3.701594	0.000214249	0.001770163	ZSCAN18
FAM228B	369.6764	1.06121	0.286767	3.700605	0.000215086	0.001775475	FAM228B
LOC10537	187.0121	1.290182	0.348755	3.69939	0.000216119	0.001782396	LOC10537
GRHL1	68.54124	-1.86178	0.50342	-3.69827	0.000217077	0.001789497	GRHL1
MKRN5P	123.0127	1.355175	0.366904	3.693545	0.00022115	0.001816537	MKRN5P
TG	91.76105	1.682963	0.455835	3.692044	0.000222459	0.001825655	TG
HPSE	96.40455	-1.81539	0.492305	-3.68753	0.000226445	0.001855048	HPSE
LINC01138	183.5117	1.137582	0.308848	3.683301	0.000230233	0.001880205	LINC01138
BIRC7	321.9043	1.032211	0.280329	3.682135	0.000231289	0.001887985	BIRC7
LOC10798	117.8964	-1.41404	0.384078	-3.68164	0.000231734	0.001889937	LOC10798
LOC11226	54.74952	2.066307	0.561479	3.680112	0.000233131	0.001898799	LOC11226
SSR4P1	120.8598	1.430664	0.388969	3.678095	0.000234983	0.001911333	SSR4P1
DNER	172.5266	1.24265	0.337902	3.677547	0.000235488	0.001914589	DNER
PCCA-DT	258.2036	-1.00067	0.272246	-3.6756	0.000237294	0.001926716	PCCA-DT
LINC00310	25.36882	3.831097	1.042425	3.675179	0.000237683	0.001929017	LINC00310
FER1L6	193.5601	-1.48508	0.404462	-3.67174	0.000240903	0.001952561	FER1L6
RPS24P15	41.28086	-2.31428	0.630331	-3.67153	0.000241102	0.001953312	RPS24P15
HLX	220.0589	1.138994	0.310768	3.665089	0.000247253	0.001995331	HLX
LOC10710	53.93929	2.556014	0.69758	3.664115	0.000248195	0.002001935	LOC10710
LOC11226	69.32786	-1.79004	0.489829	-3.65441	0.000257775	0.002071004	LOC11226
ITGBL1	60.29291	1.898349	0.520572	3.646657	0.000265674	0.002127947	ITGBL1
ICAM1	125.2652	1.515321	0.415919	3.643304	0.000269161	0.002153053	ICAM1
ANXA10	232.2609	-1.33481	0.367137	-3.63572	0.000277207	0.002207784	ANXA10
PHYHIP	122.1744	1.460795	0.402109	3.632835	0.000280324	0.002229708	PHYHIP
CRMP1	157.8945	1.204725	0.331657	3.632442	0.000280751	0.002230204	CRMP1
KRT15	220.7085	-1.56907	0.432144	-3.6309	0.00028244	0.002241673	KRT15
ANO7	176.0943	1.178632	0.324661	3.63034	0.000283048	0.002245527	ANO7
CORO2B	94.45174	1.505429	0.414749	3.629739	0.000283708	0.002249791	CORO2B
PNCK	31.83751	3.458677	0.953642	3.626808	0.000286946	0.002272527	PNCK
MACROD2	160.902	1.177523	0.325929	3.612822	0.000302882	0.002378532	MACROD2
LAYN	173.8569	1.253322	0.347714	3.604457	0.000312807	0.00244461	LAYN
LOC10537	106.336	-1.48618	0.413087	-3.59773	0.000321013	0.002501281	LOC10537
HSD11B1L	287.1873	1.014352	0.282157	3.594994	0.000324399	0.002525521	HSD11B1L
FGF17	81.8731	1.700774	0.473767	3.589896	0.00033081	0.00257216	FGF17
SYNE3	108.6054	1.454356	0.406754	3.575514	0.000349541	0.002697214	SYNE3
LOC10798	105.6074	1.393656	0.390228	3.571393	0.000355088	0.002731974	LOC10798
ADAMTSL3	56.74476	2.041514	0.571833	3.570122	0.000356815	0.002742956	ADAMTSL3
ENKUR	140.6845	1.286988	0.360672	3.568303	0.000359301	0.002757445	ENKUR
TBC1D9B	129.3704	1.521868	0.426857	3.565291	0.000363453	0.002783154	TBC1D9B
LOC10537	41.71754	2.261161	0.634467	3.563875	0.00036542	0.002791556	LOC10537
LOC10798	65.88312	1.748635	0.491292	3.55926	0.000371902	0.002833993	LOC10798
PKD1L2	1142.305	2.25844	0.635073	3.556189	0.000376273	0.002861361	PKD1L2

GCOM1	82.94789	2.041856	0.574291	3.555435	0.000377355	0.00286602	GCOM1
SPNS2	341.1007	1.022878	0.287935	3.552461	0.000381645	0.002893814	SPNS2
CFAP47	148.5207	-1.27424	0.358951	-3.54991	0.000385369	0.002917223	CFAP47
ABHD12B	57.72651	1.902887	0.536373	3.547693	0.00038862	0.002936991	ABHD12B
DPF3	20.1358	3.92025	1.105553	3.545963	0.000391181	0.002951481	DPF3
CDH4	186.3061	1.097686	0.310584	3.53426	0.000408919	0.003058886	CDH4
PCDHGB4	168.3607	1.14688	0.324544	3.533817	0.000409605	0.003061522	PCDHGB4
PCAT6	84.79679	1.546456	0.437691	3.533215	0.000410539	0.003067254	PCAT6
LOC10536	142.3926	1.238387	0.350986	3.528307	0.000418227	0.003115811	LOC10536
ALK	159.0463	1.302713	0.36939	3.526661	0.000420835	0.003126352	ALK
GOLGA2P(	32.91408	2.547626	0.722474	3.526252	0.000421486	0.003127389	GOLGA2P(
DNAJB6P2	33.37002	2.618767	0.742699	3.526013	0.000421866	0.003127676	DNAJB6P2
RAPSN	29.65216	2.852506	0.811383	3.515611	0.000438743	0.003228012	RAPSN
NR4A2	253.7032	-1.15383	0.328743	-3.50982	0.000448411	0.003289113	NR4A2
VSIG1	135.4802	-1.42278	0.406665	-3.49866	0.000467598	0.003403446	VSIG1
CYP27C1	285.5687	1.235207	0.353354	3.495663	0.000472885	0.003427913	CYP27C1
ABCA10	128.3811	1.44707	0.414297	3.49283	0.000477931	0.003455635	ABCA10
LOC10537	84.21314	1.55681	0.446127	3.489609	0.000483727	0.003491747	LOC10537
RRAD	88.94429	1.516983	0.434972	3.48754	0.000487486	0.003514729	RRAD
NIBAN1	196.7533	-1.14652	0.329309	-3.48159	0.00049844	0.003583848	NIBAN1
HLA-B	43.5265	2.356072	0.677617	3.476996	0.000507065	0.003637301	HLA-B
HOXB2	170.8225	-1.11144	0.31968	-3.47672	0.000507582	0.003638165	HOXB2
ADAP1	232.8254	1.036862	0.298362	3.475182	0.000510507	0.003654847	ADAP1
KCNQ5	105.5213	-1.39883	0.402755	-3.47316	0.000514376	0.003679678	KCNQ5
FSIP1	205.9103	-1.03225	0.297225	-3.47297	0.000514736	0.003680814	FSIP1
SLC52A3	276.1669	1.170503	0.337056	3.472727	0.000515199	0.003681337	SLC52A3
AICDA	35.44573	2.3382	0.673882	3.469744	0.000520954	0.00371948	AICDA
SCRG1	60.03864	1.869751	0.54009	3.461927	0.000536322	0.003814357	SCRG1
IL33	39.75515	-2.67834	0.775414	-3.45408	0.000552178	0.003909863	IL33
LOC10537	91.3685	1.511422	0.437758	3.452642	0.000555125	0.003923742	LOC10537
LOC15843	54.09989	-1.88389	0.545937	-3.45075	0.000559033	0.003945282	LOC15843
NRAD1	270.2825	1.076438	0.311939	3.450801	0.000558925	0.003945282	NRAD1
DHRS2	56.14252	-1.98079	0.574177	-3.4498	0.000561012	0.003957722	DHRS2
LOC10192	34.0218	2.41274	0.6997	3.448249	0.000564233	0.003978918	LOC10192
LINC0220	30.15649	2.837883	0.823549	3.445919	0.00056912	0.004004143	LINC0220
LMNTD2-/	41.63367	2.141554	0.62183	3.443951	0.000573279	0.004028767	LMNTD2-/
NOX5	51.38034	2.032342	0.590918	3.439294	0.000583233	0.004089321	NOX5
CATSPERG	89.59985	1.469084	0.427274	3.438269	0.000585446	0.004098571	CATSPERG
H4C12	218.838	-1.03812	0.30201	-3.43736	0.000587413	0.004109205	H4C12
SNORD29	190.9803	-1.1292	0.328684	-3.4355	0.000591457	0.004133929	SNORD29
RNA5S9	120.3989	-4.32291	1.258672	-3.4345	0.000593648	0.004146496	RNA5S9
LOC10537	38.5287	-2.31718	0.67482	-3.43377	0.000595249	0.004152933	LOC10537
FNDC11	33.38812	2.781309	0.810971	3.429604	0.000604464	0.004207618	FNDC11
YPEL1	106.389	1.523728	0.444737	3.426137	0.000612232	0.004255231	YPEL1
RASSF7	158.011	1.334831	0.389716	3.425143	0.000614476	0.00426598	RASSF7
SLC6A12	2221.036	5.826427	1.702512	3.422254	0.000621044	0.004301805	SLC6A12
LOC10537	72.93171	-1.63889	0.478982	-3.42161	0.000622512	0.004310351	LOC10537
KCNC3	112.2624	1.358986	0.398826	3.407466	0.000655692	0.004504551	KCNC3
PPP1R3B	54.87657	1.874904	0.550912	3.403271	0.000665841	0.004562144	PPP1R3B
NPM1P29	52.66976	1.869164	0.549417	3.402084	0.00066874	0.004576878	NPM1P29
BOLA3-AS	217.9065	1.050093	0.308874	3.39974	0.000674499	0.004614571	BOLA3-AS
OLR1	46.9173	1.994606	0.586886	3.398625	0.000677255	0.004629974	OLR1
CD274	142.809	-1.21263	0.357126	-3.39553	0.000684954	0.004668683	CD274
SCG2	82.91333	-1.6723	0.49254	-3.39527	0.000685622	0.004671499	SCG2
CCL2	1697.646	3.276781	0.968249	3.384234	0.000713771	0.004822097	CCL2
LOC10798	277.8001	1.085963	0.321562	3.377151	0.000732408	0.004934847	LOC10798
TRAM1L1	141.039	-1.18035	0.349731	-3.37502	0.000738107	0.004962744	TRAM1L1
RAB11B-A	144.7966	1.196007	0.354485	3.373925	0.000741047	0.00498068	RAB11B-A

PTGER4	167.6227	-1.09563	0.324975	-3.37143	0.000747779	0.005014906	PTGER4
SLC25A48	22.25952	3.830045	1.136166	3.371026	0.000748889	0.005020512	SLC25A48
FAS-AS1	18.53186	-3.52639	1.046562	-3.3695	0.000753045	0.005039262	FAS-AS1
SLC7A9	121.6924	1.270478	0.377365	3.366708	0.000760711	0.005083048	SLC7A9
RAB42	65.58003	1.701344	0.505674	3.364507	0.000766806	0.005115454	RAB42
PLXDC1	97.02355	1.371775	0.407834	3.363562	0.000769434	0.005128258	PLXDC1
LINC02761	65.58835	1.633028	0.486561	3.356268	0.000790021	0.005255917	LINC02761
HSPA2	13564.03	1.903786	0.568255	3.350235	0.000807429	0.005348451	HSPA2
NXPE2	49.97825	1.927851	0.57591	3.347485	0.000815483	0.005397902	NXPE2
LOC10537	35.67135	2.404052	0.718582	3.34555	0.000821196	0.005429405	LOC10537
PKD2L1	98.71404	1.472644	0.440767	3.341089	0.000834504	0.005503947	PKD2L1
LINC01474	173.4609	1.05412	0.315703	3.338961	0.000840924	0.00553832	LINC01474
MFNG	138.8714	1.204882	0.360838	3.339121	0.00084044	0.00553832	MFNG
LOC10192	97.0217	1.591366	0.476838	3.337333	0.000845866	0.005566874	LOC10192
LOC10537	94.13357	1.466587	0.439805	3.334628	0.000854136	0.005609227	LOC10537
LINC01977	92.56706	1.496391	0.448887	3.333562	0.000857417	0.005626741	LINC01977
HAS1	104.1926	1.570046	0.47135	3.330955	0.000865487	0.005669556	HAS1
BEGAIN	206.2507	1.002253	0.301027	3.329443	0.000870199	0.005692129	BEGAIN
S100A2	153.559	1.266457	0.381557	3.319185	0.000902805	0.005878329	S100A2
LOC39156	51.82042	1.994725	0.601296	3.317377	0.000908668	0.00591021	LOC39156
CIART	47.78376	1.95966	0.590982	3.315938	0.00091336	0.005932312	CIART
ZNF684	176.464	-1.12214	0.338788	-3.31222	0.000925571	0.005992524	ZNF684
TRNH	88.15137	-2.06925	0.625098	-3.31029	0.000932005	0.006025677	TRNH
ZFP2	29.05191	2.487834	0.751855	3.308927	0.000936541	0.006048605	ZFP2
CELF5	130.8727	1.265331	0.382632	3.306912	0.000943306	0.006085869	CELF5
FAM131C	350.6422	1.015997	0.307293	3.306277	0.000945446	0.006097531	FAM131C
TBC1D10C	99.06779	1.330907	0.404	3.294325	0.000986584	0.006320615	TBC1D10C
N4BP2L1	97.88325	1.426664	0.434349	3.284602	0.001021266	0.006506446	N4BP2L1
CYS1	152.0139	1.104714	0.337397	3.27422	0.00105954	0.00671455	CYS1
LINC01151	20.29739	3.503153	1.069941	3.274154	0.001059787	0.00671455	LINC01151
UBE2QL1	26.17902	2.913211	0.891081	3.269301	0.001078134	0.006816664	UBE2QL1
POT1-AS1	35.07621	2.187632	0.66936	3.268246	0.001082163	0.006830365	POT1-AS1
AATK	111.6027	1.280685	0.391916	3.267756	0.001084037	0.006837483	AATK
G0S2	120.4985	1.221643	0.374083	3.265703	0.001091926	0.006882512	G0S2
CENPS	120.7268	-1.26445	0.38769	-3.26151	0.001108207	0.006957204	CENPS
EFCAB12	89.99305	-1.43109	0.439998	-3.2525	0.001143948	0.007139264	EFCAB12
LOC10537	18.40518	-3.25455	1.001884	-3.24843	0.001160427	0.007228455	LOC10537
LOC10192	122.3598	1.483047	0.456777	3.246762	0.001167261	0.007262517	LOC10192
EYA2	375.5809	1.004235	0.309515	3.244548	0.00117637	0.007314231	EYA2
BCL2A1	119.1521	1.54567	0.476963	3.240648	0.001192585	0.007402502	BCL2A1
SSTR5	66.93807	-1.67501	0.51711	-3.23917	0.001198792	0.007429431	SSTR5
GUCY1B1	69.01919	-1.58726	0.490149	-3.23833	0.001202324	0.007442801	GUCY1B1
SNORD19I	44.10396	-1.95928	0.606411	-3.23095	0.001233792	0.007611913	SNORD19I
PECAM1	59.47278	1.991662	0.617228	3.226788	0.001251883	0.007705384	PECAM1
GTF2IRD2I	148.4991	1.166591	0.361626	3.225956	0.001255526	0.007720033	GTF2IRD2I
OBSCN-A!	150.8509	1.090994	0.338207	3.225818	0.001256132	0.00772117	OBSCN-A!
TBX6	79.5976	1.558868	0.483655	3.223098	0.001268123	0.007779232	TBX6
LOC10798	29.98989	2.487354	0.771847	3.222598	0.001270336	0.0077824	LOC10798
DCST2	77.75269	1.453466	0.451103	3.222028	0.001272869	0.007795311	DCST2
LOC15005	38.54281	-2.10952	0.655316	-3.21909	0.001285995	0.007862571	LOC15005
KLF3-AS1	93.40162	1.365197	0.424149	3.218674	0.001287848	0.007871281	KLF3-AS1
CSPG4P5	101.9598	1.452153	0.451619	3.215441	0.001302443	0.007952531	CSPG4P5
LOC10537	85.19158	1.432742	0.446371	3.209754	0.001328488	0.008087328	LOC10537
RNF112	82.83708	1.394644	0.434575	3.209213	0.001330988	0.008099863	RNF112
FBXL16	171.6106	1.010048	0.314906	3.20746	0.001339126	0.008144581	FBXL16
LOC10192	92.94363	1.575923	0.491466	3.206576	0.001343249	0.00816635	LOC10192
NECTIN4	37.86057	-2.06101	0.643071	-3.20495	0.001350854	0.008198993	NECTIN4
FLVCR1-D	118.5197	-1.20491	0.376532	-3.20002	0.001374164	0.008315711	FLVCR1-D

CSTA	25.08607	-2.73989	0.856437	-3.19917	0.001378243	0.008337645	CSTA
FAM71E1	131.5628	1.146045	0.358356	3.198058	0.001383564	0.008358802	FAM71E1
SDC2	73.90816	1.500699	0.469456	3.196677	0.001390203	0.008396147	SDC2
GCNA	193.0641	1.041933	0.326951	3.186815	0.001438489	0.008631554	GCNA
VIL1	616.1894	1.008986	0.316765	3.185282	0.00144613	0.008671109	VIL1
SNORD14:	72.25871	1.549012	0.486973	3.180896	0.001468204	0.008791966	SNORD14:
LOC10798	116.1412	-1.19458	0.376239	-3.17506	0.001498033	0.008932677	LOC10798
RN7SL128	196.588	1.088495	0.342912	3.174271	0.001502136	0.008954235	RN7SL128I
LOC10537	111.8406	1.265248	0.399342	3.168335	0.001533149	0.009104078	LOC10537
PTPRQ	95.34236	1.390881	0.438994	3.168336	0.001533144	0.009104078	PTPRQ
SORBS2	81.7676	-1.41137	0.445755	-3.16624	0.001544236	0.009157574	SORBS2
PRKG1-AS	136.0961	-1.38272	0.437942	-3.1573	0.001592363	0.009406481	PRKG1-AS
KCNB1	105.7039	1.340831	0.424967	3.155141	0.001604205	0.009467285	KCNB1
MIR924HG	135.6316	-1.1997	0.38042	-3.15361	0.001612621	0.009507779	MIR924HG
LRRC37A3	312.6705	1.008915	0.320159	3.151297	0.001625471	0.00956816	LRRC37A3
TINAGL1	34.17986	2.234361	0.709186	3.150601	0.001629348	0.009584833	TINAGL1
SNORD30	90.68515	-1.43082	0.454257	-3.1498	0.001633809	0.009598756	SNORD30
DEPP1	93.64387	1.380793	0.43848	3.149045	0.001638051	0.009620602	DEPP1
ANO9	167.6754	1.111397	0.35356	3.143448	0.001669699	0.009781417	ANO9
PPBP	21.07929	2.788856	0.887577	3.142102	0.001677398	0.009810851	PPBP
PGK1P2	22.8739	2.647734	0.842908	3.14119	0.001682626	0.009838295	PGK1P2
LINC0265(	63.96217	2.309437	0.735427	3.140267	0.001687937	0.009863058	LINC0265(
GLS	36859.31	1.833759	0.58447	3.137475	0.001704099	0.00993534	GLS
ZNF785	83.79699	-1.39236	0.444087	-3.13533	0.001716617	0.009995613	ZNF785
CEP20	226.647	-1.05383	0.336202	-3.1345	0.001721443	0.010014182	CEP20
SLCO4A1	2826.496	2.840269	0.906777	3.132268	0.001734613	0.010086793	SLCO4A1
LOC64748	60.17703	1.592528	0.508608	3.131149	0.001741239	0.0101133	LOC64748
DDN-AS1	89.21835	1.339481	0.427989	3.129712	0.001749778	0.010150042	DDN-AS1
ZNF695	29.44561	-2.32502	0.74346	-3.1273	0.001764181	0.01022066	ZNF695
SPHK1	181.1225	1.118389	0.357712	3.126505	0.001768978	0.010245214	SPHK1
LOC10798	25.19322	3.052656	0.97687	3.124936	0.001778438	0.010287013	LOC10798
MLXIPL	155.397	-1.02723	0.329005	-3.12223	0.001794891	0.010362571	MLXIPL
RNU5F-1	108.2099	-1.21606	0.389587	-3.12141	0.001799875	0.010377619	RNU5F-1
FAM83A	169.2883	-1.03042	0.330478	-3.11798	0.001820971	0.010476866	FAM83A
CRYAB	203.1697	3.12364	1.002274	3.116554	0.001829783	0.010524263	CRYAB
PLD4	117.8595	1.166832	0.374857	3.112739	0.001853601	0.010631253	PLD4
SLC6A13	47.82962	2.680594	0.861549	3.111365	0.001862245	0.010677494	SLC6A13
LOC10537	119.7784	1.16881	0.375728	3.11079	0.001865875	0.01069496	LOC10537
CSMD3	133.0668	1.118947	0.359779	3.110099	0.00187025	0.010716687	CSMD3
ADH6	342.9508	-3.6162	1.163505	-3.10803	0.001883415	0.010775294	ADH6
NWD1	20.59124	-2.82323	0.908586	-3.10728	0.001888175	0.010795791	NWD1
SH3PXD2A	28.19456	-2.34872	0.756542	-3.10455	0.001905681	0.010889098	SH3PXD2A
SCDP1	89.99315	1.700204	0.548452	3.100002	0.001935195	0.011026756	SCDP1
LOC10669	22.67943	2.631305	0.849745	3.096581	0.001957661	0.011127184	LOC10669
MYO1A	90.88775	-1.52105	0.491477	-3.09485	0.001969116	0.011181897	MYO1A
MOV10L1	43.27184	5.811569	1.881566	3.088688	0.002010425	0.011395309	MOV10L1
GUSBP5	23.9251	2.614994	0.847303	3.086256	0.00202694	0.011474536	GUSBP5
IGFL2	3156.409	1.948147	0.631992	3.082547	0.00205237	0.011600792	IGFL2
FAM177B	33.51554	2.341899	0.760095	3.081063	0.002062629	0.011648009	FAM177B
LOC10537	139.9047	1.580282	0.513124	3.079726	0.002071915	0.011696847	LOC10537
RPLP1P6	54.99347	1.662952	0.541025	3.073705	0.002114181	0.011906143	RPLP1P6
TRNF	35.36923	-2.65006	0.862316	-3.07319	0.002117826	0.01191935	TRNF
ADCY8	53.7223	2.187325	0.711832	3.072811	0.002120529	0.011927243	ADCY8
LOC10537	100.693	-1.29654	0.422342	-3.06989	0.00214136	0.012025974	LOC10537
MAP3K20-	64.82505	-1.55649	0.507327	-3.06802	0.002154794	0.012097715	MAP3K20-
IL7R	39.97842	-2.16099	0.705497	-3.06307	0.00219079	0.01226976	IL7R
FAIM2	30.95647	-2.34254	0.765051	-3.06194	0.002199067	0.012308603	FAIM2
LOC10537	26.79375	-2.40501	0.786616	-3.05741	0.002232552	0.012476989	LOC10537

LOC11226	189.0005	1.079361	0.353071	3.057062	0.00223518	0.012487869	LOC11226
LINC01239	137.1896	1.067902	0.349528	3.055267	0.002248606	0.012555231	LINC01239
SNORD88	32.38255	-2.16824	0.709926	-3.05419	0.002256717	0.012596275	SNORD88
SLC2A14	186.2512	2.927096	0.959332	3.051181	0.002279435	0.012688737	SLC2A14
SLC4A8	271.3955	-1.07204	0.352174	-3.04406	0.002334066	0.012941789	SLC4A8
SCN3A	215.469	-1.32118	0.434167	-3.04302	0.002342169	0.012971033	SCN3A
WWC2-AS	78.51261	1.491173	0.490421	3.040597	0.002361095	0.0130522	WWC2-AS
TRNK	21.22998	-3.12144	1.028632	-3.03455	0.00240894	0.013264719	TRNK
KDELC1P1	65.81876	-1.56706	0.517007	-3.03102	0.002437305	0.013388755	KDELC1P1
TMEM26	38.77295	-1.96844	0.649757	-3.0295	0.002449607	0.01344023	TMEM26
PIGAP1	115.7923	-1.15224	0.380373	-3.02923	0.002451759	0.013448017	PIGAP1
TSPAN12	117.4564	-1.15668	0.382096	-3.0272	0.002468314	0.013530728	TSPAN12
FLJ45513	147.0242	1.181185	0.390339	3.02605	0.002477713	0.013570085	FLJ45513
STEAP3-A	82.73012	1.405932	0.464686	3.025554	0.002481779	0.013584241	STEAP3-AS
IFFO1	127.1854	1.112758	0.367974	3.024016	0.002494435	0.013645373	IFFO1
LOC10537	128.1808	1.115714	0.36925	3.021569	0.002514686	0.013739759	LOC10537
PTGS1	330.9129	2.362648	0.782297	3.020144	0.002526547	0.013796347	PTGS1
LOC10537	67.74838	-1.44792	0.480352	-3.01428	0.002575921	0.014036705	LOC10537
NUAK1	4054.414	1.73	0.5747	3.01027	0.002610159	0.014170802	NUAK1
TMEM25	83.25583	1.302149	0.432576	3.010221	0.002610573	0.014170802	TMEM25
CNOT3	139.3397	-1.03378	0.343797	-3.00696	0.002638748	0.014298352	CNOT3
SLC5A3	13157.78	2.481209	0.82523	3.006689	0.002641099	0.014306865	SLC5A3
LOC10798	19.8359	2.876302	0.957764	3.003142	0.002672073	0.014432029	LOC10798
CATSPERE	108.0302	1.168704	0.389353	3.001653	0.002685177	0.014481481	CATSPERE
LOC11226	149.073	-1.00979	0.336473	-3.0011	0.002690093	0.014503727	LOC11226
TOX3	461.9105	-1.00463	0.33479	-3.00077	0.00269297	0.01451065	TOX3
SOX21-AS	101.9738	-1.27309	0.424897	-2.99624	0.002733308	0.014684926	SOX21-AS
CDYL2	72.91244	1.40828	0.470163	2.995303	0.002741722	0.014712893	CDYL2
GFAP	142.0724	1.072516	0.358598	2.990856	0.002781965	0.014893987	GFAP
ACHE	43.0782	1.778624	0.595194	2.988307	0.002805276	0.014983801	ACHE
FAM153A	17.95001	3.012454	1.009289	2.984728	0.002838309	0.015129401	FAM153A
LOC10192	61.04621	1.554182	0.520969	2.983252	0.002852032	0.015193717	LOC10192
TLR9	36.8513	2.103607	0.705186	2.983052	0.002853892	0.015197891	TLR9
CDH16	289.8415	1.168701	0.392032	2.981135	0.00287182	0.015272519	CDH16
SLC38A4	97.46387	1.254798	0.421443	2.977385	0.002907184	0.015422576	SLC38A4
RPS5P2	21.83194	2.945902	0.99031	2.974728	0.002932484	0.015533821	RPS5P2
LOC10537	85.77648	-1.42663	0.480645	-2.96816	0.002995922	0.01583152	LOC10537
DOCK9-D	95.89745	1.211244	0.408817	2.9628	0.003048544	0.016081791	DOCK9-D
GCAT	139.1446	-1.04239	0.351892	-2.96225	0.003054027	0.016099203	GCAT
CBLN3	143.9493	1.002441	0.338953	2.957466	0.003101792	0.016301739	CBLN3
ARMC3	22.56461	-2.59285	0.876793	-2.9572	0.003104454	0.016311057	ARMC3
AGFG2	7890.727	1.716237	0.581098	2.953439	0.003142544	0.01648757	AGFG2
GPR17	21.26805	2.689038	0.910911	2.952032	0.003156907	0.016553454	GPR17
AQP4	18.37911	2.879076	0.976426	2.948587	0.003192299	0.01669132	AQP4
LOC10537	143.979	-1.20056	0.408165	-2.94135	0.003267808	0.017013378	LOC10537
FKBP5	1241.767	-1.91368	0.650858	-2.94024	0.003279538	0.017069605	FKBP5
LOC10798	34.54404	2.108893	0.717938	2.93743	0.003309453	0.017210657	LOC10798
CAPN8	542.7093	3.199	1.089221	2.936962	0.003314442	0.017231719	CAPN8
RAB17	564.8059	4.013032	1.366898	2.935868	0.003326157	0.017273038	RAB17
ANKRD24	81.98055	1.356652	0.462256	2.934854	0.003337046	0.017309146	ANKRD24
LOC10537	39.5271	1.820303	0.62083	2.932046	0.003367369	0.017422931	LOC10537
PRSS35	886.1556	4.684561	1.599878	2.928074	0.003410693	0.017607356	PRSS35
ARHGEF6	72.90594	1.429435	0.489759	2.91865	0.003515502	0.018056185	ARHGEF6
LINC00705	29.00875	2.440112	0.836087	2.918489	0.003517324	0.018056185	LINC00705
SNAP91	65.50156	1.422533	0.487883	2.915725	0.003548632	0.018176213	SNAP91
CYP2C9	50.72916	2.145632	0.737785	2.908207	0.003635082	0.01855166	CYP2C9
KLRC3	110.4255	1.42341	0.490092	2.904377	0.003679852	0.018759269	KLRC3
TRPV4	69.142	1.401342	0.483149	2.900433	0.003726476	0.018960064	TRPV4

LOC10798	82.1309	1.362796	0.469987	2.899649	0.003735811	0.018997017	LOC10798
CZ1P-ASN	96.3959	1.228724	0.424159	2.896844	0.003769372	0.01915175	CZ1P-ASN
EPB41L4A-	157.165	1.08519	0.374607	2.896873	0.003769026	0.01915175	EPB41L4A-
LOC10798	49.18145	1.676808	0.578891	2.896588	0.003772452	0.019162093	LOC10798
LOC10537	48.3284	1.643888	0.56819	2.893202	0.003813356	0.019343082	LOC10537
LRRC3	165.0712	1.021095	0.353096	2.891835	0.003829992	0.01941136	LRRC3
PRKAR2A-	133.008	1.01846	0.35335	2.882298	0.003947861	0.01993184	PRKAR2A-
LINC0232(	18.86459	2.795619	0.970311	2.881157	0.003962178	0.019994793	LINC0232(
LOC11226	65.26221	1.458974	0.50639	2.881128	0.003962549	0.019994793	LOC11226
PLEKHS1	36.31947	-2.09399	0.727356	-2.87891	0.003990568	0.020114039	PLEKHS1
MUC1	184.9485	1.222742	0.42502	2.876902	0.004016004	0.020220021	MUC1
LOC10537	25.02365	2.474479	0.86053	2.87553	0.004033502	0.020291414	LOC10537
LOC10798	32.80227	2.101422	0.730974	2.874825	0.004042517	0.020331189	LOC10798
PCDHGA9	39.35306	1.804966	0.628294	2.872807	0.004068424	0.020444667	PCDHGA9
LRRC19	59.62527	-1.49386	0.520669	-2.86912	0.004116195	0.020656432	LRRC19
MYLK4	127.6253	1.089156	0.379609	2.869156	0.004115688	0.020656432	MYLK4
SLC2A1-A	97.79409	1.16281	0.405355	2.868622	0.00412264	0.020677459	SLC2A1-A
SPOCD1	115.9135	1.086814	0.37912	2.866676	0.004148077	0.020787807	SPOCD1
GAPDHP6!	55.80173	1.520253	0.530851	2.863803	0.004185888	0.020920314	GAPDHP6!
SCX	128.5386	1.018304	0.356067	2.85987	0.004238149	0.021141184	SCX
GRID1	102.4898	1.477902	0.517384	2.85649	0.004283536	0.021321199	GRID1
LOC10537	126.3451	-1.22471	0.428975	-2.85497	0.004304094	0.021400294	LOC10537
LOC10537	18.60873	2.732262	0.957737	2.852831	0.004333162	0.021492391	LOC10537
SMIM38	55.56163	1.636915	0.574221	2.850674	0.004362673	0.021609543	SMIM38
LOC10192	44.84966	1.667541	0.585944	2.845905	0.004428534	0.021888488	LOC10192
SH3BP2	2023.966	1.540683	0.541852	2.843366	0.004463979	0.022057735	SH3BP2
CHRNA9	37.302	2.06351	0.725844	2.84291	0.00447037	0.02208336	CHRNA9
CSPG5	154.2571	1.097327	0.386755	2.837268	0.004550142	0.022411845	CSPG5
SNX18P16	53.67091	-1.53787	0.54204	-2.83719	0.004551276	0.022411845	SNX18P16
FOXH1	27.92346	2.312356	0.81631	2.832696	0.004615732	0.022667139	FOXH1
RPL12P14	169.7295	1.235548	0.437104	2.826672	0.00470345	0.023036228	RPL12P14
CACNB4	99.13715	-1.14739	0.406148	-2.82505	0.004727321	0.023106083	CACNB4
LOC10192	116.2006	1.079772	0.382264	2.824677	0.004732829	0.023106083	LOC10192
NR4A3	82.00552	-1.38004	0.488479	-2.82518	0.004725344	0.023106083	NR4A3
SAP25	63.83712	1.524555	0.539616	2.825258	0.004724257	0.023106083	SAP25
CLIP2	8493.474	1.447371	0.512494	2.824171	0.004740314	0.023113287	CLIP2
LOC10026	160.9887	1.000358	0.354736	2.820006	0.004802277	0.02336429	LOC10026
LOC10537	67.7336	1.352876	0.479841	2.819425	0.004810976	0.023388001	LOC10537
SFTPD	41.33048	1.781196	0.63199	2.818395	0.004826444	0.023425943	SFTPD
KCNQ3	145.8054	1.021849	0.362762	2.816859	0.00484958	0.023507136	KCNQ3
LOC10192	51.48462	-1.52976	0.543957	-2.81229	0.004919089	0.023787486	LOC10192
RNF165	58.22599	1.434913	0.511362	2.806059	0.005015149	0.02417552	RNF165
DDR1	57.4665	1.435617	0.513373	2.796442	0.005166876	0.024789652	DDR1
CBR3	170.1697	-1.0406	0.372665	-2.79231	0.005233239	0.025055615	CBR3
RPL7AP53	21.23828	2.399826	0.860548	2.788717	0.005291727	0.025276265	RPL7AP53
GPR132	54.1049	1.613727	0.578715	2.788463	0.005295877	0.0252895	GPR132
LOC10798	58.84798	1.438255	0.515896	2.787876	0.005305485	0.025315143	LOC10798
LOC10537	130.8647	1.036516	0.371963	2.786612	0.005326215	0.025388107	LOC10537
TYMSOS	77.26642	-1.28866	0.462876	-2.78402	0.005368997	0.025558824	TYMSOS
PTGER2	86.80192	-1.21191	0.435498	-2.78282	0.005388807	0.025626522	PTGER2
LOC10192	33.81231	1.875818	0.675994	2.774902	0.005521838	0.026103493	LOC10192
LBP	99.63182	-1.25901	0.453979	-2.77329	0.005549278	0.026212945	LBP
ST6GALNA	2541.486	1.513157	0.546367	2.769489	0.005614438	0.026473014	ST6GALNA
GPER1	81.65315	1.314851	0.474992	2.768155	0.005637456	0.026561064	GPER1
MYRFL	61.64881	1.476174	0.533408	2.76744	0.005649853	0.026598973	MYRFL
ANKK1	27.20473	2.106089	0.761675	2.765077	0.005690939	0.026744352	ANKK1
LOC10798	84.31841	1.634934	0.591276	2.765094	0.005690633	0.026744352	LOC10798
LOC10798	101.3951	-1.17244	0.424228	-2.7637	0.005714987	0.026836735	LOC10798

LOC10272	92.67997	1.164409	0.421348	2.763532	0.005717948	0.026836899	LOC10272
COL13A1	43.06669	1.680303	0.608415	2.761771	0.005748883	0.026961394	COL13A1
SNORD58/	38.98585	-1.77396	0.643281	-2.75768	0.005821349	0.027245518	SNORD58/
HPDL	53.96165	-1.49552	0.542358	-2.75744	0.005825627	0.027256984	HPDL
ABLIM2	77.74288	1.352353	0.490735	2.755771	0.005855394	0.027355994	ABLIM2
BRINP3	24.7047	-2.42249	0.879867	-2.75324	0.005900867	0.027505389	BRINP3
TGM3	40.37949	-1.94897	0.709498	-2.74697	0.006014931	0.0279094	TGM3
BSPRY	23.54977	2.230088	0.812495	2.744739	0.006055901	0.028064004	BSPRY
KMO	120.4431	1.058033	0.386542	2.737178	0.006196874	0.028594481	KMO
SLC1A2	54.0325	-1.46603	0.535682	-2.73676	0.006204747	0.028623607	SLC1A2
BEST3	55.89627	1.457307	0.532793	2.735222	0.006233828	0.028734783	BEST3
BCL2	92.68325	-1.29764	0.474651	-2.73388	0.006259276	0.028809947	BCL2
TMEM198	56.84885	1.4193	0.519206	2.733596	0.00626469	0.028820404	TMEM198
NUDT9P1	30.33858	1.945151	0.712237	2.731046	0.00631337	0.029015243	NUDT9P1
IL32	108.4173	1.176027	0.430863	2.729467	0.006343676	0.029129573	IL32
LOC10192	117.1342	1.026512	0.376092	2.729419	0.0063446	0.029129573	LOC10192
LINC02432	42.03368	-1.81519	0.665096	-2.72922	0.006348423	0.029139831	LINC02432
LOC10537	77.70355	1.242985	0.45597	2.726021	0.006410299	0.029379738	LOC10537
LOC10537	32.13112	2.09877	0.771214	2.721387	0.006500869	0.029735398	LOC10537
JCHAIN	24.79793	2.322281	0.854406	2.718006	0.006567674	0.02998862	JCHAIN
RTEL1	58.92655	-1.38921	0.511485	-2.71603	0.006606908	0.03012277	RTEL1
LOC10537	69.4811	1.279001	0.470949	2.715795	0.006611681	0.030137039	LOC10537
LOC10798	29.55865	1.968389	0.725903	2.711642	0.006695077	0.03044907	LOC10798
DUOXA1	22.70468	2.264563	0.835411	2.710718	0.00671377	0.030518953	DUOXA1
CD83	83.33965	-1.20481	0.444493	-2.71053	0.006717537	0.03052851	CD83
ARMCX4	117.0706	1.027319	0.37923	2.70896	0.006749447	0.030658911	ARMCX4
LINC01352	41.41153	1.742279	0.643313	2.70829	0.006763083	0.030675485	LINC01352
N4BP3	98.05718	1.100931	0.406497	2.708336	0.006762143	0.030675485	N4BP3
SLCO4A1-	36.18482	2.14315	0.791893	2.706362	0.006802476	0.03080766	SLCO4A1-
GFRA2	28.42678	2.153993	0.796579	2.704054	0.006849921	0.031007221	GFRA2
ADORA2A	57.50272	1.779333	0.658346	2.702731	0.006877233	0.031097988	ADORA2A
HMSD	32.77768	-1.86111	0.688648	-2.70256	0.006880749	0.031100714	HMSD
DAPK1-IT1	45.75844	1.583792	0.586568	2.700097	0.006931918	0.031321045	DAPK1-IT1
C2orf92	67.16465	1.413358	0.523922	2.69765	0.006983089	0.031485698	C2orf92
LOC10537	78.10467	-1.22591	0.454686	-2.69618	0.007014073	0.031609733	LOC10537
TMEM200I	32.28601	1.853608	0.687529	2.696045	0.007016828	0.031609733	TMEM200I
TBC1D3L	70.45014	1.295515	0.48093	2.69377	0.007064887	0.03175304	TBC1D3L
HIC1	105.9775	1.304287	0.485198	2.688154	0.007184825	0.032205295	HIC1
LOC90246	39.92359	1.672049	0.622214	2.687258	0.007204125	0.032278279	LOC90246
LOC10537	32.05572	1.874684	0.70017	2.677468	0.007418102	0.033078407	LOC10537
RPSAP13	22.02752	-2.28558	0.85365	-2.67741	0.007419287	0.033078407	RPSAP13
LOC10798	32.11924	2.180021	0.814855	2.675348	0.007465163	0.033234471	LOC10798
LINC00632	54.61581	-1.57373	0.588736	-2.67306	0.007516228	0.033429354	LINC00632
WNT5A	114.122	-1.17459	0.43949	-2.67263	0.007526017	0.033464776	WNT5A
LOC10013	39.57848	1.697237	0.635743	2.66969	0.007592119	0.033701514	LOC10013
S100A5	149.5407	1.081709	0.405246	2.669265	0.007601741	0.033719741	S100A5
PCDH18	32.53989	-1.83617	0.688891	-2.6654	0.007689567	0.034051677	PCDH18
LOC10050	52.1362	1.436547	0.539257	2.663939	0.007723156	0.034171391	LOC10050
SERPINB7	95.19462	-1.12543	0.422661	-2.66273	0.007751036	0.03424946	SERPINB7
GSTA1	45.83113	-1.87379	0.704991	-2.65789	0.007863238	0.034665338	GSTA1
AANAT	96.81957	1.223288	0.461094	2.653012	0.007977702	0.035036322	AANAT
LOC10537	98.19869	1.100464	0.414805	2.652967	0.007978776	0.035036322	LOC10537
LOC10798	19.60756	2.407195	0.907467	2.652652	0.007986208	0.035060563	LOC10798
LOC72839	109.5659	1.048486	0.395602	2.650357	0.008040681	0.035274379	LOC72839
HPCA	44.01383	1.551553	0.587105	2.642719	0.008224328	0.03598536	HPCA
TRH-GTG1	95.2924	-1.14557	0.434036	-2.63935	0.008306564	0.036275951	TRH-GTG1
MYH15	1043.041	3.404698	1.290066	2.639167	0.008311012	0.036286738	MYH15
PCDHGB7	24.48034	2.114667	0.801357	2.638857	0.008318606	0.036302611	PCDHGB7

WDR93	43.70898	1.560725	0.592228	2.635344	0.008405202	0.036558743	WDR93
CHSY3	50.91367	-1.52832	0.580086	-2.63464	0.008422552	0.036625522	CHSY3
GBX2	56.4644	-1.50751	0.573045	-2.6307	0.008520901	0.036948077	GBX2
CTB-3M24	70.18265	-1.32154	0.502825	-2.62824	0.008582816	0.037172614	CTB-3M24
DRICH1	42.40111	1.582756	0.60299	2.624845	0.008668844	0.037421495	DRICH1
EGOT	28.60185	1.954468	0.745429	2.621938	0.008743137	0.037674825	EGOT
EEF1A1P5	107.8425	1.073474	0.409698	2.620157	0.008788923	0.037841884	EEF1A1P5
PCDHGB2	41.59256	1.6067	0.614088	2.6164	0.008886249	0.03821608	PCDHGB2
WNT5B	66.33848	1.311352	0.50169	2.61387	0.008952301	0.038424977	WNT5B
SH3BP1	85.47606	-1.18184	0.452231	-2.61336	0.008965611	0.038458192	SH3BP1
LINC01252	55.29276	1.411596	0.540469	2.611796	0.009006787	0.038598713	LINC01252
KLRG2	93.14761	-1.21102	0.465792	-2.59991	0.009324695	0.039738278	KLRG2
LOC10028	97.92482	1.068002	0.410865	2.599396	0.009338806	0.039779929	LOC10028
NPHS1	84.81094	1.128752	0.434269	2.599202	0.009344086	0.039793178	NPHS1
CALCRL	81.59089	-1.2105	0.466763	-2.5934	0.009503337	0.040349586	CALCRL
UGT1A6	31.36366	-1.88172	0.726325	-2.59074	0.009576948	0.040596344	UGT1A6
CRYBG2	61.0776	1.400469	0.541308	2.587193	0.009676139	0.040941117	CRYBG2
LOC10537	141.8573	-3.32692	1.286328	-2.58637	0.009699277	0.041010638	LOC10537
LOC10798	23.02583	2.109756	0.817335	2.581263	0.009843965	0.041536232	LOC10798
LOC40049	79.76942	1.393393	0.540318	2.578842	0.00991322	0.041770795	LOC40049
BHLHE40-	95.72403	1.04909	0.406938	2.57801	0.009937115	0.041823445	BHLHE40-
LOC10798	82.72937	-1.12278	0.435953	-2.57547	0.010010388	0.042064269	LOC10798
UPB1	20.62301	2.773342	1.080756	2.566114	0.010284498	0.042999374	UPB1
LOC10537	74.60346	1.275008	0.497088	2.564953	0.010318985	0.043112296	LOC10537
LOC10013	44.36042	1.493265	0.582816	2.562155	0.010402502	0.043364247	LOC10013
LOC10272	27.63598	-1.89544	0.739771	-2.5622	0.010401126	0.043364247	LOC10272
DLX1	56.35451	-1.41261	0.552637	-2.55612	0.010584611	0.043973487	DLX1
LINC02649	68.97139	1.552801	0.607991	2.553988	0.010649692	0.044223826	LINC02649
LOC10537	39.87693	1.614371	0.632751	2.551353	0.010730565	0.044509277	LOC10537
C1orf61	101.4751	-1.0228	0.400996	-2.55066	0.010752026	0.044578134	C1orf61
XKRX	105.3514	-1.36104	0.534825	-2.54484	0.010932889	0.045215568	XKRX
SRL	45.83628	1.479747	0.582109	2.542043	0.011020646	0.045513149	SRL
CTF1	29.47479	1.818566	0.716653	2.537582	0.011162125	0.046008021	CTF1
SLC9A9	49.32109	-1.48112	0.584106	-2.5357	0.011222194	0.046234832	SLC9A9
C5-OT1	55.97266	-1.52972	0.60391	-2.53303	0.011308277	0.046505922	C5-OT1
SLC14A1	53.22446	-1.57035	0.620892	-2.52918	0.011432963	0.04692401	SLC14A1
ACP7	21.64777	2.188056	0.865275	2.528741	0.011447261	0.046951175	ACP7
ABCA6	28.04308	2.011814	0.795825	2.52796	0.011472732	0.046994182	ABCA6
SNHG25	86.6282	-1.0669	0.423083	-2.52174	0.011677698	0.047704307	SNHG25
CCDC74B	91.96702	-1.05508	0.419555	-2.51475	0.011911832	0.048595825	CCDC74B
LOC11009	90.21577	-1.04759	0.416927	-2.51265	0.01198271	0.048818239	LOC11009
ZNF75CP	24.33997	2.086628	0.83047	2.512586	0.011984982	0.048818239	ZNF75CP
LOC10798	48.5124	1.463003	0.582303	2.512442	0.011989868	0.0488273	LOC10798
SLC28A2	42.42702	-1.75096	0.698144	-2.50802	0.012140995	0.04932229	SLC28A2
LOC10012	34.21256	1.671845	0.66713	2.506025	0.012209708	0.049522908	LOC10012
LINC00444	88.04178	1.143573	0.456445	2.505392	0.012231587	0.049580503	LINC00444
PODN	18.99209	2.349647	0.937904	2.505212	0.012237817	0.049583841	PODN
NTF4	103.4438	1.045809	0.417891	2.502587	0.012328947	0.049897956	NTF4
FLT3LG	74.86735	1.192759	0.476748	2.501866	0.012354072	0.049988614	FLT3LG
SEC14L5	20.90745	2.148288	0.858769	2.50159	0.012363696	0.050016521	SEC14L5
LOC10192	61.62479	1.311437	0.524969	2.498121	0.012485357	0.050397541	LOC10192
TBC1D3E	69.73382	1.198977	0.480417	2.495699	0.012570913	0.05061672	TBC1D3E
WDR86	142.0922	1.03874	0.416221	2.495647	0.012572772	0.05061672	WDR86
TNFRSF9	34.24154	1.723922	0.691424	2.493292	0.012656481	0.050875267	TNFRSF9
ADRA1B	1061.76	1.954749	0.784287	2.492391	0.012688614	0.050971213	ADRA1B
PDE2A	52.1904	1.51569	0.609543	2.486601	0.01289701	0.051661599	PDE2A
OPRL1	88.50187	1.097734	0.442295	2.481902	0.013068303	0.052199219	OPRL1
TLR10	18.74878	-2.31947	0.934988	-2.48074	0.013110875	0.052346466	TLR10

LOC10537	28.52385	6.830066	2.756417	2.477879	0.013216606	0.052711238	LOC10537
LOC10537	100.5194	-1.08041	0.436074	-2.47759	0.013227393	0.05274106	LOC10537
LOC10192	97.26905	1.026449	0.4144	2.47695	0.013251061	0.052814202	LOC10192
GRIN2C	69.65809	-1.16646	0.471775	-2.47248	0.013417861	0.053293722	GRIN2C
RPS29P18	54.42978	1.302773	0.527203	2.471105	0.013469636	0.053418393	RPS29P18
ASGR1	65.59038	-1.23411	0.49965	-2.46995	0.013513349	0.053522318	ASGR1
DACT1	45.86575	1.423625	0.576739	2.468406	0.01357164	0.053698746	DACT1
HAO1	22.52659	-2.52751	1.023954	-2.46838	0.013572532	0.053698746	HAO1
WNT10B	90.34968	1.035444	0.41986	2.466164	0.013656868	0.053950954	WNT10B
CNRIP1	47.90537	1.38431	0.561504	2.465361	0.013687514	0.054058635	CNRIP1
LOC10537	80.38954	1.093705	0.443794	2.464444	0.013722612	0.054129064	LOC10537
LOC10798	36.36137	1.824553	0.741373	2.461048	0.013853183	0.05452684	LOC10798
RNF2P1	69.96518	-1.14923	0.46706	-2.46056	0.013872064	0.054589443	RNF2P1
RPL7P26	31.67866	1.777511	0.72281	2.459169	0.013925917	0.054707467	RPL7P26
TAPT1-AS	116.8461	1.00238	0.407573	2.459389	0.013917372	0.054707467	TAPT1-AS
ZNF493	47.09098	1.450257	0.589775	2.458998	0.013932523	0.05472074	ZNF493
LOC10272	35.00723	1.628593	0.662624	2.457794	0.013979352	0.054846904	LOC10272
RNVU1-7	1895.628	1.317339	0.536131	2.457119	0.01400564	0.054914803	RNVU1-7
MGARP	78.39143	1.107296	0.451756	2.451091	0.01424239	0.055664591	MGARP
CD22	20.92628	-2.23219	0.91131	-2.44943	0.014308091	0.05587375	CD22
YES1P1	32.39848	-1.70691	0.697286	-2.44794	0.01436753	0.0560462	YES1P1
ARHGAP2	17.93153	2.41296	0.987898	2.442521	0.014585085	0.056834425	ARHGAP2
PPIAP42	39.6953	-1.52617	0.62672	-2.43517	0.014884625	0.057841905	PPIAP42
DDX58	2172.787	1.347019	0.553997	2.431453	0.015038403	0.058283215	DDX58
TAT	45.72053	-1.43735	0.591154	-2.43143	0.015039497	0.058283215	TAT
SLC25A21	96.20627	-1.066	0.438467	-2.43119	0.015049258	0.058308721	SLC25A21
LOC10798	54.79086	-1.36339	0.561522	-2.42804	0.01518086	0.05875656	LOC10798
LOC10192	49.78409	-1.35598	0.558855	-2.42636	0.015251158	0.058978862	LOC10192
LOC10537	87.55887	-1.03692	0.427439	-2.42588	0.015271254	0.059044129	LOC10537
MTRNR2L	84.75057	-1.04506	0.433173	-2.41258	0.015840276	0.060821087	MTRNR2L
SCAT8	45.08864	-1.38906	0.57613	-2.41102	0.015907892	0.061029609	SCAT8
SNORA37	113.4437	1.011204	0.41947	2.410671	0.015923209	0.061062825	SNORA37
LOC10537	65.81352	1.156822	0.480455	2.407766	0.016050461	0.061435215	LOC10537
IRAK2	2030.624	1.909554	0.793875	2.405359	0.01615658	0.061746477	IRAK2
LOC10192	90.92795	-1.05266	0.437864	-2.40408	0.016213017	0.061902407	LOC10192
LOC10537	64.4124	1.231151	0.512159	2.403845	0.016223651	0.061930117	LOC10537
LOC10537	68.32163	1.148594	0.478313	2.401344	0.016334965	0.062264326	LOC10537
TFF1	138.3899	-1.21129	0.504835	-2.39938	0.016422652	0.062507634	TFF1
TSPAN7	28.01234	-1.7666	0.736509	-2.39862	0.016457017	0.062599464	TSPAN7
B3GLCT	2053.936	1.381138	0.576247	2.396782	0.016539772	0.062810042	B3GLCT
SLC25A39	59.874	1.201798	0.50141	2.396835	0.016537387	0.062810042	SLC25A39
IZUMO4	46.83377	1.38335	0.578313	2.392046	0.016754737	0.063455581	IZUMO4
KLHL35	71.62465	1.101225	0.460387	2.391952	0.016759027	0.063458728	KLHL35
LOC10798	54.51124	-1.47658	0.617601	-2.39084	0.016810015	0.063612398	LOC10798
LOC10537	103.4407	1.079653	0.451673	2.390342	0.016832668	0.063634906	LOC10537
CLDN2	6377.539	-1.73937	0.728187	-2.38863	0.016911341	0.063825548	CLDN2
LOC15843	24.91261	-2.04529	0.856246	-2.38868	0.0169092	0.063825548	LOC15843
LOC10272	80.06728	1.07152	0.448904	2.386968	0.016987973	0.064021658	LOC10272
LOC10537	54.78637	-1.27646	0.534805	-2.38677	0.016996962	0.064042376	LOC10537
DNAJB13	75.65908	1.122519	0.470845	2.384055	0.017123049	0.064385193	DNAJB13
SHBG	41.62065	1.479188	0.622227	2.37725	0.017442242	0.065397712	SHBG
LOC10192	115.535	1.042472	0.438561	2.37703	0.017452666	0.065423421	LOC10192
LOC10537	38.06337	1.581765	0.666898	2.371823	0.017700576	0.066185966	LOC10537
SLC11A1	92.65798	1.011165	0.426347	2.371697	0.017706604	0.066185966	SLC11A1
SNORD28	103.0186	-1.19079	0.502328	-2.37054	0.017762068	0.066341133	SNORD28
DISP2	125.5194	1.184422	0.499898	2.369329	0.017820379	0.06651636	DISP2
PRPH	37.02576	1.544808	0.652482	2.367588	0.017904472	0.066733207	PRPH
STX11	25.16041	1.924415	0.814235	2.363465	0.018104928	0.067318183	STX11

LINC01447	24.37634	-1.96192	0.83032	-2.36284	0.018135259	0.067403632	LINC01447
SINHCAF	51.65198	1.29661	0.549398	2.360056	0.018272167	0.067857478	SINHCAF
NXPE4	19.29624	2.219065	0.942928	2.353376	0.01860379	0.068768786	NXPE4
CNNM1	7563.733	1.193228	0.507112	2.352988	0.018623259	0.068813017	CNNM1
COL9A3	35.38717	-1.53285	0.651502	-2.3528	0.018632839	0.068834547	COL9A3
OLFML2B	118.6973	1.145178	0.487003	2.351483	0.018698747	0.069022422	OLFML2B
LOC10798	83.77382	1.007689	0.428723	2.350443	0.018751078	0.069154199	LOC10798
LINC00964	25.35947	1.812753	0.771417	2.349901	0.018778425	0.069169998	LINC00964
CFHR1	48.51765	-1.49074	0.635783	-2.34472	0.019041145	0.069878487	CFHR1
LOC10536	19.60484	2.121826	0.905185	2.344081	0.019074039	0.069976251	LOC10536
TMEM59L	81.72116	-1.03319	0.441645	-2.33942	0.019313776	0.070666922	TMEM59L
APLN	33.84133	1.678186	0.717785	2.338006	0.019386926	0.07086389	APLN
LOC10537	31.86537	1.628353	0.696574	2.337661	0.019404832	0.070915209	LOC10537
LINC02274	39.56583	1.609475	0.689083	2.335675	0.01950819	0.071250342	LINC02274
SYBU	1749.997	1.317157	0.565238	2.330272	0.019791787	0.072085178	SYBU
A4GNT	58.93177	1.212789	0.521017	2.327735	0.019926154	0.072402044	A4GNT
EIF5AL1	51.85575	-1.27181	0.546928	-2.32537	0.020051958	0.072815881	EIF5AL1
LRRC32	53.08774	1.591572	0.684663	2.324607	0.020093003	0.072936049	LRRC32
LOC28562	54.66592	1.261977	0.544001	2.319805	0.020351419	0.073728172	LOC28562
LHFPL4	69.33293	1.090028	0.4705	2.316742	0.0205178	0.074171009	LHFPL4
SPON1	56.39151	1.206261	0.520713	2.316556	0.020527917	0.074191735	SPON1
SLC38A11	30.80765	-1.74094	0.752987	-2.31204	0.020775472	0.074808	SLC38A11
CHRNB4	42.81717	-1.69325	0.733482	-2.30852	0.020970436	0.075321587	CHRNB4
MIR137HG	70.74178	1.080335	0.468186	2.307491	0.021027452	0.075461851	MIR137HG
SNORD50I	63.1992	-1.14822	0.497746	-2.30684	0.021063883	0.075563025	SNORD50I
ITPR1-DT	21.20876	2.016343	0.87512	2.304076	0.021218387	0.075924239	ITPR1-DT
LINC02777	51.62233	1.252362	0.544007	2.302106	0.021329196	0.076201813	LINC02777
AKR1B1	148806.3	2.310215	1.004802	2.299175	0.021495026	0.076689705	AKR1B1
LOC10537	65.83417	1.119239	0.487175	2.297406	0.021595619	0.076854258	LOC10537
LOC10537	65.65739	1.120463	0.487792	2.297011	0.02161817	0.076919588	LOC10537
GAS6-AS1	77.34752	-1.03479	0.45146	-2.29209	0.021900522	0.077743251	GAS6-AS1
CP	25431.44	1.763875	0.769774	2.29142	0.021939112	0.077813976	CP
ITIH5	238.4458	2.052073	0.896412	2.289206	0.022067373	0.078093721	ITIH5
LOC10272	59.42939	1.196272	0.523011	2.287277	0.022179653	0.078339919	LOC10272
AATBC	41.54393	-1.40736	0.61559	-2.28619	0.022243016	0.078533476	AATBC
DOCK4-AS	54.91689	1.216515	0.532572	2.284224	0.022358372	0.078895202	DOCK4-AS
AP3B2	45.40779	1.324247	0.580233	2.282267	0.022473585	0.07924867	AP3B2
ADGRE2	73.03458	-1.04235	0.45853	-2.27325	0.02301131	0.080810538	ADGRE2
HLF	95.6545	-1.14574	0.505883	-2.26484	0.023522733	0.082354259	HLF
LOC10192	52.62459	1.21376	0.536487	2.262421	0.023671417	0.082732684	LOC10192
RPS3P5	50.46577	1.252986	0.554097	2.261311	0.023739984	0.082940721	RPS3P5
ENHO	27.77746	-1.67177	0.739882	-2.25951	0.023851814	0.083236296	ENHO
GPR27	113.6214	-1.03828	0.459961	-2.25731	0.023988567	0.083633968	GPR27
SPTSSB	60.14182	1.249582	0.55409	2.255197	0.024120977	0.083951986	SPTSSB
LOC10537	80.19888	1.027495	0.455667	2.254924	0.024138095	0.083995624	LOC10537
GPHA2	20.95426	2.085064	0.926168	2.251279	0.024367865	0.084633416	GPHA2
LOC10537	84.2822	1.1309	0.502531	2.25041	0.024422906	0.084745545	LOC10537
NOG	45.50384	1.581514	0.702748	2.250471	0.02441904	0.084745545	NOG
LOC10537	86.78396	1.082819	0.481914	2.246913	0.024645608	0.085308501	LOC10537
KIAA1671-	78.38002	-1.12298	0.500083	-2.24559	0.024730249	0.08553641	KIAA1671-
SCARF2	83.4522	1.048543	0.467059	2.244991	0.024768717	0.085621525	SCARF2
FOXP3	18.27195	-2.23535	0.996773	-2.24259	0.024923575	0.085994774	FOXP3
CALY	32.47674	1.621387	0.72415	2.23902	0.02515462	0.086596479	CALY
SLC30A3	79.05325	-1.01944	0.455375	-2.23868	0.02517703	0.086657363	SLC30A3
LOC10192	45.64981	1.343643	0.600267	2.238409	0.025194407	0.086684639	LOC10192
CEP83-DT	22.95068	-1.98723	0.888811	-2.23583	0.025363062	0.087101533	CEP83-DT
LOC10012	78.22838	1.000117	0.447863	2.233088	0.025543155	0.087507012	LOC10012
LOC10798	36.55078	1.425689	0.638557	2.232673	0.025570528	0.087584429	LOC10798

TEX52	40.81207	1.489067	0.667346	2.231326	0.025659532	0.087790922	TEX52
FAM186B	60.12103	-1.1455	0.515359	-2.22273	0.02623394	0.089289836	FAM186B
CPA4	98.10574	1.105915	0.497734	2.221901	0.02628998	0.08943079	CPA4
LINC0092ɛ	34.95644	1.50948	0.680072	2.219591	0.026446573	0.089830193	LINC0092ɛ
COL28A1	66.35854	-1.25552	0.566146	-2.21766	0.026577795	0.090192398	COL28A1
LOC11226	78.22186	1.013626	0.457808	2.214084	0.026823026	0.090923662	LOC11226
MIR193B	20.44287	1.885261	0.852459	2.211557	0.026997298	0.09141304	MIR193B
C1orf195	76.93237	1.07103	0.48451	2.210542	0.027067556	0.091600204	C1orf195
PPP1R36	51.64091	1.287778	0.582889	2.209304	0.027153495	0.091840202	PPP1R36
HGF	126.9104	-3.0698	1.39064	-2.20747	0.02728111	0.092101992	HGF
LOC10012	71.76687	1.016829	0.461612	2.202776	0.027610512	0.093145486	LOC10012
RPH3A	21.70049	-2.1349	0.97016	-2.20057	0.027766545	0.093585809	RPH3A
RUNX2	65.82723	1.095203	0.498693	2.196148	0.028081379	0.09447334	RUNX2
EPHB6	34.66987	1.540349	0.702005	2.194214	0.02822004	0.094842338	EPHB6
LDHAP7	34.0003	1.510413	0.690214	2.188327	0.028645801	0.095792395	LDHAP7
SMIM10L2	25.8873	1.711659	0.782157	2.188382	0.028641795	0.095792395	SMIM10L2
SNORD12ʻ	23.25582	1.743111	0.797412	2.185962	0.028818415	0.096194025	SNORD12ʻ
LOC10798	22.33264	2.119103	0.97142	2.181448	0.029150291	0.096984334	LOC10798
LOC10537	26.72794	1.687539	0.773733	2.181034	0.029180884	0.097068512	LOC10537
LOC10537	57.19015	1.134629	0.520651	2.179251	0.029313052	0.097362871	LOC10537
VTRNA1-2	32.37173	-1.48069	0.680664	-2.17537	0.029602718	0.098067085	VTRNA1-2
BMP7	38.14691	-1.36151	0.626433	-2.17344	0.029747499	0.098391264	BMP7
VWA2	88.33199	1.001325	0.460858	2.172739	0.029799947	0.098538063	VWA2
PRO1804	26.30476	1.671153	0.769989	2.17036	0.029979611	0.099060716	PRO1804
LOC61320	54.1856	1.155243	0.533231	2.166497	0.030273198	0.099868885	LOC61320
MSANTD1	35.13404	1.435929	0.663418	2.16444	0.030430575	0.100347399	MSANTD1
KLB	918.9586	2.655727	1.22972	2.159619	0.030802166	0.101285026	KLB
LOC10537	25.68045	-1.71648	0.794909	-2.15934	0.030823519	0.101301735	LOC10537
CNR1	95.43504	1.053234	0.488011	2.158216	0.030911016	0.101514628	CNR1
LOC10272	60.49634	1.111117	0.514834	2.158207	0.030911754	0.101514628	LOC10272
LOC10537	75.24577	-1.0482	0.486001	-2.15678	0.031022653	0.101810254	LOC10537
LINC0066ɜ	39.86395	1.354082	0.627961	2.156316	0.031059031	0.101893176	LINC0066ɜ
LOC10537	21.62838	1.907088	0.884451	2.156239	0.031064982	0.101894475	LOC10537
LOC10537	18.42531	-1.95603	0.907614	-2.15514	0.031151029	0.102121925	LOC10537
LOC10537	27.73339	1.569133	0.729084	2.152198	0.031381781	0.102731506	LOC10537
TMEM171	92.45859	3.050302	1.418173	2.150868	0.031486614	0.102982787	TMEM171
NXNL2	50.20446	1.168076	0.543798	2.147998	0.031713928	0.103523201	NXNL2
RPS26P37	25.93569	1.645004	0.766061	2.147352	0.031765237	0.103653793	RPS26P37
APOD	39.74661	-1.32162	0.615573	-2.14697	0.031795818	0.103735127	APOD
PMCH	63.43117	-1.09518	0.510195	-2.14658	0.031826398	0.103779517	PMCH
GUCA1B	33.38369	-1.74444	0.813564	-2.1442	0.032016814	0.104215157	GUCA1B
TRIB2	62.17502	1.065023	0.496796	2.143786	0.032050031	0.104304769	TRIB2
LOC10537	21.71262	1.798967	0.839499	2.142906	0.032120681	0.104460555	LOC10537
ACTR3-AS	41.63448	1.28461	0.599672	2.142189	0.032178292	0.104610817	ACTR3-AS
TBX4	49.89454	-1.19538	0.558234	-2.14136	0.03224538	0.104754651	TBX4
GOT2P4	44.66608	1.245906	0.581914	2.14105	0.032270032	0.104797616	GOT2P4
LOC10192	20.67911	1.812906	0.847487	2.139155	0.03242315	0.10516453	LOC10192
LOC10798	37.40548	1.354865	0.635788	2.131	0.033089166	0.10688735	LOC10798
TRN-GTT6	65.1449	-1.03786	0.487721	-2.12797	0.03333919	0.107508338	TRN-GTT6
LOC10050	44.85544	1.229743	0.578022	2.127503	0.033378307	0.107615558	LOC10050
CAVIN4	87.11035	1.035354	0.486861	2.126592	0.033453982	0.107798674	CAVIN4
PGAM1P5	72.43198	1.343671	0.632496	2.124393	0.033637265	0.108241179	PGAM1P5
FGL2	44.6516	1.234115	0.581204	2.123375	0.033722442	0.108382169	FGL2
MICOS10F	66.47	1.013116	0.478107	2.119015	0.034089192	0.109350114	MICOS10P
LOC10028	19.99264	1.872636	0.884986	2.116008	0.03434413	0.109994769	LOC10028
PRSS37	37.71692	1.335432	0.631224	2.115624	0.034376803	0.110060974	PRSS37
C11orf91	62.28697	1.047217	0.495091	2.115199	0.034413014	0.110121253	C11orf91
LOC10155	31.06176	-1.55061	0.733643	-2.11358	0.034551499	0.110485284	LOC10155

OGN	38.66389	-1.45896	0.69081	-2.11196	0.034689962	0.110824151	OGN
SMIM1	63.2315	1.05773	0.502615	2.104452	0.035339031	0.112418062	SMIM1
LOC10537	26.86502	1.561749	0.742752	2.102651	0.035496314	0.11273998	LOC10537
AGGF1P3	19.70472	1.819748	0.865846	2.101698	0.035579739	0.112848678	AGGF1P3
LOC10537	58.70759	1.153707	0.549191	2.10074	0.035663806	0.113017542	LOC10537
AKR1B1P2	27.09655	1.912196	0.911748	2.097285	0.035968326	0.113805594	AKR1B1P2
HMGA2-A	51.8523	-1.13711	0.542256	-2.09699	0.035994569	0.113868986	HMGA2-A
MAB21L1	43.61431	1.23605	0.590269	2.094044	0.036256059	0.114495227	MAB21L1
LOC10537	25.98408	1.580409	0.756052	2.090346	0.036586752	0.115285037	LOC10537
JMJD1C-A	50.48065	1.164116	0.558135	2.085723	0.037003703	0.116219507	JMJD1C-A
HSD11B2	41.72855	-1.26265	0.60542	-2.08558	0.03701646	0.116239669	HSD11B2
SLC17A4	68.53122	-1.30344	0.625175	-2.08492	0.037076654	0.116388839	SLC17A4
CSPG4P10	34.42587	1.438383	0.690096	2.084322	0.037130893	0.1164597	CSPG4P10
LOC10798	44.30647	1.240371	0.595718	2.082145	0.037329195	0.116981394	LOC10798
TRS-GCT6	73.60569	-1.0129	0.486566	-2.08173	0.037367254	0.117060662	TRS-GCT6
KRT18P62	24.9402	1.617473	0.777248	2.081027	0.037431424	0.117214395	KRT18P62
SPATA32	59.92345	-1.15203	0.553764	-2.08036	0.03749252	0.117372898	SPATA32
LOC10798	24.49943	1.625024	0.781568	2.079185	0.037600393	0.117611028	LOC10798
NID2	705.8165	1.42205	0.683947	2.079182	0.037600647	0.117611028	NID2
LOC10028	19.58923	1.798766	0.86547	2.078368	0.037675468	0.117824966	LOC10028
TRT-TGT4	20.22751	-1.91796	0.923392	-2.07708	0.037793997	0.118115095	TRT-TGT4
RASSF10-I	64.76834	-1.03571	0.499066	-2.07529	0.03795949	0.118531771	RASSF10-I
LOC10798	41.49127	1.258447	0.607112	2.072841	0.038187083	0.119140581	LOC10798
LOC10106	31.15786	-1.42711	0.689704	-2.06916	0.038530668	0.119947302	LOC10106
LOC10798	23.82542	-1.72624	0.834963	-2.06744	0.038692321	0.120328003	LOC10798
CBLN2	44.3182	-1.41166	0.684489	-2.06236	0.039173149	0.121452654	CBLN2
LOC10798	33.38276	1.619857	0.785416	2.062419	0.039167897	0.121452654	LOC10798
HPCAL4	37.20484	1.430216	0.693509	2.062289	0.039180224	0.121454059	HPCAL4
CCNP	35.7024	1.31998	0.640348	2.061346	0.039270001	0.121670669	CCNP
LOC11226	37.28976	-1.51263	0.734188	-2.06028	0.039371903	0.121924985	LOC11226
LINC0114(	35.6479	-1.36927	0.664647	-2.06014	0.03938479	0.121943926	LINC0114(
EGLN3-AS	30.59461	1.569608	0.762422	2.058715	0.039521589	0.12224436	EGLN3-AS
SNORD18(	44.78623	-1.43934	0.699144	-2.05871	0.039521811	0.12224436	SNORD18(
SASH3	21.68581	1.697998	0.825968	2.055766	0.039805048	0.122892475	SASH3
LOC10050	22.5093	-1.67114	0.812981	-2.05557	0.039823701	0.122929373	LOC10050
LINC0142	58.22311	-1.0461	0.509995	-2.0512	0.040247865	0.123925858	LINC0142
GPCPD1	6791.865	-1.06351	0.519061	-2.04892	0.040470078	0.124455308	GPCPD1
TRQ-CTG2	38.57362	-1.27554	0.622788	-2.04811	0.040549023	0.12462302	TRQ-CTG2
LOC10798	82.78893	1.081056	0.528511	2.045477	0.040807881	0.125280497	LOC10798
C1QTNF1-	33.06566	1.369509	0.669649	2.045115	0.040843559	0.125360201	C1QTNF1-
SLC2A13	4505.29	1.192076	0.583318	2.04361	0.040992059	0.125659507	SLC2A13
ST8SIA1	63.21119	1.007315	0.493462	2.041324	0.041218668	0.126257985	ST8SIA1
APOH	2084.953	-1.07851	0.528463	-2.04084	0.041266808	0.126330049	APOH
MIR6748	32.18472	1.378495	0.675429	2.040916	0.041259198	0.126330049	MIR6748
PIK3R3	57.69035	-1.11285	0.545639	-2.03954	0.041396556	0.126585118	PIK3R3
AGR2	40284.92	-1.44113	0.709025	-2.03256	0.042097379	0.128264139	AGR2
SNORD114	56.59309	1.060547	0.522422	2.030058	0.042350597	0.128967481	SNORD114
VTRNA2-1	37.12398	-1.32221	0.651358	-2.02993	0.04236378	0.128968705	VTRNA2-1
HMGN2P3	21.27584	-1.68903	0.8322	-2.0296	0.04239712	0.129048783	HMGN2P3
HM13-AS1	29.38484	-1.46954	0.725165	-2.0265	0.042713857	0.12988391	HM13-AS1
PRDX2	52.6927	1.153604	0.569327	2.026259	0.042738264	0.12991469	PRDX2
TTR	22.91421	1.85197	0.914228	2.025721	0.042793335	0.130060539	TTR
ERVMER34	57.95982	-1.04099	0.514372	-2.0238	0.042990807	0.13058433	ERVMER34
SH2D2A	51.23448	-1.13482	0.560896	-2.02323	0.043049068	0.130686197	SH2D2A
LOC10192	34.10134	1.362702	0.673701	2.022711	0.043102934	0.130763151	LOC10192
PRKN	50.30327	1.165175	0.576624	2.020685	0.043312402	0.131268354	PRKN
KIAA0754	3910.275	1.025973	0.509387	2.01413	0.043995839	0.132856713	KIAA0754
CDH26	48.18944	-1.22552	0.609863	-2.00949	0.044484792	0.133964726	CDH26

BMERB1	72.89754	1.016183	0.50575	2.00926	0.044509567	0.133966932	BMERB1
LOC10065	55.14426	1.050731	0.523651	2.00655	0.044797576	0.134657019	LOC10065
LOC10537	50.43855	1.162613	0.579484	2.00629	0.04482536	0.134718459	LOC10537
LDHAP3	23.94251	1.581456	0.789235	2.003782	0.045093385	0.135390876	LDHAP3
GNG4	44.84335	1.157732	0.578227	2.002211	0.045262095	0.135696552	GNG4
PTH1R	30.79001	1.382268	0.691585	1.998697	0.045641163	0.1364993	PTH1R
PTCSC2	35.62317	1.376271	0.689136	1.997095	0.045814868	0.136929494	PTCSC2
LOC10537	23.8972	1.578204	0.790456	1.996574	0.045871519	0.137024249	LOC10537
LOC10537	53.50543	1.046626	0.525109	1.993158	0.046244098	0.137808163	LOC10537
S1PR4	36.25887	1.262157	0.633361	1.992793	0.046284116	0.137905012	S1PR4
MIR365A	30.56919	1.412101	0.709121	1.991341	0.04644343	0.138236339	MIR365A
CCL26	251.0407	2.413708	1.212815	1.99017	0.046572234	0.138510212	CCL26
SNRPCP3	19.677	1.781857	0.895383	1.99005	0.046585405	0.138510212	SNRPCP3
CYTIP	45.40482	-1.22079	0.613878	-1.98865	0.046739355	0.138811913	CYTIP
PSD	45.70006	1.157581	0.582635	1.986804	0.04694413	0.139260751	PSD
LOC10537	18.8634	1.813522	0.913242	1.985805	0.047054937	0.139476672	LOC10537
LOC38878	27.03052	-1.50855	0.760051	-1.98481	0.047165999	0.139738128	LOC38878
KLHDC8A	28.65733	1.509674	0.761437	1.982665	0.047404881	0.140309881	KLHDC8A
LOC10537	27.70725	1.525487	0.769659	1.982029	0.047476003	0.140452732	LOC10537
LOC11226	60.24421	-1.07957	0.544681	-1.98203	0.047476117	0.140452732	LOC11226
CREB3L1	177.2849	-2.54495	1.285497	-1.97974	0.047732484	0.141137841	CREB3L1
MYB	51.70849	-1.15795	0.585316	-1.97833	0.047891629	0.141431052	MYB
SLCO2B1	49.57416	-1.12356	0.568046	-1.97794	0.047935505	0.141515054	SLCO2B1
CDX2	50.64522	-1.14895	0.581507	-1.97582	0.048175283	0.142017203	CDX2
PIWIL4	52.62652	1.096881	0.555485	1.974634	0.048309641	0.142344649	PIWIL4
LOC10536	43.36938	1.363386	0.690538	1.974382	0.048338334	0.142383448	LOC10536
LOC10192	53.42321	-1.05177	0.53299	-1.97334	0.048456685	0.142640429	LOC10192
LINC0048	50.56172	-1.22376	0.620992	-1.97066	0.048762676	0.143265258	LINC0048
LOC10798	23.94731	-1.5609	0.792304	-1.97007	0.04882995	0.143394004	LOC10798
COL14A1	22.04449	1.745833	0.886514	1.969324	0.048915925	0.143600495	COL14A1
SNORD42	32.81294	-1.35046	0.685842	-1.96906	0.048946535	0.143621396	SNORD42
ANGPTL2	48.96148	1.212897	0.61612	1.968605	0.048998482	0.143727835	ANGPTL2
LOC10537	19.67297	1.929516	0.980302	1.968288	0.049034948	0.143788812	LOC10537
DRC7	38.66426	-1.24919	0.634698	-1.96817	0.049048723	0.143806215	DRC7
NPEPPSP1	53.85934	1.030384	0.524158	1.965788	0.049323157	0.144338294	NPEPPSP1
SNORD10	48.39164	-1.11902	0.569881	-1.96361	0.049575577	0.144956994	SNORD10
ABCC12	9.901408	-6.72323	1.863706	-3.60745	0.000309221	NA	ABCC12
ACADL	14.22164	-2.90121	1.198909	-2.41987	0.015525903	NA	ACADL
ACSL6	3.809203	-5.34652	2.399112	-2.22854	0.025844536	NA	ACSL6
ACTL8	12.23152	-3.88636	1.312534	-2.96096	0.003066852	NA	ACTL8
ADGRA1	8.425318	2.962884	1.46685	2.019895	0.043394281	NA	ADGRA1
ADH4	17.04412	-4.46738	1.243739	-3.59189	0.000328283	NA	ADH4
ADRA2A	12.50149	-2.35952	1.135706	-2.07758	0.037748327	NA	ADRA2A
AGAP10P	16.66702	-2.22865	1.024115	-2.17617	0.029542281	NA	AGAP10P
AKR1B1P3	16.14517	2.137393	1.088239	1.964084	0.049520368	NA	AKR1B1P3
ANKRD45	9.559237	-2.75612	1.3331	-2.06746	0.038691297	NA	ANKRD45
ARL14EPL	9.101711	5.196002	1.916274	2.711513	0.00669769	NA	ARL14EPL
ARMCX2	10.13593	2.598138	1.258321	2.064765	0.038945237	NA	ARMCX2
ATP1A2	11.77521	2.818604	1.214958	2.31992	0.020345231	NA	ATP1A2
ATP2B2	13.40705	3.381018	1.382437	2.445694	0.014457367	NA	ATP2B2
B3GNT6	10.00638	-3.67112	1.4636	-2.50828	0.012132076	NA	B3GNT6
BFAR	13.48093	2.169674	1.063986	2.039195	0.041430565	NA	BFAR
BMPR1AP	3.587936	5.334164	2.499326	2.13424	0.0328231	NA	BMPR1AP
BNIP3P1	13.95431	2.595538	1.097712	2.364499	0.018054473	NA	BNIP3P1
C5orf46	3.20562	5.160429	2.564344	2.012378	0.04418007	NA	C5orf46
C9orf135	6.416195	-6.09891	2.072676	-2.94253	0.003255428	NA	C9orf135
CALCR	15.23829	3.28322	1.167534	2.812098	0.004921945	NA	CALCR
CASC19	13.29502	-2.67588	1.117716	-2.39406	0.016662899	NA	CASC19

CCDC89	15.8522	2.019094	1.0239	1.971965	0.048613627	NA	CCDC89
CD177	5.3427	5.897526	2.201383	2.679009	0.007384034	NA	CD177
CD200R1L	9.366128	-2.64462	1.311795	-2.01603	0.043796891	NA	CD200R1L
CD34	9.911328	2.871568	1.34697	2.131872	0.033017384	NA	CD34
CDH22	10.57075	2.641156	1.235373	2.137943	0.032521382	NA	CDH22
CDSN	7.796483	3.95005	1.762918	2.240632	0.025049903	NA	CDSN
CHRDL1	3.147388	-5.06948	2.540605	-1.99538	0.046001202	NA	CHRDL1
CHRFAM7	8.517536	2.962925	1.428064	2.074784	0.038006583	NA	CHRFAM7
CLEC19A	12.87035	2.48795	1.11347	2.234412	0.025456008	NA	CLEC19A
CLLU1-AS	3.296234	5.198643	2.627707	1.978396	0.047884094	NA	CLLU1-AS
CPS1-IT1	17.07238	-2.46437	1.122139	-2.19614	0.028081869	NA	CPS1-IT1
CST8	9.410186	6.724148	1.932344	3.479788	0.00050181	NA	CST8
CTD-2201	16.06209	2.025288	0.964175	2.100541	0.035681284	NA	CTD-2201
CXCR3	13.06265	2.552478	1.129606	2.259618	0.023844994	NA	CXCR3
CYP2E1	15.02216	2.571274	1.064728	2.414959	0.015736963	NA	CYP2E1
DBF4P1	5.687063	-5.92093	2.137648	-2.76983	0.005608528	NA	DBF4P1
DPPA2	4.498069	-5.58687	2.290651	-2.43899	0.014728442	NA	DPPA2
F13A1	6.453014	4.685545	2.074103	2.259071	0.023878996	NA	F13A1
FANCD2O	10.04726	-3.67396	1.459783	-2.51678	0.011843181	NA	FANCD2O
FAR2P1	3.174439	-5.08095	2.534957	-2.00435	0.045032101	NA	FAR2P1
FGF14	12.55501	3.234553	1.185058	2.729448	0.006344045	NA	FGF14
FLJ39095	13.61468	2.21665	1.054496	2.102093	0.035545087	NA	FLJ39095
FMC1-LU(	12.24039	-2.3469	1.125175	-2.08581	0.036995708	NA	FMC1-LU(
FREM3	3.024392	5.082381	2.577758	1.971629	0.048651989	NA	FREM3
GAPDHP5	17.22868	1.975945	0.996135	1.983612	0.047299071	NA	GAPDHP5
GIPC3	6.531992	4.673851	2.009752	2.325586	0.020040627	NA	GIPC3
GPRC6A	8.094177	-3.83133	1.678737	-2.28227	0.022473371	NA	GPRC6A
GPS2P2	17.3287	3.211022	1.023284	3.137957	0.001701301	NA	GPS2P2
HAAO	17.26655	2.152332	0.939493	2.290951	0.021966247	NA	HAAO
HAND2-A	8.34839	4.01777	1.583475	2.537311	0.011170753	NA	HAND2-A
HMGCS2	3.37701	-5.17145	2.482629	-2.08305	0.037246418	NA	HMGCS2
HMGN2P4	15.54121	2.398467	1.083998	2.212612	0.026924435	NA	HMGN2P4
HSD17B2	15.75356	3.069861	1.058428	2.900398	0.003726897	NA	HSD17B2
IAPP	7.875015	2.85691	1.449518	1.970939	0.048730908	NA	IAPP
IL20RA	8.9159	-3.04542	1.465233	-2.07846	0.037667251	NA	IL20RA
KASH5	3.484082	5.280342	2.502257	2.110232	0.034838384	NA	KASH5
KCNJ15	8.183235	-3.35924	1.592347	-2.10961	0.034891577	NA	KCNJ15
KCNU1	4.362815	-5.5443	2.605807	-2.12767	0.033364468	NA	KCNU1
KLRC1	13.46379	2.59625	1.135795	2.285844	0.022263358	NA	KLRC1
KRT223P	3.20562	5.160429	2.564344	2.012378	0.04418007	NA	KRT223P
L3MBTL2-	16.80887	-2.28706	0.978224	-2.33797	0.019388883	NA	L3MBTL2-
LINC0032	9.306332	6.704178	1.889117	3.548843	0.000386928	NA	LINC0032
LINC0033	3.115006	5.121843	2.557704	2.002515	0.045229327	NA	LINC0033
LINC0036	4.176926	5.540924	2.645909	2.094148	0.03624684	NA	LINC0036
LINC0058	13.28578	-3.04053	1.259639	-2.41381	0.015786736	NA	LINC0058
LINC0068	10.5041	2.895166	1.320994	2.191657	0.028404297	NA	LINC0068
LINC0083	14.48836	4.81016	1.551372	3.100583	0.001931398	NA	LINC0083
LINC0095	3.349959	-5.16066	2.494203	-2.06906	0.038540167	NA	LINC0095
LINC0111	9.703941	5.290361	1.911374	2.767831	0.005643071	NA	LINC0111
LINC0113	3.809203	-5.34652	2.399112	-2.22854	0.025844536	NA	LINC0113
LINC0141	3.255591	-5.11497	2.551857	-2.00441	0.045026021	NA	LINC0141
LINC0162	3.484082	5.280342	2.502257	2.110232	0.034838384	NA	LINC0162
LINC0191	11.00399	-5.38104	1.78332	-3.01743	0.002549299	NA	LINC0191
LINC0192	7.882144	-3.92161	1.669455	-2.34904	0.018822005	NA	LINC0192
LINC0197	4.349599	-5.5364	2.291832	-2.41571	0.015704516	NA	LINC0197
LINC0209	11.95117	3.172535	1.312733	2.416741	0.015660174	NA	LINC0209
LINC0224	16.64614	2.920003	1.003089	2.911012	0.003602604	NA	LINC0224
LINC0239	4.147028	-5.46663	2.326302	-2.34992	0.018777212	NA	LINC0239

LINC0247	4.176926	5.540924	2.645909	2.094148	0.03624684	NA	LINC02474
LINC0256	12.38887	2.213843	1.10362	2.005983	0.044858015	NA	LINC0256
LINC0269	8.932365	4.082589	1.593451	2.562106	0.01040397	NA	LINC0269
LINC0277	3.762544	5.39106	2.448234	2.20202	0.027663926	NA	LINC02771
LINC0286	15.129	2.321468	1.178936	1.969121	0.048939253	NA	LINC02861
LMO2	10.86814	-2.71436	1.332712	-2.03672	0.041678147	NA	LMO2
LOC10012	6.337524	4.620082	2.179684	2.119611	0.034038883	NA	LOC10012
LOC10013	3.963632	5.478375	2.670788	2.051221	0.040245447	NA	LOC10013
LOC10042	6.396211	-3.60654	1.793419	-2.01099	0.044326962	NA	LOC10042
LOC10042	6.132333	3.584667	1.799058	1.992525	0.046313492	NA	LOC10042
LOC10050	4.695163	5.713312	2.251919	2.537086	0.011177942	NA	LOC10050
LOC10050	6.964631	4.799393	2.028426	2.366068	0.017978159	NA	LOC10050
LOC10192	8.574479	2.996159	1.405747	2.131365	0.03305911	NA	LOC10192
LOC10192	16.33593	3.125313	1.174168	2.661726	0.007774111	NA	LOC10192
LOC10192	7.531707	6.395569	1.986524	3.219477	0.001284245	NA	LOC10192
LOC10192	3.917406	-5.38409	2.369676	-2.27208	0.023081862	NA	LOC10192
LOC10192	4.092926	-5.44903	2.336024	-2.33261	0.019668705	NA	LOC10192
LOC10192	11.83158	2.874732	1.225913	2.344972	0.01902851	NA	LOC10192
LOC10192	10.84484	5.429165	1.880664	2.886833	0.003891404	NA	LOC10192
LOC10192	14.20083	2.244642	1.07323	2.091483	0.036484792	NA	LOC10192
LOC10272	3.458162	-5.20332	2.474579	-2.10271	0.035491208	NA	LOC10272
LOC10272	9.460433	2.749652	1.307886	2.102364	0.035521385	NA	LOC10272
LOC10536	6.722927	3.68556	1.687376	2.184196	0.028947866	NA	LOC10536
LOC10537	12.07275	-3.43972	1.245915	-2.7608	0.005766057	NA	LOC10537
LOC10537	6.793556	6.250091	2.029398	3.079776	0.002071563	NA	LOC10537
LOC10537	3.20562	5.160429	2.564344	2.012378	0.04418007	NA	LOC10537
LOC10537	13.04965	2.309013	1.103244	2.092931	0.036355307	NA	LOC10537
LOC10537	8.146857	2.908427	1.4792	1.966217	0.04927359	NA	LOC10537
LOC10537	10.6431	2.631016	1.249924	2.10494	0.035296528	NA	LOC10537
LOC10537	3.024392	5.082381	2.577758	1.971629	0.048651989	NA	LOC10537
LOC10537	7.330173	3.857516	1.847751	2.087682	0.036826533	NA	LOC10537
LOC10537	12.70429	2.698832	1.148763	2.349338	0.018806814	NA	LOC10537
LOC10537	6.094092	6.092356	2.085079	2.921883	0.003479228	NA	LOC10537
LOC10537	10.89496	3.320959	1.35889	2.443877	0.014530398	NA	LOC10537
LOC10537	7.833241	3.302244	1.500748	2.200399	0.027778597	NA	LOC10537
LOC10537	6.320626	6.141304	2.086449	2.943424	0.003246032	NA	LOC10537
LOC10537	4.883011	5.771433	2.218263	2.60178	0.00927412	NA	LOC10537
LOC10537	10.81598	3.333905	1.425458	2.338831	0.019344198	NA	LOC10537
LOC10537	6.068769	3.523375	1.734213	2.031685	0.042185505	NA	LOC10537
LOC10537	9.594625	3.617102	1.424627	2.538981	0.011117591	NA	LOC10537
LOC10537	4.889994	-5.70416	2.216386	-2.57363	0.010063712	NA	LOC10537
LOC10537	9.510397	-3.14618	1.437803	-2.18818	0.028656189	NA	LOC10537
LOC10537	16.45654	-2.31262	0.977776	-2.36518	0.018021405	NA	LOC10537
LOC10537	4.768575	-5.6655	2.279525	-2.48539	0.012941058	NA	LOC10537
LOC10537	5.015399	4.308818	2.166855	1.988513	0.046755016	NA	LOC10537
LOC10537	10.41941	2.658937	1.312012	2.026611	0.042702216	NA	LOC10537
LOC10537	11.91819	3.464442	1.313576	2.637414	0.008354086	NA	LOC10537
LOC10537	5.763703	6.008717	2.131133	2.819494	0.004809941	NA	LOC10537
LOC10537	5.073946	4.337088	2.164697	2.003554	0.045117851	NA	LOC10537
LOC10537	5.877834	4.510538	2.09226	2.15582	0.031097687	NA	LOC10537
LOC10537	10.50694	2.966517	1.447462	2.049461	0.040417078	NA	LOC10537
LOC10537	9.760684	3.175325	1.335162	2.378232	0.017395859	NA	LOC10537
LOC10798	3.348161	5.226798	2.493372	2.096277	0.03605764	NA	LOC10798
LOC10798	12.46897	2.706809	1.188271	2.27794	0.022730167	NA	LOC10798
LOC10798	17.19756	-1.92977	0.930856	-2.07312	0.038161383	NA	LOC10798
LOC10798	4.893527	-4.21237	2.146652	-1.9623	0.049727724	NA	LOC10798
LOC10798	8.314274	2.977491	1.505062	1.978317	0.047892911	NA	LOC10798
LOC10798	11.54237	4.478998	1.487992	3.010095	0.002611656	NA	LOC10798

LOC10798	13.43479	3.629153	1.309131	2.772184		0.005568156	NA	LOC10798
LOC10798	8.962936	-4.11221	1.600293	-2.56966		0.010179832	NA	LOC10798
LOC10798	13.50317	2.765518	1.138328	2.429455		0.015121534	NA	LOC10798
LOC10798	5.600649	-4.35901	2.064475	-2.11144		0.034734869	NA	LOC10798
LOC10798	10.97268	3.070068	1.365766	2.247873		0.024584258	NA	LOC10798
LOC10798	6.956591	-6.21465	2.019707	-3.07701		0.002090913	NA	LOC10798
LOC10798	11.64829	-2.65998	1.242105	-2.14151		0.032232703	NA	LOC10798
LOC10798	13.85971	2.0619	1.048999	1.965589		0.049346117	NA	LOC10798
LOC10798	10.85558	3.045965	1.432331	2.126578		0.033455127	NA	LOC10798
LOC10798	14.80577	2.742547	1.05029	2.611229		0.009021756	NA	LOC10798
LOC10798	16.18042	2.083257	1.000245	2.082747		0.037274267	NA	LOC10798
LOC10798	13.85137	2.423043	1.056397	2.293685		0.021808588	NA	LOC10798
LOC10798	6.896339	-3.71641	1.748086	-2.12599		0.033504184	NA	LOC10798
LOC10798	17.80124	1.840031	0.908025	2.02641		0.042722789	NA	LOC10798
LOC11226	3.809203	-5.34652	2.399112	-2.22854		0.025844536	NA	LOC11226
LOC11226	8.408867	5.08014	1.913703	2.654612		0.007939974	NA	LOC11226
LOC11226	8.712575	2.959922	1.442852	2.051438		0.040224294	NA	LOC11226
LOC11257	3.905085	5.447677	2.378931	2.289969		0.022023139	NA	LOC11257
LOC39031	14.88122	2.547502	1.037861	2.45457		0.014105323	NA	LOC39031
LOC39253	7.152479	4.839631	1.993603	2.42758		0.01519995	NA	LOC39253
LOC64513	10.41657	2.59825	1.240524	2.094477		0.036217512	NA	LOC64513
LOC64601	7.480535	-3.2203	1.599338	-2.01352		0.044059622	NA	LOC64601
LRRC10B	10.98147	3.023844	1.270871	2.379348		0.017343282	NA	LRRC10B
LUNAR1	11.15757	-2.61819	1.219345	-2.14721		0.031776194	NA	LUNAR1
MAGEB6	7.311793	6.359389	2.020774	3.147007		0.00164951	NA	MAGEB6
MC4R	6.168096	-4.50534	2.020601	-2.22971		0.02576702	NA	MC4R
MCEMP1	3.660733	-5.28664	2.419141	-2.18534		0.028864017	NA	MCEMP1
MDFI	10.84395	3.01809	1.287425	2.344285		0.019063616	NA	MDFI
MEP1B	4.376649	-5.54472	2.287506	-2.42391		0.015354261	NA	MEP1B
MGC3280	15.69599	-2.1903	1.010017	-2.16857		0.030115165	NA	MGC3280
MIR1-1HG	9.668787	6.755344	1.886799	3.580319		0.000343175	NA	MIR1-1HG
MIR30C2	15.33031	2.11474	0.998335	2.118266		0.034152535	NA	MIR30C2
MIR3143	12.38466	-2.67656	1.267257	-2.11209		0.034678568	NA	MIR3143
MIR3680-:	3.730477	5.390985	2.717892	1.983517		0.047309725	NA	MIR3680-:
MIR3960	3.529389	5.297968	2.523241	2.099668		0.03575803	NA	MIR3960
MMP10	13.69089	3.334316	1.217258	2.739203		0.006158841	NA	MMP10
MORN3	16.13962	-1.99604	0.976459	-2.04416		0.04093728	NA	MORN3
MPPED1	17.66777	3.025948	1.056703	2.863573		0.004188921	NA	MPPED1
MRO	9.671786	-6.68925	1.87223	-3.57288		0.000353079	NA	MRO
NAV2-AS	9.319697	4.182965	1.547452	2.70313		0.00686899	NA	NAV2-AS
NDUFAF2	3.37701	-5.17145	2.482629	-2.08305		0.037246418	NA	NDUFAF2
NDUFB1P	8.057665	-3.95647	1.673103	-2.36475		0.018042145	NA	NDUFB1P1
NIFKP6	14.99232	-2.901	1.063645	-2.72741		0.006383294	NA	NIFKP6
NLRP1	4.241396	-5.50254	2.341686	-2.34982		0.018782513	NA	NLRP1
NPM2	17.55208	2.458116	0.95902	2.563153		0.010372621	NA	NPM2
NPPB	8.658795	6.601862	1.928138	3.423958		0.000617161	NA	NPPB
NSA2P3	11.82142	-2.28925	1.146435	-1.99684		0.045842441	NA	NSA2P3
NTRK1	3.348161	5.226798	2.493372	2.096277		0.03605764	NA	NTRK1
NYAP2	17.49022	2.102933	1.034208	2.033375		0.04201466	NA	NYAP2
OCA2	8.596269	3.482253	1.525702	2.282394		0.022466078	NA	OCA2
P4HA3-AS	10.91803	2.424114	1.216236	1.993128		0.046247392	NA	P4HA3-AS
PARM1-A	5.609444	-4.41771	2.069041	-2.13515		0.032748771	NA	PARM1-A
PCAT14	6.186574	-6.04621	2.089791	-2.89321		0.003813229	NA	PCAT14
PCAT18	10.90249	3.032254	1.273921	2.380253		0.017300766	NA	PCAT18
PCAT19	4.268447	-5.51112	2.323251	-2.37216		0.017684503	NA	PCAT19
PCDHGA1	13.02441	3.011338	1.163917	2.587244		0.009674697	NA	PCDHGA1
PDC-AS1	15.26892	2.069011	0.997087	2.075055		0.037981395	NA	PDC-AS1
PIGR	9.251081	-4.10416	1.573109	-2.60895		0.009082046	NA	PIGR

PKHD1	7.864737	3.353414	1.655673	2.025408	0.042825447	NA	PKHD1
POLR2A	12.72488	-3.18261	1.180612	-2.69573	0.007023553	NA	POLR2A
PRF1	3.717237	5.374637	2.436554	2.205835	0.027395541	NA	PRF1
PTCHD3P2	3.762544	5.39106	2.448234	2.20202	0.027663926	NA	PTCHD3P2
RASAL3	4.371394	5.611696	2.291878	2.448515	0.01434466	NA	RASAL3
RASGEF1B	15.40971	-4.31752	1.275364	-3.38532	0.00071095	NA	RASGEF1B
RAX	9.992556	6.802295	1.881247	3.615843	0.000299372	NA	RAX
RBP2	11.88568	2.842154	1.183575	2.401331	0.016335554	NA	RBP2
RCAN2	4.047625	5.502414	2.344264	2.347182	0.018916006	NA	RCAN2
RERG-AS1	3.393468	5.244803	2.489908	2.106425	0.035167479	NA	RERG-AS1
RFX6	12.98178	-3.62901	1.2878	-2.81799	0.004832477	NA	RFX6
RHAG	5.862583	-5.96596	2.107483	-2.83085	0.004642503	NA	RHAG
RNA5SP14	16.96973	-6.04829	1.6677	-3.62672	0.00028704	NA	RNA5SP14
RNA5SP16	3.836254	-5.35601	2.386182	-2.2446	0.024794043	NA	RNA5SP16
RNA5SP38	5.052298	-5.74845	2.270684	-2.53159	0.011354558	NA	RNA5SP38
RNA5SP99	14.87255	-7.3092	1.735722	-4.21105	2.54E-05	NA	RNA5SP99
RNF223	7.73954	3.898722	1.623359	2.401638	0.016321851	NA	RNF223
RNU7-47P	7.835415	3.229345	1.590967	2.0298	0.042376888	NA	RNU7-47P
RNY4P13	3.069699	5.102241	2.564786	1.989344	0.046663264	NA	RNY4P13
ROBO4	6.114076	3.533969	1.731266	2.041263	0.041224659	NA	ROBO4
RPL10AP5	4.22818	-5.49272	2.335836	-2.3515	0.018697791	NA	RPL10AP5
RPL21P72	10.0759	2.859832	1.29935	2.200972	0.027738023	NA	RPL21P72
RPL26P23	12.837	3.611725	1.219781	2.960963	0.003066788	NA	RPL26P23
RPL31P43	3.807851	5.407446	2.471508	2.187914	0.028675877	NA	RPL31P43
RPL35AP9	6.70323	-3.52859	1.743883	-2.02341	0.043031164	NA	RPL35AP9
RPL37AP8	6.600817	3.603566	1.776669	2.028271	0.042532619	NA	RPL37AP8
RPL5P11	7.606706	3.260156	1.533175	2.126408	0.033469263	NA	RPL5P11
RPL7AP63	8.728053	-3.40588	1.450095	-2.34873	0.018837788	NA	RPL7AP63
RPLP1P8	14.32638	-2.11318	1.068872	-1.97702	0.048039483	NA	RPLP1P8
RPS10P6	17.50427	1.938422	0.951194	2.037883	0.041561638	NA	RPS10P6
RPS29P7	10.05239	3.229129	1.340857	2.408258	0.016028838	NA	RPS29P7
RPSAP18	9.035773	3.060741	1.487143	2.058134	0.03957725	NA	RPSAP18
SALRNA3	11.42792	3.079232	1.244603	2.474067	0.013358477	NA	SALRNA3
SCGN	11.7728	-2.84938	1.340988	-2.12484	0.033600222	NA	SCGN
SEC14L3	15.41507	3.260115	1.073971	3.035571	0.002400805	NA	SEC14L3
SELENOKP	14.74505	2.76592	1.112956	2.485201	0.012947814	NA	SELENOKP
SERPINB5	5.929901	-5.98538	2.118293	-2.82557	0.0047197	NA	SERPINB5
SH3GL3	16.0173	3.320258	1.061722	3.127239	0.001764567	NA	SH3GL3
SHD	5.322187	-5.82743	2.161388	-2.69615	0.007014592	NA	SHD
SLC25A2	13.70356	2.628003	1.101597	2.385629	0.017049927	NA	SLC25A2
SLC2A9	14.5019	-2.13526	1.063818	-2.00717	0.044731738	NA	SLC2A9
SLC43A2	10.29687	-5.31677	1.813789	-2.93131	0.003375374	NA	SLC43A2
SLC9A4	8.972398	-3.99423	1.591182	-2.51023	0.012065386	NA	SLC9A4
SNORD114	8.70378	3.015364	1.455275	2.072024	0.038263213	NA	SNORD114
SPART-AS	3.606632	-5.26668	2.431544	-2.16598	0.030312505	NA	SPART-AS
SPINK4	11.23917	-3.40874	1.357229	-2.51154	0.012020515	NA	SPINK4
ST8SIA6	17.29224	1.993163	0.978338	2.037294	0.041620541	NA	ST8SIA6
TAF1L	9.609806	-3.11492	1.367026	-2.27861	0.022690324	NA	TAF1L
TAGAP	5.070859	5.827332	2.192364	2.658013	0.007860275	NA	TAGAP
TCAF2	15.15712	5.947616	1.707357	3.483522	0.000494862	NA	TCAF2
THPO	17.12376	2.303329	1.05332	2.186732	0.028762106	NA	THPO
TMEM140	9.161986	3.517519	1.428752	2.461951	0.013818346	NA	TMEM140
TPTE2	7.6907	3.2711	1.514919	2.159258	0.030830182	NA	TPTE2
TRG-GCC2	13.86684	-2.79719	1.132201	-2.47058	0.013489389	NA	TRG-GCC2
TRL-CAG1	3.22854	-5.10368	2.539557	-2.00967	0.044465731	NA	TRL-CAG1
TRR-CCT5	14.78254	-2.05991	1.022444	-2.01469	0.043937063	NA	TRR-CCT5
TRY-GTA5	4.174078	-5.47536	2.325971	-2.35401	0.018571995	NA	TRY-GTA5
TSPAN32	11.47656	2.499848	1.228956	2.034122	0.041939254	NA	TSPAN32

TSPOAP1	3.431111	-5.19276	2.472824	-2.09993	0.035734854	NA		TSPOAP1
TUBBP5	5.533635	4.462117	2.082518	2.142655	0.032140846	NA		TUBBP5
UGT1A1	16.72013	-4.43763	1.245859	-3.5619	0.000368181	NA		UGT1A1
WARS2-IT	3.174439	-5.08095	2.534957	-2.00435	0.045032101	NA		WARS2-IT
WDR46	3.458162	-5.20332	2.474579	-2.10271	0.035491208	NA		WDR46
WFDC10B	6.644395	6.212576	2.073152	2.996682	0.002729354	NA		WFDC10B
WFDC13	6.599088	6.2033	2.066945	3.001193	0.002689244	NA		WFDC13
WFDC3	12.31877	3.544532	1.23976	2.859047	0.004249154	NA		WFDC3
WWP1P1	3.762544	5.39106	2.448234	2.20202	0.027663926	NA		WWP1P1
ZNF806	3.069699	5.102241	2.564786	1.989344	0.046663264	NA		ZNF806

ɔol

4985

7156

4020

B1

A

¡

G

4660

4791

1
-1

2

7789

,

4908

4019

A

[illegible]1

2

[illegible]

A

S1

[illegible]

8653

0

3961

9034

4

-

AS1

3

8798

A
-

2838

8301

AS1

[illegible]

3409

6148

7507

[illegible]

.P1

3

2

)
4234

.1

8465

0924

6544
2

6951

S1
9301

AS1
3

5

0027

2

T
5

3

1

8726

8267

8089
3
5754

1822

8419
4790

5
1291

8481

6558

5579

7824

2692
S1

.

9743
7152
1

3
9555

8480

AS1

-AS2

1492

4476
8626
2814
6
8031
[illegible]

8678

8261

[illegible]

3581
2

[illegible]
4291

[illegible]

[illegible]
2717

9

0

.

0686

6

4889

7387

1

8762
4808

4811

8252
2
4118
6

9337

8079

0

0461

8

4262
8474

0

5282
8271

2

5334

6986
3

5118
6167

9746

6

6373

8228
4

8255
4
AS1

[illegible]

1578

7558

S1

5216

S1

-

8452

4

7366
6722
,

2

:

-

0082
7413

3

3
51

6397

1

6504

7790

T

2
6648
P
6934

1

[illegible]

[illegible]

1

6414

8842

-AS1

9570

3148

5752
-AS1

3424

8277
9
C

S2

S1

1224

7744

6202

8399

1

7825

8936
T

5170

4394

2345

5

7250
IS
-DT
6876
8525

AS1
)
8133

3018
6168

S1

5

7275
1204

8674

8768

8168
1856

7420

5244
1934

8841

AC6

5684
4858

3809

A

8489

2

1064

4740

3618

4573

2

AS1

-AS1

1

4333

3

6269

4282

2

0872

7103

8526

6113

2

-8

.3

2

9495

8520
6103
9
AS1
4763

2945
0207
4808

9
3717

1776

6655

8076
4

9256

9675
7622
8725

3041
5553

1

3444

3

-AS1
5293
8659
6114
2

3311

8000
3218
7632

P1

4120
3046

5
3814
2424

8034
7320

,

3949
ł

9515

4906
ł

6

;
3

,

2705
3369

4397

S1

7401

5284

6505

7650
-AS1

7385

9381
6196

3
8241
9534
B
9
7223
4715
2767
2
6
4952
4322
9395
3
6199
1813
8211
6155
1
8701
6083
-1
6672
?1
8175
9451

2431

2449

S1

9477
S1

6424
-1

5362

7175
-1
DT
5416
0227
4814

4844

8066

1

6885

2-1
5599
AS1

4-21

1

4-1

7809

2768
6042

5119
1483

6497
0

6127
8420

9561
9057
3
7172

A

1849

2

2

A

1

I18.1

S

:7L2

5

S1

.6

AS1
3
4
5
9
5
5
3
3
5
3
7
4
9
5
7
3
5

4
0
2
-
-
-

9878
0582
1293
2032
5851
6281
7325
7623
8126
8477
8651
9128
9384
9727
3408
3765
9975
0007
0575
0588
1056
1574
2756
2825
3883
3954
4164
5310
5760
5821
5920
6485
6579
7124
7196
7547
7627
7855
8085
8199
8208
8269
8433
8476
8477
8570
4435
4516
4552
4564
4576
4698

4781
4827
5504
5541
5805
6140
6169
6433
6473
6487
6571
6649
7115
7118
7918
8418
8444
7592
4
9
5
2

[illegible]
[illegible]-AS1

[illegible]

[illegible]
[illegible]1
[illegible]

[illegible]1
[illegible]1

2

2

9
1
7

1

4-2
1

2-5
-6
-1
-3

1
