# Supplementary material for: A Novel Hypoxia-Related Gene Signature with Strong Predicting Ability in Non-Small-Cell Lung Cancer Identified by Comprehensive Profiling
Source: Int J Genomics. 2022 May 19;2022:8594658. doi: 10.1155/2022/8594658 (PMC9135579; doi:10.1155/2022/8594658)
Supplement: Supplementary Materials — Supplementary Figure S1: the expressions ofCNKSR3, DGAT2, FAMB1A, SERPINE1, TGFB1, and TMEM132B in the ceRNA network showed no significant difference between NSCLC and normal samples. Supplementary Figure S2: no significant difference of survival between groups divided by gender or expression of ADM, BHLHE40, BIRC5, C1QL1, C11orf86, CCNA2, CCND3, CNKSR3, DKK1, DKK3, DGAT2, ETV1, FAM81A, FAM160A1, HECA, HMGA2, HOXC8, ISOC1, KDM7A, NECTIN1, HNRNPA2B1, PAM, PEA15, PPIH, PPP1R3B, RASGEF1B, SLC12A2, ZWILCH, WSB1, TMEM132B, or STC2. Supplementary Table S1: identification of 1293 upregulated DEGs and 746 downregulated DEGs in hypoxia-treated A549 cells compared to normoxia-treated A549 cells displayed in the heat map. Supplementary Table S2: 21 upregulated DEcircRNAs and 49 downregulated DEcircRNAs identified in hypoxia-treated A549 cells compared to normoxia-treated A549 cells. Supplementary Table S3 and S4: upregulated DEGs were significantly enriched into 284 GO terms and 42 KEGG pathways. Supplementary Table S5 and S6: downregulated DEGs were significantly enriched into 184 GO terms and 25 KEGG pathways. [file 8594658.f1.zip › Supplementary 2_DEcircRNA_sig.pdf]

Supplementary Table S2. 21 up-regulated DEcircRNAs and 49 down-regulated DEcircRNAs identified in hypoxia treated A549 cells compared to normoxia treated A549 cells.

GeneID	Genomic.c	Genomic.s	Host.gene	Ensembl.g	Host.gene	circBase.ID	circRNAdb	Confidenc
1686	chr7:5792	28197	PRKAR1B	ENSG0000	protein_c	hsa_circ_0	hsa_circ_1	High
2320	chr9:9347	27746	FAM120A	ENSG0000	protein_c	hsa_circ_0	hsa_circ_2	High
46	chr1:2108	22246	EIF4G3	ENSG0000	protein_c	hsa_circ_0	hsa_circ_0	High
170	chr1:9514	29999	TMEM56	ENSG0000	protein_c	hsa_circ_0	hsa_circ_0	High
339	chr1:2249	54875	DNAH14	ENSG0000	protein_c	hsa_circ_0	hsa_circ_0	High
404	chr1:2463	8274	SMYD3	ENSG0000	protein_c	hsa_circ_0	hsa_circ_2	High
424	chr2:1539	23486	NBAS	ENSG0000	protein_c	hsa_circ_0	hsa_circ_1	Low
462	chr2:3055	15684	LCLAT1	ENSG0000	protein_c	hsa_circ_0	hsa_circ_2	High
550	chr2:6274	23998	EHBP1	ENSG0000	protein_c	hsa_circ_0	hsa_circ_0	Low
699	chr2:1906	17176	NAB1	ENSG0000	protein_c	hsa_circ_0	hsa_circ_3	High
741	chr2:2274	33369	AGFG1	ENSG0000	protein_c	hsa_circ_0	hsa_circ_0	High
753	chr2:2399	17297	NDUFA10	ENSG0000	protein_c	hsa_circ_0	hsa_circ_0	High
807	chr3:2743	11410	SLC4A7	ENSG0000	protein_c	hsa_circ_0	hsa_circ_2	High
862	chr3:5789	6892	SLMAP	ENSG0000	protein_c	hsa_circ_0	hsa_circ_3	High
911	chr3:1146	24000	ZBTB20	ENSG0000	protein_c	hsa_circ_0	NA	Low
1094	chr4:3973	18320	UBE2K	ENSG0000	protein_c	hsa_circ_0	hsa_circ_3	High
1136	chr4:7777	3312	CNOT6L	ENSG0000	protein_c	hsa_circ_0	hsa_circ_2	High
1238	chr4:1773	421	NEIL3	ENSG0000	protein_c	hsa_circ_0	hsa_circ_0	High
1334	chr5:6605	1546	ERBB2IP	ENSG0000	protein_c	hsa_circ_0	hsa_circ_1	High
1362	chr5:7808	39915	AP3B1	ENSG0000	protein_c	hsa_circ_0	hsa_circ_1	High
1376	chr5:8077	17151	MSH3	ENSG0000	protein_c	hsa_circ_0	hsa_circ_1	High
1461	chr5:1404	9187	ANKHD1	ENSG0000	protein_c	hsa_circ_0	hsa_circ_2	High
1476	chr5:1462	3651	RP11-449	ENSG0000	protein_c	hsa_circ_0	hsa_circ_1	Low
1553	chr6:1766	4308	NUP153	ENSG0000	protein_c	hsa_circ_0	hsa_circ_3	High
1642	chr6:1363	1125	MAP7	ENSG0000	protein_c	hsa_circ_0	hsa_circ_1	High
1684	chr6:1703	13181	FAM120B	ENSG0000	protein_c	hsa_circ_0	hsa_circ_0	High
1883	chr7:1114	74686	IMMP2L	ENSG0000	protein_c	hsa_circ_0	hsa_circ_1	High
2001	chr8:2639	17134	BNIP3L	ENSG0000	protein_c	hsa_circ_0	hsa_circ_1	High
2036	chr8:4295	7381	HOOK3	ENSG0000	protein_c	hsa_circ_0	hsa_circ_2	High
2061	chr8:6074	1839	CHD7	ENSG0000	protein_c	hsa_circ_0	hsa_circ_0	High
2139	chr8:1084	6108	EMC2	ENSG0000	protein_c	hsa_circ_0	NA	High
2156	chr8:1278	410	PVT1	ENSG0000	lincRNA	hsa_circ_0	hsa_circ_1	High
2289	chr9:7290	17576	ALDH1A1	ENSG0000	protein_c	NA	NA	High
2318	chr9:9347	28558	FAM120A	ENSG0000	protein_c	hsa_circ_0	NA	High
2386	chr9:1162	28182	PAPPA	ENSG0000	protein_c	hsa_circ_0	hsa_circ_2	High
2416	chr9:1358	32942	CAMSAP1	ENSG0000	protein_c	hsa_circ_0	hsa_circ_1	High
2533	chr10:343	36290	PARD3	ENSG0000	protein_c	hsa_circ_0	NA	High
2668	chr10:118	1116	CACUL1	ENSG0000	protein_c	hsa_circ_0	hsa_circ_1	High
2744	chr11:331	498	CSTF3	ENSG0000	protein_c	hsa_circ_0	hsa_circ_2	High
2747	chr11:340	4917	CAPRIN1	ENSG0000	protein_c	hsa_circ_0	hsa_circ_1	High
2944	chr12:307	22486	CAPRIN2	ENSG0000	protein_c	hsa_circ_0	hsa_circ_0	High
2950	chr12:410	14992	CNTN1	ENSG0000	protein_c	hsa_circ_0	hsa_circ_0	Low
3042	chr12:928	25114	EEA1	ENSG0000	protein_c	hsa_circ_0	hsa_circ_0	High
3252	chr14:307	18935	SCFD1	ENSG0000	protein_c	hsa_circ_0	hsa_circ_1	High
3273	chr14:345	16698	SNX6	ENSG0000	protein_c	hsa_circ_0	hsa_circ_2	High
3324	chr14:617	315	HIF1A	ENSG0000	protein_c	hsa_circ_0	hsa_circ_3	High
3401	chr14:103	18774	PPP1R13B	ENSG0000	protein_c	hsa_circ_0	hsa_circ_0	High
3498	chr15:604	3376	ICE2	ENSG0000	protein_c	hsa_circ_0	NA	High
3501	chr15:619	53256	VPS13C	ENSG0000	protein_c	NA	NA	Low
3511	chr15:636	20350	HERC1	ENSG0000	protein_c	hsa_circ_0	hsa_circ_0	High
3567	chr15:762	4855	ETFA	ENSG0000	protein_c	hsa_circ_0	hsa_circ_2	High
3571	chr15:771	3251	PEAK1	ENSG0000	protein_c	hsa_circ_0	NA	High
3724	chr16:474	18203	PHKB	ENSG0000	protein_c	hsa_circ_0	hsa_circ_0	High
3831	chr17:428	14017	UBE2G1	ENSG0000	protein_c	hsa_circ_0	hsa_circ_0	High
3845	chr17:120	17786	MAP2K4	ENSG0000	protein_c	hsa_circ_0	hsa_circ_2	High
3878	chr17:285	1369	SPAG5	ENSG0000	protein_c	hsa_circ_0	hsa_circ_0	Low
4015	chr17:597	42465	VMP1	ENSG0000	protein_c	hsa_circ_0	hsa_circ_0	High

4019	chr17:6026	2032	USP32	ENSG0000	protein_cc	hsa_circ_0(	hsa_circ_2!	High
4126	chr18:1053	15789	NAPG	ENSG0000	protein_cc	hsa_circ_0(	hsa_circ_2	High
4133	chr18:1299	19786	CEP192	ENSG0000	protein_cc	hsa_circ_0(	hsa_circ_2(	High
4205	chr19:5047	35029	KDM4B	ENSG0000	protein_cc	hsa_circ_0(	hsa_circ_2	High
4232	chr19:2998	1195	URI1	ENSG0000	protein_cc	hsa_circ_0(	hsa_circ_2(	High
4255	chr19:3443	6906	UBA2	ENSG0000	protein_cc	hsa_circ_0(	hsa_circ_1(	High
4349	chr20:3471	776	NCOA6	ENSG0000	protein_cc	hsa_circ_0(	NA	High
4358	chr20:3572	3416	RBM39	ENSG0000	protein_cc	hsa_circ_0(	hsa_circ_1(	High
4395	chr20:5415	13585	CYP24A1	ENSG0000	protein_cc	NA	NA	Low
4542	chrX:5361	1302	HUWE1	ENSG0000	protein_cc	hsa_circ_0(	hsa_circ_0(	High
4556	chrX:8028	21328	CHMP1B2	ENSG0000	unitary_ps	hsa_circ_0	hsa_circ_0	High
4570	chrX:1101	2034	TMEM164	ENSG0000	protein_cc	hsa_circ_0	NA	High
4582	chrX:1407	1485	CDR1-AS	NA	NA	hsa_circ_0(	NA	High

baseMean	logFC	lfcSE	stat	PValue	FDR
20.21576	4.222558	0.838534	5.035642	4.76E-07	0.00219
15.52272	-2.91401	0.695993	-4.18683	2.83E-05	0.065047
2.326165	-4.80618	2.34818	-2.04677	0.040681	0.865159
9.26434	3.003294	0.951892	3.155079	0.001605	0.865159
11.69866	1.474802	0.654157	2.254509	0.024164	0.865159
6.460344	2.020126	1.027142	1.966744	0.049213	0.865159
2.590843	-4.96121	2.269627	-2.18591	0.028822	0.865159
3.906289	-3.9984	1.991889	-2.00734	0.044713	0.865159
3.1202	-5.22885	2.179326	-2.3993	0.016427	0.865159
4.33537	2.908345	1.476016	1.970402	0.048792	0.865159
2.298193	-4.7908	2.4248	-1.97575	0.048183	0.865159
6.866188	-3.18092	1.101852	-2.88688	0.003891	0.865159
3.62376	5.255951	1.947538	2.698767	0.00696	0.865159
2.869507	-5.10774	2.33155	-2.1907	0.028473	0.865159
2.782864	4.87186	2.284185	2.132866	0.032936	0.865159
3.218405	5.090816	2.485296	2.048375	0.040523	0.865159
3.1202	-5.22885	2.179326	-2.3993	0.016427	0.865159
3.599653	-3.8814	1.808339	-2.14639	0.031842	0.865159
4.515449	-3.21793	1.502073	-2.14233	0.032167	0.865159
5.586125	2.653939	1.253809	2.116701	0.034285	0.865159
2.576857	-4.95438	2.232096	-2.21961	0.026445	0.865159
4.323761	-4.1662	1.812472	-2.29863	0.021526	0.865159
2.326165	-4.80618	2.34818	-2.04677	0.040681	0.865159
2.312179	-4.79858	2.345381	-2.04597	0.040759	0.865159
5.572255	-2.36681	1.097021	-2.15749	0.030967	0.865159
2.312179	-4.79858	2.345381	-2.04597	0.040759	0.865159
2.562872	-4.94754	2.26321	-2.18607	0.02881	0.865159
3.833984	5.338162	1.906395	2.800134	0.005108	0.865159
2.34015	-4.81392	2.431537	-1.97979	0.047728	0.865159
5.410447	-4.50797	1.675016	-2.6913	0.007117	0.865159
8.932578	-2.24973	0.948794	-2.37115	0.017733	0.865159
8.050792	-2.02289	0.842021	-2.40242	0.016287	0.865159
4.25179	2.866681	1.433308	2.000046	0.045495	0.865159
3.328936	-5.32509	2.055214	-2.59102	0.009569	0.865159
4.826635	-3.27747	1.331475	-2.46154	0.013834	0.865159
7.980709	2.034738	1.034815	1.966281	0.049266	0.865159
4.622449	-3.15865	1.500236	-2.10544	0.035253	0.865159
2.785593	-5.06902	2.501259	-2.02659	0.042705	0.865159
2.312179	-4.79858	2.345381	-2.04597	0.040759	0.865159
7.414081	-3.26573	1.24582	-2.62135	0.008758	0.865159
2.507596	4.723862	2.263552	2.086925	0.036895	0.865159
3.609089	-3.92211	1.988676	-1.97222	0.048584	0.865159
2.895522	4.935387	2.137674	2.308765	0.020957	0.865159
4.303736	-5.69658	2.214714	-2.57215	0.010107	0.865159
5.600111	2.644413	1.2565	2.104585	0.035327	0.865159
3.836361	-3.98237	1.785303	-2.23064	0.025705	0.865159
2.590843	-4.96121	2.269627	-2.18591	0.028822	0.865159
4.136958	-5.63654	1.89368	-2.9765	0.002916	0.865159
3.283449	5.119103	2.167776	2.361454	0.018203	0.865159
3.334975	-3.76276	1.834139	-2.05151	0.040217	0.865159
2.562872	-4.94754	2.26321	-2.18607	0.02881	0.865159
3.864332	-3.99084	1.791887	-2.22717	0.025936	0.865159
6.850294	-2.30192	1.045663	-2.2014	0.027708	0.865159
4.163706	3.910958	1.785643	2.190224	0.028508	0.865159
5.841484	-2.4212	1.103034	-2.19503	0.028161	0.865159
2.298193	-4.7908	2.4248	-1.97575	0.048183	0.865159
4.371757	-3.0647	1.552039	-1.97463	0.04831	0.865159

3.454005	3.636053	1.841574	1.974427	0.048333	0.865159
2.326165	-4.80618	2.34818	-2.04677	0.040681	0.865159
21.95849	-1.16835	0.446634	-2.61591	0.008899	0.865159
9.138496	2.309195	1.04324	2.213483	0.026864	0.865159
13.35681	-1.32186	0.602153	-2.19522	0.028148	0.865159
3.370893	-5.34068	2.066355	-2.58459	0.00975	0.865159
2.841536	-5.09495	2.15526	-2.36396	0.018081	0.865159
5.277228	2.53821	1.162112	2.184135	0.028952	0.865159
2.326165	-4.80618	2.34818	-2.04677	0.040681	0.865159
3.348961	-3.76713	1.854366	-2.03149	0.042205	0.865159
3.836361	-3.98237	1.785303	-2.23064	0.025705	0.865159
2.928044	4.949518	2.103189	2.35334	0.018606	0.865159
25.26474	-1.17687	0.471212	-2.49754	0.012506	0.865159
