# Supplementary material for: A Novel Hypoxia-Related Gene Signature with Strong Predicting Ability in Non-Small-Cell Lung Cancer Identified by Comprehensive Profiling
Source: Int J Genomics. 2022 May 19;2022:8594658. doi: 10.1155/2022/8594658 (PMC9135579; doi:10.1155/2022/8594658)
Supplement: Supplementary Materials — Supplementary Figure S1: the expressions ofCNKSR3, DGAT2, FAMB1A, SERPINE1, TGFB1, and TMEM132B in the ceRNA network showed no significant difference between NSCLC and normal samples. Supplementary Figure S2: no significant difference of survival between groups divided by gender or expression of ADM, BHLHE40, BIRC5, C1QL1, C11orf86, CCNA2, CCND3, CNKSR3, DKK1, DKK3, DGAT2, ETV1, FAM81A, FAM160A1, HECA, HMGA2, HOXC8, ISOC1, KDM7A, NECTIN1, HNRNPA2B1, PAM, PEA15, PPIH, PPP1R3B, RASGEF1B, SLC12A2, ZWILCH, WSB1, TMEM132B, or STC2. Supplementary Table S1: identification of 1293 upregulated DEGs and 746 downregulated DEGs in hypoxia-treated A549 cells compared to normoxia-treated A549 cells displayed in the heat map. Supplementary Table S2: 21 upregulated DEcircRNAs and 49 downregulated DEcircRNAs identified in hypoxia-treated A549 cells compared to normoxia-treated A549 cells. Supplementary Table S3 and S4: upregulated DEGs were significantly enriched into 284 GO terms and 42 KEGG pathways. Supplementary Table S5 and S6: downregulated DEGs were significantly enriched into 184 GO terms and 25 KEGG pathways. [file 8594658.f1.zip › Supplementary Table S3_mRNA_UP_AllenrichGO and S4_mRNA_UP_AllEnrichKEGG.pdf]

Supplementary Table S3 and S4. Up-regulated DEGs were significantly enriched into 284 GO terms and 42 KEGG pathways.

Supplementary Table S3

Terms	Counts	GeneRatio	BgRatio	pValue	FDR	foldEnrich	geneID	geneSymb
GO:000673	15	15/791	29/20610	2.38E-14	3.94E-11	13.47705	ENSG0000	PGK1/ENO
GO:004306	55	55/791	474/20610	2.56E-13	1.82E-10	3.023332	ENSG0000	LOXL2/SER
GO:003158	44	44/791	385/20610	1.09E-10	4.93E-08	2.977786	ENSG0000	SERPINE1/
GO:009748	34	34/791	290/20610	7.66E-09	2.53E-06	3.054798	ENSG0000	VEGFA/CX
GO:000166	39	39/791	407/20610	1.62E-07	2.01E-05	2.496731	ENSG0000	LOXL2/STC
GO:000756	25	25/791	206/20610	3.80E-07	4.11E-05	3.162091	ENSG0000	STC2/APO
GO:007048	40	40/791	443/20610	5.36E-07	5.43E-05	2.352652	ENSG0000	LOXL2/STC
GO:001081	26	26/791	230/20610	8.93E-07	7.78E-05	2.945419	ENSG0000	SERPINE1/
GO:003240	30	30/791	295/20610	1.27E-06	0.000104	2.649725	ENSG0000	DAPK1/SY
GO:006074	8	8/791	24/20610	1.93E-06	0.000137	8.685209	ENSG0000	VEGFA/DD
GO:006137	8	8/791	24/20610	1.93E-06	0.000137	8.685209	ENSG0000	VEGFA/DD
GO:006056	27	27/791	255/20610	1.99E-06	0.000139	2.758831	ENSG0000	VEGFA/CX
GO:003362	13	13/791	70/20610	2.23E-06	0.000153	4.838902	ENSG0000	SERPINE1/
GO:000301	41	41/791	492/20610	3.02E-06	0.000201	2.171302	ENSG0000	CXCR4/STC
GO:000599	30	30/791	311/20610	3.77E-06	0.00024	2.513404	ENSG0000	SLC2A3/PF
GO:004561	6	6/791	13/20610	4.27E-06	0.000259	12.02567	ENSG0000	CDSN/NA
GO:000301	17	17/791	125/20610	5.91E-06	0.000333	3.543565	ENSG0000	ADM/PDG
GO:004426	28	28/791	293/20610	9.39E-06	0.000486	2.489957	ENSG0000	PFKFB4/SL
GO:004858	25	25/791	259/20610	2.35E-05	0.001053	2.515022	ENSG0000	VEGFA/CX
GO:004568	7	7/791	25/20610	3.13E-05	0.001321	7.295575	ENSG0000	CDH3/CDS
GO:000155	37	37/791	470/20610	3.17E-05	0.001321	2.051188	ENSG0000	VEGFA/CX
GO:005127	34	34/791	419/20610	3.58E-05	0.001471	2.114299	ENSG0000	SERPINE1/
GO:000716	29	29/791	336/20610	4.42E-05	0.001756	2.248849	ENSG0000	SERPINE1/
GO:003433	28	28/791	320/20610	4.68E-05	0.001843	2.279867	ENSG0000	VEGFA/TN
GO:000756	10	10/791	57/20610	5.51E-05	0.002073	4.571162	ENSG0000	STC2/STC1
GO:200014	31	31/791	380/20610	7.09E-05	0.002569	2.125591	ENSG0000	SERPINE1/
GO:190352	11	11/791	75/20610	0.000129	0.00424	3.821492	ENSG0000	ADM/HRH
GO:004001	34	34/791	451/20610	0.000151	0.004908	1.964282	ENSG0000	SERPINE1/
GO:005067	20	20/791	211/20610	0.000191	0.005937	2.469728	ENSG0000	ITGB3/VEG
GO:000170	15	15/791	140/20610	0.000321	0.009327	2.791674	ENSG0000	ITGB3/COL
GO:003241	12	12/791	97/20610	0.000336	0.009617	3.223376	ENSG0000	NEDD4/EP
GO:004484	5	5/791	17/20610	0.000346	0.009617	7.663419	ENSG0000	OXTR/GNI
GO:000941	26	26/791	325/20610	0.000358	0.009877	2.08445	ENSG0000	PDGFB/CX
GO:004269	10	10/791	71/20610	0.000363	0.009972	3.669806	ENSG0000	PAM/AXL/
GO:003283	4	4/791	10/20610	0.000376	0.01011	10.42225	ENSG0000	COL4A3/C
GO:000821	18	18/791	190/20610	0.000395	0.010555	2.468428	ENSG0000	ADM/PDG
GO:009028	25	25/791	310/20610	0.000409	0.010697	2.10126	ENSG0000	ITGB3/VEG
GO:000803	8	8/791	49/20610	0.000508	0.01254	4.25398	ENSG0000	CXCR4/AP
GO:003241	13	13/791	116/20610	0.000512	0.012598	2.920027	ENSG0000	SYNGR3/A
GO:004327	17	17/791	180/20610	0.000587	0.01416	2.460809	ENSG0000	STC1/NED
GO:000110	28	28/791	374/20610	0.000626	0.014797	1.950689	ENSG0000	VEGFA/PD
GO:004311	7	7/791	41/20610	0.000859	0.019075	4.448521	ENSG0000	ADM/VEG
GO:000193	18	18/791	203/20610	0.000866	0.019075	2.310351	ENSG0000	LOXL2/ITG
GO:190401	20	20/791	238/20610	0.000903	0.019422	2.189548	ENSG0000	SERPINE1/
GO:001046	17	17/791	191/20610	0.001142	0.024134	2.319087	ENSG0000	SERPINE1/
GO:004239	32	32/791	467/20610	0.001191	0.024854	1.785396	ENSG0000	BNIP3L/BN
GO:004434	5	5/791	22/20610	0.001256	0.02599	5.921733	ENSG0000	IGFBP5/ER
GO:006201	15	15/791	160/20610	0.0013	0.026576	2.442715	ENSG0000	PFKFB4/PC
GO:003476	14	14/791	144/20610	0.001314	0.026644	2.533186	ENSG0000	GRB10/NE
GO:004568	11	11/791	100/20610	0.001573	0.029905	2.866119	ENSG0000	KLF7/MAF
GO:000961	18	18/791	216/20610	0.001751	0.031845	2.171302	ENSG0000	SLC2A1/BN
GO:190377	9	9/791	74/20610	0.002049	0.035447	3.168927	ENSG0000	ATP1B1/FL
GO:001815	4	4/791	15/20610	0.002095	0.035992	6.948167	ENSG0000	LOXL2/LO
GO:004592	21	21/791	274/20610	0.00215	0.036707	1.996964	ENSG0000	STC2/MT1
GO:009917	32	32/791	488/20610	0.00239	0.039695	1.708566	ENSG0000	SORCS2/H
GO:004327	22	22/791	295/20610	0.002399	0.039718	1.943131	ENSG0000	PDGFB/STC
GO:007259	23	23/791	316/20610	0.002635	0.042524	1.896454	ENSG0000	BNIP3/PDC

GO:00328	8	8/791	63/20610	0.002723	0.042524	3.308651	ENSG0000	PDGFB/CC
GO:00459	7	7/791	51/20610	0.003176	0.047945	3.576262	ENSG0000	PTAFR/ED
GO:00018	19	19/791	248/2061	0.003427	0.050626	1.996197	ENSG0000	ITGB3/VEG
GO:00506	27	27/791	401/2061	0.00353	0.051712	1.754369	ENSG0000	ITGB3/VEG
GO:00020	5	5/791	28/20610	0.003876	0.055792	4.65279	ENSG0000	ADM/CAV
GO:00459	9	9/791	83/20610	0.004494	0.061481	2.825309	ENSG0000	PFKFB4/PD
GO:00458	10	10/791	100/2061	0.005052	0.067769	2.605563	ENSG0000	PDGFB/INS
GO:00720	13	13/791	150/2061	0.005217	0.069137	2.258154	ENSG0000	VEGFA/PD
GO:00620	31	31/791	497/2061	0.005734	0.071183	1.6252	ENSG0000	PFKFB4/PC
GO:00613	13	13/791	152/2061	0.005825	0.072135	2.228442	ENSG0000	ATP1B1/C
GO:00026	17	17/791	224/2061	0.005982	0.073535	1.977436	ENSG0000	AXL/INHA
GO:00230	31	31/791	500/2061	0.006239	0.07575	1.615449	ENSG0000	PPFIA4/AD
GO:00600	4	4/791	20/20610	0.006383	0.076202	5.211125	ENSG0000	FLNA/ICA
GO:00468	8	8/791	73/20610	0.006769	0.08023	2.855411	ENSG0000	KLF7/INH
GO:00060	26	26/791	403/2061	0.007149	0.082945	1.681008	ENSG0000	APOL2/AB
GO:00347	16	16/791	210/2061	0.007252	0.083055	1.985191	ENSG0000	ATP1B1/A
GO:00032	14	14/791	175/2061	0.007695	0.085681	2.08445	ENSG0000	CITED2/DI
GO:00108	11	11/791	123/2061	0.007774	0.086305	2.330178	ENSG0000	VEGFA/PL
GO:00508	18	18/791	249/2061	0.007786	0.086305	1.883539	ENSG0000	PDGFB/AX
GO:00107	5	5/791	33/20610	0.007996	0.08727	3.947822	ENSG0000	SMAD7/EF
GO:00106	14	14/791	177/2061	0.008469	0.090838	2.060897	ENSG0000	ITGB3/VEG
GO:00075	24	24/791	369/2061	0.008651	0.091602	1.694675	ENSG0000	SERPINE1/
GO:00603	5	5/791	34/20610	0.009085	0.095381	3.83171	ENSG0000	TIPARP/CS
GO:00905	3	3/791	12/20610	0.009557	0.098053	6.513906	ENSG0000	SPNS2/SPI
GO:00442	29	29/791	476/2061	0.010021	0.101145	1.587423	ENSG0000	PGK1/ENC
GO:00700	5	5/791	35/20610	0.010272	0.102223	3.722232	ENSG0000	DNAJB2/C
GO:00714	4	4/791	23/20610	0.010653	0.104962	4.531413	ENSG0000	WNT1/WN
GO:00301	8	8/791	79/20610	0.010764	0.105436	2.638544	ENSG0000	SERPINE1/
GO:00032	11	11/791	130/2061	0.011546	0.109865	2.204707	ENSG0000	CITED2/DI
GO:00216	8	8/791	80/20610	0.011571	0.109865	2.605563	ENSG0000	EPHB2/PLX
GO:19000	8	8/791	80/20610	0.011571	0.109865	2.605563	ENSG0000	SERPINE1/
GO:00451	10	10/791	113/2061	0.011661	0.110509	2.305808	ENSG0000	SPAG4/HK
GO:00019	3	3/791	13/20610	0.012074	0.111445	6.012837	ENSG0000	APP/OXTR
GO:00147	3	3/791	13/20610	0.012074	0.111445	6.012837	ENSG0000	IGFBP5/NC
GO:00435	4	4/791	24/20610	0.012403	0.112145	4.342604	ENSG0000	GLS/SLC5
GO:00601	7	7/791	66/20610	0.013061	0.116443	2.763475	ENSG0000	STC2/APO
GO:00305	18	18/791	263/2061	0.013185	0.116926	1.783275	ENSG0000	SERPINE1/
GO:00310	15	15/791	207/2061	0.014022	0.122811	1.888089	ENSG0000	ADM/JUN
GO:00196	3	3/791	14/20610	0.014934	0.126776	5.583348	ENSG0000	ARG2/CYP
GO:00600	3	3/791	14/20610	0.014934	0.126776	5.583348	ENSG0000	FLNA/ICA
GO:00219	11	11/791	135/2061	0.014999	0.126888	2.123051	ENSG0000	SLC2A1/B
GO:00508	8	8/791	84/20610	0.015244	0.128094	2.481488	ENSG0000	SERPINE1/
GO:00712	22	22/791	348/2061	0.015657	0.130241	1.647195	ENSG0000	SLC2A1/B
GO:01040	22	22/791	348/2061	0.015657	0.130241	1.647195	ENSG0000	SLC2A1/B
GO:00303	15	15/791	210/2061	0.015799	0.131199	1.861116	ENSG0000	DNAJB2/S
GO:00515	5	5/791	39/20610	0.016075	0.131951	3.340465	ENSG0000	GPER1/KM
GO:00716	5	5/791	39/20610	0.016075	0.131951	3.340465	ENSG0000	WNT11/CI
GO:00486	23	23/791	370/2061	0.016396	0.132227	1.619674	ENSG0000	STC2/VEG
GO:00995	4	4/791	26/20610	0.016429	0.132227	4.008558	ENSG0000	ITGB3/CA
GO:00509	17	17/791	250/2061	0.016622	0.132924	1.771783	ENSG0000	SERPINE1/
GO:00451	6	6/791	54/20610	0.016934	0.135199	2.89507	ENSG0000	PPL/VIM/E
GO:00347	12	12/791	156/2061	0.017285	0.137558	2.004279	ENSG0000	ADM/CRA
GO:00902	13	13/791	175/2061	0.017669	0.138836	1.935561	ENSG0000	FSTL3/TGF
GO:00016	14	14/791	194/2061	0.017803	0.138836	1.880303	ENSG0000	VEGFA/CX
GO:00468	19	19/791	292/2061	0.018019	0.138836	1.6954	ENSG0000	SLC2A1/KI
GO:00094	3	3/791	15/20610	0.018144	0.138836	5.211125	ENSG0000	PKD2L1/A
GO:00719	3	3/791	15/20610	0.018144	0.138836	5.211125	ENSG0000	ARG2/CYP
GO:00325	24	24/791	395/2061	0.018515	0.140373	1.583127	ENSG0000	VEGFA/CR

GO:003019	4	4/791	27/20610	0.018714	0.140384	3.860093	ENSG0000	SERPINE1/
GO:004593	4	4/791	27/20610	0.018714	0.140384	3.860093	ENSG0000	TNNT1/DC
GO:190000	4	4/791	27/20610	0.018714	0.140384	3.860093	ENSG0000	SERPINE1/
GO:005043	7	7/791	71/20610	0.018951	0.141309	2.568865	ENSG0000	SYT12/OX
GO:005123	22	22/791	355/20610	0.019204	0.142982	1.614715	ENSG0000	ITGB3/PLIN
GO:006032	5	5/791	41/20610	0.019659	0.145714	3.177515	ENSG0000	TIPARP/CS
GO:003134	10	10/791	124/20610	0.021152	0.154501	2.10126	ENSG0000	GAPDH/VA
GO:005082	4	4/791	28/20610	0.021187	0.154501	3.722232	ENSG0000	SERPINE1/
GO:001488	6	6/791	57/20610	0.021621	0.155772	2.742697	ENSG0000	IGFBP5/TN
GO:009890	6	6/791	57/20610	0.021621	0.155772	2.742697	ENSG0000	FLNA/CAV
GO:002154	14	14/791	199/20610	0.021663	0.155772	1.833059	ENSG0000	SLC2A1/BN
GO:009014	3	3/791	16/20610	0.021706	0.155772	4.88543	ENSG0000	BNIP3/PIN
GO:003090	25	25/791	423/20610	0.022292	0.158825	1.539931	ENSG0000	SLC2A1/BN
GO:004860	26	26/791	445/20610	0.022779	0.161834	1.522351	ENSG0000	STC2/ADM
GO:006201	9	9/791	108/20610	0.023142	0.164178	2.171302	ENSG0000	DDIT4/INS
GO:004578	28	28/791	489/20610	0.023542	0.165032	1.491938	ENSG0000	VEGFA/FS
GO:000697	8	8/791	91/20610	0.023595	0.165032	2.290604	ENSG0000	SLC2A1/EC
GO:002160	4	4/791	29/20610	0.023851	0.165425	3.593879	ENSG0000	EPHB2/PLX
GO:012016	9	9/791	109/20610	0.0244	0.168761	2.151382	ENSG0000	VEGFA/CX
GO:008606	6	6/791	59/20610	0.025189	0.173016	2.649725	ENSG0000	ATP1B1/FL
GO:003004	11	11/791	146/20610	0.025257	0.173238	1.963095	ENSG0000	STC1/TNN
GO:004547	3	3/791	17/20610	0.025622	0.174297	4.598052	ENSG0000	CIART/OPI
GO:002153	18	18/791	284/20610	0.026357	0.178561	1.651413	ENSG0000	SLC2A1/BN
GO:000688	4	4/791	30/20610	0.026709	0.179237	3.474083	ENSG0000	ANO6/SCT
GO:000247	6	6/791	60/20610	0.027112	0.180969	2.605563	ENSG0000	HLA-B/NA
GO:000176	15	15/791	225/20610	0.0274	0.182155	1.737042	ENSG0000	ADM/VEG
GO:005080	14	14/791	206/20610	0.028078	0.185915	1.770771	ENSG0000	EGFR/SYT1
GO:190303	7	7/791	77/20610	0.028211	0.186215	2.368693	ENSG0000	SERPINE1/
GO:001991	7	7/791	78/20610	0.030006	0.194866	2.338325	ENSG0000	ITGB3/PLIN
GO:007155	17	17/791	268/20610	0.030073	0.194866	1.652782	ENSG0000	JUN/TGFB
GO:009886	5	5/791	46/20610	0.030792	0.198585	2.832133	ENSG0000	COL12A1/
GO:009752	16	16/791	249/20610	0.031268	0.200075	1.674257	ENSG0000	SERPINE1/
GO:000315	7	7/791	79/20610	0.031877	0.202949	2.308726	ENSG0000	VEGFA/JUI
GO:004224	7	7/791	79/20610	0.031877	0.202949	2.308726	ENSG0000	PKM/GSN/
GO:007180	15	15/791	230/20610	0.032406	0.205004	1.69928	ENSG0000	ATP1B1/A
GO:005196	9	9/791	115/20610	0.03298	0.206483	2.039136	ENSG0000	APP/EPHB
GO:006113	14	14/791	211/20610	0.033449	0.206645	1.728809	ENSG0000	ADM/VEG
GO:190547	10	10/791	134/20610	0.033807	0.207883	1.94445	ENSG0000	EGFR/EPH
GO:000834	7	7/791	80/20610	0.033824	0.207883	2.279867	ENSG0000	APP/HOXD
GO:200024	11	11/791	153/20610	0.03399	0.208057	1.87328	ENSG0000	DUSP1/CIT
GO:000164	16	16/791	252/20610	0.034343	0.208057	1.654325	ENSG0000	PDLIM7/W
GO:003260	3	3/791	19/20610	0.034506	0.208057	4.114046	ENSG0000	RASGRP1/
GO:004512	7	7/791	81/20610	0.035849	0.214746	2.251721	ENSG0000	PTAFR/CC
GO:003577	4	4/791	33/20610	0.036465	0.216349	3.158258	ENSG0000	LRP1/TME
GO:006103	4	4/791	33/20610	0.036465	0.216349	3.158258	ENSG0000	WISP1/WN
GO:003030	13	13/791	194/20610	0.036868	0.218222	1.745996	ENSG0000	VEGFA/CX
GO:005196	20	20/791	338/20610	0.037528	0.221608	1.541753	ENSG0000	IDH2/APP/
GO:005166	11	11/791	156/20610	0.038318	0.223869	1.837256	ENSG0000	ITGB3/ATP
GO:010610	11	11/791	156/20610	0.038318	0.223869	1.837256	ENSG0000	VEGFA/CX
GO:012016	11	11/791	156/20610	0.038318	0.223869	1.837256	ENSG0000	VEGFA/CX
GO:004390	27	27/791	490/20610	0.038906	0.226236	1.435718	ENSG0000	JUN/CXCR
GO:000756	3	3/791	20/20610	0.039469	0.227646	3.908344	ENSG0000	PTAFR/MA
GO:003165	3	3/791	20/20610	0.039469	0.227646	3.908344	ENSG0000	ARRDC3/A
GO:003606	3	3/791	20/20610	0.039469	0.227646	3.908344	ENSG0000	FUT11/MF
GO:000998	7	7/791	83/20610	0.040134	0.229352	2.197462	ENSG0000	ALDOA/DC
GO:007149	21	21/791	362/20610	0.040297	0.230015	1.511514	ENSG0000	SLC2A1/BN
GO:007177	12	12/791	177/20610	0.040605	0.230977	1.766483	ENSG0000	FSTL3/SM/
GO:000268	15	15/791	238/20610	0.041756	0.237257	1.642161	ENSG0000	SERPINE1/

GO:006032	5	5/791	50/20610	0.042067	0.2378	2.605563	ENSG0000	TIPARP/CS
GO:006138	5	5/791	50/20610	0.042067	0.2378	2.605563	ENSG0000	HEG1/SFR
GO:005092	11	11/791	159/20610	0.043016	0.241826	1.80259	ENSG0000	SERPINE1/
GO:004275	4	4/791	35/20610	0.043966	0.244772	2.977786	ENSG0000	OXTR/OPF
GO:001025	26	26/791	474/20610	0.044038	0.244896	1.429212	ENSG0000	NDRG1/CX
GO:005080	16	16/791	261/20610	0.044887	0.2474	1.59728	ENSG0000	LZTS3/APF
GO:004814	5	5/791	51/20610	0.045227	0.247628	2.554473	ENSG0000	JUN/PDGF
GO:190353	26	26/791	476/20610	0.045914	0.250282	1.423206	ENSG0000	ABCG1/EG
GO:005195	4	4/791	36/20610	0.048017	0.258345	2.89507	ENSG0000	OXTR/PIN
GO:001087	24	24/791	436/20610	0.049772	0.262943	1.434255	ENSG0000	APOL2/ITC
GO:007099	24	24/791	436/20610	0.049772	0.262943	1.434255	ENSG0000	EGLN3/BN
GO:001063	18	18/791	307/20610	0.050153	0.264064	1.527691	ENSG0000	ITGB3/VEG
GO:009013	21	21/791	373/20610	0.052328	0.270124	1.466939	ENSG0000	LOXL2/ITG
GO:004308	2	2/791	10/20610	0.053962	0.27289	5.211125	ENSG0000	OXTR/CNF
GO:009718	2	2/791	10/20610	0.053962	0.27289	5.211125	ENSG0000	SERPINE1/
GO:190045	2	2/791	10/20610	0.053962	0.27289	5.211125	ENSG0000	KCNB1/AD
GO:000979	7	7/791	89/20610	0.054938	0.276416	2.049319	ENSG0000	VEGFA/TIF
GO:001017	5	5/791	54/20610	0.055535	0.278009	2.412558	ENSG0000	TIPARP/CS
GO:004240	3	3/791	23/20610	0.056373	0.279389	3.39856	ENSG0000	CPQ/TG/D
GO:005089	6	6/791	72/20610	0.057926	0.283967	2.171302	ENSG0000	PRKAR1B/
GO:190437	6	6/791	72/20610	0.057926	0.283967	2.171302	ENSG0000	EGFR/EPH
GO:200024	6	6/791	72/20610	0.057926	0.283967	2.171302	ENSG0000	CITED2/PT
GO:007026	9	9/791	128/20610	0.05823	0.28518	1.832036	ENSG0000	PPL/PERP/
GO:005170	16	16/791	273/20610	0.062267	0.297743	1.52707	ENSG0000	ITGB3/CXC
GO:006014	3	3/791	24/20610	0.062654	0.297743	3.256953	ENSG0000	EGFR/WTII
GO:005104	17	17/791	295/20610	0.063382	0.297938	1.501511	ENSG0000	KLF7/IDH2
GO:000676	10	10/791	150/20610	0.063675	0.297938	1.737042	ENSG0000	SLC2A3/SL
GO:000962	2	2/791	11/20610	0.064315	0.297938	4.737387	ENSG0000	PKM/PTAF
GO:001487	2	2/791	11/20610	0.064315	0.297938	4.737387	ENSG0000	PKM/SCN5
GO:002160	2	2/791	11/20610	0.064315	0.297938	4.737387	ENSG0000	PLXNA3/N
GO:005101	2	2/791	11/20610	0.064315	0.297938	4.737387	ENSG0000	GSN/VIL1
GO:004860	6	6/791	74/20610	0.064509	0.298	2.112618	ENSG0000	IGFBP5/NF
GO:004580	12	12/791	191/20610	0.064952	0.299559	1.637003	ENSG0000	SERPINE1/
GO:000191	8	8/791	112/20610	0.066531	0.304789	1.861116	ENSG0000	VAV1/RAS
GO:006117	5	5/791	57/20610	0.067086	0.306484	2.285581	ENSG0000	KLF7/LRP1
GO:003250	6	6/791	75/20610	0.067958	0.30883	2.08445	ENSG0000	SPAG4/HK
GO:000658	3	3/791	25/20610	0.069245	0.310631	3.126675	ENSG0000	CDH3/OC/
GO:004669	3	3/791	25/20610	0.069245	0.310631	3.126675	ENSG0000	STC2/STC1
GO:004244	8	8/791	113/20610	0.06932	0.310689	1.844646	ENSG0000	STC2/ADM
GO:003596	14	14/791	236/20610	0.07166	0.316603	1.545673	ENSG0000	STC2/ANK
GO:004860	12	12/791	195/20610	0.073363	0.320013	1.603423	ENSG0000	JUN/PDGF
GO:001988	6	6/791	77/20610	0.075172	0.320013	2.030309	ENSG0000	HLA-B/NA
GO:006041	6	6/791	77/20610	0.075172	0.320013	2.030309	ENSG0000	CITED2/DN
GO:000246	2	2/791	12/20610	0.075267	0.320013	4.342604	ENSG0000	SLC11A1/F
GO:001488	2	2/791	12/20610	0.075267	0.320013	4.342604	ENSG0000	NOL3/GSN
GO:004465	2	2/791	12/20610	0.075267	0.320013	4.342604	ENSG0000	ICAM1/SF
GO:006045	2	2/791	12/20610	0.075267	0.320013	4.342604	ENSG0000	OPRL1/NA
GO:004828	4	4/791	42/20610	0.076451	0.321468	2.481488	ENSG0000	IGFBP5/ER
GO:004343	12	12/791	197/20610	0.077819	0.32667	1.587145	ENSG0000	MXI1/FLN/
GO:004865	12	12/791	197/20610	0.077819	0.32667	1.587145	ENSG0000	JUN/PDGF
GO:000327	8	8/791	116/20610	0.078113	0.327625	1.79694	ENSG0000	CITED2/DN
GO:007267	9	9/791	137/20610	0.081506	0.337863	1.711683	ENSG0000	APP/PADI2
GO:006002	7	7/791	98/20610	0.082797	0.340512	1.861116	ENSG0000	TIPARP/IN
GO:007237	3	3/791	27/20610	0.083311	0.340512	2.89507	ENSG0000	F3/A2M/F1
GO:004660	8	8/791	118/20610	0.084328	0.343638	1.766483	ENSG0000	VEGFA/AX
GO:009055	7	7/791	99/20610	0.086314	0.343638	1.842317	ENSG0000	BNIP3L/BN
GO:000202	2	2/791	13/20610	0.086757	0.343638	4.008558	ENSG0000	SCTR/TRP\
GO:009020	2	2/791	13/20610	0.086757	0.343638	4.008558	ENSG0000	HEG1/WN

GO:00902	2	2/791	13/20610	0.086757	0.343638	4.008558	ENSG0000	HEG1/WN
GO:00989	2	2/791	13/20610	0.086757	0.343638	4.008558	ENSG0000	FLNA/CAV
GO:00991	2	2/791	13/20610	0.086757	0.343638	4.008558	ENSG0000	ITGB3/HP
GO:00026	10	10/791	160/2061	0.089005	0.347978	1.628477	ENSG0000	SERPINE1/
GO:00076	10	10/791	160/2061	0.089005	0.347978	1.628477	ENSG0000	JUN/HRH1
GO:19035	15	15/791	266/2061	0.089229	0.34836	1.469302	ENSG0000	KLF7/IDH2
GO:00486	12	12/791	202/2061	0.089698	0.348831	1.547859	ENSG0000	VEGFA/CX
GO:00026	19	19/791	354/2061	0.089713	0.348831	1.398466	ENSG0000	GPI/NA/PT
GO:00015	21	21/791	399/2061	0.090432	0.348831	1.371349	ENSG0000	PPFIA4/IT
GO:00015	3	3/791	28/20610	0.090766	0.348831	2.791674	ENSG0000	GBP2/SLC
GO:00360	3	3/791	28/20610	0.090766	0.348831	2.791674	ENSG0000	SCX/COL1
GO:00435	8	8/791	120/2061	0.090825	0.348831	1.737042	ENSG0000	IGFBP5/TN
GO:00140	4	4/791	45/20610	0.093218	0.355001	2.316056	ENSG0000	PPFIA4/SL
GO:00323	4	4/791	45/20610	0.093218	0.355001	2.316056	ENSG0000	STC2/ADM
GO:19035	4	4/791	45/20610	0.093218	0.355001	2.316056	ENSG0000	ADM/DOC
GO:00451	13	13/791	226/2061	0.096388	0.36127	1.498775	ENSG0000	VEGFA/FS
GO:00102	2	2/791	14/20610	0.098727	0.36127	3.722232	ENSG0000	MT1X/MT
GO:00358	2	2/791	14/20610	0.098727	0.36127	3.722232	ENSG0000	NPR1/NPF
GO:00434	2	2/791	14/20610	0.098727	0.36127	3.722232	ENSG0000	PGAM1/CI
GO:00517	2	2/791	14/20610	0.098727	0.36127	3.722232	ENSG0000	GAPDH/PF
GO:00607	2	2/791	14/20610	0.098727	0.36127	3.722232	ENSG0000	CREBRF/O
GO:00610	2	2/791	14/20610	0.098727	0.36127	3.722232	ENSG0000	JUN/EGFR
GO:00713	2	2/791	14/20610	0.098727	0.36127	3.722232	ENSG0000	SFRP1/ARI
GO:00060	6	6/791	83/20610	0.099302	0.36127	1.883539	ENSG0000	KDM3A/ID
GO:00619	6	6/791	83/20610	0.099302	0.36127	1.883539	ENSG0000	JUN/EGFR
GO:00508	23	23/791	451/2061	0.102482	0.371749	1.328779	ENSG0000	NDRG1/SD
GO:00720	6	6/791	84/20610	0.103679	0.374444	1.861116	ENSG0000	VEGFA/HC
GO:00075	5	5/791	65/20610	0.103828	0.374444	2.004279	ENSG0000	STC1/NPR
GO:00322	7	7/791	104/2061	0.105151	0.378392	1.753744	ENSG0000	FLNA/SFRI
GO:00329	21	21/791	410/2061	0.110939	0.388348	1.334556	ENSG0000	PAM/STC1
GO:00031	2	2/791	15/20610	0.111124	0.388348	3.474083	ENSG0000	EFNA1/SC
GO:00336	4	4/791	48/20610	0.111564	0.389064	2.171302	ENSG0000	SERPINE1/
GO:00459	16	16/791	298/2061	0.111849	0.389784	1.39896	ENSG0000	VEGFA/CX
GO:00083	10	10/791	168/2061	0.113074	0.391141	1.55093	ENSG0000	VEGFA/RH
GO:00007	5	5/791	67/20610	0.114302	0.391141	1.94445	ENSG0000	MYOF/AD
GO:00064	3	3/791	31/20610	0.11468	0.391141	2.521512	ENSG0000	KDM3A/KI
GO:00106	3	3/791	31/20610	0.11468	0.391141	2.521512	ENSG0000	ATP1B1/C
GO:00716	8	8/791	127/2061	0.115759	0.39401	1.641299	ENSG0000	SERPINE1/
GO:00069	4	4/791	49/20610	0.11801	0.400573	2.12699	ENSG0000	INSIG2/BC
GO:00321	20	20/791	391/2061	0.11862	0.402369	1.332769	ENSG0000	SERPINE1/
GO:00309	8	8/791	128/2061	0.119594	0.404997	1.628477	ENSG0000	STC2/PPP
GO:00466	18	18/791	347/2061	0.121595	0.407394	1.351589	ENSG0000	BNIP3/JUN
GO:00510	17	17/791	325/2061	0.122891	0.407394	1.36291	ENSG0000	VEGFA/AP
GO:00190	3	3/791	32/20610	0.123128	0.407394	2.442715	ENSG0000	APP/CREB
GO:00445	3	3/791	32/20610	0.123128	0.407394	2.442715	ENSG0000	CDH3/OC
GO:00610	3	3/791	32/20610	0.123128	0.407394	2.442715	ENSG0000	LOXL2/SC
GO:00069	2	2/791	16/20610	0.123898	0.407394	3.256953	ENSG0000	SCARB1/P
GO:00975	2	2/791	16/20610	0.123898	0.407394	3.256953	ENSG0000	MT1X/MT
GO:00071	4	4/791	50/20610	0.124614	0.407394	2.08445	ENSG0000	MEIOB/NA
GO:00069	5	5/791	69/20610	0.125259	0.408964	1.888089	ENSG0000	MYOF/AD
GO:00326	5	5/791	69/20610	0.125259	0.408964	1.888089	ENSG0000	HOMER3/
GO:00163	14	14/791	259/2061	0.125519	0.409545	1.408412	ENSG0000	KLF7/SDK1
GO:00481	6	6/791	89/20610	0.127027	0.412566	1.756559	ENSG0000	JUN/PDGF
GO:00487	11	11/791	194/2061	0.127871	0.414767	1.477381	ENSG0000	PAM/CRAI
GO:00104	3	3/791	33/20610	0.131795	0.423072	2.368693	ENSG0000	VEGFA/WI
GO:00097	6	6/791	90/20610	0.131979	0.423116	1.737042	ENSG0000	STC1/CITE
GO:00065	12	12/791	218/2061	0.134787	0.431109	1.434255	ENSG0000	EGLN3/PL
GO:00019	7	7/791	111/2061	0.134906	0.431109	1.643148	ENSG0000	HLA-B/NA

GO:00517(	2	2/791	17/20610	0.137002	0.431969	3.065368	ENSG0000	GAPDH/PI
GO:00343(	3	3/791	34/20610	0.140667	0.438513	2.299026	ENSG0000	ABCG1/AE
GO:00029:	4	4/791	53/20610	0.14532	0.449072	1.966462	ENSG0000	HK2/NOL3
GO:00301!	4	4/791	53/20610	0.14532	0.449072	1.966462	ENSG0000	SERPINE1/
GO:00316(	15	15/791	289/2061C	0.146886	0.453067	1.352368	ENSG0000	SLC2A1/JU
GO:00301(	10	10/791	178/2061C	0.147795	0.454836	1.463799	ENSG0000	ITGB3/PD(
GO:00098!	23	23/791	474/2061C	0.148893	0.454836	1.264303	ENSG0000	PFKFB4/BI
GO:00513!	3	3/791	35/20610	0.149732	0.454836	2.233339	ENSG0000	CAV1/CNF
GO:00989(	3	3/791	35/20610	0.149732	0.454836	2.233339	ENSG0000	FLNA/CAV
GO:00300:	2	2/791	18/20610	0.150391	0.454836	2.89507	ENSG0000	WNT11/AI
GO:00316!	2	2/791	18/20610	0.150391	0.454836	2.89507	ENSG0000	APLN/CNF
GO:00485:	2	2/791	18/20610	0.150391	0.454836	2.89507	ENSG0000	PLXNA3/N
GO:00105:	4	4/791	54/20610	0.152503	0.458957	1.930046	ENSG0000	CAV1/JPH:
GO:00329(	4	4/791	54/20610	0.152503	0.458957	1.930046	ENSG0000	COL5A1/E
GO:19000(	4	4/791	54/20610	0.152503	0.458957	1.930046	ENSG0000	SERPINE1/
GO:00516!	6	6/791	94/20610	0.152678	0.458957	1.663125	ENSG0000	SPAG4/HK
GO:00468{	8	8/791	137/2061C	0.157033	0.470626	1.521496	ENSG0000	EGFR/INH/
GO:00226(	7	7/791	116/2061C	0.158424	0.474169	1.572322	ENSG0000	SCARB1/C
GO:00018!	3	3/791	36/20610	0.158979	0.474169	2.171302	ENSG0000	STC2/STC:
GO:00164{	3	3/791	36/20610	0.158979	0.474169	2.171302	ENSG0000	BACE2/CP
GO:00970(	3	3/791	36/20610	0.158979	0.474169	2.171302	ENSG0000	EDN2/AMI
GO:00085(	4	4/791	55/20610	0.159817	0.47581	1.894955	ENSG0000	HRH1/APF
GO:00108{	4	4/791	55/20610	0.159817	0.47581	1.894955	ENSG0000	ITGB3/AB(
GO:00458(	5	5/791	75/20610	0.160821	0.477939	1.737042	ENSG0000	CAV1/ANK
GO:00607(	5	5/791	75/20610	0.160821	0.477939	1.737042	ENSG0000	CXCR4/AX
GO:00425!	11	11/791	204/2061C	0.162437	0.482166	1.40496	ENSG0000	ADM/SLC2
GO:00364	6	6/791	96/20610	0.163536	0.483697	1.628477	ENSG0000	PDK1/NOL
GO:19059:	2	2/791	19/20610	0.164024	0.483697	2.742697	ENSG0000	CITED2/GI
GO:00095{	8	8/791	139/2061C	0.166032	0.489321	1.499604	ENSG0000	CXCR4/CC
GO:00970:	4	4/791	56/20610	0.167257	0.489607	1.861116	ENSG0000	ANO6/ATI
GO:00434:	15	15/791	296/2061C	0.167734	0.489607	1.320386	ENSG0000	GAL3ST1/I
GO:00486(	7	7/791	118/2061C	0.168322	0.489607	1.545673	ENSG0000	STC2/SEM
GO:00509:	5	5/791	77/20610	0.173495	0.499879	1.691924	ENSG0000	DUSP1/PA
GO:00457	6	6/791	98/20610	0.174714	0.499879	1.595242	ENSG0000	PDLIM7/W
GO:00312!	4	4/791	57/20610	0.174817	0.499879	1.828465	ENSG0000	CAV1/VAV
GO:00508:	4	4/791	57/20610	0.174817	0.499879	1.828465	ENSG0000	SERPINE1/
GO:00466(	9	9/791	163/2061C	0.175627	0.499879	1.438654	ENSG0000	FSTL3/INH
GO:00327!	2	2/791	20/20610	0.177864	0.499879	2.605563	ENSG0000	VEGFA/AD
GO:00430(	2	2/791	20/20610	0.177864	0.499879	2.605563	ENSG0000	MOV10L1/
GO:19000	3	3/791	38/20610	0.177968	0.499879	2.057023	ENSG0000	GRB10/PTI
GO:00095{	8	8/791	142/2061C	0.179961	0.504396	1.467923	ENSG0000	CXCR4/CC
GO:00075(	14	14/791	277/2061C	0.180323	0.505045	1.316891	ENSG0000	VEGFA/FS
GO:00026{	4	4/791	58/20610	0.182491	0.508844	1.79694	ENSG0000	DUSP1/PA
GO:00439(	11	11/791	210/2061C	0.18519	0.515502	1.364818	ENSG0000	JUN/NR5A
GO:00092(	14	14/791	279/2061C	0.187049	0.518378	1.307451	ENSG0000	ADM/CXC
GO:00513(	7	7/791	122/2061C	0.188899	0.521441	1.494995	ENSG0000	EGFR/CAV
GO:19004(	4	4/791	59/20610	0.190275	0.523828	1.766483	ENSG0000	NOL3/PIN
GO:00358:	2	2/791	21/20610	0.191873	0.523828	2.481488	ENSG0000	NPR1/NPF
GO:00467:	2	2/791	21/20610	0.191873	0.523828	2.481488	ENSG0000	ALDOA/L(
GO:00550!	2	2/791	21/20610	0.191873	0.523828	2.481488	ENSG0000	BNIP3/CA
GO:20000:	3	3/791	40/20610	0.197535	0.535692	1.954172	ENSG0000	LOXL2/LBI
GO:00486(	7	7/791	125/2061C	0.204968	0.544362	1.459115	ENSG0000	JUN/PDGF
GO:00218:	2	2/791	22/20610	0.20602	0.544362	2.368693	ENSG0000	C16orf45/I
GO:00361:	2	2/791	22/20610	0.20602	0.544362	2.368693	ENSG0000	ERRFI1/HA
GO:01401:	2	2/791	22/20610	0.20602	0.544362	2.368693	ENSG0000	PRKN/CD3
GO:00486(	4	4/791	61/20610	0.206144	0.544362	1.708566	ENSG0000	CITED2/FC
GO:19028{	4	4/791	61/20610	0.206144	0.544362	1.708566	ENSG0000	NOL3/PIN
GO:00901{	3	3/791	41/20610	0.207507	0.544362	1.906509	ENSG0000	VEGFA/PD

GO:190189	3	3/791	41/20610	0.207507	0.544362	1.906509	ENSG0000	VEGFA/CA
GO:000975	13	13/791	262/20610	0.20881	0.546914	1.292836	ENSG0000	LBH/KDM3
GO:001052	6	6/791	104/20610	0.209991	0.549428	1.503209	ENSG0000	CAV1/JPH
GO:000208	5	5/791	83/20610	0.213619	0.557159	1.569616	ENSG0000	VIM/BFSP1
GO:000926	3	3/791	42/20610	0.217588	0.56327	1.861116	ENSG0000	PAM/PCK1
GO:190138	3	3/791	42/20610	0.217588	0.56327	1.861116	ENSG0000	ATP1B1/A
GO:000753	2	2/791	23/20610	0.220272	0.56327	2.265707	ENSG0000	CITED2/GN
GO:002151	2	2/791	23/20610	0.220272	0.56327	2.265707	ENSG0000	MDGA1/W
GO:004393	2	2/791	23/20610	0.220272	0.56327	2.265707	ENSG0000	ANO6/FAT
GO:000202	6	6/791	106/20610	0.222272	0.567217	1.474847	ENSG0000	ADM/CAV
GO:000237	7	7/791	129/20610	0.22716	0.577973	1.413871	ENSG0000	GPI/NA/PA
GO:190595	5	5/791	85/20610	0.227603	0.577973	1.532684	ENSG0000	ABCG1/HI
GO:004512	3	3/791	43/20610	0.227768	0.577973	1.817834	ENSG0000	ITGB3/EGF
GO:000283	4	4/791	64/20610	0.230619	0.583717	1.628477	ENSG0000	HTRA1/AR
GO:004614	4	4/791	64/20610	0.230619	0.583717	1.628477	ENSG0000	MTHFD1L/
GO:190495	12	12/791	245/20610	0.233378	0.585603	1.276194	ENSG0000	KLF7/IDH2
GO:001088	2	2/791	24/20610	0.234601	0.585603	2.171302	ENSG0000	HILPDA/SO
GO:004299	2	2/791	24/20610	0.234601	0.585603	2.171302	ENSG0000	MXI1/MDF
GO:190437	2	2/791	24/20610	0.234601	0.585603	2.171302	ENSG0000	PID1/LYPD
GO:005080	5	5/791	86/20610	0.234694	0.585603	1.514862	ENSG0000	SORCS2/K
GO:000690	3	3/791	44/20610	0.238035	0.591041	1.77652	ENSG0000	SYTL2/UN
GO:003223	4	4/791	65/20610	0.238933	0.592656	1.603423	ENSG0000	FLNA/SFRI
GO:003134	11	11/791	223/20610	0.239043	0.592656	1.285255	ENSG0000	GAPDH/VA
GO:190188	6	6/791	109/20610	0.24112	0.596909	1.434255	ENSG0000	VEGFA/CA
GO:003803	5	5/791	87/20610	0.241846	0.597814	1.49745	ENSG0000	DAPK3/BC
GO:006075	11	11/791	224/20610	0.243415	0.601074	1.279517	ENSG0000	CXCR4/AX
GO:000761	7	7/791	132/20610	0.24432	0.60243	1.381738	ENSG0000	HRH1/VLD
GO:003052	15	15/791	319/20610	0.245791	0.603138	1.225186	ENSG0000	LBH/KDM3
GO:000194	2	2/791	25/20610	0.24898	0.603138	2.08445	ENSG0000	VEGFA/HE
GO:000205	2	2/791	25/20610	0.24898	0.603138	2.08445	ENSG0000	VEGFA/CH
GO:001052	2	2/791	25/20610	0.24898	0.603138	2.08445	ENSG0000	AICDA/PIW
GO:001052	2	2/791	25/20610	0.24898	0.603138	2.08445	ENSG0000	AICDA/PIW
GO:009008	2	2/791	25/20610	0.24898	0.603138	2.08445	ENSG0000	DNAJB2/H
GO:190370	9	9/791	179/20610	0.250583	0.606429	1.310059	ENSG0000	FSTL3/INH
GO:009716	11	11/791	226/20610	0.252244	0.610153	1.268194	ENSG0000	DEGS2/NP
GO:000243	4	4/791	67/20610	0.255761	0.616558	1.55556	ENSG0000	RASGRP1/
GO:007177	8	8/791	158/20610	0.261749	0.621846	1.319272	ENSG0000	FAT4/SFRF
GO:000191	2	2/791	26/20610	0.263384	0.621846	2.004279	ENSG0000	OPTN/ACH
GO:001088	2	2/791	26/20610	0.263384	0.621846	2.004279	ENSG0000	ITGB3/ABC
GO:003462	2	2/791	26/20610	0.263384	0.621846	2.004279	ENSG0000	SMAD7/NA
GO:005187	2	2/791	26/20610	0.263384	0.621846	2.004279	ENSG0000	CDH3/RAE
GO:006034	2	2/791	26/20610	0.263384	0.621846	2.004279	ENSG0000	SFRP1/WN
GO:006074	2	2/791	26/20610	0.263384	0.621846	2.004279	ENSG0000	SFRP1/NO
GO:005160	13	13/791	276/20610	0.263715	0.621846	1.227258	ENSG0000	BNIP3L/BN
GO:004001	4	4/791	68/20610	0.264263	0.622253	1.532684	ENSG0000	STC2/APP/
GO:003238	11	11/791	229/20610	0.265694	0.625325	1.25158	ENSG0000	NEDD4/FL
GO:000761	3	3/791	47/20610	0.269256	0.629235	1.663125	ENSG0000	APP/OXTR
GO:007084	3	3/791	47/20610	0.269256	0.629235	1.663125	ENSG0000	EGFR/ERRI
GO:000189	5	5/791	91/20610	0.270995	0.632703	1.431628	ENSG0000	ADM/PDG
GO:004390	13	13/791	278/20610	0.271949	0.634038	1.218429	ENSG0000	JUN/CXCR
GO:003210	19	19/791	423/20610	0.27278	0.635675	1.170347	ENSG0000	SERPINE1/
GO:005130	2	2/791	27/20610	0.277789	0.642525	1.930046	ENSG0000	MEIOB/NA
GO:003236	3	3/791	48/20610	0.279769	0.6456	1.628477	ENSG0000	ITGB3/PLA
GO:004851	3	3/791	48/20610	0.279769	0.6456	1.628477	ENSG0000	CIART/OPI
GO:000738	21	21/791	474/20610	0.280046	0.645782	1.154363	ENSG0000	BARX1/STO
GO:007026	4	4/791	70/20610	0.281418	0.6473	1.488893	ENSG0000	BNIP3/NO
GO:009907	4	4/791	70/20610	0.281418	0.6473	1.488893	ENSG0000	ITGB3/CAC
GO:190595	4	4/791	70/20610	0.281418	0.6473	1.488893	ENSG0000	ITGB3/ABC

GO:00326:	4	4/791	71/20610	0.290059	0.662229	1.467923	ENSG0000	TLR9/TRIB
GO:00603!	4	4/791	71/20610	0.290059	0.662229	1.467923	ENSG0000	JUN/INHA
GO:00067:	2	2/791	28/20610	0.292176	0.662229	1.861116	ENSG0000	SFXN3/MT
GO:00107:	2	2/791	28/20610	0.292176	0.662229	1.861116	ENSG0000	VIM/SCX
GO:00218!	2	2/791	28/20610	0.292176	0.662229	1.861116	ENSG0000	NRP2/RAX
GO:00605:	2	2/791	28/20610	0.292176	0.662229	1.861116	ENSG0000	SFRP1/NO
GO:00971{	2	2/791	28/20610	0.292176	0.662229	1.861116	ENSG0000	PERP/ITGB
GO:00094(	10	10/791	212/2061C	0.297675	0.669682	1.229039	ENSG0000	TGFB1I1/N
GO:00517(	5	5/791	95/20610	0.30084	0.669682	1.371349	ENSG0000	JUN/GAPD
GO:00032!	3	3/791	50/20610	0.300895	0.669682	1.563338	ENSG0000	SMAD7/B(
GO:00094(	3	3/791	50/20610	0.300895	0.669682	1.563338	ENSG0000	ADM/PCSI
GO:00107:	3	3/791	50/20610	0.300895	0.669682	1.563338	ENSG0000	ERRFI1/VIN
GO:00215:	3	3/791	50/20610	0.300895	0.669682	1.563338	ENSG0000	VLDLR/HC
GO:00991:	3	3/791	50/20610	0.300895	0.669682	1.563338	ENSG0000	APP/MDG/
GO:00481(	10	10/791	213/2061C	0.30263	0.669682	1.223269	ENSG0000	SORCS2/H
GO:00224(	15	15/791	334/2061C	0.303117	0.669682	1.170163	ENSG0000	FSTL3/CITI
GO:00363:	2	2/791	29/20610	0.306523	0.669682	1.79694	ENSG0000	CXCR4/AN
GO:00468	5	5/791	96/20610	0.308384	0.669682	1.357064	ENSG0000	ITGB3/EGF
GO:19013:	5	5/791	96/20610	0.308384	0.669682	1.357064	ENSG0000	ATP1B1/A
GO:00512:	11	11/791	239/2061C	0.312063	0.669682	1.199213	ENSG0000	KLF7/IDH2
GO:00352(	10	10/791	215/2061C	0.3126	0.669682	1.21189	ENSG0000	COL12A1/
GO:00510!	18	18/791	410/2061C	0.312657	0.669682	1.143906	ENSG0000	JUN/PDGF
GO:19030:	5	5/791	97/20610	0.315954	0.669682	1.343073	ENSG0000	SERPINE1/
GO:00192:	4	4/791	74/20610	0.316175	0.669682	1.408412	ENSG0000	CACNG4/I
GO:19000:	4	4/791	74/20610	0.316175	0.669682	1.408412	ENSG0000	GRB10/PTI
GO:00030:	2	2/791	30/20610	0.320813	0.669682	1.737042	ENSG0000	GLS/ATP1/
GO:00801!	2	2/791	30/20610	0.320813	0.669682	1.737042	ENSG0000	NOX5/PRS
GO:19038	2	2/791	30/20610	0.320813	0.669682	1.737042	ENSG0000	TGFB1I1/C
GO:20010:	5	5/791	98/20610	0.323548	0.669682	1.329369	ENSG0000	EGFR/PLA:
GO:00031	1	1/791	10/20610	0.323918	0.669682	2.605563	ENSG0000	NOG
GO:00148:	1	1/791	10/20610	0.323918	0.669682	2.605563	ENSG0000	SCN5A
GO:00309:	1	1/791	10/20610	0.323918	0.669682	2.605563	ENSG0000	WNT1
GO:00313	1	1/791	10/20610	0.323918	0.669682	2.605563	ENSG0000	TMEM27
GO:00326:	1	1/791	10/20610	0.323918	0.669682	2.605563	ENSG0000	TLR9
GO:00344	1	1/791	10/20610	0.323918	0.669682	2.605563	ENSG0000	VLDLR
GO:00358:	1	1/791	10/20610	0.323918	0.669682	2.605563	ENSG0000	ITGB4
GO:00427!	1	1/791	10/20610	0.323918	0.669682	2.605563	ENSG0000	APLN
GO:00448(	1	1/791	10/20610	0.323918	0.669682	2.605563	ENSG0000	CXCR4
GO:00613(	1	1/791	10/20610	0.323918	0.669682	2.605563	ENSG0000	WNT9A
GO:00703	1	1/791	10/20610	0.323918	0.669682	2.605563	ENSG0000	PID1
GO:00718(	1	1/791	10/20610	0.323918	0.669682	2.605563	ENSG0000	FLT3LG
GO:00907:	1	1/791	10/20610	0.323918	0.669682	2.605563	ENSG0000	FGL2
GO:19024!	1	1/791	10/20610	0.323918	0.669682	2.605563	ENSG0000	LOXL2
GO:19033:	1	1/791	10/20610	0.323918	0.669682	2.605563	ENSG0000	DNAJB2
GO:00717(	10	10/791	218/2061C	0.327693	0.67636	1.195212	ENSG0000	AXL/APP/I
GO:00075(	8	8/791	170/2061C	0.329113	0.678179	1.226147	ENSG0000	SERPINE1/
GO:00328{	11	11/791	243/2061C	0.331146	0.678179	1.179473	ENSG0000	DNAH11/I
GO:00309(	5	5/791	99/20610	0.331161	0.678179	1.315941	ENSG0000	SYNGR3/K
GO:00341(	2	2/791	31/20610	0.335029	0.678179	1.681008	ENSG0000	SFRP1/IAP
GO:00506{	2	2/791	31/20610	0.335029	0.678179	1.681008	ENSG0000	HTRA1/FG
GO:19054:	2	2/791	31/20610	0.335029	0.678179	1.681008	ENSG0000	PID1/LYPD
GO:19059!	8	8/791	172/2061C	0.340648	0.678179	1.21189	ENSG0000	ITGB3/AB(
GO:00519(	4	4/791	77/20610	0.342473	0.678179	1.353539	ENSG0000	EPHB2/OX
GO:00017(	3	3/791	54/20610	0.343302	0.678179	1.447535	ENSG0000	DSCAML1/
GO:19035:	3	3/791	54/20610	0.343302	0.678179	1.447535	ENSG0000	PRKN/BFA
GO:00519!	2	2/791	32/20610	0.349156	0.678179	1.628477	ENSG0000	ADRA2C/C
GO:00021!	1	1/791	11/20610	0.349878	0.678179	2.368693	ENSG0000	FLT3LG
GO:00319{	1	1/791	11/20610	0.349878	0.678179	2.368693	ENSG0000	ARRDC3

GO:003235	1	1/791	11/20610	0.349878	0.678179	2.368693	ENSG0000	DKK3
GO:004001	1	1/791	11/20610	0.349878	0.678179	2.368693	ENSG0000	STC2
GO:005112	1	1/791	11/20610	0.349878	0.678179	2.368693	ENSG0000	APP
GO:005191	1	1/791	11/20610	0.349878	0.678179	2.368693	ENSG0000	SERPINE1
GO:006035	1	1/791	11/20610	0.349878	0.678179	2.368693	ENSG0000	CAV1
GO:007025	1	1/791	11/20610	0.349878	0.678179	2.368693	ENSG0000	SYTL2
GO:007211	1	1/791	11/20610	0.349878	0.678179	2.368693	ENSG0000	PDGFB
GO:190594	1	1/791	11/20610	0.349878	0.678179	2.368693	ENSG0000	CITED2
GO:000956	9	9/791	198/20610	0.350275	0.678683	1.184347	ENSG0000	HOXD10/A
GO:004516	13	13/791	297/20610	0.353728	0.683139	1.140482	ENSG0000	TGFB1I1/H
GO:200102	5	5/791	103/20610	0.361748	0.688966	1.264836	ENSG0000	SYT12/PIN
GO:006067	2	2/791	33/20610	0.363179	0.688966	1.579129	ENSG0000	JUNB/ITGE
GO:200102	2	2/791	33/20610	0.363179	0.688966	1.579129	ENSG0000	PINK1/CN
GO:000961	16	16/791	374/20610	0.364087	0.688966	1.114679	ENSG0000	BNIP3L/BN
GO:199008	3	3/791	56/20610	0.364452	0.688966	1.395837	ENSG0000	APP/NTF4
GO:003526	7	7/791	152/20610	0.36633	0.688966	1.19993	ENSG0000	STC2/APP/
GO:004208	7	7/791	152/20610	0.36633	0.688966	1.19993	ENSG0000	INHA/APP
GO:004545	4	4/791	80/20610	0.368823	0.688966	1.302781	ENSG0000	QSOX1/N(
GO:005079	4	4/791	80/20610	0.368823	0.688966	1.302781	ENSG0000	ARRDC3/C
GO:007169	8	8/791	177/20610	0.36971	0.688966	1.177655	ENSG0000	ANO6/APF
GO:003027	10	10/791	227/20610	0.373716	0.688966	1.147825	ENSG0000	PDLIM7/W
GO:000699	1	1/791	12/20610	0.374843	0.688966	2.171302	ENSG0000	INSIG2
GO:000732	1	1/791	12/20610	0.374843	0.688966	2.171302	ENSG0000	OXTR
GO:000829	1	1/791	12/20610	0.374843	0.688966	2.171302	ENSG0000	ACHE
GO:000988	1	1/791	12/20610	0.374843	0.688966	2.171302	ENSG0000	FBN1
GO:001062	1	1/791	12/20610	0.374843	0.688966	2.171302	ENSG0000	NTRK1
GO:001618	1	1/791	12/20610	0.374843	0.688966	2.171302	ENSG0000	UNC13C
GO:004369	1	1/791	12/20610	0.374843	0.688966	2.171302	ENSG0000	PDGFB
GO:006067	1	1/791	12/20610	0.374843	0.688966	2.171302	ENSG0000	ADM
GO:006145	1	1/791	12/20610	0.374843	0.688966	2.171302	ENSG0000	SYDE1
GO:190061	1	1/791	12/20610	0.374843	0.688966	2.171302	ENSG0000	ACHE
GO:190116	1	1/791	12/20610	0.374843	0.688966	2.171302	ENSG0000	SYDE1
GO:000300	16	16/791	377/20610	0.376068	0.688966	1.105809	ENSG0000	BARX1/CIT
GO:004826	2	2/791	34/20610	0.377086	0.688966	1.532684	ENSG0000	CACNA1B/
GO:008601	2	2/791	34/20610	0.377086	0.688966	1.532684	ENSG0000	FLNA/SCN
GO:000191	9	9/791	203/20610	0.377541	0.68914	1.155176	ENSG0000	VAV1/RAS
GO:001598	13	13/791	304/20610	0.384963	0.69718	1.114221	ENSG0000	BNIP3/PPF
GO:000257	2	2/791	35/20610	0.390865	0.69718	1.488893	ENSG0000	SLC11A1/F
GO:000714	2	2/791	35/20610	0.390865	0.69718	1.488893	ENSG0000	MEIOB/NA
GO:004263	2	2/791	35/20610	0.390865	0.69718	1.488893	ENSG0000	CDH3/WN
GO:009917	8	8/791	181/20610	0.393103	0.69718	1.15163	ENSG0000	NDRG1/LZ
GO:000267	1	1/791	13/20610	0.39885	0.69718	2.004279	ENSG0000	PRDX2
GO:001402	1	1/791	13/20610	0.39885	0.69718	2.004279	ENSG0000	SFRP1
GO:004575	1	1/791	13/20610	0.39885	0.69718	2.004279	ENSG0000	CNR1
GO:004576	1	1/791	13/20610	0.39885	0.69718	2.004279	ENSG0000	SCN5A
GO:007057	1	1/791	13/20610	0.39885	0.69718	2.004279	ENSG0000	LRP1
GO:200000	1	1/791	13/20610	0.39885	0.69718	2.004279	ENSG0000	SNX33
GO:200034	1	1/791	13/20610	0.39885	0.69718	2.004279	ENSG0000	PRSS37
GO:004883	9	9/791	207/20610	0.399472	0.697777	1.132853	ENSG0000	INSIG2/CC
GO:002153	4	4/791	84/20610	0.403833	0.701143	1.240744	ENSG0000	NRP2/WN
GO:001025	2	2/791	36/20610	0.404506	0.701143	1.447535	ENSG0000	CRYAB/RN
GO:003289	2	2/791	36/20610	0.404506	0.701143	1.447535	ENSG0000	PLA2R1/KN
GO:004209	2	2/791	36/20610	0.404506	0.701143	1.447535	ENSG0000	ARG2/HLX
GO:190533	2	2/791	36/20610	0.404506	0.701143	1.447535	ENSG0000	VEGFA/NC
GO:005088	3	3/791	60/20610	0.406354	0.70312	1.302781	ENSG0000	TNNT1/ITF
GO:003260	5	5/791	110/20610	0.415321	0.703132	1.184347	ENSG0000	APP/LRP1/
GO:005128	6	6/791	135/20610	0.416492	0.703132	1.158028	ENSG0000	NOL3/JPH
GO:007121	12	12/791	286/20610	0.417805	0.703132	1.093243	ENSG0000	SERPINE1/

GO:00018[illegible]	2	2/791	37/20610	0.417998	0.703132	1.408412	ENSG0000	OPTN/ACI
GO:00430[illegible]	2	2/791	37/20610	0.417998	0.703132	1.408412	ENSG0000	AMPD3/CI
GO:00509[illegible]	2	2/791	37/20610	0.417998	0.703132	1.408412	ENSG0000	OCA2/NA
GO:00975[illegible]	2	2/791	37/20610	0.417998	0.703132	1.408412	ENSG0000	DPY19L2P
GO:00439[illegible]	10	10/791	236/2061[illegible]	0.42032	0.703132	1.104052	ENSG0000	JUN/GSN/
GO:00302[illegible]	1	1/791	14/20610	0.421936	0.703132	1.861116	ENSG0000	B4GALNT2
GO:00433[illegible]	1	1/791	14/20610	0.421936	0.703132	1.861116	ENSG0000	DOCK2
GO:00603[illegible]	1	1/791	14/20610	0.421936	0.703132	1.861116	ENSG0000	CAV1
GO:00906[illegible]	1	1/791	14/20610	0.421936	0.703132	1.861116	ENSG0000	PDGFB
GO:00907[illegible]	1	1/791	14/20610	0.421936	0.703132	1.861116	ENSG0000	FGL2
GO:19017[illegible]	1	1/791	14/20610	0.421936	0.703132	1.861116	ENSG0000	PDGFB
GO:00320[illegible]	5	5/791	111/2061[illegible]	0.422933	0.703141	1.173677	ENSG0000	TLR9/IRAK
GO:00351[illegible]	7	7/791	161/2061[illegible]	0.423074	0.703141	1.132853	ENSG0000	CRABP2/H
GO:00313[illegible]	13	13/791	313/2061[illegible]	0.425483	0.706672	1.082183	ENSG0000	VEGFA/JU
GO:00350[illegible]	3	3/791	62/20610	0.427006	0.708017	1.260756	ENSG0000	ALDOA/C
GO:00513[illegible]	3	3/791	62/20610	0.427006	0.708017	1.260756	ENSG0000	EDN2/SCA
GO:00607[illegible]	4	4/791	87/20610	0.429855	0.711391	1.19796	ENSG0000	PXDN/NO
GO:00512[illegible]	6	6/791	137/2061[illegible]	0.430206	0.711391	1.141122	ENSG0000	NOL3/JPH
GO:00619[illegible]	6	6/791	137/2061[illegible]	0.430206	0.711391	1.141122	ENSG0000	C11orf80/
GO:00018[illegible]	15	15/791	365/2061[illegible]	0.430436	0.711391	1.070779	ENSG0000	AXL/INHA
GO:00903[illegible]	5	5/791	112/2061[illegible]	0.430528	0.711391	1.163198	ENSG0000	NEK6/YPE
GO:00344[illegible]	2	2/791	38/20610	0.431333	0.711391	1.371349	ENSG0000	CITED2/SN
GO:00510[illegible]	8	8/791	188/2061[illegible]	0.434072	0.713215	1.10875	ENSG0000	APP/CAV1
GO:00073[illegible]	7	7/791	163/2061[illegible]	0.435647	0.713215	1.118953	ENSG0000	HOXD10/
GO:00068[illegible]	6	6/791	138/2061[illegible]	0.437045	0.713215	1.132853	ENSG0000	GSTM3/ST
GO:00071[illegible]	3	3/791	63/20610	0.437237	0.713215	1.240744	ENSG0000	C11orf80/
GO:00323[illegible]	3	3/791	63/20610	0.437237	0.713215	1.240744	ENSG0000	ABCG1/LR
GO:00329[illegible]	3	3/791	63/20610	0.437237	0.713215	1.240744	ENSG0000	BHLHE40/
GO:00512[illegible]	20	20/791	495/2061[illegible]	0.439128	0.713215	1.052753	ENSG0000	ABCG1/EG
GO:00024[illegible]	1	1/791	15/20610	0.444137	0.713215	1.737042	ENSG0000	UNC13D
GO:00025[illegible]	1	1/791	15/20610	0.444137	0.713215	1.737042	ENSG0000	AICDA
GO:00097[illegible]	1	1/791	15/20610	0.444137	0.713215	1.737042	ENSG0000	ADCY8
GO:00302[illegible]	1	1/791	15/20610	0.444137	0.713215	1.737042	ENSG0000	GNRH1
GO:00480[illegible]	1	1/791	15/20610	0.444137	0.713215	1.737042	ENSG0000	SPNS2
GO:00602[illegible]	1	1/791	15/20610	0.444137	0.713215	1.737042	ENSG0000	IDH2
GO:00602[illegible]	1	1/791	15/20610	0.444137	0.713215	1.737042	ENSG0000	JCHAIN
GO:19010[illegible]	1	1/791	15/20610	0.444137	0.713215	1.737042	ENSG0000	BOK
GO:19055[illegible]	1	1/791	15/20610	0.444137	0.713215	1.737042	ENSG0000	PRSS37
GO:00015[illegible]	2	2/791	39/20610	0.444503	0.713215	1.336186	ENSG0000	DNAH11/
GO:00069[illegible]	2	2/791	39/20610	0.444503	0.713215	1.336186	ENSG0000	BOK/GPER
GO:00326[illegible]	2	2/791	39/20610	0.444503	0.713215	1.336186	ENSG0000	ARG2/SPH
GO:00484[illegible]	8	8/791	190/2061[illegible]	0.445736	0.714962	1.097079	ENSG0000	VEGFA/AX
GO:00075[illegible]	10	10/791	241/2061[illegible]	0.446216	0.715502	1.081146	ENSG0000	STC2/STC
GO:00031[illegible]	3	3/791	64/20610	0.447399	0.715781	1.221357	ENSG0000	EFNA1/SC
GO:00512[illegible]	6	6/791	140/2061[illegible]	0.450675	0.720789	1.11667	ENSG0000	NOL3/JPH
GO:00098[illegible]	3	3/791	65/20610	0.457486	0.724868	1.202567	ENSG0000	NRP2/WN
GO:00147[illegible]	2	2/791	40/20610	0.457501	0.724868	1.302781	ENSG0000	IGFBP5/ER
GO:20006[illegible]	2	2/791	40/20610	0.457501	0.724868	1.302781	ENSG0000	VEGFA/TH
GO:00508[illegible]	4	4/791	91/20610	0.464052	0.724868	1.145302	ENSG0000	INHA/EDN
GO:00517[illegible]	4	4/791	91/20610	0.464052	0.724868	1.145302	ENSG0000	VEGFA/LB
GO:00017[illegible]	1	1/791	16/20610	0.465486	0.724868	1.628477	ENSG0000	AXL
GO:00220[illegible]	1	1/791	16/20610	0.465486	0.724868	1.628477	ENSG0000	EPHB2
GO:00309[illegible]	1	1/791	16/20610	0.465486	0.724868	1.628477	ENSG0000	RHOB
GO:00400[illegible]	1	1/791	16/20610	0.465486	0.724868	1.628477	ENSG0000	GDPD5
GO:00424[illegible]	1	1/791	16/20610	0.465486	0.724868	1.628477	ENSG0000	ACHE
GO:00514[illegible]	1	1/791	16/20610	0.465486	0.724868	1.628477	ENSG0000	DUSP1
GO:00906[illegible]	1	1/791	16/20610	0.465486	0.724868	1.628477	ENSG0000	APP
GO:19002[illegible]	1	1/791	16/20610	0.465486	0.724868	1.628477	ENSG0000	PDGFB

GO:190218	1	1/791	16/20610	0.465486	0.724868	1.628477	ENSG0000	PC
GO:190354	1	1/791	16/20610	0.465486	0.724868	1.628477	ENSG0000	PRKN
GO:003582	8	8/791	194/20610	0.468945	0.728741	1.074459	ENSG0000	JUN/APOL
GO:004218	11	11/791	272/20610	0.473133	0.732869	1.05372	ENSG0000	ADM/PDK
GO:000194	4	4/791	93/20610	0.480879	0.736964	1.120672	ENSG0000	EGFR/IGFB
GO:005068	4	4/791	93/20610	0.480879	0.736964	1.120672	ENSG0000	HTRA1/DD
GO:005130	4	4/791	93/20610	0.480879	0.736964	1.120672	ENSG0000	DUSP1/NE
GO:004001	2	2/791	42/20610	0.482958	0.736964	1.240744	ENSG0000	WNT1/SCX
GO:001890	1	1/791	17/20610	0.486016	0.736964	1.532684	ENSG0000	TMEM86A
GO:003368	1	1/791	17/20610	0.486016	0.736964	1.532684	ENSG0000	SFRP1
GO:005188	1	1/791	17/20610	0.486016	0.736964	1.532684	ENSG0000	GAPDH
GO:190301	1	1/791	17/20610	0.486016	0.736964	1.532684	ENSG0000	FBN1
GO:190488	3	3/791	68/20610	0.487257	0.737155	1.149513	ENSG0000	INSIG2/MT
GO:004852	6	6/791	146/20610	0.491065	0.741675	1.070779	ENSG0000	JUN/GSN/
GO:000283	7	7/791	172/20610	0.491569	0.74176	1.060403	ENSG0000	OPTN/HTF
GO:190589	2	2/791	43/20610	0.495406	0.74506	1.21189	ENSG0000	CAV1/BOK
GO:006068	3	3/791	69/20610	0.497004	0.746258	1.132853	ENSG0000	VEGFA/SFI
GO:190134	8	8/791	200/20610	0.503329	0.747055	1.042225	ENSG0000	SERPINE1/
GO:002240	4	4/791	96/20610	0.505718	0.747055	1.085651	ENSG0000	EGFR/IGFB
GO:002240	4	4/791	96/20610	0.505718	0.747055	1.085651	ENSG0000	EGFR/IGFB
GO:000759	1	1/791	18/20610	0.505759	0.747055	1.447535	ENSG0000	A2M
GO:000763	1	1/791	18/20610	0.505759	0.747055	1.447535	ENSG0000	NTRK1
GO:001071	1	1/791	18/20610	0.505759	0.747055	1.447535	ENSG0000	ERRFI1
GO:006004	1	1/791	18/20610	0.505759	0.747055	1.447535	ENSG0000	PRSS37
GO:009709	1	1/791	18/20610	0.505759	0.747055	1.447535	ENSG0000	INSIG2
GO:190011	1	1/791	18/20610	0.505759	0.747055	1.447535	ENSG0000	BOK
GO:003164	3	3/791	70/20610	0.506657	0.747937	1.11667	ENSG0000	PTAFR/OP
GO:000931	19	19/791	488/20610	0.507771	0.748025	1.014461	ENSG0000	JUN/HRH1
GO:003028	5	5/791	123/20610	0.512193	0.75253	1.059172	ENSG0000	ANO6/LOX
GO:004263	5	5/791	123/20610	0.512193	0.75253	1.059172	ENSG0000	EGFR/IGFB
GO:003027	4	4/791	97/20610	0.51388	0.754561	1.074459	ENSG0000	IGFBP5/SF
GO:006107	3	3/791	71/20610	0.516213	0.757316	1.100942	ENSG0000	DNAJB2/H
GO:000733	2	2/791	45/20610	0.519722	0.758185	1.158028	ENSG0000	ALDOA/PF
GO:004361	2	2/791	45/20610	0.519722	0.758185	1.158028	ENSG0000	CDH3/EPP
GO:011011	2	2/791	45/20610	0.519722	0.758185	1.158028	ENSG0000	LIMS2/NO
GO:002290	8	8/791	203/20610	0.520267	0.758185	1.026823	ENSG0000	LOXL2/P4I
GO:000269	15	15/791	387/20610	0.521744	0.758185	1.009908	ENSG0000	AXL/PTAFI
GO:000734	1	1/791	19/20610	0.524744	0.758185	1.371349	ENSG0000	NOX5
GO:001608	1	1/791	19/20610	0.524744	0.758185	1.371349	ENSG0000	UNC13C
GO:003630	1	1/791	19/20610	0.524744	0.758185	1.371349	ENSG0000	VEGFA
GO:007257	1	1/791	19/20610	0.524744	0.758185	1.371349	ENSG0000	LIMS2
GO:190027	1	1/791	19/20610	0.524744	0.758185	1.371349	ENSG0000	APP
GO:002260	2	2/791	46/20610	0.531582	0.765392	1.132853	ENSG0000	PAM/PTPF
GO:000226	3	3/791	73/20610	0.535021	0.768725	1.070779	ENSG0000	JUN/APP/S
GO:007019	3	3/791	73/20610	0.535021	0.768725	1.070779	ENSG0000	SYCE1L/M
GO:005066	12	12/791	312/20610	0.538496	0.768725	1.002139	ENSG0000	WISP1/GA
GO:002301	1	1/791	20/20610	0.543001	0.768725	1.302781	ENSG0000	TBX6
GO:004263	1	1/791	20/20610	0.543001	0.768725	1.302781	ENSG0000	WNT10B
GO:006045	1	1/791	20/20610	0.543001	0.768725	1.302781	ENSG0000	OXTR
GO:000197	2	2/791	47/20610	0.543241	0.768725	1.10875	ENSG0000	AXL/NOL3
GO:003260	6	6/791	154/20610	0.543281	0.768725	1.015154	ENSG0000	AXL/INHA
GO:005068	7	7/791	181/20610	0.545714	0.769469	1.007676	ENSG0000	LIMS2/CAV
GO:003410	4	4/791	101/20610	0.54588	0.769469	1.031906	ENSG0000	ITGB3/EGF
GO:200025	3	3/791	75/20610	0.553408	0.776607	1.042225	ENSG0000	C1S/SUSD
GO:200105	4	4/791	102/20610	0.553708	0.776607	1.021789	ENSG0000	CAV1/ARG
GO:000182	2	2/791	48/20610	0.554697	0.776607	1.085651	ENSG0000	JUNB/MFN
GO:006124	2	2/791	48/20610	0.554697	0.776607	1.085651	ENSG0000	WNT11/AI
GO:012016	2	2/791	48/20610	0.554697	0.776607	1.085651	ENSG0000	ARRDC3/V

GO:000241	1	1/791	21/20610	0.560558	0.777144	1.240744	ENSG0000	PRF1
GO:003072	1	1/791	21/20610	0.560558	0.777144	1.240744	ENSG0000	TNFAIP6
GO:003289	1	1/791	21/20610	0.560558	0.777144	1.240744	ENSG0000	PLA2R1
GO:006071	1	1/791	21/20610	0.560558	0.777144	1.240744	ENSG0000	ADM
GO:004213	7	7/791	184/20610	0.563225	0.779319	0.991247	ENSG0000	CAV1/MO
GO:004591	2	2/791	49/20610	0.565947	0.780476	1.063495	ENSG0000	DDIT4/PRK
GO:003263	4	4/791	104/20610	0.569146	0.784234	1.002139	ENSG0000	SERPINE1/
GO:007016	2	2/791	50/20610	0.576991	0.786713	1.042225	ENSG0000	ANO6/WN
GO:003166	1	1/791	22/20610	0.57744	0.786713	1.184347	ENSG0000	PRDX2
GO:003235	1	1/791	22/20610	0.57744	0.786713	1.184347	ENSG0000	ADM
GO:004506	1	1/791	22/20610	0.57744	0.786713	1.184347	ENSG0000	DOCK2
GO:004852	1	1/791	22/20610	0.57744	0.786713	1.184347	ENSG0000	ARRDC3
GO:006004	1	1/791	22/20610	0.57744	0.786713	1.184347	ENSG0000	NOG
GO:003164	12	12/791	322/20610	0.582815	0.793165	0.971017	ENSG0000	ATP1B1/FL
GO:006099	4	4/791	106/20610	0.584282	0.793591	0.983231	ENSG0000	SDK1/LZTS
GO:007066	4	4/791	106/20610	0.584282	0.793591	0.983231	ENSG0000	ARG2/GNF
GO:006004	6	6/791	161/20610	0.586948	0.793591	0.971017	ENSG0000	SDK1/LAM
GO:001071	2	2/791	51/20610	0.587828	0.793591	1.021789	ENSG0000	LOXL2/TG
GO:004409	2	2/791	51/20610	0.587828	0.793591	1.021789	ENSG0000	EPHB2/CA
GO:190382	13	13/791	350/20610	0.587854	0.793591	0.96778	ENSG0000	EGFR/EPH
GO:002241	14	14/791	377/20610	0.588636	0.793591	0.967583	ENSG0000	KDM3A/SF
GO:000979	5	5/791	134/20610	0.588644	0.793591	0.972225	ENSG0000	CITED2/DN
GO:000728	1	1/791	23/20610	0.593675	0.793591	1.132853	ENSG0000	KDM3A
GO:003438	1	1/791	23/20610	0.593675	0.793591	1.132853	ENSG0000	SCARB1
GO:009015	1	1/791	23/20610	0.593675	0.793591	1.132853	ENSG0000	SPHK1
GO:190042	1	1/791	23/20610	0.593675	0.793591	1.132853	ENSG0000	ARG2
GO:200103	1	1/791	23/20610	0.593675	0.793591	1.132853	ENSG0000	PINK1
GO:003355	3	3/791	80/20610	0.597419	0.796136	0.977086	ENSG0000	CACNA1B/
GO:000270	2	2/791	52/20610	0.598457	0.796136	1.002139	ENSG0000	SMAD7/PA
GO:000645	2	2/791	52/20610	0.598457	0.796136	1.002139	ENSG0000	HSPA2/DN
GO:005144	2	2/791	52/20610	0.598457	0.796136	1.002139	ENSG0000	DUSP1/NF
GO:000270	8	8/791	218/20610	0.601458	0.797291	0.95617	ENSG0000	GPI/NA/SM
GO:000695	12	12/791	327/20610	0.604286	0.797291	0.95617	ENSG0000	GPI/NA/GA
GO:005086	15	15/791	409/20610	0.608639	0.797291	0.955585	ENSG0000	AXL/PTAFI
GO:003057	2	2/791	53/20610	0.608879	0.797291	0.983231	ENSG0000	COL13A1/
GO:200017	2	2/791	53/20610	0.608879	0.797291	0.983231	ENSG0000	VEGFA/FLI
GO:000257	1	1/791	24/20610	0.609287	0.797291	1.085651	ENSG0000	FGL2
GO:000327	1	1/791	24/20610	0.609287	0.797291	1.085651	ENSG0000	NOG
GO:000761	1	1/791	24/20610	0.609287	0.797291	1.085651	ENSG0000	APP
GO:007098	1	1/791	24/20610	0.609287	0.797291	1.085651	ENSG0000	CYP2C9
GO:009950	1	1/791	24/20610	0.609287	0.797291	1.085651	ENSG0000	STX11
GO:014001	7	7/791	193/20610	0.613803	0.801621	0.945023	ENSG0000	C11orf80/S
GO:003501	3	3/791	82/20610	0.61421	0.801621	0.953255	ENSG0000	LBH/SFRP1
GO:011011	3	3/791	82/20610	0.61421	0.801621	0.953255	ENSG0000	VEGFA/NC
GO:000270	6	6/791	166/20610	0.616774	0.804121	0.94177	ENSG0000	GPI/NA/GI
GO:000317	2	2/791	54/20610	0.619093	0.805204	0.965023	ENSG0000	EFNA1/SC
GO:004202	2	2/791	54/20610	0.619093	0.805204	0.965023	ENSG0000	DNAJB2/H
GO:005160	2	2/791	54/20610	0.619093	0.805204	0.965023	ENSG0000	HPCA/NTF
GO:000836	5	5/791	139/20610	0.621144	0.805475	0.937253	ENSG0000	NDRG1/CX
GO:004851	6	6/791	167/20610	0.622592	0.805475	0.93613	ENSG0000	KDM3A/D
GO:000940	1	1/791	25/20610	0.6243	0.805475	1.042225	ENSG0000	PAM
GO:003106	1	1/791	25/20610	0.6243	0.805475	1.042225	ENSG0000	IGFBP5
GO:003209	1	1/791	25/20610	0.6243	0.805475	1.042225	ENSG0000	SCTR
GO:190022	1	1/791	25/20610	0.6243	0.805475	1.042225	ENSG0000	LRP1
GO:190207	1	1/791	25/20610	0.6243	0.805475	1.042225	ENSG0000	HPCA
GO:009700	4	4/791	112/20610	0.627782	0.808728	0.930558	ENSG0000	ABCG1/VL
GO:190370	8	8/791	224/20610	0.631935	0.811321	0.930558	ENSG0000	JUN/AXL/F
GO:000964	1	1/791	26/20610	0.638737	0.812702	1.002139	ENSG0000	BHLHE40

GO:00328	1	1/791	26/20610	0.638737	0.812702	1.002139	ENSG0000	GSN
GO:00481	1	1/791	26/20610	0.638737	0.812702	1.002139	ENSG0000	SDK1
GO:00514	1	1/791	26/20610	0.638737	0.812702	1.002139	ENSG0000	NPM2
GO:00515	1	1/791	26/20610	0.638737	0.812702	1.002139	ENSG0000	PRKN
GO:00607	1	1/791	26/20610	0.638737	0.812702	1.002139	ENSG0000	MDFI
GO:20000	1	1/791	26/20610	0.638737	0.812702	1.002139	ENSG0000	SNX33
GO:20001	1	1/791	26/20610	0.638737	0.812702	1.002139	ENSG0000	LIMS2
GO:19034	5	5/791	142/2061	0.639892	0.813545	0.917452	ENSG0000	CAV1/ARG
GO:00351	2	2/791	57/20610	0.6485	0.820483	0.914232	ENSG0000	OXTR/GRI
GO:00023	1	1/791	27/20610	0.652619	0.820483	0.965023	ENSG0000	PRF1
GO:00095	1	1/791	27/20610	0.652619	0.820483	0.965023	ENSG0000	SCARB1
GO:00459	1	1/791	27/20610	0.652619	0.820483	0.965023	ENSG0000	COL5A1
GO:00605	1	1/791	27/20610	0.652619	0.820483	0.965023	ENSG0000	CRYAB
GO:00606	1	1/791	27/20610	0.652619	0.820483	0.965023	ENSG0000	ADM
GO:00801	1	1/791	27/20610	0.652619	0.820483	0.965023	ENSG0000	AICDA
GO:00996	1	1/791	27/20610	0.652619	0.820483	0.965023	ENSG0000	CACNG4
GO:00485	4	4/791	116/2061	0.655114	0.822994	0.89847	ENSG0000	JUN/NR5A
GO:00457	15	15/791	426/2061	0.670535	0.834765	0.917452	ENSG0000	PDGFB/EG
GO:00329	12	12/791	344/2061	0.673129	0.83688	0.908917	ENSG0000	DOCK2/H
GO:00018	4	4/791	119/2061	0.674709	0.83688	0.875819	ENSG0000	JUNB/MF
GO:00197	5	5/791	148/2061	0.675619	0.83688	0.880258	ENSG0000	GAPDH/P
GO:00439	2	2/791	60/20610	0.676075	0.83688	0.868521	ENSG0000	C1QL1/CD
GO:00217	3	3/791	90/20610	0.676548	0.83688	0.868521	ENSG0000	ANXA3/PL
GO:00019	1	1/791	29/20610	0.678806	0.83688	0.89847	ENSG0000	PRKN
GO:00351	1	1/791	29/20610	0.678806	0.83688	0.89847	ENSG0000	ST8SIA6
GO:00600	1	1/791	29/20610	0.678806	0.83688	0.89847	ENSG0000	SYBU
GO:19052	1	1/791	29/20610	0.678806	0.83688	0.89847	ENSG0000	EGFR
GO:20010	1	1/791	29/20610	0.678806	0.83688	0.89847	ENSG0000	PINK1
GO:20001	3	3/791	91/20610	0.683792	0.842399	0.858977	ENSG0000	VEGFA/LI
GO:00336	1	1/791	30/20610	0.691151	0.844136	0.868521	ENSG0000	SFRP1
GO:00098	12	12/791	349/2061	0.692016	0.844984	0.895895	ENSG0000	DDIT4/EG
GO:00164	2	2/791	62/20610	0.693462	0.845365	0.840504	ENSG0000	PARP3/AI
GO:19030	7	7/791	209/2061	0.695279	0.847302	0.872676	ENSG0000	C11orf80/
GO:00988	4	4/791	123/2061	0.699615	0.850684	0.847337	ENSG0000	PXDN/AL
GO:00032	1	1/791	31/20610	0.703022	0.850684	0.840504	ENSG0000	ERRFI1
GO:00903	1	1/791	31/20610	0.703022	0.850684	0.840504	ENSG0000	WNT1
GO:00069	3	3/791	94/20610	0.704796	0.852	0.831563	ENSG0000	C1S/SUSD
GO:00025	2	2/791	64/20610	0.710071	0.856709	0.814238	ENSG0000	PARP3/AI
GO:00082	1	1/791	32/20610	0.714437	0.858015	0.814238	ENSG0000	DKK3
GO:00602	1	1/791	32/20610	0.714437	0.858015	0.814238	ENSG0000	CNR1
GO:00906	1	1/791	32/20610	0.714437	0.858015	0.814238	ENSG0000	EFNB3
GO:01400	6	6/791	185/2061	0.718174	0.86167	0.845047	ENSG0000	SYTL2/TU
GO:00351	5	5/791	157/2061	0.724632	0.86449	0.829797	ENSG0000	ADM/VEG
GO:00108	1	1/791	33/20610	0.725414	0.86449	0.789564	ENSG0000	SDK1
GO:00400	1	1/791	33/20610	0.725414	0.86449	0.789564	ENSG0000	VIL1
GO:20010	1	1/791	33/20610	0.725414	0.86449	0.789564	ENSG0000	PINK1
GO:19018	2	2/791	66/20610	0.725921	0.86449	0.789564	ENSG0000	IGFBP5/N
GO:19038	4	4/791	128/2061	0.728777	0.867476	0.814238	ENSG0000	ANKRD13
GO:00513	9	9/791	275/2061	0.732722	0.868129	0.85273	ENSG0000	DUSP1/C1
GO:00488	2	2/791	67/20610	0.733567	0.868129	0.77778	ENSG0000	SYNGR3/P
GO:00060	1	1/791	34/20610	0.735969	0.868129	0.766342	ENSG0000	IDH2
GO:00315	1	1/791	34/20610	0.735969	0.868129	0.766342	ENSG0000	DUSP1
GO:00714	1	1/791	34/20610	0.735969	0.868129	0.766342	ENSG0000	ABCG1
GO:00451	3	3/791	99/20610	0.737403	0.869407	0.789564	ENSG0000	SYCE1L/M
GO:00072	9	9/791	278/2061	0.744151	0.872845	0.843527	ENSG0000	KDM3A/D
GO:00316	1	1/791	35/20610	0.746119	0.872845	0.744446	ENSG0000	PRDX2
GO:00326	1	1/791	35/20610	0.746119	0.872845	0.744446	ENSG0000	ARG2
GO:00601	1	1/791	35/20610	0.746119	0.872845	0.744446	ENSG0000	FLT3LG

GO:190138	1	1/791	35/20610	0.746119	0.872845	0.744446	ENSG0000	CAV1
GO:200077	2	2/791	69/20610	0.748316	0.873974	0.755236	ENSG0000	YPEL3/AR
GO:006096	5	5/791	162/20610	0.74946	0.874677	0.804186	ENSG0000	EGFR/WTI
GO:009031	5	5/791	162/20610	0.74946	0.874677	0.804186	ENSG0000	FLNA/PIN
GO:004662	2	2/791	70/20610	0.755423	0.877237	0.744446	ENSG0000	HLX/SASH
GO:004336	1	1/791	36/20610	0.755879	0.877237	0.723767	ENSG0000	DOCK2
GO:004814	1	1/791	36/20610	0.755879	0.877237	0.723767	ENSG0000	SFRP1
GO:000652	14	14/791	426/20610	0.760645	0.882562	0.856288	ENSG0000	EGLN3/PL
GO:190383	1	1/791	37/20610	0.765265	0.883176	0.704206	ENSG0000	LYPD1
GO:000705	8	8/791	255/20610	0.766834	0.884462	0.817431	ENSG0000	INHA/DUS
GO:007209	2	2/791	72/20610	0.76912	0.885463	0.723767	ENSG0000	VEGFA/TH
GO:009034	2	2/791	72/20610	0.76912	0.885463	0.723767	ENSG0000	YPEL3/AR
GO:003263	6	6/791	197/20610	0.771811	0.887842	0.793572	ENSG0000	APP/PTAF
GO:003952	1	1/791	38/20610	0.77429	0.888841	0.685674	ENSG0000	DDX58
GO:190103	1	1/791	38/20610	0.77429	0.888841	0.685674	ENSG0000	BOK
GO:000175	2	2/791	73/20610	0.775714	0.889858	0.713853	ENSG0000	SFRP1/TB
GO:000220	2	2/791	73/20610	0.775714	0.889858	0.713853	ENSG0000	PARP3/AI
GO:000166	1	1/791	39/20610	0.782969	0.893228	0.668093	ENSG0000	LYPD1
GO:003263	1	1/791	39/20610	0.782969	0.893228	0.668093	ENSG0000	SASH3
GO:009006	10	10/791	319/20610	0.785465	0.895459	0.816791	ENSG0000	PDGFB/EG
GO:000170	3	3/791	108/20610	0.788826	0.897184	0.723767	ENSG0000	HOXD10/
GO:000721	1	1/791	40/20610	0.791314	0.897184	0.651391	ENSG0000	HRH1
GO:003250	1	1/791	40/20610	0.791314	0.897184	0.651391	ENSG0000	SNX33
GO:190274	1	1/791	40/20610	0.791314	0.897184	0.651391	ENSG0000	CRYAB
GO:007066	9	9/791	293/20610	0.796227	0.90152	0.800343	ENSG0000	HHLA2/A
GO:000734	1	1/791	41/20610	0.799339	0.903234	0.635503	ENSG0000	PRSS37
GO:002158	1	1/791	41/20610	0.799339	0.903234	0.635503	ENSG0000	WNT1
GO:000269	6	6/791	205/20610	0.803098	0.906546	0.762604	ENSG0000	SMAD7/H
GO:003001	4	4/791	143/20610	0.803467	0.906546	0.728829	ENSG0000	ALPK2/GS
GO:000701	9	9/791	296/20610	0.805637	0.906546	0.792232	ENSG0000	APP/DNA
GO:190186	5	5/791	175/20610	0.806145	0.906546	0.744446	ENSG0000	IGFBP5/JP
GO:003438	2	2/791	78/20610	0.806266	0.906546	0.668093	ENSG0000	VLDLR/SC
GO:001714	1	1/791	42/20610	0.807056	0.906546	0.620372	ENSG0000	LBH
GO:004662	1	1/791	42/20610	0.807056	0.906546	0.620372	ENSG0000	NOG
GO:190011	1	1/791	42/20610	0.807056	0.906546	0.620372	ENSG0000	BOK
GO:006096	1	1/791	43/20610	0.814476	0.911171	0.605945	ENSG0000	AICDA
GO:005077	7	7/791	239/20610	0.815389	0.911782	0.763135	ENSG0000	SMAD7/P
GO:000716	4	4/791	147/20610	0.820306	0.916042	0.708997	ENSG0000	SFRP1/W
GO:000236	5	5/791	179/20610	0.821389	0.916469	0.727811	ENSG0000	SMAD7/G
GO:004275	3	3/791	115/20610	0.822795	0.91676	0.679712	ENSG0000	BHLHE40/
GO:001982	5	5/791	180/20610	0.825045	0.918142	0.723767	ENSG0000	LOXL2/LB
GO:005501	3	3/791	116/20610	0.827247	0.918142	0.673852	ENSG0000	HEG1/ND
GO:003164	1	1/791	45/20610	0.828472	0.918142	0.579014	ENSG0000	TG
GO:003438	1	1/791	45/20610	0.828472	0.918142	0.579014	ENSG0000	SCARB1
GO:190197	1	1/791	45/20610	0.828472	0.918142	0.579014	ENSG0000	DUSP1
GO:001485	2	2/791	83/20610	0.833057	0.921136	0.627846	ENSG0000	NDRG4/N
GO:000225	1	1/791	46/20610	0.83507	0.921136	0.566427	ENSG0000	RAB17
GO:002157	1	1/791	46/20610	0.83507	0.921136	0.566427	ENSG0000	WNT1
GO:003410	1	1/791	46/20610	0.83507	0.921136	0.566427	ENSG0000	EGFR
GO:004259	1	1/791	46/20610	0.83507	0.921136	0.566427	ENSG0000	LYPD1
GO:004568	1	1/791	46/20610	0.83507	0.921136	0.566427	ENSG0000	WNT10B
GO:005198	1	1/791	46/20610	0.83507	0.921136	0.566427	ENSG0000	DUSP1
GO:001092	3	3/791	118/20610	0.835864	0.921685	0.662431	ENSG0000	TNNT1/O
GO:003410	4	4/791	151/20610	0.835938	0.921685	0.690215	ENSG0000	VEGFA/AX
GO:003261	4	4/791	152/20610	0.839664	0.924556	0.685674	ENSG0000	APP/ERRF
GO:007066	5	5/791	185/20610	0.842426	0.925961	0.704206	ENSG0000	HHLA2/TL
GO:006025	1	1/791	48/20610	0.847514	0.92847	0.542826	ENSG0000	IDH2
GO:003134	10	10/791	342/20610	0.849605	0.929735	0.76186	ENSG0000	HTRA1/PR

GO:00458	4	4/791	155/2061	0.850417	0.930419	0.672403	ENSG0000	ADM/PDG
GO:00466	3	3/791	122/2061	0.85199	0.931571	0.640712	ENSG0000	HLX/NOG/
GO:00604	3	3/791	123/2061	0.855799	0.934353	0.635503	ENSG0000	HEG1/NDI
GO:00147	1	1/791	50/20610	0.859021	0.935914	0.521113	ENSG0000	PDE9A
GO:19052	1	1/791	52/20610	0.86966	0.943366	0.50107	ENSG0000	GPER1
GO:00099	1	1/791	55/20610	0.884134	0.953103	0.473739	ENSG0000	NPM2
GO:00341	1	1/791	55/20610	0.884134	0.953103	0.473739	ENSG0000	TLR9
GO:00309	4	4/791	167/2061	0.887509	0.956097	0.624087	ENSG0000	FLNA/SCN
GO:00028	1	1/791	56/20610	0.888593	0.956097	0.465279	ENSG0000	OPTN
GO:19015	5	5/791	204/2061	0.895953	0.961061	0.638618	ENSG0000	EDN2/ALC
GO:00600	1	1/791	58/20610	0.897003	0.961061	0.449235	ENSG0000	NOG
GO:00971	1	1/791	58/20610	0.897003	0.961061	0.449235	ENSG0000	CACNG4
GO:20002	1	1/791	58/20610	0.897003	0.961061	0.449235	ENSG0000	DUSP1
GO:19021	1	1/791	59/20610	0.900967	0.96321	0.441621	ENSG0000	PC
GO:00600	1	1/791	62/20610	0.911968	0.967907	0.420252	ENSG0000	SDK1
GO:00074	1	1/791	63/20610	0.915357	0.969018	0.413581	ENSG0000	VEGFA
GO:00076	1	1/791	63/20610	0.915357	0.969018	0.413581	ENSG0000	GUCY2D
GO:00521	1	1/791	63/20610	0.915357	0.969018	0.413581	ENSG0000	PC
GO:00434	5	5/791	214/2061	0.917219	0.970783	0.608776	ENSG0000	SAMD4A/
GO:00519	2	2/791	106/2061	0.917943	0.971341	0.491616	ENSG0000	DUSP1/NE
GO:00486	1	1/791	64/20610	0.918616	0.971639	0.407119	ENSG0000	NOG
GO:00022	1	1/791	65/20610	0.921749	0.972881	0.400856	ENSG0000	SPNS2
GO:00513	4	4/791	182/2061	0.922394	0.973355	0.572651	ENSG0000	VEGFA/LB
GO:19037	1	1/791	66/20610	0.924762	0.974611	0.394782	ENSG0000	PINK1
GO:00096	1	1/791	67/20610	0.927659	0.976628	0.38889	ENSG0000	GAPDH
GO:00323	1	1/791	68/20610	0.930444	0.977488	0.383171	ENSG0000	CRYAB
GO:00450	1	1/791	68/20610	0.930444	0.977488	0.383171	ENSG0000	PPP2R5B
GO:00604	4	4/791	188/2061	0.933386	0.979001	0.554375	ENSG0000	CAV1/WN
GO:00215	2	2/791	113/2061	0.934348	0.979724	0.461162	ENSG0000	SCN5A/W
GO:00447	1	1/791	72/20610	0.940555	0.982085	0.361884	ENSG0000	AICDA
GO:00512	1	1/791	72/20610	0.940555	0.982085	0.361884	ENSG0000	PKHD1
GO:00034	1	1/791	73/20610	0.942845	0.983648	0.356926	ENSG0000	SDK1
GO:00422	4	4/791	197/2061	0.947246	0.986138	0.529048	ENSG0000	SLAMF7/U
GO:00063	2	2/791	120/2061	0.947614	0.986138	0.43426	ENSG0000	ZNF304/A
GO:00220	2	2/791	123/2061	0.95248	0.98975	0.423669	ENSG0000	SCN5A/W
GO:00481	1	1/791	78/20610	0.953037	0.989914	0.334046	ENSG0000	COL7A1
GO:19001	1	1/791	78/20610	0.953037	0.989914	0.334046	ENSG0000	FLNA
GO:00106	2	2/791	124/2061	0.954004	0.990505	0.420252	ENSG0000	STC1/EPP
GO:00024	1	1/791	80/20610	0.956585	0.992355	0.325695	ENSG0000	VAV1
GO:00480	8	8/791	348/2061	0.959331	0.99389	0.59898	ENSG0000	NCF2/HLA
GO:00723	1	1/791	83/20610	0.961413	0.994869	0.313923	ENSG0000	MUC1
GO:00326	1	1/791	84/20610	0.9629	0.995386	0.310186	ENSG0000	TLR9
GO:00447	1	1/791	89/20610	0.969519	0.999716	0.29276	ENSG0000	CAV1
GO:00009	3	3/791	179/2061	0.970088	0.999999	0.436686	ENSG0000	RHOB/SN
GO:00977	1	1/791	91/20610	0.971824	0.999999	0.286326	ENSG0000	DNAH11
GO:20010	1	1/791	97/20610	0.977745	0.999999	0.268615	ENSG0000	MUC1
GO:00198	7	7/791	341/2061	0.978153	0.999999	0.534866	ENSG0000	NCF2/HLA
GO:00070	3	3/791	193/2061	0.980356	0.999999	0.40501	ENSG0000	DUSP1/W
GO:00326	2	2/791	153/2061	0.98245	0.999999	0.340596	ENSG0000	TLR9/DDX
GO:00988	5	5/791	278/2061	0.983265	0.999999	0.468626	ENSG0000	DUSP1/NE
GO:00344	1	1/791	105/2061	0.983753	0.999999	0.248149	ENSG0000	CNR1
GO:00070	3	3/791	202/2061	0.985075	0.999999	0.386965	ENSG0000	FLNA/NEK
GO:01200	1	1/791	108/2061	0.985561	0.999999	0.241256	ENSG0000	WNT1
GO:00353	1	1/791	121/2061	0.991344	0.999999	0.215336	ENSG0000	PDK1
GO:00109	8	8/791	424/2061	0.992854	0.999999	0.491616	ENSG0000	DUSP1/PP
GO:19021	3	3/791	226/2061	0.992934	0.999999	0.345871	ENSG0000	GSN/LRFN
GO:00070	1	1/791	132/2061	0.994387	0.999999	0.197391	ENSG0000	PKHD1
GO:00315	2	2/791	188/2061	0.994715	0.999999	0.277188	ENSG0000	PEA15/MU

GO:00067!	7	7/791	399/2061(	0.994773	0.999999	0.457116	ENSG0000	PDK1/MC(
GO:00022:	7	7/791	406/2061(	0.995637	0.999999	0.449235	ENSG0000	CAV1/TNII
GO:00310:	1	1/791	141/2061(	0.996063	0.999999	0.184792	ENSG0000	PKHD1
GO:00434:	4	4/791	337/2061(	0.999113	0.999999	0.309266	ENSG0000	KDM3A/ZI
GO:00620:	48	48/841	438/21737	9.11E-11	2.23E-08	2.832505	ENSG0000	LOXL2/SEI
GO:00300!	9	9/841	30/21737	1.28E-06	0.000104	7.753983	ENSG0000	PPL/PERP/
GO:00444:	12	12/841	57/21737	1.48E-06	0.000104	5.441392	ENSG0000	COL12A1/
GO:00423(	18	18/841	147/21737	1.57E-05	0.000944	3.164891	ENSG0000	SLC2A1/A
GO:00083(	8	8/841	31/21737	1.73E-05	0.000944	6.670093	ENSG0000	ITGB3/ITG
GO:00970(	37	37/841	470/21737	3.80E-05	0.001693	2.034733	ENSG0000	SLC16A3/I
GO:00312!	33	33/841	430/21737	0.000158	0.006466	1.983577	ENSG0000	ITGB3/CX(
GO:00163:	27	27/841	354/21737	0.000665	0.023281	1.971352	ENSG0000	SLC2A1/UI
GO:00314:	6	6/841	29/21737	0.000729	0.023803	5.347575	ENSG0000	OBSL1/EN
GO:00312!	17	17/841	183/21737	0.000777	0.023803	2.401051	ENSG0000	ITGB3/JCA
GO:00316	15	15/841	152/21737	0.000837	0.024129	2.550652	ENSG0000	SLC2A1/PI
GO:00448!	13	13/841	125/21737	0.001122	0.028417	2.688048	ENSG0000	SLC2A1/A
GO:00300:	14	14/841	141/21737	0.00116	0.028417	2.56633	ENSG0000	SLC2A1/PI
GO:00451	30	30/841	426/21737	0.001248	0.029123	1.820184	ENSG0000	SLC2A1/UI
GO:00452:	26	26/841	357/21737	0.001607	0.0328	1.882386	ENSG0000	SLC16A3/I
GO:00986(	6	6/841	34/21737	0.001752	0.033289	4.561167	ENSG0000	SYT12/PRI
GO:00300:	18	18/841	216/21737	0.001917	0.033289	2.153884	ENSG0000	SLC2A1/PI
GO:00444	19	19/841	234/21737	0.001964	0.033289	2.098656	ENSG0000	SLC2A1/PI
GO:00312!	27	27/841	386/21737	0.002345	0.035548	1.807924	ENSG0000	ITGB3/JCA
GO:00986	5	5/841	25/21737	0.002387	0.035548	5.169322	ENSG0000	COL4A3/C
GO:00163:	19	19/841	244/21737	0.003138	0.044799	2.012646	ENSG0000	SLC2A1/PI
GO:00198!	15	15/841	174/21737	0.0032	0.044799	2.228156	ENSG0000	ERRFI1/SN
GO:00316	6	6/841	40/21737	0.004109	0.052984	3.876992	ENSG0000	OBSL1/EN
GO:00432!	3	3/841	10/21737	0.005647	0.064475	7.753983	ENSG0000	LAMB3/LA
GO:00319(	26	26/841	393/21737	0.00579	0.064475	1.709954	ENSG0000	SERPINE1/
GO:00988!	24	24/841	355/21737	0.006039	0.065756	1.747377	ENSG0000	PGK1/SLC
GO:00017:	15	15/841	187/21737	0.006231	0.066379	2.073258	ENSG0000	ITGB3/PDL
GO:00015:	8	8/841	72/21737	0.006537	0.067049	2.871846	ENSG0000	PPL/DSC2/
GO:00996:	10	10/841	105/21737	0.007508	0.072472	2.461582	ENSG0000	SLC16A3/(
GO:00325(	7	7/841	60/21737	0.008226	0.073484	3.015438	ENSG0000	GABARAPI
GO:00059:	29	29/841	467/21737	0.008723	0.076324	1.605036	ENSG0000	ITGB3/TNS
GO:00988:	8	8/841	76/21737	0.009006	0.077417	2.720696	ENSG0000	SLC16A3/(
GO:00985(	24	24/841	368/21737	0.009229	0.077968	1.685649	ENSG0000	PGK1/SLC
GO:00988(	15	15/841	200/21737	0.011236	0.085936	1.938496	ENSG0000	ITGB3/ITG
GO:00996!	13	13/841	164/21737	0.011456	0.085936	2.048817	ENSG0000	SLC16A3/(
GO:00059:	11	11/841	129/21737	0.011575	0.085936	2.203975	ENSG0000	NDRG1/VI
GO:00725(	12	12/841	150/21737	0.013815	0.094019	2.067729	ENSG0000	ANGPTL4/
GO:00989:	11	11/841	134/21737	0.015065	0.101063	2.121737	ENSG0000	SLC16A3/(
GO:00430(	3	3/841	14/21737	0.015263	0.101063	5.53856	ENSG0000	LAMB2/C1
GO:00991	6	6/841	54/21737	0.017558	0.111752	2.871846	ENSG0000	SLC16A3/(
GO:00319(	15	15/841	211/21737	0.017561	0.111752	1.837437	ENSG0000	BNIP3L/BN
GO:00427:	13	13/841	175/21737	0.018769	0.11348	1.920034	ENSG0000	EPHB2/EFI
GO:00328(	4	4/841	27/21737	0.019222	0.11348	3.829128	ENSG0000	KCNC3/KC
GO:00992	13	13/841	176/21737	0.019577	0.114197	1.909125	ENSG0000	SLC16A3/(
GO:19024!	22	22/841	354/21737	0.020286	0.115583	1.606287	ENSG0000	CACNA1B/
GO:00995	23	23/841	375/21737	0.020567	0.115839	1.585259	ENSG0000	BNIP3/SLC
GO:00198!	20	20/841	315/21737	0.021286	0.115892	1.641055	ENSG0000	ERRFI1/SN
GO:00442!	4	4/841	28/21737	0.021757	0.117153	3.692373	ENSG0000	KCNC3/KC
GO:00990(	7	7/841	77/21737	0.029312	0.152799	2.349692	ENSG0000	SLC16A3/(
GO:00432(	11	11/841	149/21737	0.030288	0.154593	1.908139	ENSG0000	SLC2A3/EI
GO:00140(	21	21/841	349/21737	0.031148	0.157345	1.55524	ENSG0000	BNIP3/SLC
GO:00442!	7	7/841	79/21737	0.033104	0.162212	2.290206	ENSG0000	SLC2A1/A
GO:00322	21	21/841	353/21737	0.034585	0.167064	1.537617	ENSG0000	BNIP3/SLC
GO:00059(	7	7/841	80/21737	0.035118	0.167064	2.261578	ENSG0000	VLDLR/AP

GO:00989	7	7/841	80/21737	0.035118	0.167064	2.261578	ENSG0000	SLC16A3/
GO:00098	24	24/841	429/21737	0.046203	0.203961	1.445964	ENSG0000	CXCR4/AB
GO:00972	3	3/841	22/21737	0.051423	0.222985	3.524538	ENSG0000	ENKUR/CA
GO:00015	2	2/841	10/21737	0.054751	0.235331	5.169322	ENSG0000	ADAMTS1
GO:00303	5	5/841	54/21737	0.057123	0.241296	2.393205	ENSG0000	CACNG4/S
GO:00306	20	20/841	353/21737	0.058072	0.243207	1.464397	ENSG0000	SLC2A3/IT
GO:00021	2	2/841	11/21737	0.065241	0.257808	4.699384	ENSG0000	PLXNA3/N
GO:01060	2	2/841	11/21737	0.065241	0.257808	4.699384	ENSG0000	SPAG4/SY
GO:00306	16	16/841	283/21737	0.084365	0.322459	1.461293	ENSG0000	CACNG4/
GO:00056	3	3/841	27/21737	0.084892	0.322459	2.871846	ENSG0000	GUCY2D/E
GO:00325	2	2/841	13/21737	0.08797	0.329049	3.976402	ENSG0000	KCNC3/GF
GO:00988	2	2/841	13/21737	0.08797	0.329049	3.976402	ENSG0000	PSD/SH3G
GO:00487	6	6/841	80/21737	0.089389	0.331823	1.938496	ENSG0000	PPFIA4/AF
GO:00312	11	11/841	189/21737	0.116936	0.421314	1.5043	ENSG0000	MELTF/EF
GO:00331	2	2/841	16/21737	0.125557	0.424471	3.230826	ENSG0000	OCA2/NA
GO:00336	2	2/841	16/21737	0.125557	0.424471	3.230826	ENSG0000	AXL/GBP2
GO:00430	2	2/841	16/21737	0.125557	0.424471	3.230826	ENSG0000	NCF2/NO
GO:01201	6	6/841	88/21737	0.125609	0.424471	1.762269	ENSG0000	GABARAP
GO:00059	17	17/841	325/21737	0.129373	0.434197	1.351977	ENSG0000	SLC2A1/N
GO:00055	2	2/841	17/21737	0.138809	0.453444	3.040778	ENSG0000	COL5A1/C
GO:00986	2	2/841	17/21737	0.138809	0.453444	3.040778	ENSG0000	COL5A1/C
GO:00986	2	2/841	17/21737	0.138809	0.453444	3.040778	ENSG0000	SLC16A3/
GO:00432	4	4/841	52/21737	0.141204	0.455197	1.988201	ENSG0000	GSN/TUBA
GO:00001	2	2/841	18/21737	0.152346	0.484737	2.871846	ENSG0000	PPP1R3B/
GO:00312	6	6/841	94/21737	0.156762	0.495569	1.649784	ENSG0000	ERRFI1/M
GO:00163	2	2/841	19/21737	0.166125	0.518479	2.720696	ENSG0000	NEDD4/EF
GO:00990	2	2/841	20/21737	0.180108	0.555048	2.584661	ENSG0000	PSD/SH3G
GO:00985	5	5/841	79/21737	0.190769	0.577016	1.635861	ENSG0000	BNIP3/FU
GO:00300	11	11/841	211/21737	0.195941	0.585433	1.347454	ENSG0000	ITGB3/AJU
GO:00431	3	3/841	40/21737	0.200736	0.589201	1.938496	ENSG0000	APP/FLNA
GO:00332	19	19/841	397/21737	0.200978	0.589201	1.236991	ENSG0000	APP/LRP1/
GO:00985	10	10/841	191/21737	0.206699	0.597573	1.353226	ENSG0000	ERRFI1/M
GO:00306	15	15/841	306/21737	0.208437	0.597573	1.266991	ENSG0000	PAM/SYN
GO:00099	3	3/841	41/21737	0.210826	0.60061	1.891215	ENSG0000	EGFR/TF/
GO:00990	2	2/841	23/21737	0.222926	0.616744	2.247531	ENSG0000	PSD/SH3G
GO:00319	16	16/841	334/21737	0.224495	0.616744	1.238161	ENSG0000	SLC16A3/
GO:00988	5	5/841	84/21737	0.225337	0.616744	1.538489	ENSG0000	EPHB2/EF
GO:00082	4	4/841	63/21737	0.226559	0.616744	1.641055	ENSG0000	PPP2R5B/
GO:19032	4	4/841	63/21737	0.226559	0.616744	1.641055	ENSG0000	PPP2R5B/
GO:00451	4	4/841	64/21737	0.234905	0.635932	1.615413	ENSG0000	QSOX1/PF
GO:00312	4	4/841	65/21737	0.243324	0.652922	1.590561	ENSG0000	QSOX1/B4
GO:00972	10	10/841	201/21737	0.252418	0.660008	1.285901	ENSG0000	CAV1/NCF
GO:00059	6	6/841	110/21737	0.253228	0.660008	1.409815	ENSG0000	SLC38A2/
GO:00343	2	2/841	26/21737	0.266413	0.683468	1.988201	ENSG0000	APOL1/VL
GO:00431	9	9/841	181/21737	0.268098	0.684208	1.285191	ENSG0000	APP/NEDD
GO:00308	2	2/841	27/21737	0.280935	0.705938	1.914564	ENSG0000	PLOD2/GN
GO:00098	8	8/841	163/21737	0.296767	0.730734	1.268545	ENSG0000	ERRFI1/M
GO:00098	8	8/841	163/21737	0.296767	0.730734	1.268545	ENSG0000	FSCN2/SL
GO:00443	7	7/841	143/21737	0.317342	0.765308	1.265219	ENSG0000	FSTL3/PTP
GO:00164	1	1/841	10/21737	0.326094	0.765308	2.584661	ENSG0000	MYL6
GO:00058	2	2/841	31/21737	0.338588	0.768885	1.667523	ENSG0000	GNG4/NA
GO:19053	2	2/841	31/21737	0.338588	0.768885	1.667523	ENSG0000	GNG4/NA
GO:00361	5	5/841	100/21737	0.344971	0.768885	1.292331	ENSG0000	GSTM3/EN
GO:00058	1	1/841	11/21737	0.352179	0.768885	2.349692	ENSG0000	TNNT1
GO:00330	1	1/841	11/21737	0.352179	0.768885	2.349692	ENSG0000	AKR1B1
GO:00352	1	1/841	11/21737	0.352179	0.768885	2.349692	ENSG0000	APP
GO:00618	1	1/841	11/21737	0.352179	0.768885	2.349692	ENSG0000	ALDOA
GO:00985	6	6/841	126/21737	0.361557	0.773637	1.230791	ENSG0000	HLA-B/NA

GO:00451	3	3/841	58/21737	0.390403	0.786672	1.336894	ENSG0000	EGFR/TF/C
GO:00363	2	2/841	35/21737	0.394754	0.786672	1.476949	ENSG0000	TNNT1/OE
GO:00312	12	12/841	279/21737	0.395514	0.786672	1.111682	ENSG0000	INSIG2/DN
GO:00304	8	8/841	180/21737	0.395773	0.786672	1.148738	ENSG0000	APP/LRP1/
GO:00163	3	3/841	61/21737	0.421804	0.793984	1.271145	ENSG0000	SNTA1/SC
GO:00320	1	1/841	14/21737	0.424538	0.793984	1.846187	ENSG0000	RASGRP3
GO:00336	1	1/841	14/21737	0.424538	0.793984	1.846187	ENSG0000	GBP2
GO:00432	1	1/841	14/21737	0.424538	0.793984	1.846187	ENSG0000	AKR1B1
GO:00436	1	1/841	14/21737	0.424538	0.793984	1.846187	ENSG0000	GBP2
GO:00992	1	1/841	14/21737	0.424538	0.793984	1.846187	ENSG0000	SNAP91
GO:00304	8	8/841	186/21737	0.431174	0.803327	1.111682	ENSG0000	APP/LRP1/
GO:00016	5	5/841	113/21737	0.445002	0.808211	1.143655	ENSG0000	CAV1/NCF
GO:00059	1	1/841	15/21737	0.446817	0.808211	1.723107	ENSG0000	PRKAR1B
GO:00315	1	1/841	15/21737	0.446817	0.808211	1.723107	ENSG0000	NIM1K
GO:00330	2	2/841	39/21737	0.448639	0.808211	1.325467	ENSG0000	JPH2/TME
GO:00315	8	8/841	191/21737	0.460516	0.811944	1.082581	ENSG0000	SPAG4/HA
GO:00715	1	1/841	16/21737	0.468234	0.811944	1.615413	ENSG0000	DPF3
GO:00987	1	1/841	16/21737	0.468234	0.811944	1.615413	ENSG0000	ANKZF1
GO:00988	1	1/841	16/21737	0.468234	0.811944	1.615413	ENSG0000	UNC13C
GO:00313	9	9/841	221/21737	0.485672	0.811944	1.052577	ENSG0000	BNIP3/QS
GO:00444	2	2/841	42/21737	0.487228	0.811944	1.230791	ENSG0000	DNAH11/I
GO:00432	1	1/841	17/21737	0.488823	0.811944	1.520389	ENSG0000	AKR1B1
GO:00985	1	1/841	17/21737	0.488823	0.811944	1.520389	ENSG0000	GNRH1
GO:00306	11	11/841	275/21737	0.498493	0.82243	1.033864	ENSG0000	EGFR/SEC
GO:00080	8	8/841	199/21737	0.506772	0.822701	1.03906	ENSG0000	SYNGR3/S
GO:00485	1	1/841	18/21737	0.508616	0.822701	1.435923	ENSG0000	BHLHE40-
GO:00343	2	2/841	44/21737	0.511998	0.822701	1.174846	ENSG0000	APOL1/VL
GO:19907	2	2/841	44/21737	0.511998	0.822701	1.174846	ENSG0000	APOL1/VL
GO:00436	5	5/841	124/21737	0.526565	0.826023	1.042202	ENSG0000	PTPRN/AD
GO:00001	1	1/841	19/21737	0.527643	0.826023	1.360348	ENSG0000	NA
GO:00058	1	1/841	19/21737	0.527643	0.826023	1.360348	ENSG0000	MYL9
GO:19027	1	1/841	20/21737	0.545935	0.838583	1.292331	ENSG0000	GABRE
GO:00306	8	8/841	207/21737	0.551708	0.841307	0.998903	ENSG0000	SEC31B/HI
GO:00357	10	10/841	260/21737	0.552859	0.841307	0.9941	ENSG0000	SAMD4A/
GO:00001	1	1/841	21/21737	0.563519	0.849613	1.230791	ENSG0000	PPP2R5B
GO:00452	1	1/841	21/21737	0.563519	0.849613	1.230791	ENSG0000	C15orf48
GO:19902	5	5/841	130/21737	0.568777	0.85491	0.9941	ENSG0000	PDK1/P4H
GO:00160	1	1/841	22/21737	0.580422	0.858121	1.174846	ENSG0000	SNTA1
GO:00019	2	2/841	50/21737	0.581421	0.858121	1.033864	ENSG0000	ENO2/INH
GO:00020	1	1/841	23/21737	0.596672	0.864998	1.123766	ENSG0000	CAV1
GO:00164	1	1/841	23/21737	0.596672	0.864998	1.123766	ENSG0000	MYL9
GO:00331	1	1/841	23/21737	0.596672	0.864998	1.123766	ENSG0000	ATP6V0A4
GO:00469	1	1/841	23/21737	0.596672	0.864998	1.123766	ENSG0000	CD34
GO:00313	9	9/841	244/21737	0.605337	0.868554	0.953359	ENSG0000	BNIP3/QS
GO:00001	1	1/841	24/21737	0.612293	0.868554	1.076942	ENSG0000	CARHSP1
GO:00329	1	1/841	24/21737	0.612293	0.868554	1.076942	ENSG0000	MYH15
GO:00302	2	2/841	54/21737	0.623512	0.875418	0.957282	ENSG0000	DNAH11/I
GO:00324	2	2/841	54/21737	0.623512	0.875418	0.957282	ENSG0000	FSCN2/DC
GO:19053	1	1/841	25/21737	0.62731	0.875732	1.033864	ENSG0000	CARHSP1
GO:00973	1	1/841	26/21737	0.641746	0.890809	0.9941	ENSG0000	GUCY2D
GO:00321	2	2/841	57/21737	0.652878	0.898624	0.906899	ENSG0000	RHOB/PSD
GO:00324	2	2/841	59/21737	0.671421	0.913879	0.876156	ENSG0000	FSCN2/DC
GO:00001	2	2/841	60/21737	0.680388	0.917792	0.861554	ENSG0000	FAM60A/N
GO:00017	3	3/841	95/21737	0.716674	0.95518	0.816209	ENSG0000	INHA/GUC
GO:00487	1	1/841	32/21737	0.717359	0.95518	0.807707	ENSG0000	STX11
GO:00343	1	1/841	33/21737	0.728311	0.96452	0.783231	ENSG0000	APOL1
GO:00321	2	2/841	68/21737	0.745078	0.970265	0.760194	ENSG0000	RHOB/PSD
GO:00319	3	3/841	100/21737	0.748478	0.970265	0.775398	ENSG0000	APP/PRELI

GO:00059[illegible]	1	1/841	35/21737	0.748959	0.970265	0.738475	ENSG0000	GJB3
GO:19049[illegible]	4	4/841	131/21737	0.750849	0.970265	0.78921	ENSG0000	ATP1B1/FX
GO:00162[illegible]	3	3/841	101/21737	0.754484	0.970265	0.767721	ENSG0000	GYS1/PINK
GO:00601[illegible]	3	3/841	101/21737	0.754484	0.970265	0.767721	ENSG0000	GUCY2D/F
GO:00017[illegible]	1	1/841	36/21737	0.758687	0.970265	0.717961	ENSG0000	ICAM1
GO:00975[illegible]	1	1/841	37/21737	0.768039	0.974972	0.698557	ENSG0000	PRKAR1B
GO:00444[illegible]	8	8/841	255/21737	0.774326	0.97506	0.810874	ENSG0000	BNIP3/AN
GO:00321[illegible]	2	2/841	73/21737	0.779546	0.97506	0.708126	ENSG0000	RHOB/PSD
GO:00701[illegible]	4	4/841	137/21737	0.781148	0.97506	0.754646	ENSG0000	AMOTL2/E
GO:00018[illegible]	1	1/841	39/21737	0.78567	0.97506	0.662734	ENSG0000	PEAR1
GO:00306[illegible]	3	3/841	107/21737	0.788124	0.97506	0.724671	ENSG0000	SYNGR3/S
GO:00436[illegible]	2	2/841	75/21737	0.792148	0.97506	0.689243	ENSG0000	AXL/GBP2
GO:00444[illegible]	16	16/841	495/21737	0.803051	0.983738	0.835446	ENSG0000	INHA/APP
GO:00003[illegible]	1	1/841	43/21737	0.817019	0.994406	0.601084	ENSG0000	CCNG2
GO:00442[illegible]	2	2/841	83/21737	0.836399	1	0.62281	ENSG0000	AXL/GBP2
GO:00432[illegible]	4	4/841	152/21737	0.844129	1	0.680174	ENSG0000	AMOTL2/E
GO:00301[illegible]	1	1/841	48/21737	0.849844	1	0.538471	ENSG0000	AP3B2
GO:00312[illegible]	1	1/841	48/21737	0.849844	1	0.538471	ENSG0000	STX11
GO:00977[illegible]	4	4/841	159/21737	0.86786	1	0.650229	ENSG0000	INHA/GUC
GO:00313[illegible]	1	1/841	52/21737	0.871815	1	0.49705	ENSG0000	PSD
GO:00431[illegible]	1	1/841	53/21737	0.876786	1	0.487672	ENSG0000	UNC13C
GO:00059[illegible]	3	3/841	130/21737	0.883453	1	0.59646	ENSG0000	DNAH11/D
GO:00970[illegible]	3	3/841	131/21737	0.886561	1	0.591907	ENSG0000	DNAH11/D
GO:00164[illegible]	1	1/841	57/21737	0.894819	1	0.453449	ENSG0000	ATP6V0A4
GO:00301[illegible]	2	2/841	100/21737	0.903367	1	0.516932	ENSG0000	SEC31B/AI
GO:00988[illegible]	2	2/841	101/21737	0.906375	1	0.511814	ENSG0000	C15orf48/\
GO:00057[illegible]	2	2/841	104/21737	0.914881	1	0.49705	ENSG0000	C15orf48/\
GO:00058[illegible]	1	1/841	63/21737	0.917047	1	0.410264	ENSG0000	KIFC2
GO:19053[illegible]	2	2/841	106/21737	0.920143	1	0.487672	ENSG0000	F3/DNAJB[illegible]
GO:00057[illegible]	1	1/841	66/21737	0.926334	1	0.391615	ENSG0000	WDR93
GO:00309[illegible]	1	1/841	66/21737	0.926334	1	0.391615	ENSG0000	WDR93
GO:00447[illegible]	5	5/841	226/21737	0.940256	1	0.571828	ENSG0000	JUN/MXI1/
GO:00704[illegible]	2	2/841	116/21737	0.942158	1	0.445631	ENSG0000	C15orf48/\
GO:00058[illegible]	1	1/841	74/21737	0.946331	1	0.349279	ENSG0000	VIM
GO:00056[illegible]	10	10/841	400/21737	0.949053	1	0.646165	ENSG0000	JUN/MXI1/
GO:00005[illegible]	1	1/841	82/21737	0.960904	1	0.315203	ENSG0000	DNAJB2
GO:00360[illegible]	2	2/841	139/21737	0.972967	1	0.371894	ENSG0000	FANK1/PK
GO:00001[illegible]	6	6/841	298/21737	0.97557	1	0.520402	ENSG0000	NEDD4/ZS
GO:00312[illegible]	1	1/841	100/21737	0.980841	1	0.258466	ENSG0000	DPF3
GO:00988[illegible]	2	2/841	158/21737	0.985807	1	0.327172	ENSG0000	C15orf48/\
GO:00304[illegible]	2	2/841	178/21737	0.992886	1	0.290411	ENSG0000	SLC2A1/CI
GO:00305[illegible]	1	1/841	125/21737	0.992893	1	0.206773	ENSG0000	RNVU1-7
GO:00347[illegible]	1	1/841	130/21737	0.994172	1	0.19882	ENSG0000	KDM4B
GO:00480[illegible]	15	15/787	77/19728	3.17E-07	8.03E-05	4.883249	ENSG0000	SLC2A3/E(
GO:00198[illegible]	21	21/787	149/19728	4.98E-07	8.03E-05	3.532981	ENSG0000	WISP2/ITG
GO:00055[illegible]	15	15/787	84/19728	1.03E-06	0.000132	4.476311	ENSG0000	TGFBI/PD(
GO:00019[illegible]	7	7/787	27/19728	6.91E-05	0.003892	6.498941	ENSG0000	ITGB3/VEG
GO:00051[illegible]	16	16/787	144/19728	0.000208	0.009648	2.78526	ENSG0000	WISP2/TGI
GO:00300[illegible]	8	8/787	47/19728	0.000489	0.016934	4.266782	ENSG0000	COL12A1/
GO:00053[illegible]	16	16/787	156/19728	0.000515	0.016934	2.57101	ENSG0000	SLC16A3/S
GO:00611[illegible]	21	21/787	247/19728	0.000951	0.024203	2.131232	ENSG0000	SERPINE1/
GO:00151[illegible]	7	7/787	42/19728	0.001247	0.028945	4.177891	ENSG0000	SLC2A3/SL
GO:00228[illegible]	34	34/787	496/19728	0.001604	0.035152	1.718326	ENSG0000	TMEM37/(
GO:00306[illegible]	16	16/787	187/19728	0.00344	0.055343	2.1448	ENSG0000	GRB10/OP
GO:00055[illegible]	30	30/787	443/19728	0.003502	0.055343	1.697563	ENSG0000	ABCG1/F3[illegible]
GO:00422[illegible]	28	28/787	409/19728	0.004044	0.060343	1.716102	ENSG0000	CACNA1B/
GO:00605[illegible]	27	27/787	390/19728	0.004053	0.060343	1.735432	ENSG0000	JUN/ATP1
GO:00167[illegible]	15	15/787	176/19728	0.004701	0.06869	2.136421	ENSG0000	EGLN3/PL(

GO:00016	14	14/787	162/1972	0.005487	0.077306	2.166314	ENSG0000	CXCR4/SC
GO:00510	17	17/787	215/1972	0.005806	0.079549	1.982069	ENSG0000	TAGLN/EG
GO:00228	27	27/787	401/1972	0.005848	0.079549	1.687826	ENSG0000	SLC16A3/
GO:00315	3	3/787	12/19728	0.010622	0.10352	6.266836	ENSG0000	EGLN3/P4
GO:00170	5	5/787	34/19728	0.010628	0.10352	3.686374	ENSG0000	NEDD4/SN
GO:00055	16	16/787	212/1972	0.011157	0.107351	1.891875	ENSG0000	DAPK1/EG
GO:00168	4	4/787	23/19728	0.012151	0.114136	4.359538	ENSG0000	NPR1/GU
GO:00199	3	3/787	13/19728	0.013404	0.122778	5.784772	ENSG0000	RASGRP1/
GO:00512	9	9/787	95/19728	0.013538	0.122778	2.374801	ENSG0000	EGLN3/PL
GO:00168	6	6/787	51/19728	0.015471	0.136418	2.949099	ENSG0000	ITGB3/QS
GO:00053	4	4/787	27/19728	0.021248	0.161196	3.713681	ENSG0000	SLC6A6/SL
GO:00170	6	6/787	55/19728	0.021806	0.16257	2.734619	ENSG0000	NPR1/GLP
GO:00991	10	10/787	120/1972	0.021841	0.16257	2.088945	ENSG0000	NEDD4/C
GO:00048	26	26/787	429/1972	0.023196	0.166297	1.519233	ENSG0000	SERPINE1/
GO:00723	3	3/787	16/19728	0.024019	0.166297	4.700127	ENSG0000	SLC6A8/SL
GO:00168	4	4/787	29/19728	0.027018	0.175942	3.457565	ENSG0000	GPI/NA/P
GO:00431	15	15/787	217/1972	0.027817	0.175942	1.732766	ENSG0000	EGLN3/PL
GO:00199	11	11/787	143/1972	0.02822	0.175942	1.928257	ENSG0000	ITGB3/CX
GO:00151	3	3/787	18/19728	0.033001	0.195772	4.177891	ENSG0000	SLCO1C1/
GO:00017	6	6/787	61/19728	0.034255	0.201695	2.46564	ENSG0000	AXL/SYT1
GO:00192	4	4/787	33/19728	0.041118	0.230902	3.038466	ENSG0000	AMPD3/C
GO:00355	7	7/787	82/19728	0.04516	0.24075	2.139895	ENSG0000	GRB10/AF
GO:00055	16	16/787	252/1972	0.045961	0.241971	1.591577	ENSG0000	WISP2/VE
GO:00082	13	13/787	193/1972	0.046002	0.241971	1.688474	ENSG0000	WISP2/VE
GO:00054	8	8/787	101/1972	0.04878	0.24701	1.985532	ENSG0000	ABCG1/CA
GO:00166	3	3/787	21/19728	0.049273	0.24701	3.581049	ENSG0000	LOXL2/LO
GO:00341	3	3/787	21/19728	0.049273	0.24701	3.581049	ENSG0000	VLDLR/LR
GO:00381	3	3/787	21/19728	0.049273	0.24701	3.581049	ENSG0000	PTAFR/SC
GO:00421	3	3/787	21/19728	0.049273	0.24701	3.581049	ENSG0000	ACHE/CH
GO:00168	2	2/787	10/19728	0.057833	0.275547	5.013469	ENSG0000	MCCC1/P
GO:00718	4	4/787	37/19728	0.058672	0.275547	2.709983	ENSG0000	APOL2/VL
GO:00431	7	7/787	88/19728	0.061747	0.282342	1.993993	ENSG0000	ABCG1/CR
GO:00055	5	5/787	54/19728	0.063451	0.285089	2.32105	ENSG0000	ABCG1/AN
GO:00198	4	4/787	38/19728	0.063594	0.285089	2.638668	ENSG0000	CRABP2/C
GO:00198	10	10/787	145/1972	0.065158	0.290449	1.728782	ENSG0000	EGLN3/PL
GO:00040	2	2/787	11/19728	0.068861	0.290541	4.557699	ENSG0000	ADCY9/AD
GO:00302	5	5/787	60/19728	0.090636	0.318033	2.088945	ENSG0000	SYT12/LR
GO:00324	3	3/787	27/19728	0.091106	0.318033	2.78526	ENSG0000	KDM3A/K
GO:00050	2	2/787	13/19728	0.092709	0.318033	3.856515	ENSG0000	VLDLR/LR
GO:00162	2	2/787	13/19728	0.092709	0.318033	3.856515	ENSG0000	PFKP/PFKL
GO:00434	2	2/787	13/19728	0.092709	0.318033	3.856515	ENSG0000	SPAG4/RA
GO:00152	7	7/787	99/19728	0.100674	0.336574	1.772438	ENSG0000	SLC25A29
GO:00429	2	2/787	14/19728	0.105401	0.343641	3.581049	ENSG0000	SLC2A1/SL
GO:00430	4	4/787	46/19728	0.110224	0.357886	2.179769	ENSG0000	NOL3/TN
GO:00463	6	6/787	83/19728	0.113826	0.366566	1.812097	ENSG0000	JUN/TGF
GO:00432	3	3/787	30/19728	0.116064	0.370746	2.506734	ENSG0000	PLEKHA2/
GO:00481	2	2/787	15/19728	0.118524	0.374061	3.342313	ENSG0000	FSTL3/SM
GO:00167	3	3/787	31/19728	0.124895	0.38644	2.425872	ENSG0000	P4HA2/P4
GO:00150	4	4/787	49/19728	0.130754	0.388684	2.046314	ENSG0000	ITGB3/CX
GO:00037	2	2/787	16/19728	0.132024	0.388684	3.133418	ENSG0000	ITGB3/QS
GO:19904	2	2/787	16/19728	0.132024	0.388684	3.133418	ENSG0000	TIPARP/PA
GO:00443	8	8/787	127/1972	0.135456	0.394371	1.579045	ENSG0000	FLNA/CAV
GO:00986	4	4/787	50/19728	0.137925	0.398617	2.005388	ENSG0000	DSCAML1
GO:00167	16	16/787	299/1972	0.144251	0.405201	1.341396	ENSG0000	FUT11/TIP
GO:00192	2	2/787	17/19728	0.145852	0.405201	2.949099	ENSG0000	VIM/EPPK
GO:00050	9	9/787	151/1972	0.150195	0.414349	1.49408	ENSG0000	ARHGEF37
GO:00018	21	21/787	415/1972	0.158175	0.430345	1.268468	ENSG0000	DAPK1/AK
GO:00200	9	9/787	154/1972	0.162991	0.437415	1.464975	ENSG0000	STC2/BAC

GO:00044	7	7/787	114/1972	0.170654	0.449419	1.539223	ENSG0000	PAM/MO
GO:00167	2	2/787	19/19728	0.174299	0.449419	2.638668	ENSG0000	STEAP3/C
GO:00080	11	11/787	202/1972	0.184886	0.463095	1.365053	ENSG0000	CACNA1B
GO:00168	8	8/787	138/1972	0.186673	0.464713	1.453179	ENSG0000	PADI2/AM
GO:00050	2	2/787	20/19728	0.188834	0.467054	2.506734	ENSG0000	PSD4/PSD
GO:00316	2	2/787	20/19728	0.188834	0.467054	2.506734	ENSG0000	GNG4/NA
GO:00426	7	7/787	118/1972	0.192201	0.470159	1.487046	ENSG0000	HLA-B/NA
GO:00015	3	3/787	38/19728	0.192473	0.470159	1.979001	ENSG0000	PTAFR/SC
GO:00162	3	3/787	38/19728	0.192473	0.470159	1.979001	ENSG0000	NEDD4/C
GO:00904	3	3/787	38/19728	0.192473	0.470159	1.979001	ENSG0000	SLC2A1/S
GO:00433	3	3/787	39/19728	0.202786	0.479347	1.928257	ENSG0000	COL5A1/L
GO:00436	4	4/787	59/19728	0.208656	0.486716	1.699481	ENSG0000	SLC2A1/R
GO:00001	7	7/787	121/1972	0.209039	0.486716	1.450177	ENSG0000	DAPK1/SY
GO:00469	9	9/787	164/1972	0.209121	0.486716	1.375647	ENSG0000	STC2/BAC
GO:00551	2	2/787	22/19728	0.218336	0.502236	2.278849	ENSG0000	TRIB3/TRI
GO:00166	4	4/787	61/19728	0.225606	0.512977	1.64376	ENSG0000	PXDN/ALC
GO:00167	2	2/787	24/19728	0.248188	0.542439	2.088945	ENSG0000	GAPDH/G
GO:00483	4	4/787	64/19728	0.251645	0.545461	1.566709	ENSG0000	VLDLR/AN
GO:00305	7	7/787	129/1972	0.256376	0.552679	1.360243	ENSG0000	HRH1/GA
GO:00047	10	10/787	199/1972	0.272386	0.580845	1.259666	ENSG0000	PPP1R3C/
GO:00312	2	2/787	26/19728	0.278157	0.582369	1.928257	ENSG0000	RASGRP1/
GO:00455	2	2/787	26/19728	0.278157	0.582369	1.928257	ENSG0000	DNAH11/
GO:00168	3	3/787	47/19728	0.288734	0.596254	1.600043	ENSG0000	RIMKLA/
GO:00305	3	3/787	47/19728	0.288734	0.596254	1.600043	ENSG0000	PXDN/DK
GO:00510	6	6/787	112/1972	0.289436	0.596254	1.342893	ENSG0000	DNAJB2/H
GO:00230	4	4/787	70/19728	0.305307	0.619248	1.43242	ENSG0000	ATP1B1/P
GO:00090	6	6/787	116/1972	0.317431	0.635926	1.296587	ENSG0000	LOXL2/P4
GO:00192	6	6/787	116/1972	0.317431	0.635926	1.296587	ENSG0000	PPP2R5B/
GO:00452	1	1/787	10/19728	0.334486	0.635926	2.506734	ENSG0000	AJUBA
GO:00455	2	2/787	30/19728	0.337672	0.638904	1.671156	ENSG0000	DNAH11/
GO:00168	4	4/787	75/19728	0.350873	0.644496	1.336925	ENSG0000	ENO2/TPI
GO:00168	1	1/787	11/19728	0.361048	0.644496	2.278849	ENSG0000	TMEM86A
GO:00432	1	1/787	11/19728	0.361048	0.644496	2.278849	ENSG0000	GSTM3
GO:00519	2	2/787	32/19728	0.366905	0.649076	1.566709	ENSG0000	DNAH11/
GO:00152	1	1/787	12/19728	0.386552	0.657858	2.088945	ENSG0000	SLC6A8
GO:00466	1	1/787	12/19728	0.386552	0.657858	2.088945	ENSG0000	SPNS2
GO:00168	3	3/787	56/19728	0.387711	0.657858	1.342893	ENSG0000	ALDOA/A
GO:00718	2	2/787	35/19728	0.409756	0.672842	1.43242	ENSG0000	DDIT4/AA
GO:00167	1	1/787	13/19728	0.411039	0.672842	1.928257	ENSG0000	CYP2C9
GO:00305	1	1/787	13/19728	0.411039	0.672842	1.928257	ENSG0000	APP
GO:00426	1	1/787	13/19728	0.411039	0.672842	1.928257	ENSG0000	DOCK2
GO:00302	2	2/787	36/19728	0.423726	0.685786	1.39263	ENSG0000	PPP1R3G/
GO:00168	1	1/787	14/19728	0.43455	0.685786	1.790525	ENSG0000	PAM
GO:00397	1	1/787	14/19728	0.43455	0.685786	1.790525	ENSG0000	DKK3
GO:00198	2	2/787	37/19728	0.437521	0.685786	1.354992	ENSG0000	CYP26A1/
GO:00037	3	3/787	61/19728	0.441449	0.685786	1.23282	ENSG0000	NR5A2/PA
GO:00512	4	4/787	85/19728	0.441545	0.685786	1.17964	ENSG0000	NEDD4/S
GO:00037	11	11/787	257/1972	0.449309	0.693596	1.072921	ENSG0000	MXI1/WTI
GO:00171	2	2/787	38/19728	0.451135	0.693596	1.319334	ENSG0000	AXL/SFRP
GO:00166	3	3/787	62/19728	0.451987	0.693596	1.212936	ENSG0000	PGK1/QSC
GO:00165	1	1/787	15/19728	0.457123	0.693596	1.671156	ENSG0000	GRIN3B
GO:00704	1	1/787	15/19728	0.457123	0.693596	1.671156	ENSG0000	LOXL2
GO:00080	4	4/787	87/19728	0.459301	0.695563	1.152522	ENSG0000	CALCOCO
GO:00168	1	1/787	16/19728	0.478796	0.702175	1.566709	ENSG0000	ALOX5AP
GO:00055	1	1/787	17/19728	0.499605	0.716706	1.47455	ENSG0000	BNIP3L
GO:00162	2	2/787	42/19728	0.503634	0.719868	1.193683	ENSG0000	HSD3B7/H
GO:00433	1	1/787	18/19728	0.519584	0.729452	1.39263	ENSG0000	LRP1
GO:00700	1	1/787	18/19728	0.519584	0.729452	1.39263	ENSG0000	NEDD4

GO:00976(	1	1/787	18/19728	0.519584	0.729452	1.39263	ENSG0000	PRKN
GO:00169(	2	2/787	44/19728	0.528637	0.739529	1.139425	ENSG0000	GAPDH/AI
GO:190168	2	2/787	44/19728	0.528637	0.739529	1.139425	ENSG0000	SLC6A6/SL
GO:00167!	1	1/787	21/19728	0.574867	0.765612	1.193683	ENSG0000	F13A1
GO:00518(	1	1/787	21/19728	0.574867	0.765612	1.193683	ENSG0000	HSPA2
GO:00996(	1	1/787	21/19728	0.574867	0.765612	1.193683	ENSG0000	LYPD1
GO:00166!	5	5/787	127/19728	0.575422	0.765612	0.986903	ENSG0000	PGK1/NCF
GO:00506(	12	12/787	310/19728	0.583903	0.774285	0.970349	ENSG0000	EGLN3/PL(
GO:00051!	1	1/787	23/19728	0.608146	0.787893	1.089885	ENSG0000	GRB10
GO:00300	1	1/787	23/19728	0.608146	0.787893	1.089885	ENSG0000	PODN
GO:00511	1	1/787	23/19728	0.608146	0.787893	1.089885	ENSG0000	SLC6A8
GO:19013	1	1/787	23/19728	0.608146	0.787893	1.089885	ENSG0000	ADRA2C
GO:00171	8	8/787	212/19728	0.613743	0.791248	0.945938	ENSG0000	F3/HTRA1/
GO:00046(	1	1/787	24/19728	0.623796	0.79522	1.044473	ENSG0000	ALOX5AP
GO:01200	1	1/787	24/19728	0.623796	0.79522	1.044473	ENSG0000	ABCG1
GO:00510	2	2/787	53/19728	0.630212	0.797535	0.945938	ENSG0000	PRKAR1B/
GO:00321	4	4/787	109/19728	0.636987	0.80132	0.919903	ENSG0000	CXCR4/NE
GO:01400	6	6/787	165/19728	0.648347	0.81069	0.91154	ENSG0000	OPTN/KDI
GO:00150(	1	1/787	28/19728	0.680398	0.823365	0.895262	ENSG0000	C15orf48
GO:00192(	8	8/787	227/19728	0.688796	0.827395	0.883431	ENSG0000	PRKAR1B/
GO:00166	1	1/787	29/19728	0.693166	0.827395	0.864391	ENSG0000	MTHFD1L
GO:00166	1	1/787	29/19728	0.693166	0.827395	0.864391	ENSG0000	C15orf48
GO:00045	2	2/787	61/19728	0.705452	0.835738	0.82188	ENSG0000	MEIOB/NA
GO:00054	1	1/787	31/19728	0.717193	0.842763	0.808624	ENSG0000	STX11
GO:00152	1	1/787	31/19728	0.717193	0.842763	0.808624	ENSG0000	ABCG1
GO:00435	1	1/787	31/19728	0.717193	0.842763	0.808624	ENSG0000	AXL
GO:00971	2	2/787	63/19728	0.722145	0.844108	0.795789	ENSG0000	VIM/SCN5
GO:00167(	2	2/787	65/19728	0.738033	0.853211	0.771303	ENSG0000	GSTM3/AL
GO:00167!	4	4/787	127/19728	0.750807	0.860775	0.789523	ENSG0000	GBE1/AMY
GO:00055(	1	1/787	35/19728	0.759758	0.860775	0.71621	ENSG0000	ALOX5AP
GO:00441	1	1/787	35/19728	0.759758	0.860775	0.71621	ENSG0000	HSPA2
GO:00198(	1	1/787	40/19728	0.80408	0.897339	0.626684	ENSG0000	JCHAIN
GO:00020	2	2/787	79/19728	0.828798	0.915856	0.634616	ENSG0000	NUAK1/M
GO:00159	1	1/787	45/19728	0.840233	0.919479	0.557052	ENSG0000	SLC29A4
GO:00517	1	1/787	45/19728	0.840233	0.919479	0.557052	ENSG0000	HSPA2
GO:00192	1	1/787	46/19728	0.846621	0.92263	0.544942	ENSG0000	MACROD2
GO:00167	9	9/787	300/19728	0.850156	0.9227	0.75202	ENSG0000	NATD1/CE
GO:00166	4	4/787	149/19728	0.850193	0.9227	0.672949	ENSG0000	IDH2/HSD
GO:00310	4	4/787	149/19728	0.850193	0.9227	0.672949	ENSG0000	DNAJB2/H
GO:19015(	1	1/787	47/19728	0.852754	0.92294	0.533348	ENSG0000	SLC29A4
GO:00510	4	4/787	153/19728	0.864111	0.927541	0.655355	ENSG0000	DNAJB2/H
GO:00985	1	1/787	52/19728	0.879935	0.936935	0.482064	ENSG0000	NR5A2
GO:19908	1	1/787	58/19728	0.90602	0.955108	0.432196	ENSG0000	ZNF304
GO:00197	13	13/787	449/19728	0.91205	0.957503	0.725781	ENSG0000	NEDD4/RI
GO:00037	2	2/787	100/19728	0.912601	0.957503	0.501347	ENSG0000	MOV10L1/
GO:00167	1	1/787	72/19728	0.94695	0.977937	0.348158	ENSG0000	GAL3ST1
GO:00474	2	2/787	129/19728	0.967068	0.987085	0.388641	ENSG0000	SYNGR3/A
GO:00423	4	4/787	215/19728	0.974209	0.991808	0.466369	ENSG0000	KDM7A/D
GO:00037	7	7/787	352/19728	0.988035	1	0.498498	ENSG0000	JUN/KLF7/
GO:01010(	1	1/787	120/19728	0.992556	1	0.208895	ENSG0000	ANKZF1
GO:00314	1	1/787	123/19728	0.993417	1	0.2038	ENSG0000	KDM3A

Category
GO_BP
GO_BP
GO_BP
GO_BP
GO_BP
GO_BP
GO_BP
GO_BP
GO_BP
GO_BP
GO_BP
GO_BP
GO_BP
GO_BP
GO_BP
GO_BP
GO_BP
GO_BP
GO_BP
GO_BP
GO_BP
GO_BP
GO_BP
GO_BP
GO_BP
GO_BP
GO_BP
GO_BP
GO_BP
GO_BP
GO_BP
GO_BP
GO_BP
GO_BP
GO_BP
GO_BP
GO_BP
GO_BP
GO_BP
GO_BP
GO_BP
GO_BP
GO_BP
GO_BP
GO_BP
GO_BP
GO_BP
GO_BP
GO_BP
GO_BP
GO_BP
GO_BP
GO_BP
GO_BP
GO_BP
GO_BP
GO_BP

GO_BP
GO_BP
GO_BP
GO_BP
GO_BP
GO_BP
GO_BP
GO_BP
GO_BP
GO_BP
GO_BP
GO_BP
GO_BP
GO_BP
GO_BP
GO_BP
GO_BP
GO_BP
GO_BP
GO_BP
GO_BP
GO_BP
GO_BP
GO_BP
GO_BP
GO_BP
GO_BP
GO_BP
GO_BP
GO_BP
GO_BP
GO_BP
GO_BP
GO_BP
GO_BP
GO_BP
GO_BP
GO_BP
GO_BP
GO_BP
GO_BP
GO_BP
GO_BP
GO_BP
GO_BP
GO_BP
GO_BP
GO_BP
GO_BP
GO_BP
GO_BP
GO_BP
GO_BP
GO_BP
GO_BP
GO_BP
GO_BP
GO_BP

GO_BP
GO_BP
GO_BP
GO_BP
GO_BP
GO_BP
GO_BP
GO_BP
GO_BP
GO_BP
GO_BP
GO_BP
GO_BP
GO_BP
GO_BP
GO_BP
GO_BP
GO_BP
GO_BP
GO_BP
GO_BP
GO_BP
GO_BP
GO_BP
GO_BP
GO_BP
GO_BP
GO_BP
GO_BP
GO_BP
GO_BP
GO_BP
GO_BP
GO_BP
GO_BP
GO_BP
GO_BP
GO_BP
GO_BP
GO_BP
GO_BP
GO_BP
GO_BP
GO_BP
GO_BP
GO_BP
GO_BP
GO_BP
GO_BP
GO_BP
GO_BP
GO_BP
GO_BP
GO_BP
GO_BP
GO_BP
GO_BP
GO_BP

GO_BP
GO_BP
GO_BP
GO_BP
GO_BP
GO_BP
GO_BP
GO_BP
GO_BP
GO_BP
GO_BP
GO_BP
GO_BP
GO_BP
GO_BP
GO_BP
GO_BP
GO_BP
GO_BP
GO_BP
GO_BP
GO_BP
GO_BP
GO_BP
GO_BP
GO_BP
GO_BP
GO_BP
GO_BP
GO_BP
GO_BP
GO_BP
GO_BP
GO_BP
GO_BP
GO_BP
GO_BP
GO_BP
GO_BP
GO_BP
GO_BP
GO_BP
GO_BP
GO_BP
GO_BP
GO_BP
GO_BP
GO_BP
GO_BP
GO_BP
GO_BP
GO_BP
GO_BP
GO_BP
GO_BP
GO_BP
GO_BP
GO_BP

GO_BP
GO_BP
GO_BP
GO_BP
GO_BP
GO_BP
GO_BP
GO_BP
GO_BP
GO_BP
GO_BP
GO_BP
GO_BP
GO_BP
GO_BP
GO_BP
GO_BP
GO_BP
GO_BP
GO_BP
GO_BP
GO_BP
GO_BP
GO_BP
GO_BP
GO_BP
GO_BP
GO_BP
GO_BP
GO_BP
GO_BP
GO_BP
GO_BP
GO_BP
GO_BP
GO_BP
GO_BP
GO_BP
GO_BP
GO_BP
GO_BP
GO_BP
GO_BP
GO_BP
GO_BP
GO_BP
GO_BP
GO_BP
GO_BP
GO_BP
GO_BP
GO_BP
GO_BP
GO_BP
GO_BP
GO_BP
GO_BP
GO_BP

GO_BP
GO_BP
GO_BP
GO_BP
GO_BP
GO_BP
GO_BP
GO_BP
GO_BP
GO_BP
GO_BP
GO_BP
GO_BP
GO_BP
GO_BP
GO_BP
GO_BP
GO_BP
GO_BP
GO_BP
GO_BP
GO_BP
GO_BP
GO_BP
GO_BP
GO_BP
GO_BP
GO_BP
GO_BP
GO_BP
GO_BP
GO_BP
GO_BP
GO_BP
GO_BP
GO_BP
GO_BP
GO_BP
GO_BP
GO_BP
GO_BP
GO_BP
GO_BP
GO_BP
GO_BP
GO_BP
GO_BP
GO_BP
GO_BP
GO_BP
GO_BP
GO_BP
GO_BP
GO_BP
GO_BP
GO_BP
GO_BP
GO_BP

GO_BP
GO_BP
GO_BP
GO_BP
GO_BP
GO_BP
GO_BP
GO_BP
GO_BP
GO_BP
GO_BP
GO_BP
GO_BP
GO_BP
GO_BP
GO_BP
GO_BP
GO_BP
GO_BP
GO_BP
GO_BP
GO_BP
GO_BP
GO_BP
GO_BP
GO_BP
GO_BP
GO_BP
GO_BP
GO_BP
GO_BP
GO_BP
GO_BP
GO_BP
GO_BP
GO_BP
GO_BP
GO_BP
GO_BP
GO_BP
GO_BP
GO_BP
GO_BP
GO_BP
GO_BP
GO_BP
GO_BP
GO_BP
GO_BP
GO_BP
GO_BP
GO_BP
GO_BP
GO_BP
GO_BP
GO_BP
GO_BP
GO_BP

GO_BP
GO_BP
GO_BP
GO_BP
GO_BP
GO_BP
GO_BP
GO_BP
GO_BP
GO_BP
GO_BP
GO_BP
GO_BP
GO_BP
GO_BP
GO_BP
GO_BP
GO_BP
GO_BP
GO_BP
GO_BP
GO_BP
GO_BP
GO_BP
GO_BP
GO_BP
GO_BP
GO_BP
GO_BP
GO_BP
GO_BP
GO_BP
GO_BP
GO_BP
GO_BP
GO_BP
GO_BP
GO_BP
GO_BP
GO_BP
GO_BP
GO_BP
GO_BP
GO_BP
GO_BP
GO_BP
GO_BP
GO_BP
GO_BP
GO_BP
GO_BP
GO_BP
GO_BP
GO_BP
GO_BP
GO_BP
GO_BP
GO_BP

GO_BP
GO_BP
GO_BP
GO_BP
GO_BP
GO_BP
GO_BP
GO_BP
GO_BP
GO_BP
GO_BP
GO_BP
GO_BP
GO_BP
GO_BP
GO_BP
GO_BP
GO_BP
GO_BP
GO_BP
GO_BP
GO_BP
GO_BP
GO_BP
GO_BP
GO_BP
GO_BP
GO_BP
GO_BP
GO_BP
GO_BP
GO_BP
GO_BP
GO_BP
GO_BP
GO_BP
GO_BP
GO_BP
GO_BP
GO_BP
GO_BP
GO_BP
GO_BP
GO_BP
GO_BP
GO_BP
GO_BP
GO_BP
GO_BP
GO_BP
GO_BP
GO_BP
GO_BP
GO_BP
GO_BP
GO_BP
GO_BP
GO_BP

GO_BP
GO_BP
GO_BP
GO_BP
GO_BP
GO_BP
GO_BP
GO_BP
GO_BP
GO_BP
GO_BP
GO_BP
GO_BP
GO_BP
GO_BP
GO_BP
GO_BP
GO_BP
GO_BP
GO_BP
GO_BP
GO_BP
GO_BP
GO_BP
GO_BP
GO_BP
GO_BP
GO_BP
GO_BP
GO_BP
GO_BP
GO_BP
GO_BP
GO_BP
GO_BP
GO_BP
GO_BP
GO_BP
GO_BP
GO_BP
GO_BP
GO_BP
GO_BP
GO_BP
GO_BP
GO_BP
GO_BP
GO_BP
GO_BP
GO_BP
GO_BP
GO_BP
GO_BP
GO_BP
GO_BP
GO_BP
GO_BP
GO_BP

GO_BP
GO_BP
GO_BP
GO_BP
GO_BP
GO_BP
GO_BP
GO_BP
GO_BP
GO_BP
GO_BP
GO_BP
GO_BP
GO_BP
GO_BP
GO_BP
GO_BP
GO_BP
GO_BP
GO_BP
GO_BP
GO_BP
GO_BP
GO_BP
GO_BP
GO_BP
GO_BP
GO_BP
GO_BP
GO_BP
GO_BP
GO_BP
GO_BP
GO_BP
GO_BP
GO_BP
GO_BP
GO_BP
GO_BP
GO_BP
GO_BP
GO_BP
GO_BP
GO_BP
GO_BP
GO_BP
GO_BP
GO_BP
GO_BP
GO_BP
GO_BP
GO_BP
GO_BP
GO_BP
GO_BP
GO_BP
GO_BP
GO_BP

GO_BP
GO_BP
GO_BP
GO_BP
GO_BP
GO_BP
GO_BP
GO_BP
GO_BP
GO_BP
GO_BP
GO_BP
GO_BP
GO_BP
GO_BP
GO_BP
GO_BP
GO_BP
GO_BP
GO_BP
GO_BP
GO_BP
GO_BP
GO_BP
GO_BP
GO_BP
GO_BP
GO_BP
GO_BP
GO_BP
GO_BP
GO_BP
GO_BP
GO_BP
GO_BP
GO_BP
GO_BP
GO_BP
GO_BP
GO_BP
GO_BP
GO_BP
GO_BP
GO_BP
GO_BP
GO_BP
GO_BP
GO_BP
GO_BP
GO_BP
GO_BP
GO_BP
GO_BP
GO_BP
GO_BP
GO_BP
GO_BP
GO_BP

GO_BP
GO_BP
GO_BP
GO_BP
GO_BP
GO_BP
GO_BP
GO_BP
GO_BP
GO_BP
GO_BP
GO_BP
GO_BP
GO_BP
GO_BP
GO_BP
GO_BP
GO_BP
GO_BP
GO_BP
GO_BP
GO_BP
GO_BP
GO_BP
GO_BP
GO_BP
GO_BP
GO_BP
GO_BP
GO_BP
GO_BP
GO_BP
GO_BP
GO_BP
GO_BP
GO_BP
GO_BP
GO_BP
GO_BP
GO_BP
GO_BP
GO_BP
GO_BP
GO_BP
GO_BP
GO_BP
GO_BP
GO_BP
GO_BP
GO_BP
GO_BP
GO_BP
GO_BP
GO_BP
GO_BP
GO_BP
GO_BP
GO_BP

GO_BP
GO_BP
GO_BP
GO_BP
GO_BP
GO_BP
GO_BP
GO_BP
GO_BP
GO_BP
GO_BP
GO_BP
GO_BP
GO_BP
GO_BP
GO_BP
GO_BP
GO_BP
GO_BP
GO_BP
GO_BP
GO_BP
GO_BP
GO_BP
GO_BP
GO_BP
GO_BP
GO_BP
GO_BP
GO_BP
GO_BP
GO_BP
GO_BP
GO_BP
GO_BP
GO_BP
GO_BP
GO_BP
GO_BP
GO_BP
GO_BP
GO_BP
GO_BP
GO_BP
GO_BP
GO_BP
GO_BP
GO_BP
GO_BP
GO_BP
GO_BP
GO_BP
GO_BP
GO_BP
GO_BP
GO_BP
GO_BP
GO_BP

GO_BP
GO_BP
GO_BP
GO_BP
GO_BP
GO_BP
GO_BP
GO_BP
GO_BP
GO_BP
GO_BP
GO_BP
GO_BP
GO_BP
GO_BP
GO_BP
GO_BP
GO_BP
GO_BP
GO_BP
GO_BP
GO_BP
GO_BP
GO_BP
GO_BP
GO_BP
GO_BP
GO_BP
GO_BP
GO_BP
GO_BP
GO_BP
GO_BP
GO_BP
GO_BP
GO_BP
GO_BP
GO_BP
GO_BP
GO_BP
GO_BP
GO_BP
GO_BP
GO_BP
GO_BP
GO_BP
GO_BP
GO_BP
GO_BP
GO_BP
GO_BP
GO_BP
GO_BP
GO_BP
GO_BP
GO_BP
GO_BP
GO_BP

GO_BP
GO_BP
GO_BP
GO_BP
GO_CC
GO_CC
GO_CC
GO_CC
GO_CC
GO_CC
GO_CC
GO_CC
GO_CC
GO_CC
GO_CC
GO_CC
GO_CC
GO_CC
GO_CC
GO_CC
GO_CC
GO_CC
GO_CC
GO_CC
GO_CC
GO_CC
GO_CC
GO_CC
GO_CC
GO_CC
GO_CC
GO_CC
GO_CC
GO_CC
GO_CC
GO_CC
GO_CC
GO_CC
GO_CC
GO_CC
GO_CC
GO_CC
GO_CC
GO_CC
GO_CC
GO_CC
GO_CC
GO_CC
GO_CC
GO_CC
GO_CC
GO_CC
GO_CC
GO_CC
GO_CC
GO_CC
GO_CC
GO_CC

GO_CC
GO_CC
GO_CC
GO_CC
GO_CC
GO_CC
GO_CC
GO_CC
GO_CC
GO_CC
GO_CC
GO_CC
GO_CC
GO_CC
GO_CC
GO_CC
GO_CC
GO_CC
GO_CC
GO_CC
GO_CC
GO_CC
GO_CC
GO_CC
GO_CC
GO_CC
GO_CC
GO_CC
GO_CC
GO_CC
GO_CC
GO_CC
GO_CC
GO_CC
GO_CC
GO_CC
GO_CC
GO_CC
GO_CC
GO_CC
GO_CC
GO_CC
GO_CC
GO_CC
GO_CC
GO_CC
GO_CC
GO_CC
GO_CC
GO_CC
GO_CC
GO_CC
GO_CC
GO_CC
GO_CC
GO_CC
GO_CC
GO_CC

GO_CC
GO_CC
GO_CC
GO_CC
GO_CC
GO_CC
GO_CC
GO_CC
GO_CC
GO_CC
GO_CC
GO_CC
GO_CC
GO_CC
GO_CC
GO_CC
GO_CC
GO_CC
GO_CC
GO_CC
GO_CC
GO_CC
GO_CC
GO_CC
GO_CC
GO_CC
GO_CC
GO_CC
GO_CC
GO_CC
GO_CC
GO_CC
GO_CC
GO_CC
GO_CC
GO_CC
GO_CC
GO_CC
GO_CC
GO_CC
GO_CC
GO_CC
GO_CC
GO_CC
GO_CC
GO_CC
GO_CC
GO_CC
GO_CC
GO_CC
GO_CC
GO_CC
GO_CC
GO_CC
GO_CC
GO_CC
GO_CC
GO_CC

GO_CC
GO_CC
GO_CC
GO_CC
GO_CC
GO_CC
GO_CC
GO_CC
GO_CC
GO_CC
GO_CC
GO_CC
GO_CC
GO_CC
GO_CC
GO_CC
GO_CC
GO_CC
GO_CC
GO_CC
GO_CC
GO_CC
GO_CC
GO_CC
GO_CC
GO_CC
GO_CC
GO_CC
GO_CC
GO_CC
GO_CC
GO_CC
GO_CC
GO_CC
GO_CC
GO_CC
GO_CC
GO_CC
GO_CC
GO_CC
GO_CC
GO_CC
GO_CC
GO_MF
GO_MF
GO_MF
GO_MF
GO_MF
GO_MF
GO_MF
GO_MF
GO_MF
GO_MF
GO_MF
GO_MF
GO_MF
GO_MF
GO_MF

GO_MF
GO_MF
GO_MF
GO_MF
GO_MF
GO_MF
GO_MF
GO_MF
GO_MF
GO_MF
GO_MF
GO_MF
GO_MF
GO_MF
GO_MF
GO_MF
GO_MF
GO_MF
GO_MF
GO_MF
GO_MF
GO_MF
GO_MF
GO_MF
GO_MF
GO_MF
GO_MF
GO_MF
GO_MF
GO_MF
GO_MF
GO_MF
GO_MF
GO_MF
GO_MF
GO_MF
GO_MF
GO_MF
GO_MF
GO_MF
GO_MF
GO_MF
GO_MF
GO_MF
GO_MF
GO_MF
GO_MF
GO_MF
GO_MF
GO_MF
GO_MF
GO_MF
GO_MF
GO_MF
GO_MF
GO_MF
GO_MF
GO_MF

GO_MF
GO_MF
GO_MF
GO_MF
GO_MF
GO_MF
GO_MF
GO_MF
GO_MF
GO_MF
GO_MF
GO_MF
GO_MF
GO_MF
GO_MF
GO_MF
GO_MF
GO_MF
GO_MF
GO_MF
GO_MF
GO_MF
GO_MF
GO_MF
GO_MF
GO_MF
GO_MF
GO_MF
GO_MF
GO_MF
GO_MF
GO_MF
GO_MF
GO_MF
GO_MF
GO_MF
GO_MF
GO_MF
GO_MF
GO_MF
GO_MF
GO_MF
GO_MF
GO_MF
GO_MF
GO_MF
GO_MF
GO_MF
GO_MF
GO_MF
GO_MF
GO_MF
GO_MF
GO_MF
GO_MF
GO_MF
GO_MF
GO_MF

GO_MF
GO_MF
GO_MF
GO_MF
GO_MF
GO_MF
GO_MF
GO_MF
GO_MF
GO_MF
GO_MF
GO_MF
GO_MF
GO_MF
GO_MF
GO_MF
GO_MF
GO_MF
GO_MF
GO_MF
GO_MF
GO_MF
GO_MF
GO_MF
GO_MF
GO_MF
GO_MF
GO_MF
GO_MF
GO_MF
GO_MF
GO_MF
GO_MF
GO_MF
GO_MF
GO_MF
GO_MF
GO_MF
GO_MF
GO_MF
GO_MF
GO_MF
GO_MF
GO_MF
GO_MF
GO_MF
GO_MF
GO_MF
GO_MF
GO_MF
GO_MF

Supplementary Table S4

Terms	Counts	GeneRatio	BgRatio	pValue	FDR	foldEnrichı	geneID	geneSymb
hsa00051~	10	10/337	33/8031	5.79E-07	8.58E-05	7.221473		
hsa00010~	14	14/337	68/8031	6.31E-07	8.58E-05	4.906354		
hsa01230~	14	14/337	75/8031	2.20E-06	0.00017	4.448427		
hsa04066~	17	17/337	109/8031	2.49E-06	0.00017	3.71674		
hsa04510~	21	21/337	201/8031	9.97E-05	0.004701	2.489791		
hsa01200~	15	15/337	117/8031	0.000104	0.004701	3.055239		
hsa05230~	11	11/337	69/8031	0.000124	0.004808	3.799123		
hsa04974~	13	13/337	95/8031	0.000154	0.005228	3.261065		
hsa04512~	11	11/337	88/8031	0.001067	0.032233	2.978858		
hsa04976~	11	11/337	90/8031	0.001287	0.034999	2.912661		
hsa04964~	5	5/337	23/8031	0.002275	0.056245	5.180622		
hsa00500~	6	6/337	36/8031	0.0035	0.079327	3.97181		
hsa04727~	10	10/337	89/8031	0.00395	0.082639	2.677625		
hsa04022~	15	15/337	167/8031	0.004325	0.084019	2.140496		
hsa05418~	13	13/337	139/8031	0.005474	0.094533	2.228786		
hsa05205~	17	17/337	205/8031	0.005561	0.094533	1.976218		
hsa04151~	25	25/337	354/8031	0.007261	0.108446	1.68297		
hsa04137~	8	8/337	68/8031	0.007272	0.108446	2.803631		
hsa00030~	5	5/337	30/8031	0.007575	0.108446	3.97181		
hsa04978~	7	7/337	58/8031	0.010274	0.139728	2.876138		
hsa04270~	12	12/337	135/8031	0.011027	0.141077	2.118299		
hsa05165~	23	23/337	330/8031	0.011411	0.141077	1.660939		
hsa04918~	8	8/337	75/8031	0.0129	0.148746	2.541958		
hsa04973~	6	6/337	47/8031	0.013125	0.148746	3.042238		
hsa05412~	8	8/337	77/8031	0.014974	0.162923	2.475934		
hsa04080~	23	23/337	340/8031	0.015845	0.165758	1.612088		
hsa05144~	6	6/337	50/8031	0.017498	0.176278	2.859703		
hsa05414~	9	9/337	96/8031	0.019007	0.184636	2.234143		
hsa04925~	9	9/337	98/8031	0.02146	0.201278	2.188548		
hsa04961~	6	6/337	53/8031	0.022781	0.206549	2.697833		
hsa04933~	9	9/337	100/8031	0.024133	0.211746	2.144777		
hsa04972~	9	9/337	102/8031	0.027035	0.225569	2.102723		
hsa04911~	8	8/337	86/8031	0.027367	0.225569	2.216824		
hsa04020~	14	14/337	193/8031	0.032179	0.257435	1.728663		
hsa04014~	16	16/337	232/8031	0.034585	0.266698	1.643508		
hsa04810~	15	15/337	214/8031	0.035375	0.266698	1.670387		
hsa04970~	8	8/337	91/8031	0.036687	0.266698	2.095021		
hsa04024~	15	15/337	216/8031	0.037931	0.266698	1.654921		
hsa02010~	5	5/337	45/8031	0.039066	0.266698	2.647873		
hsa00052~	4	4/337	31/8031	0.03922	0.266698	3.07495		
hsa04360~	13	13/337	181/8031	0.040786	0.270579	1.711609		
hsa04261~	11	11/337	149/8031	0.04844	0.313704	1.759325		
hsa04145~	11	11/337	152/8031	0.054497	0.344725	1.724602		
hsa04015~	14	14/337	210/8031	0.058166	0.355445	1.588724		
hsa04921~	11	11/337	154/8031	0.058805	0.355445	1.702204		
hsa04919~	9	9/337	121/8031	0.067503	0.397678	1.772543		
hsa04010~	18	18/337	294/8031	0.068716	0.397678	1.459032		
hsa04540~	7	7/337	88/8031	0.075712	0.420608	1.895637		
hsa04922~	8	8/337	106/8031	0.076641	0.420608	1.798556		
hsa04977~	3	3/337	24/8031	0.077318	0.420608	2.978858		
hsa05410~	7	7/337	90/8031	0.083151	0.443473	1.853511		
hsa04216~	4	4/337	41/8031	0.091548	0.464304	2.324962		
hsa05219~	4	4/337	41/8031	0.091548	0.464304	2.324962		
hsa04926~	9	9/337	129/8031	0.092178	0.464304	1.662618		
hsa00230~	9	9/337	130/8031	0.095592	0.467988	1.649829		
hsa04971~	6	6/337	76/8031	0.098758	0.467988	1.881384		
hsa04650~	9	9/337	131/8031	0.099078	0.467988	1.637235		

hsa04062~	12	12/337	189/8031	0.099791	0.467988	1.513071
hsa04670~	8	8/337	113/8031	0.101782	0.469234	1.687141
hsa04940~	4	4/337	43/8031	0.104694	0.471369	2.216824
hsa04724~	8	8/337	114/8031	0.105711	0.471369	1.672341
hsa04934~	10	10/337	155/8031	0.116238	0.50877	1.537475
hsa04392~	3	3/337	29/8031	0.12011	0.50877	2.465261
hsa04371~	9	9/337	137/8031	0.12151	0.50877	1.565531
hsa04640~	7	7/337	99/8031	0.121581	0.50877	1.68501
hsa05217~	5	5/337	63/8031	0.123763	0.510054	1.891338
hsa00604~	2	2/337	15/8031	0.128782	0.51713	3.177448
hsa00020~	3	3/337	30/8031	0.129535	0.51713	2.383086
hsa04916~	7	7/337	101/8031	0.131184	0.51713	1.651644
hsa00600~	4	4/337	47/8031	0.133355	0.51818	2.028158
hsa00520~	4	4/337	48/8031	0.140977	0.540081	1.985905
hsa04610~	6	6/337	85/8031	0.146043	0.551719	1.682178
hsa04672~	4	4/337	49/8031	0.148768	0.554312	1.945376
hsa05211~	5	5/337	69/8031	0.162777	0.589564	1.726874
hsa05224~	9	9/337	147/8031	0.164419	0.589564	1.459032
hsa04913~	4	4/337	51/8031	0.164827	0.589564	1.869087
hsa04931~	7	7/337	108/8031	0.167602	0.589564	1.544593
hsa04514~	9	9/337	148/8031	0.169066	0.589564	1.449174
hsa05032~	6	6/337	91/8031	0.182328	0.627381	1.571266
hsa05222~	6	6/337	92/8031	0.1887	0.627381	1.554187
hsa00250~	3	3/337	36/8031	0.190805	0.627381	1.985905
hsa04115~	5	5/337	73/8031	0.191193	0.627381	1.632251
hsa04912~	6	6/337	93/8031	0.195157	0.627381	1.537475
hsa05323~	6	6/337	93/8031	0.195157	0.627381	1.537475
hsa04725~	7	7/337	113/8031	0.196057	0.627381	1.476248
hsa04960~	3	3/337	37/8031	0.201655	0.63046	1.932232
hsa05143~	3	3/337	37/8031	0.201655	0.63046	1.932232
hsa04923~	4	4/337	56/8031	0.207422	0.63552	1.702204
hsa04726~	7	7/337	115/8031	0.207946	0.63552	1.450574
hsa04390~	9	9/337	157/8031	0.213512	0.636882	1.3661
hsa00220~	2	2/337	21/8031	0.219568	0.636882	2.269606
hsa04915~	8	8/337	138/8031	0.22281	0.636882	1.381499
hsa00620~	3	3/337	39/8031	0.223761	0.636882	1.833143
hsa01522~	6	6/337	98/8031	0.228618	0.636882	1.459032
hsa04612~	5	5/337	78/8031	0.228939	0.636882	1.527619
hsa04721~	5	5/337	78/8031	0.228939	0.636882	1.527619
hsa04310~	9	9/337	160/8031	0.229281	0.636882	1.340486
hsa04935~	7	7/337	119/8031	0.2325	0.636882	1.401815
hsa05033~	3	3/337	40/8031	0.234988	0.636882	1.787315
hsa01521~	5	5/337	79/8031	0.236739	0.636882	1.508282
hsa04152~	7	7/337	120/8031	0.238787	0.636882	1.390134
hsa04061~	6	6/337	100/8031	0.242493	0.636882	1.429852
hsa04750~	6	6/337	100/8031	0.242493	0.636882	1.429852
hsa05416~	4	4/337	60/8031	0.243514	0.636882	1.588724
hsa05332~	3	3/337	41/8031	0.246313	0.638067	1.743722
hsa00140~	4	4/337	61/8031	0.252754	0.648576	1.562679
hsa05146~	6	6/337	102/8031	0.25661	0.649072	1.401815
hsa00380~	3	3/337	42/8031	0.25772	0.649072	1.702204
hsa04213~	4	4/337	62/8031	0.262066	0.653939	1.537475
hsa04611~	7	7/337	124/8031	0.264461	0.653939	1.345291
hsa05225~	9	9/337	168/8031	0.273275	0.669647	1.276653
hsa04929~	4	4/337	64/8031	0.280876	0.678525	1.489429
hsa04072~	8	8/337	148/8031	0.281887	0.678525	1.288155
hsa04928~	6	6/337	106/8031	0.28547	0.680844	1.348917
hsa05226~	8	8/337	149/8031	0.288021	0.680844	1.279509

hsa04927~	4	4/337	65/8031	0.29036	0.680844	1.466514
hsa04950~	2	2/337	26/8031	0.298383	0.693676	1.833143
hsa04930~	3	3/337	46/8031	0.303917	0.700554	1.554187
hsa04068~	7	7/337	131/8031	0.311043	0.710957	1.273405
hsa00565~	3	3/337	47/8031	0.315546	0.71483	1.521119
hsa05235~	5	5/337	89/8031	0.317994	0.71483	1.338812
hsa04150~	8	8/337	155/8031	0.325467	0.725632	1.22998
hsa04744~	2	2/337	28/8031	0.329713	0.729121	1.702204
hsa05030~	3	3/337	49/8031	0.33882	0.731113	1.459032
hsa04210~	7	7/337	136/8031	0.345222	0.731113	1.226588
hsa00591~	2	2/337	29/8031	0.345239	0.731113	1.643508
hsa00330~	3	3/337	50/8031	0.350443	0.731113	1.429852
hsa04979~	3	3/337	50/8031	0.350443	0.731113	1.429852
hsa04060~	14	14/337	294/8031	0.351436	0.731113	1.134803
hsa04666~	5	5/337	93/8031	0.351516	0.731113	1.281229
hsa04140~	7	7/337	137/8031	0.352117	0.731113	1.217635
hsa04910~	7	7/337	139/8031	0.365948	0.754075	1.200115
hsa04071~	6	6/337	119/8031	0.382784	0.78178	1.201556
hsa05320~	3	3/337	53/8031	0.385142	0.78178	1.348917
hsa04215~	2	2/337	32/8031	0.391037	0.787868	1.489429
hsa05167~	9	9/337	189/8031	0.397381	0.794762	1.134803
hsa00980~	4	4/337	77/8031	0.405684	0.799609	1.237967
hsa03320~	4	4/337	77/8031	0.405684	0.799609	1.237967
hsa00983~	4	4/337	79/8031	0.424758	0.820628	1.206626
hsa03018~	4	4/337	79/8031	0.424758	0.820628	1.206626
hsa05206~	14	14/337	310/8031	0.427787	0.820628	1.076232
hsa04723~	7	7/337	148/8031	0.428416	0.820628	1.127135
hsa04380~	6	6/337	128/8031	0.450717	0.85567	1.117072
hsa04370~	3	3/337	59/8031	0.453002	0.85567	1.211739
hsa05204~	4	4/337	83/8031	0.462407	0.867411	1.148475
hsa05163~	10	10/337	225/8031	0.472544	0.877909	1.059149
hsa00603~	1	1/337	15/8031	0.474601	0.877909	1.588724
hsa05330~	2	2/337	38/8031	0.477872	0.877909	1.254256
hsa04012~	4	4/337	85/8031	0.480913	0.877909	1.121452
hsa00590~	3	3/337	63/8031	0.496497	0.893247	1.134803
hsa00730~	1	1/337	16/8031	0.496689	0.893247	1.489429
hsa04260~	4	4/337	87/8031	0.499167	0.893247	1.095672
hsa05145~	5	5/337	112/8031	0.509001	0.904891	1.063878
hsa00120~	1	1/337	17/8031	0.517851	0.908746	1.401815
hsa00910~	1	1/337	17/8031	0.517851	0.908746	1.401815
hsa04630~	7	7/337	162/8031	0.523589	0.912925	1.029729
hsa01210~	1	1/337	18/8031	0.538126	0.924344	1.323937
hsa00830~	3	3/337	67/8031	0.538198	0.924344	1.067053
hsa04664~	3	3/337	68/8031	0.548313	0.924344	1.051361
hsa05223~	3	3/337	68/8031	0.548313	0.924344	1.051361
hsa04962~	2	2/337	44/8031	0.556757	0.924344	1.083221
hsa00770~	1	1/337	19/8031	0.557551	0.924344	1.254256
hsa04924~	3	3/337	69/8031	0.558297	0.924344	1.036124
hsa04350~	4	4/337	94/8031	0.560697	0.924344	1.014079
hsa04722~	5	5/337	119/8031	0.563395	0.924344	1.001297
hsa05131~	10	10/337	242/8031	0.566496	0.924344	0.984746
hsa05120~	3	3/337	70/8031	0.568148	0.924344	1.021323
hsa05135~	5	5/337	120/8031	0.570919	0.924344	0.992953
hsa00670~	1	1/337	20/8031	0.576162	0.927313	1.191543
hsa04713~	4	4/337	97/8031	0.585787	0.92772	0.982716
hsa00982~	3	3/337	72/8031	0.587441	0.92772	0.992953
hsa05218~	3	3/337	72/8031	0.587441	0.92772	0.992953
hsa00514~	2	2/337	47/8031	0.592911	0.92772	1.014079

hsa05231~	4	4/337	98/8031	0.593965	0.92772	0.972688
hsa05100~	3	3/337	73/8031	0.596879	0.92772	0.97935
hsa05214~	3	3/337	75/8031	0.615328	0.941967	0.953234
hsa05133~	3	3/337	76/8031	0.624337	0.941967	0.940692
hsa05212~	3	3/337	76/8031	0.624337	0.941967	0.940692
hsa05220~	3	3/337	76/8031	0.624337	0.941967	0.940692
hsa05142~	4	4/337	102/8031	0.625701	0.941967	0.934544
hsa05110~	2	2/337	50/8031	0.626824	0.941967	0.953234
hsa05140~	3	3/337	77/8031	0.633202	0.946036	0.928475
hsa04714~	9	9/337	231/8031	0.638892	0.946036	0.928475
hsa05132~	8	8/337	206/8031	0.639966	0.946036	0.92547
hsa04728~	5	5/337	132/8031	0.655575	0.963873	0.902684
hsa05017~	5	5/337	133/8031	0.662129	0.968274	0.895897
hsa00790~	1	1/337	26/8031	0.672527	0.968487	0.916572
hsa04530~	6	6/337	161/8031	0.674603	0.968487	0.888107
hsa04662~	3	3/337	82/8031	0.675328	0.968487	0.871861
hsa00601~	1	1/337	27/8031	0.686314	0.968487	0.882624
hsa04966~	1	1/337	27/8031	0.686314	0.968487	0.882624
hsa05162~	5	5/337	138/8031	0.693674	0.968487	0.863437
hsa00240~	2	2/337	57/8031	0.697362	0.968487	0.836171
hsa00480~	2	2/337	57/8031	0.697362	0.968487	0.836171
hsa05134~	2	2/337	57/8031	0.697362	0.968487	0.836171
hsa04668~	4	4/337	112/8031	0.69788	0.968487	0.851102
hsa04120~	5	5/337	140/8031	0.705715	0.97321	0.851102
hsa04141~	6	6/337	167/8031	0.708439	0.97321	0.856199
hsa04144~	9	9/337	248/8031	0.72028	0.983489	0.86483
hsa04550~	5	5/337	143/8031	0.723153	0.983489	0.833247
hsa00410~	1	1/337	31/8031	0.735908	0.990926	0.768737
hsa04710~	1	1/337	31/8031	0.735908	0.990926	0.768737
hsa04136~	1	1/337	32/8031	0.747033	0.99663	0.744714
hsa04658~	3	3/337	92/8031	0.748646	0.99663	0.777093
hsa03410~	1	1/337	33/8031	0.757691	0.99663	0.722147
hsa04130~	1	1/337	33/8031	0.757691	0.99663	0.722147
hsa05321~	2	2/337	65/8031	0.764102	0.99663	0.733257
hsa00040~	1	1/337	34/8031	0.7679	0.99663	0.700908
hsa05150~	3	3/337	96/8031	0.774024	0.99663	0.744714
hsa04720~	2	2/337	67/8031	0.778659	0.99663	0.711369
hsa05221~	2	2/337	67/8031	0.778659	0.99663	0.711369
hsa00564~	3	3/337	98/8031	0.785908	0.99663	0.729516
hsa04114~	4	4/337	128/8031	0.791612	0.99663	0.744714
hsa04920~	2	2/337	69/8031	0.792426	0.99663	0.69075
hsa05031~	2	2/337	69/8031	0.792426	0.99663	0.69075
hsa05216~	1	1/337	37/8031	0.796027	0.99663	0.644077
hsa04917~	2	2/337	70/8031	0.799022	0.99663	0.680882
hsa05340~	1	1/337	38/8031	0.804626	0.99663	0.627128
hsa04520~	2	2/337	71/8031	0.805432	0.99663	0.671292
hsa04218~	5	5/337	160/8031	0.808011	0.99663	0.744714
hsa04064~	3	3/337	104/8031	0.81849	0.99663	0.687429
hsa04660~	3	3/337	104/8031	0.81849	0.99663	0.687429
hsa05166~	7	7/337	219/8031	0.819502	0.99663	0.761717
hsa00260~	1	1/337	40/8031	0.820754	0.99663	0.595772
hsa00860~	1	1/337	42/8031	0.835555	1	0.567401
hsa04975~	1	1/337	43/8031	0.842492	1	0.554206
hsa05169~	6	6/337	201/8031	0.85436	1	0.711369
hsa05010~	12	12/337	369/8031	0.856848	1	0.774987
hsa04742~	2	2/337	83/8031	0.869269	1	0.574238
hsa00280~	1	1/337	48/8031	0.873036	1	0.496476
hsa05152~	5	5/337	180/8031	0.879935	1	0.661968

hsa05210~	2	2/337	86/8031	0.881907	1	0.554206
hsa04621~	5	5/337	181/8031	0.882837	1	0.658311
hsa04211~	2	2/337	89/8031	0.893409	1	0.535525
hsa04330~	1	1/337	53/8031	0.897671	1	0.449639
hsa04142~	3	3/337	128/8031	0.909864	1	0.558536
hsa04657~	2	2/337	94/8031	0.910296	1	0.50704
hsa05202~	5	5/337	192/8031	0.910993	1	0.620595
hsa05014~	1	1/337	57/8031	0.913899	1	0.418085
hsa05213~	1	1/337	58/8031	0.917538	1	0.410877
hsa05215~	2	2/337	97/8031	0.919192	1	0.491358
hsa04730~	1	1/337	60/8031	0.924362	1	0.397181
hsa04914~	2	2/337	99/8031	0.924656	1	0.481432
hsa00310~	1	1/337	61/8031	0.92756	1	0.39067
hsa00561~	1	1/337	61/8031	0.92756	1	0.39067
hsa04623~	1	1/337	63/8031	0.933557	1	0.378268
hsa05203~	5	5/337	204/8031	0.934793	1	0.58409
hsa04620~	2	2/337	104/8031	0.936832	1	0.458286
hsa04625~	2	2/337	104/8031	0.936832	1	0.458286
hsa04659~	2	2/337	107/8031	0.943219	1	0.445437
hsa05170~	5	5/337	212/8031	0.94733	1	0.562049
hsa04622~	1	1/337	70/8031	0.950906	1	0.340441
hsa05012~	6	6/337	249/8031	0.953976	1	0.574238
hsa01524~	1	1/337	73/8031	0.956882	1	0.32645
hsa00562~	1	1/337	74/8031	0.958707	1	0.322039
hsa05160~	3	3/337	155/8031	0.961394	1	0.461242
hsa05130~	4	4/337	192/8031	0.963729	1	0.496476
hsa05161~	3	3/337	162/8031	0.969273	1	0.441312
hsa04146~	1	1/337	83/8031	0.972034	1	0.287119
hsa05164~	3	3/337	170/8031	0.976416	1	0.420545
hsa03015~	1	1/337	91/8031	0.980229	1	0.261878
hsa04070~	1	1/337	99/8031	0.986027	1	0.240716
hsa05034~	3	3/337	187/8031	0.986717	1	0.382313
hsa03040~	2	2/337	149/8031	0.987881	1	0.319877
hsa04932~	2	2/337	149/8031	0.987881	1	0.319877
hsa05016~	6	6/337	306/8031	0.990382	1	0.467272
hsa00190~	1	1/337	133/8031	0.996817	1	0.179179
hsa05322~	1	1/337	136/8031	0.997207	1	0.175227
hsa04217~	1	1/337	159/8031	0.998977	1	0.14988
hsa05168~	9	9/337	491/8031	0.999084	1	0.436818
hsa03013~	1	1/337	180/8031	0.999593	1	0.132394
hsa04740~	2	2/337	448/8031	1	1	0.106388

ol
