# Supplementary material for: A Novel Hypoxia-Related Gene Signature with Strong Predicting Ability in Non-Small-Cell Lung Cancer Identified by Comprehensive Profiling
Source: Int J Genomics. 2022 May 19;2022:8594658. doi: 10.1155/2022/8594658 (PMC9135579; doi:10.1155/2022/8594658)
Supplement: Supplementary Materials — Supplementary Figure S1: the expressions ofCNKSR3, DGAT2, FAMB1A, SERPINE1, TGFB1, and TMEM132B in the ceRNA network showed no significant difference between NSCLC and normal samples. Supplementary Figure S2: no significant difference of survival between groups divided by gender or expression of ADM, BHLHE40, BIRC5, C1QL1, C11orf86, CCNA2, CCND3, CNKSR3, DKK1, DKK3, DGAT2, ETV1, FAM81A, FAM160A1, HECA, HMGA2, HOXC8, ISOC1, KDM7A, NECTIN1, HNRNPA2B1, PAM, PEA15, PPIH, PPP1R3B, RASGEF1B, SLC12A2, ZWILCH, WSB1, TMEM132B, or STC2. Supplementary Table S1: identification of 1293 upregulated DEGs and 746 downregulated DEGs in hypoxia-treated A549 cells compared to normoxia-treated A549 cells displayed in the heat map. Supplementary Table S2: 21 upregulated DEcircRNAs and 49 downregulated DEcircRNAs identified in hypoxia-treated A549 cells compared to normoxia-treated A549 cells. Supplementary Table S3 and S4: upregulated DEGs were significantly enriched into 284 GO terms and 42 KEGG pathways. Supplementary Table S5 and S6: downregulated DEGs were significantly enriched into 184 GO terms and 25 KEGG pathways. [file 8594658.f1.zip › Supplementary Table S5_mRNA_Down_AllenrichGO and S6_mRNA_Down_AllEnrichKEGG.pdf]

Supplementary Table S5 and S6. Down-regulated DEGs were significantly enriched into 184 GO terms and 25 KEGG pathways.

Supplementary Table S5

Terms	Counts	GeneRatio	BgRatio	pValue	FDR	foldEnrichı	geneID	geneSymb
GO:00070!	35	35/545	193/2061C	1.53E-19	7.43E-17	6.857917	ENSG0000	PCNA/CLS
GO:00447!	22	22/545	72/20610	6.74E-18	1.96E-15	11.55505	ENSG0000	PCNA/POI
GO:00988!	39	39/545	278/2061C	1.40E-17	3.81E-15	5.305194	ENSG0000	RRS1/PLK1
GO:00315	32	32/545	188/2061C	3.96E-17	9.10E-15	6.436853	ENSG0000	PCNA/CLS
GO:00109	42	42/545	424/2061C	2.02E-13	3.04E-11	3.745975	ENSG0000	PCNA/CLS
GO:00900	36	36/545	319/2061C	2.35E-13	3.33E-11	4.267694	ENSG0000	PCNA/AUI
GO:00457!	42	42/545	426/2061C	2.37E-13	3.33E-11	3.728389	ENSG0000	PCNA/AUI
GO:00723!	19	19/545	83/20610	4.12E-13	5.29E-11	8.656792	ENSG0000	PCNA/PLK
GO:00513	33	33/545	275/2061C	4.29E-13	5.35E-11	4.537982	ENSG0000	EXO1/PLK
GO:01400	27	27/545	193/2061C	1.57E-12	1.68E-10	5.290393	ENSG0000	PLK1/AUR
GO:19030	28	28/545	209/2061C	1.78E-12	1.86E-10	5.066327	ENSG0000	PLK1/AUR
GO:20000	13	13/545	40/20610	1.68E-11	1.37E-09	12.29037	ENSG0000	TP63/HM(
GO:00070!	26	26/545	202/2061C	2.80E-11	2.11E-09	4.867472	ENSG0000	PLK1/AUR
GO:00513(	17	17/545	93/20610	3.17E-10	2.04E-08	6.912696	ENSG0000	PLK1/CDC
GO:00451	17	17/545	99/20610	8.77E-10	5.55E-08	6.493745	ENSG0000	PLK1/SGO
GO:00070!	26	26/545	255/2061C	4.67E-09	2.68E-07	3.855801	ENSG0000	PCNA/DDI
GO:00198	21	21/545	180/2061C	1.36E-08	7.31E-07	4.411927	ENSG0000	TP63/TBX3
GO:00519!	16	16/545	106/2061C	1.88E-08	9.68E-07	5.708153	ENSG0000	PLK1/CDC
GO:00322(	9	9/545	28/20610	2.61E-08	1.30E-06	12.15531	ENSG0000	PCNA/POI
GO:00018	11	11/545	48/20610	3.72E-08	1.81E-06	8.666284	ENSG0000	CNOT3/N/
GO:00315	9	9/545	34/20610	1.72E-07	6.77E-06	10.01025	ENSG0000	PLK1/CDC
GO:00070(	11	11/545	61/20610	5.05E-07	1.82E-05	6.819371	ENSG0000	PLK1/CDC
GO:01201(	14	14/545	109/2061C	1.11E-06	3.81E-05	4.857167	ENSG0000	ELOVL6/D
GO:00086(	8	8/545	34/20610	2.25E-06	7.33E-05	8.898003	ENSG0000	SPAG5/CC
GO:00519!	9	9/545	46/20610	2.73E-06	8.75E-05	7.398883	ENSG0000	PLK1/CDC
GO:00619!	15	15/545	137/2061C	3.57E-06	0.00011	4.140494	ENSG0000	PLK1/AUR
GO:00070!	14	14/545	132/2061C	1.09E-05	0.000302	4.010842	ENSG0000	PLK1/AUR
GO:00018	13	13/545	119/2061C	1.62E-05	0.000426	4.131216	ENSG0000	NEK2/BRC
GO:00701!	10	10/545	73/20610	2.14E-05	0.000544	5.180344	ENSG0000	SGO2/BUE
GO:00310	14	14/545	141/2061C	2.31E-05	0.000576	3.754831	ENSG0000	PLK1/AUR
GO:00071	7	7/545	35/20610	3.07E-05	0.000763	7.563303	ENSG0000	PLK1/AUR
GO:00071	9	9/545	63/20610	3.93E-05	0.000964	5.402359	ENSG0000	TRIP13/MI
GO:00513(	6	6/545	27/20610	6.13E-05	0.00144	8.40367	ENSG0000	TTK/NCAP
GO:01061(	14	14/545	156/2061C	7.08E-05	0.001645	3.39379	ENSG0000	ELOVL6/D
GO:01201(	14	14/545	156/2061C	7.08E-05	0.001645	3.39379	ENSG0000	ELOVL6/D
GO:00605	6	6/545	28/20610	7.63E-05	0.001735	8.103539	ENSG0000	TP63/ID4/
GO:00619!	4	4/545	10/20610	8.95E-05	0.001993	15.12661	ENSG0000	SGO2/BUE
GO:00063	11	11/545	120/2061C	0.000345	0.006977	3.466514	ENSG0000	HIST1H4A
GO:00513(	14	14/545	182/2061C	0.000359	0.007218	2.908963	ENSG0000	TP63/BLM
GO:00900	5	5/545	25/20610	0.000435	0.008517	7.563303	ENSG0000	HSPA1B/N
GO:00420	7	7/545	54/20610	0.000525	0.010047	4.902141	ENSG0000	HSP90AA1
GO:00607	5	5/545	26/20610	0.000527	0.010047	7.272406	ENSG0000	TP63/ID4/
GO:00709!	24	24/545	436/2061C	0.00063	0.011805	2.081643	ENSG0000	DKK1/TP6
GO:00016!	14	14/545	194/2061C	0.00068	0.012482	2.729027	ENSG0000	ELOVL6/D
GO:00328!	16	16/545	243/2061C	0.000789	0.01424	2.489976	ENSG0000	PLK1/AUR
GO:00015!	5	5/545	29/20610	0.000891	0.015946	6.520089	ENSG0000	AURKA/PD
GO:00609(	12	12/545	162/2061C	0.001285	0.021837	2.801223	ENSG0000	HIST1H4A
GO:00350	8	8/545	82/20610	0.001456	0.024081	3.689416	ENSG0000	TP63/ZFP3
GO:00326	9	9/545	104/2061C	0.001757	0.028412	3.272583	ENSG0000	TLR1/HSP/
GO:00071	6	6/545	50/20610	0.00197	0.030389	4.537982	ENSG0000	TRIP13/KII
GO:00064!	6	6/545	52/20610	0.002414	0.035369	4.363444	ENSG0000	HSPE1/HS
GO:00514	6	6/545	52/20610	0.002414	0.035369	4.363444	ENSG0000	CDC20/PD
GO:00011(	20	20/545	374/2061C	0.002426	0.035371	2.022273	ENSG0000	PCNA/DKI
GO:00610	7	7/545	71/20610	0.002683	0.038037	3.728389	ENSG0000	HSPE1/HS
GO:00716!	12	12/545	177/2061C	0.002707	0.038172	2.563831	ENSG0000	AURKA/PD
GO:00099!	6	6/545	55/20610	0.003217	0.044003	4.125438	ENSG0000	AURKA/PD
GO:00019	8	8/545	93/20610	0.003235	0.044003	3.253033	ENSG0000	DKK1/TP6

GO:00060	3	3/545	12/20610	0.003385	0.04545	9.454128	ENSG0000	ADH1C/A
GO:00427	5	5/545	39/20610	0.003479	0.046164	4.848271	ENSG0000	PCNA/RFC
GO:00310	13	13/545	207/2061	0.003566	0.04718	2.37495	ENSG0000	PCNA/AU
GO:00607	7	7/545	75/20610	0.00366	0.048273	3.529541	ENSG0000	HIF1A/HS
GO:00224	8	8/545	96/20610	0.003934	0.050516	3.151376	ENSG0000	DKK1/TP6
GO:00224	8	8/545	96/20610	0.003934	0.050516	3.151376	ENSG0000	DKK1/TP6
GO:00310	4	4/545	25/20610	0.003934	0.050516	6.050642	ENSG0000	TP63/FGF
GO:00309	21	21/545	423/2061	0.004551	0.055654	1.877416	ENSG0000	DKK1/TBX
GO:00456	6	6/545	59/20610	0.004576	0.055654	3.845747	ENSG0000	HIF1A/HS
GO:00426	9	9/545	123/2061	0.00543	0.062715	2.767062	ENSG0000	DKK1/TP6
GO:00067	4	4/545	28/20610	0.005982	0.068101	5.402359	ENSG0000	TYMS/DH
GO:00107	4	4/545	28/20610	0.005982	0.068101	5.402359	ENSG0000	ARRB2/SE
GO:00512	3	3/545	16/20610	0.007966	0.085034	7.090596	ENSG0000	AURKA/KI
GO:00718	13	13/545	230/2061	0.008477	0.088965	2.137455	ENSG0000	SLC9A2/SL
GO:00717	10	10/545	158/2061	0.009452	0.095863	2.39345	ENSG0000	NR4A1/GC
GO:00973	3	3/545	17/20610	0.009485	0.095863	6.673502	ENSG0000	BLM/TIME
GO:00065	20	20/545	426/2061	0.009966	0.10003	1.775423	ENSG0000	ODC1/DD
GO:00359	13	13/545	236/2061	0.01038	0.103685	2.083113	ENSG0000	SDF2L1/H
GO:01400	11	11/545	185/2061	0.010411	0.103685	2.248549	ENSG0000	CPLX2/UN
GO:00018	7	7/545	91/20610	0.010426	0.103685	2.908963	ENSG0000	E2F8/HIF1
GO:00611	12	12/545	211/2061	0.010678	0.105717	2.150702	ENSG0000	TP63/ARE
GO:00481	5	5/545	51/20610	0.010965	0.107824	3.707501	ENSG0000	CCNB1/CD
GO:00516	5	5/545	51/20610	0.010965	0.107824	3.707501	ENSG0000	PLK1/MAD
GO:00517	3	3/545	18/20610	0.011162	0.108376	6.302752	ENSG0000	BLM/E2F8
GO:00901	5	5/545	52/20610	0.011875	0.114197	3.636203	ENSG0000	GABRA5/
GO:00484	11	11/545	190/2061	0.012531	0.119455	2.189377	ENSG0000	AURKA/PD
GO:00421	4	4/545	35/20610	0.013234	0.123728	4.321887	ENSG0000	AMBP/HM
GO:00065	12	12/545	218/2061	0.013554	0.126141	2.081643	ENSG0000	PLA2G4A/
GO:00102	4	4/545	36/20610	0.014585	0.131837	4.201835	ENSG0000	TP63/DDC
GO:00426	3	3/545	20/20610	0.014998	0.133904	5.672477	ENSG0000	FST/HPSE/
GO:20010	7	7/545	98/20610	0.015245	0.135836	2.70118	ENSG0000	PCNA/TIM
GO:00105	4	4/545	37/20610	0.016019	0.14158	4.088272	ENSG0000	BRCA1/NC
GO:19037	12	12/545	224/2061	0.016459	0.14459	2.025885	ENSG0000	XBP1/HIF1
GO:00458	7	7/545	100/20610	0.016872	0.14691	2.647156	ENSG0000	BRCA1/DG
GO:00017	12	12/545	225/2061	0.016986	0.14691	2.016881	ENSG0000	TP63/ARE
GO:00611	5	5/545	57/20610	0.017194	0.14691	3.317238	ENSG0000	HIF1A/TIA
GO:00302	12	12/545	227/2061	0.018077	0.153551	1.999111	ENSG0000	DKK1/TP6
GO:00031	6	6/545	79/20610	0.0182	0.15429	2.87214	ENSG0000	TBX3/HIF1
GO:00067	9	9/545	150/2061	0.018452	0.155525	2.268991	ENSG0000	KYNU/DH
GO:00716	4	4/545	39/20610	0.019146	0.16014	3.878617	ENSG0000	HIF1A/SER
GO:00093	21	21/545	488/2061	0.020459	0.168856	1.62735	ENSG0000	PCNA/BL
GO:00009	10	10/545	179/2061	0.021017	0.172485	2.112654	ENSG0000	PLK1/AUR
GO:00344	7	7/545	105/2061	0.02147	0.175869	2.521101	ENSG0000	ACOX2/PD
GO:00486	5	5/545	61/20610	0.022438	0.181414	3.099714	ENSG0000	DKK1/TP6
GO:00466	16	16/545	347/2061	0.022961	0.184622	1.743701	ENSG0000	PCNA/ARE
GO:00215	6	6/545	84/20610	0.023868	0.190163	2.70118	ENSG0000	SOX2/AR
GO:00303	11	11/545	210/2061	0.024402	0.192771	1.980865	ENSG0000	G6PD/EN
GO:00328	5	5/545	63/20610	0.025409	0.198096	3.001311	ENSG0000	ANGPT1/E
GO:00451	4	4/545	43/20610	0.026466	0.204712	3.517815	ENSG0000	PDK4/PPA
GO:00718	2	2/545	10/20610	0.027289	0.204712	7.563303	ENSG0000	HMGA2/F
GO:00380	6	6/545	87/20610	0.027781	0.206634	2.608035	ENSG0000	HSPA1B/
GO:00486	19	19/545	445/2061	0.028471	0.211044	1.614638	ENSG0000	TP63/ARR
GO:00098	5	5/545	65/20610	0.02862	0.21143	2.908963	ENSG0000	TBX3/SEM
GO:00442	20	20/545	476/2061	0.029012	0.213965	1.588929	ENSG0000	ACOX2/KY
GO:00421	13	13/545	272/2061	0.029594	0.21752	1.807407	ENSG0000	ODC1/KY
GO:00068	8	8/545	138/2061	0.030468	0.219901	2.192262	ENSG0000	ALDH3A1/
GO:19019	4	4/545	45/20610	0.030673	0.219901	3.361468	ENSG0000	CCNB1/W
GO:00216	2	2/545	11/20610	0.032777	0.226792	6.87573	ENSG0000	SEMA3F/H

GO:00721:	2	2/545	11/20610	0.032777	0.226792	6.87573	ENSG0000	SERPINB7/
GO:00459:	3	3/545	27/20610	0.0336	0.229215	4.201835	ENSG0000	DKK1/WN'
GO:00708:	4	4/545	47/20610	0.035252	0.237886	3.218427	ENSG0000	NCL/MCM
GO:00300:	8	8/545	143/2061(	0.036487	0.245459	2.115609	ENSG0000	PLK1/HSP:
GO:00466(	7	7/545	118/2061(	0.037319	0.246022	2.243353	ENSG0000	TP63/ARR
GO:00067:	17	17/545	399/2061(	0.03744	0.246022	1.61123	ENSG0000	PHGDH/El
GO:00466:	5	5/545	70/20610	0.037733	0.246022	2.70118	ENSG0000	CCNB1/PA
GO:00024(	2	2/545	12/20610	0.038656	0.246022	6.302752	ENSG0000	CD274/FO
GO:00607:	2	2/545	12/20610	0.038656	0.246022	6.302752	ENSG0000	TP63/FGFI
GO:00725:	2	2/545	12/20610	0.038656	0.246022	6.302752	ENSG0000	GCLM/MY
GO:19058:	2	2/545	12/20610	0.038656	0.246022	6.302752	ENSG0000	AURKA/PD
GO:00215:	13	13/545	284/2061(	0.039724	0.250043	1.731038	ENSG0000	H2AFX/WI
GO:00335:	3	3/545	29/20610	0.040389	0.251554	3.912053	ENSG0000	AREG/BRC
GO:00364:	6	6/545	96/20610	0.041978	0.257049	2.363532	ENSG0000	LANCL1/N
GO:00107:	4	4/545	50/20610	0.042824	0.259988	3.025321	ENSG0000	ARRB2/SEI
GO:00302:	6	6/545	97/20610	0.043791	0.259988	2.339166	ENSG0000	DKK1/ARE
GO:20010:	6	6/545	97/20610	0.043791	0.259988	2.339166	ENSG0000	DDIAS/HM
GO:00336:	3	3/545	30/20610	0.044023	0.259988	3.781651	ENSG0000	FGFR2/HP:
GO:00516:	3	3/545	30/20610	0.044023	0.259988	3.781651	ENSG0000	AURKA/M.
GO:00140:	2	2/545	13/20610	0.0449	0.259988	5.817925	ENSG0000	NOLC1/KE
GO:00337:	2	2/545	13/20610	0.0449	0.259988	5.817925	ENSG0000	RRS1/LTV1
GO:19000:	5	5/545	74/20610	0.046168	0.265335	2.55517	ENSG0000	ENPP1/CC
GO:00341(	8	8/545	151/2061(	0.047698	0.270763	2.003524	ENSG0000	G6PD/HIF:
GO:00519:	3	3/545	31/20610	0.047815	0.270763	3.659663	ENSG0000	CCNB1/CD
GO:00027(	4	4/545	52/20610	0.04834	0.272677	2.908963	ENSG0000	ANGPT1/II
GO:00098:	19	19/545	474/2061(	0.048551	0.273161	1.515852	ENSG0000	TOB1/PLK:
GO:00488:	10	10/545	207/2061(	0.049144	0.276141	1.826885	ENSG0000	GABRA5/N
GO:00456:	6	6/545	100/2061(	0.049522	0.276944	2.268991	ENSG0000	TP63/CTSI
GO:20002:	8	8/545	153/2061(	0.050816	0.276944	1.977334	ENSG0000	AURKA/CD
GO:00094:	14	14/545	325/2061(	0.051367	0.276944	1.629019	ENSG0000	ALDH3A1/
GO:00104:	2	2/545	14/20610	0.051485	0.276944	5.402359	ENSG0000	NEK2/SGC
GO:19017:	2	2/545	14/20610	0.051485	0.276944	5.402359	ENSG0000	SERPINB7/
GO:00217:	3	3/545	32/20610	0.051761	0.276944	3.545298	ENSG0000	ARX/SALL:
GO:00602:	3	3/545	32/20610	0.051761	0.276944	3.545298	ENSG0000	NPY2R/NF
GO:00329(	4	4/545	54/20610	0.05423	0.286297	2.801223	ENSG0000	ARRB2/SEI
GO:00506:	10	10/545	211/2061(	0.054579	0.287792	1.792252	ENSG0000	TP63/ARE(
GO:00023:	7	7/545	129/2061(	0.05558	0.290332	2.052059	ENSG0000	EXO1/XBP
GO:00347:	8	8/545	156/2061(	0.055734	0.290332	1.939308	ENSG0000	FDXR/DG/
GO:00357:	3	3/545	33/20610	0.055859	0.290332	3.437865	ENSG0000	HIF1A/GPI
GO:20010:	3	3/545	33/20610	0.055859	0.290332	3.437865	ENSG0000	HMGA2/N
GO:00075:	11	11/545	241/2061(	0.056384	0.292691	1.726065	ENSG0000	ALDH3A1/
GO:00486:	6	6/545	104/2061(	0.057849	0.292691	2.181722	ENSG0000	ARRB2/CC
GO:00098:	2	2/545	15/20610	0.058391	0.292691	5.042202	ENSG0000	UGT1A6/U
GO:00459:	2	2/545	15/20610	0.058391	0.292691	5.042202	ENSG0000	LBP/CFHR.
GO:20002:	2	2/545	15/20610	0.058391	0.292691	5.042202	ENSG0000	NPY2R/M(
GO:19018(	6	6/545	105/2061(	0.060054	0.29821	2.160944	ENSG0000	ARRB2/CC
GO:00219:	3	3/545	34/20610	0.060106	0.29821	3.336751	ENSG0000	ARX/SALL:
GO:00216:	5	5/545	80/20610	0.060762	0.299757	2.363532	ENSG0000	UNC13A/(
GO:00459:	12	12/545	274/2061(	0.061362	0.302114	1.656198	ENSG0000	G6PD/ENI
GO:00620:	8	8/545	160/2061(	0.062748	0.308164	1.890826	ENSG0000	CCNB1/CD
GO:00426:	3	3/545	35/20610	0.064501	0.314363	3.241415	ENSG0000	FST/HPSE/
GO:00700:	3	3/545	35/20610	0.064501	0.314363	3.241415	ENSG0000	MAP1A/S(
GO:00351(	8	8/545	161/2061(	0.064584	0.314363	1.879081	ENSG0000	DKK1/TP6:
GO:00514:	2	2/545	16/20610	0.065594	0.314363	4.727064	ENSG0000	TTK/TRIP1
GO:20002:	4	4/545	58/20610	0.067116	0.319551	2.608035	ENSG0000	TTK/TRIP1
GO:00060:	5	5/545	83/20610	0.068932	0.325862	2.278103	ENSG0000	ALDH3A1/
GO:00316:	2	2/545	17/20610	0.073075	0.336193	4.449002	ENSG0000	NCMAP/H
GO:00509:	3	3/545	37/20610	0.073717	0.337012	3.066204	ENSG0000	ENPP1/US

GO:00486(	9	9/545	195/2061(	0.075109	0.341946	1.745378	ENSG0000	NOX1/NQ
GO:00030(	15	15/545	377/2061(	0.077195	0.350306	1.504636	ENSG0000	DKK1/TP6:
GO:00224:	15	15/545	377/2061(	0.077195	0.350306	1.504636	ENSG0000	ARRB2/PLI
GO:00605(	11	11/545	255/2061(	0.077266	0.350306	1.631301	ENSG0000	AREG/UN(
GO:00725!	13	13/545	316/2061(	0.078123	0.351996	1.555743	ENSG0000	FOXM1/G(
GO:19021:	10	10/545	226/2061(	0.078415	0.352585	1.673297	ENSG0000	PLK1/BRC/
GO:00600-	3	3/545	38/20610	0.078532	0.352617	2.985514	ENSG0000	CCNB1/CD
GO:00017(	7	7/545	140/2061(	0.078584	0.352617	1.890826	ENSG0000	DKK1/HM(
GO:00486!	9	9/545	197/2061(	0.078892	0.353275	1.727658	ENSG0000	NOX1/NQ
GO:00712:	14	14/545	348/2061(	0.079949	0.356041	1.521354	ENSG0000	PCNA/BLN
GO:01040(	14	14/545	348/2061(	0.079949	0.356041	1.521354	ENSG0000	PCNA/BLN
GO:00073{	18	18/545	474/2061(	0.080527	0.356041	1.43607	ENSG0000	DKK1/TP6:
GO:00485:	2	2/545	18/20610	0.080815	0.356041	4.201835	ENSG0000	SEMA3F/H
GO:00075(	8	8/545	170/2061(	0.082597	0.36243	1.779601	ENSG0000	TP63/FOXI
GO:00215-	9	9/545	199/2061(	0.082789	0.362909	1.710295	ENSG0000	H2AFX/WI
GO:00016(	3	3/545	39/20610	0.083481	0.363725	2.908963	ENSG0000	GABRA5/N
GO:00070!	3	3/545	39/20610	0.083481	0.363725	2.908963	ENSG0000	BRCA1/WI
GO:00326:	3	3/545	39/20610	0.083481	0.363725	2.908963	ENSG0000	IL33/CD83
GO:00316(	12	12/545	289/2061(	0.083558	0.363725	1.570236	ENSG0000	AIFM1/FA!
GO:00485{	11	11/545	259/2061(	0.084022	0.36465	1.606107	ENSG0000	UNC13A/
GO:00974{	12	12/545	290/2061(	0.085196	0.368467	1.564821	ENSG0000	UNC5D/ET
GO:00481-	5	5/545	89/20610	0.086996	0.373028	2.124523	ENSG0000	CCNB1/CD
GO:00316-	13	13/545	322/2061(	0.087315	0.373028	1.526754	ENSG0000	PLK1/AUR
GO:00550:	6	6/545	116/2061(	0.087557	0.373028	1.956027	ENSG0000	G6PD/CCN
GO:00320!	3	3/545	40/20610	0.088559	0.373028	2.836239	ENSG0000	G6PD/CPS
GO:20006-	3	3/545	40/20610	0.088559	0.373028	2.836239	ENSG0000	TBX3/HM(
GO:00160{	2	2/545	19/20610	0.088793	0.373028	3.980686	ENSG0000	UNC13A/S
GO:00097!	5	5/545	90/20610	0.090225	0.376598	2.100917	ENSG0000	AURKA/TB
GO:00508{	5	5/545	91/20610	0.093516	0.385479	2.07783	ENSG0000	TBX3/NOX
GO:19001{	3	3/545	41/20610	0.093765	0.385479	2.767062	ENSG0000	ANGPT1/D
GO:19018(	8	8/545	175/2061(	0.093765	0.385479	1.728755	ENSG0000	DKK1/ARR
GO:00070:	12	12/545	296/2061(	0.095442	0.391969	1.533102	ENSG0000	KIF11/CEN
GO:00026-	2	2/545	20/20610	0.096992	0.391969	3.781651	ENSG0000	CD274/FO
GO:00363-	2	2/545	20/20610	0.096992	0.391969	3.781651	ENSG0000	TP63/WNT
GO:00171-	3	3/545	42/20610	0.099093	0.397646	2.70118	ENSG0000	NAP1L2/Z
GO:00488!	4	4/545	67/20610	0.10127	0.404896	2.257702	ENSG0000	G6PD/ARX
GO:00714!	14	14/545	362/2061(	0.101564	0.405327	1.462517	ENSG0000	AIFM1/FA!
GO:00353{	6	6/545	121/2061(	0.101998	0.406317	1.875199	ENSG0000	ELOVL6/K
GO:19058!	3	3/545	43/20610	0.10454	0.408634	2.638361	ENSG0000	XBP1/USP:
GO:00466:	6	6/545	122/2061(	0.105028	0.408634	1.859829	ENSG0000	G6PD/CCN
GO:00422(	2	2/545	21/20610	0.105396	0.408634	3.601573	ENSG0000	LBP/CFHR
GO:00550!	2	2/545	21/20610	0.105396	0.408634	3.601573	ENSG0000	FAS/NOX1
GO:00607:	2	2/545	21/20610	0.105396	0.408634	3.601573	ENSG0000	FGFR2/BM
GO:20003{	2	2/545	21/20610	0.105396	0.408634	3.601573	ENSG0000	DKK1/TP6:
GO:00450:	4	4/545	68/20610	0.105481	0.408634	2.224501	ENSG0000	BRCA1/CD
GO:00604:	6	6/545	123/2061(	0.108104	0.417314	1.844708	ENSG0000	G6PD/CCN
GO:00606{	4	4/545	69/20610	0.10977	0.420933	2.192262	ENSG0000	FGFR2/WN
GO:00069(	3	3/545	44/20610	0.110102	0.420933	2.578399	ENSG0000	CPLX2/UN
GO:00326:	2	2/545	22/20610	0.113987	0.427916	3.437865	ENSG0000	IL33/FOXP
GO:00361:	2	2/545	22/20610	0.113987	0.427916	3.437865	ENSG0000	CCNA2/M
GO:00443-	2	2/545	22/20610	0.113987	0.427916	3.437865	ENSG0000	NR4A1/NF
GO:00485:	2	2/545	22/20610	0.113987	0.427916	3.437865	ENSG0000	NPY2R/M(
GO:00613:	2	2/545	22/20610	0.113987	0.427916	3.437865	ENSG0000	WNT5A/SI
GO:00486(	6	6/545	125/2061(	0.114394	0.428707	1.815193	ENSG0000	NOX1/NQ
GO:00140-	3	3/545	45/20610	0.115775	0.429826	2.521101	ENSG0000	SNAP25/S
GO:01101:	3	3/545	45/20610	0.115775	0.429826	2.521101	ENSG0000	BCL2/WNT
GO:00230(	18	18/545	500/2061(	0.116431	0.431892	1.361394	ENSG0000	CPLX2/UN
GO:00326:	4	4/545	71/20610	0.118577	0.437623	2.130508	ENSG0000	CD274/CD

GO:000225	3	3/545	46/20610	0.121556	0.443645	2.466294	ENSG0000	HIST1H2BI
GO:004259	3	3/545	46/20610	0.121556	0.443645	2.466294	ENSG0000	GABRA5/N
GO:004568	3	3/545	46/20610	0.121556	0.443645	2.466294	ENSG0000	FST/HPSE/
GO:003090	5	5/545	99/20610	0.121973	0.443645	1.909925	ENSG0000	DKK1/G6P
GO:200010	2	2/545	23/20610	0.122749	0.443645	3.288393	ENSG0000	NR4A3/HC
GO:005129	4	4/545	72/20610	0.123092	0.444149	2.100917	ENSG0000	BRCA1/WI
GO:200024	4	4/545	72/20610	0.123092	0.444149	2.100917	ENSG0000	AURKA/PD
GO:000175	4	4/545	73/20610	0.127679	0.455059	2.072138	ENSG0000	DKK1/XRC
GO:005109	15	15/545	410/20610	0.129522	0.459749	1.383531	ENSG0000	DKK1/ARR
GO:000320	6	6/545	130/20610	0.130901	0.460998	1.745378	ENSG0000	TBX3/HIF1
GO:006074	2	2/545	24/20610	0.131669	0.460998	3.151376	ENSG0000	AREG/HIF1
GO:006137	2	2/545	24/20610	0.131669	0.460998	3.151376	ENSG0000	AREG/HIF1
GO:009950	2	2/545	24/20610	0.131669	0.460998	3.151376	ENSG0000	CPLX2/SN
GO:000164	10	10/545	252/20610	0.132917	0.461016	1.500655	ENSG0000	TP63/ARE
GO:006124	3	3/545	48/20610	0.133425	0.461016	2.363532	ENSG0000	WNT5A/SH
GO:000205	2	2/545	25/20610	0.14073	0.474462	3.025321	ENSG0000	FGFR2/WN
GO:000658	2	2/545	25/20610	0.14073	0.474462	3.025321	ENSG0000	BCL2/WNT
GO:190533	2	2/545	25/20610	0.14073	0.474462	3.025321	ENSG0000	WNT5A/BI
GO:003052	12	12/545	319/20610	0.141306	0.475723	1.422565	ENSG0000	TP63/ARR
GO:004873	8	8/545	194/20610	0.143549	0.481358	1.559444	ENSG0000	DKK1/TP63
GO:009917	3	3/545	50/20610	0.145675	0.485881	2.268991	ENSG0000	DKK1/WNT
GO:006041	4	4/545	77/20610	0.146717	0.486383	1.964494	ENSG0000	TBX3/FGF
GO:190303	4	4/545	77/20610	0.146717	0.486383	1.964494	ENSG0000	XBP1/HPS
GO:000269	14	14/545	387/20610	0.14819	0.490563	1.368039	ENSG0000	PAK3/XBP
GO:005144	2	2/545	26/20610	0.14992	0.490669	2.908963	ENSG0000	CDC25A/V
GO:009715	2	2/545	26/20610	0.14992	0.490669	2.908963	ENSG0000	FANCD2/S
GO:200017	2	2/545	26/20610	0.14992	0.490669	2.908963	ENSG0000	BTG2/WNT
GO:001635	10	10/545	259/20610	0.150368	0.491765	1.460097	ENSG0000	PAK3/CDC
GO:003263	8	8/545	197/20610	0.15242	0.495137	1.535696	ENSG0000	ARRB2/XB
GO:190520	3	3/545	52/20610	0.158276	0.507431	2.181722	ENSG0000	ARRB2/PA
GO:000959	2	2/545	27/20610	0.159226	0.507431	2.801223	ENSG0000	TLR1/LBP
GO:003019	2	2/545	27/20610	0.159226	0.507431	2.801223	ENSG0000	HPSE/APC
GO:004593	2	2/545	27/20610	0.159226	0.507431	2.801223	ENSG0000	PRKG1/CA
GO:006060	2	2/545	27/20610	0.159226	0.507431	2.801223	ENSG0000	FGFR2/BM
GO:190004	2	2/545	27/20610	0.159226	0.507431	2.801223	ENSG0000	HPSE/APC
GO:003355	4	4/545	80/20610	0.161671	0.512977	1.890826	ENSG0000	GABRA5/N
GO:000293	3	3/545	53/20610	0.164699	0.518813	2.140557	ENSG0000	AIFM1/FAI
GO:004516	11	11/545	297/20610	0.165586	0.520856	1.400612	ENSG0000	DKK1/TBX
GO:003260	5	5/545	110/20610	0.166757	0.523785	1.718932	ENSG0000	HIF1A/IL33
GO:004863	8	8/545	202/20610	0.167771	0.524027	1.497684	ENSG0000	UNC13A/C
GO:000264	2	2/545	28/20610	0.168634	0.524027	2.70118	ENSG0000	CD274/FO
GO:002184	2	2/545	28/20610	0.168634	0.524027	2.70118	ENSG0000	ARX/FGFR
GO:005082	2	2/545	28/20610	0.168634	0.524027	2.70118	ENSG0000	HPSE/APC
GO:190288	2	2/545	28/20610	0.168634	0.524027	2.70118	ENSG0000	ACOX2/NC
GO:011011	4	4/545	82/20610	0.171936	0.530509	1.844708	ENSG0000	DKK1/XBP
GO:000150	14	14/545	399/20610	0.174085	0.53644	1.326895	ENSG0000	CPLX2/DD
GO:004259	8	8/545	204/20610	0.174103	0.53644	1.483001	ENSG0000	FAS/CPS1/
GO:002240	12	12/545	334/20610	0.176605	0.542731	1.358677	ENSG0000	PAK3/XBP
GO:001485	4	4/545	83/20610	0.177151	0.542731	1.822483	ENSG0000	CCNB1/CD
GO:004591	4	4/545	83/20610	0.177151	0.542731	1.822483	ENSG0000	HIF1A/DG
GO:002160	2	2/545	29/20610	0.178134	0.542731	2.608035	ENSG0000	SEMA3F/H
GO:004852	2	2/545	29/20610	0.178134	0.542731	2.608035	ENSG0000	NPY2R/NR
GO:190520	2	2/545	29/20610	0.178134	0.542731	2.608035	ENSG0000	DKK1/G6P
GO:005067	14	14/545	401/20610	0.178609	0.54368	1.320277	ENSG0000	TP63/ARE
GO:004244	5	5/545	113/20610	0.179968	0.546413	1.673297	ENSG0000	FDXR/DIO
GO:190401	9	9/545	238/20610	0.180763	0.548443	1.430036	ENSG0000	ASB4/BRC
GO:000759	13	13/545	369/20610	0.18147	0.550206	1.332289	ENSG0000	ARRB2/HIS
GO:000320	7	7/545	175/20610	0.182363	0.551278	1.512661	ENSG0000	TBX3/HIF1

GO:000606	14	14/545	403/20610	0.183192	0.55274	1.313725	ENSG0000	ELOVL6/FI
GO:005170	10	10/545	273/20610	0.188506	0.561786	1.38522	ENSG0000	ANPEP/TY
GO:004275	5	5/545	115/20610	0.188986	0.562067	1.644196	ENSG0000	TIMELESS/
GO:005196	5	5/545	115/20610	0.188986	0.562067	1.644196	ENSG0000	DKK1/NEC
GO:007177	7	7/545	177/20610	0.189538	0.562941	1.495568	ENSG0000	DKK1/TOB
GO:000327	5	5/545	116/20610	0.193555	0.572535	1.630022	ENSG0000	TBX3/SALL
GO:002260	5	5/545	116/20610	0.193555	0.572535	1.630022	ENSG0000	SERPINA3/
GO:000716	6	6/545	147/20610	0.194588	0.573261	1.543531	ENSG0000	DKK1/TP63
GO:005086	14	14/545	409/20610	0.197275	0.574787	1.294453	ENSG0000	PAK3/XBP
GO:009034	2	2/545	31/20610	0.197362	0.574787	2.439775	ENSG0000	FOXM1/HI
GO:000268	3	3/545	58/20610	0.197871	0.574787	1.956027	ENSG0000	IL33/PTGE
GO:006004	3	3/545	58/20610	0.197871	0.574787	1.956027	ENSG0000	CCNB1/CD
GO:000754	10	10/545	277/20610	0.200136	0.579825	1.365217	ENSG0000	TP63/ARR
GO:000599	11	11/545	311/20610	0.202991	0.587314	1.337562	ENSG0000	G6PD/OAS
GO:190040	3	3/545	59/20610	0.204691	0.590278	1.922874	ENSG0000	HIF1A/NR4
GO:007200	6	6/545	150/20610	0.206884	0.59205	1.512661	ENSG0000	ANGPT1/E
GO:003526	8	8/545	215/20610	0.210735	0.599783	1.407126	ENSG0000	G6PD/CCN
GO:005088	3	3/545	60/20610	0.211564	0.600188	1.890826	ENSG0000	MAP1A/KC
GO:000189	9	9/545	248/20610	0.211934	0.600458	1.372373	ENSG0000	PDK4/SER
GO:002176	4	4/545	90/20610	0.215038	0.605255	1.680734	ENSG0000	ID4/BTG2/
GO:001046	2	2/545	33/20610	0.216824	0.605255	2.29191	ENSG0000	FGFR2/WN
GO:200102	2	2/545	33/20610	0.216824	0.605255	2.29191	ENSG0000	NR4A3/HC
GO:190288	3	3/545	61/20610	0.218487	0.606044	1.859829	ENSG0000	HIF1A/NR4
GO:000697	4	4/545	91/20610	0.220625	0.606174	1.662264	ENSG0000	MAP7/SLC
GO:200017	4	4/545	91/20610	0.220625	0.606174	1.662264	ENSG0000	SHCBP1/H
GO:000270	8	8/545	218/20610	0.221213	0.606174	1.387762	ENSG0000	XBP1/ATA
GO:000250	7	7/545	186/20610	0.223162	0.606174	1.423202	ENSG0000	KIF11/CEN
GO:001644	3	3/545	62/20610	0.225455	0.606174	1.829831	ENSG0000	EXO1/ATA
GO:005135	3	3/545	62/20610	0.225455	0.606174	1.829831	ENSG0000	DHFR/HIF1
GO:000740	3	3/545	63/20610	0.232466	0.606174	1.800786	ENSG0000	ID4/HIF1A
GO:000314	1	1/545	10/20610	0.235133	0.606174	3.781651	ENSG0000	FGFR2
GO:003091	1	1/545	10/20610	0.235133	0.606174	3.781651	ENSG0000	GBX2
GO:003369	1	1/545	10/20610	0.235133	0.606174	3.781651	ENSG0000	HPSE
GO:004875	1	1/545	10/20610	0.235133	0.606174	3.781651	ENSG0000	FGFR2
GO:005123	1	1/545	10/20610	0.235133	0.606174	3.781651	ENSG0000	KIF4A
GO:005125	1	1/545	10/20610	0.235133	0.606174	3.781651	ENSG0000	KIF4A
GO:005140	1	1/545	10/20610	0.235133	0.606174	3.781651	ENSG0000	GCLM
GO:007031	1	1/545	10/20610	0.235133	0.606174	3.781651	ENSG0000	PHGDH
GO:007203	1	1/545	10/20610	0.235133	0.606174	3.781651	ENSG0000	SALL1
GO:007220	1	1/545	10/20610	0.235133	0.606174	3.781651	ENSG0000	BMP7
GO:190245	1	1/545	10/20610	0.235133	0.606174	3.781651	ENSG0000	BMP7
GO:200025	1	1/545	10/20610	0.235133	0.606174	3.781651	ENSG0000	NR4A3
GO:007016	2	2/545	35/20610	0.236444	0.606174	2.160944	ENSG0000	ENPP1/HIF
GO:000256	3	3/545	64/20610	0.239516	0.609753	1.772649	ENSG0000	EXO1/ATA
GO:004614	3	3/545	64/20610	0.239516	0.609753	1.772649	ENSG0000	HMBS/NA
GO:006075	8	8/545	224/20610	0.242725	0.612466	1.35059	ENSG0000	ANGPT1/H
GO:004688	10	10/545	292/20610	0.246291	0.612466	1.295086	ENSG0000	TARDBP/S
GO:004336	2	2/545	36/20610	0.246291	0.612466	2.100917	ENSG0000	BCL2/FOX
GO:009700	2	2/545	36/20610	0.246291	0.612466	2.100917	ENSG0000	MLXIPL/NI
GO:000226	3	3/545	65/20610	0.246601	0.612466	1.745378	ENSG0000	HIF1A/BCL
GO:004684	4	4/545	96/20610	0.249097	0.612466	1.575688	ENSG0000	PDK4/PPA
GO:000761	6	6/545	160/20610	0.249697	0.612466	1.418119	ENSG0000	DKK1/GAB
GO:004513	8	8/545	226/20610	0.250047	0.612466	1.338638	ENSG0000	ARRB2/KIF
GO:005080	9	9/545	261/20610	0.255348	0.612466	1.304018	ENSG0000	DKK1/PAK
GO:002153	1	1/545	11/20610	0.255368	0.612466	3.437865	ENSG0000	GBX2
GO:002154	1	1/545	11/20610	0.255368	0.612466	3.437865	ENSG0000	BCL2
GO:002169	1	1/545	11/20610	0.255368	0.612466	3.437865	ENSG0000	FAIM2
GO:005191	1	1/545	11/20610	0.255368	0.612466	3.437865	ENSG0000	APOH

GO:007025	1	1/545	11/20610	0.255368	0.612466	3.437865	ENSG0000	AGR2
GO:007027	1	1/545	11/20610	0.255368	0.612466	3.437865	ENSG0000	CREB3L1
GO:190247	1	1/545	11/20610	0.255368	0.612466	3.437865	ENSG0000	WNT5A
GO:009750	2	2/545	37/20610	0.256153	0.612466	2.044136	ENSG0000	SDF2L1/NU
GO:000975	9	9/545	262/20610	0.258804	0.616514	1.299041	ENSG0000	TP63/ARRI
GO:004577	4	4/545	98/20610	0.260701	0.616514	1.543531	ENSG0000	TP63/WNT
GO:006002	4	4/545	98/20610	0.260701	0.616514	1.543531	ENSG0000	TBX3/ANP
GO:000076	3	3/545	67/20610	0.260864	0.616514	1.693277	ENSG0000	SH3PXD2A
GO:004666	6	6/545	163/20610	0.263009	0.616514	1.392019	ENSG0000	TBX3/KIF1
GO:190007	2	2/545	38/20610	0.266021	0.616514	1.990343	ENSG0000	ENPP1/EN
GO:190103	2	2/545	38/20610	0.266021	0.616514	1.990343	ENSG0000	TP63/BCL2
GO:001062	1	1/545	12/20610	0.27507	0.616514	3.151376	ENSG0000	BCL2
GO:001618	1	1/545	12/20610	0.27507	0.616514	3.151376	ENSG0000	UNC13A
GO:004465	1	1/545	12/20610	0.27507	0.616514	3.151376	ENSG0000	NECTIN1
GO:004695	1	1/545	12/20610	0.27507	0.616514	3.151376	ENSG0000	HMGCS2
GO:006066	1	1/545	12/20610	0.27507	0.616514	3.151376	ENSG0000	FGFR2
GO:006067	1	1/545	12/20610	0.27507	0.616514	3.151376	ENSG0000	FGFR2
GO:006073	1	1/545	12/20610	0.27507	0.616514	3.151376	ENSG0000	FGFR2
GO:006078	1	1/545	12/20610	0.27507	0.616514	3.151376	ENSG0000	TBX3
GO:007169	1	1/545	12/20610	0.27507	0.616514	3.151376	ENSG0000	TBX3
GO:009000	1	1/545	12/20610	0.27507	0.616514	3.151376	ENSG0000	WNT5A
GO:000694	3	3/545	69/20610	0.27523	0.616514	1.644196	ENSG0000	SH3PXD2A
GO:004863	12	12/545	370/20610	0.275854	0.616514	1.226482	ENSG0000	UNC13A/C
GO:000692	2	2/545	39/20610	0.275888	0.616514	1.939308	ENSG0000	AIFM1/TO
GO:003410	4	4/545	101/20610	0.278294	0.619597	1.497684	ENSG0000	PDK4/PPA
GO:190353	15	15/545	476/20610	0.27992	0.622583	1.191697	ENSG0000	UNC13A/X
GO:003164	3	3/545	70/20610	0.282443	0.623457	1.620708	ENSG0000	NCMAP/C
GO:002198	5	5/545	135/20610	0.286401	0.623457	1.400612	ENSG0000	H2AFX/WI
GO:200102	4	4/545	103/20610	0.290126	0.623457	1.468602	ENSG0000	HMGA2/N
GO:000193	7	7/545	203/20610	0.29152	0.623457	1.304018	ENSG0000	NR4A1/XB
GO:000021	1	1/545	13/20610	0.294251	0.623457	2.908963	ENSG0000	AURKA
GO:000202	1	1/545	13/20610	0.294251	0.623457	2.908963	ENSG0000	MC4R
GO:000267	1	1/545	13/20610	0.294251	0.623457	2.908963	ENSG0000	LBP
GO:002168	1	1/545	13/20610	0.294251	0.623457	2.908963	ENSG0000	FAIM2
GO:003408	1	1/545	13/20610	0.294251	0.623457	2.908963	ENSG0000	DSCC1
GO:004561	1	1/545	13/20610	0.294251	0.623457	2.908963	ENSG0000	TP63
GO:006017	1	1/545	13/20610	0.294251	0.623457	2.908963	ENSG0000	FGFR2
GO:006090	1	1/545	13/20610	0.294251	0.623457	2.908963	ENSG0000	WNT5A
GO:007057	1	1/545	13/20610	0.294251	0.623457	2.908963	ENSG0000	HGF
GO:007169	1	1/545	13/20610	0.294251	0.623457	2.908963	ENSG0000	TBX3
GO:009752	1	1/545	13/20610	0.294251	0.623457	2.908963	ENSG0000	FAS
GO:004311	2	2/545	41/20610	0.295592	0.623457	1.844708	ENSG0000	PDE3A/AN
GO:004688	5	5/545	137/20610	0.296668	0.624437	1.380165	ENSG0000	TARDBP/H
GO:005089	3	3/545	72/20610	0.296915	0.624437	1.575688	ENSG0000	TP63/GRH
GO:007209	3	3/545	72/20610	0.296915	0.624437	1.575688	ENSG0000	TBX3/HMG
GO:005122	8	8/545	239/20610	0.299193	0.628591	1.265825	ENSG0000	ANGPT1/II
GO:000269	7	7/545	205/20610	0.299878	0.62885	1.291296	ENSG0000	ARRB2/AN
GO:000220	3	3/545	73/20610	0.304168	0.631929	1.554103	ENSG0000	EXO1/ATA
GO:000926	2	2/545	42/20610	0.305417	0.631929	1.800786	ENSG0000	NOX1/TTP
GO:190011	2	2/545	42/20610	0.305417	0.631929	1.800786	ENSG0000	AIFM1/MT
GO:006099	4	4/545	106/20610	0.307996	0.631929	1.427038	ENSG0000	PAK3/FSTL
GO:001922	3	3/545	74/20610	0.311428	0.631929	1.533102	ENSG0000	MYH14/SC
GO:004866	3	3/545	74/20610	0.311428	0.631929	1.533102	ENSG0000	PRKG1/AP
GO:005066	10	10/545	312/20610	0.31282	0.631929	1.212068	ENSG0000	XBP1/ANG
GO:001962	1	1/545	14/20610	0.312925	0.631929	2.70118	ENSG0000	CPS1
GO:004345	1	1/545	14/20610	0.312925	0.631929	2.70118	ENSG0000	WNT5A
GO:004885	1	1/545	14/20610	0.312925	0.631929	2.70118	ENSG0000	WNT5A
GO:006102	1	1/545	14/20610	0.312925	0.631929	2.70118	ENSG0000	GRHL3

GO:00985	1	1/545	14/20610	0.312925	0.631929	2.70118	ENSG0000	TLR1
GO:00457	15	15/545	489/20610	0.314996	0.633625	1.160016	ENSG0000	PAK3/XBP
GO:00025	2	2/545	43/20610	0.315216	0.633625	1.758908	ENSG0000	CD274/FO
GO:00903	2	2/545	43/20610	0.315216	0.633625	1.758908	ENSG0000	ANGPT1/
GO:00902	6	6/545	175/20610	0.31783	0.637412	1.296566	ENSG0000	DKK1/TOB
GO:00092	9	9/545	279/20610	0.319552	0.639984	1.219888	ENSG0000	ARRB2/HS
GO:00017	4	4/545	108/20610	0.319971	0.640235	1.400612	ENSG0000	DKK1/TBX
GO:00620	4	4/545	108/20610	0.319971	0.640235	1.400612	ENSG0000	BRCA1/DG
GO:19049	8	8/545	245/20610	0.3226	0.641922	1.234825	ENSG0000	ANGPT1/I
GO:00094	7	7/545	212/20610	0.329492	0.641922	1.248658	ENSG0000	HSP90AA1
GO:00025	1	1/545	15/20610	0.331106	0.641922	2.521101	ENSG0000	LBP
GO:00025	1	1/545	15/20610	0.331106	0.641922	2.521101	ENSG0000	EXO1
GO:00097	1	1/545	15/20610	0.331106	0.641922	2.521101	ENSG0000	MLXIPL
GO:00148	1	1/545	15/20610	0.331106	0.641922	2.521101	ENSG0000	ANGPT1
GO:00217	1	1/545	15/20610	0.331106	0.641922	2.521101	ENSG0000	GBX2
GO:00351	1	1/545	15/20610	0.331106	0.641922	2.521101	ENSG0000	TP63
GO:00480	1	1/545	15/20610	0.331106	0.641922	2.521101	ENSG0000	BCL2
GO:00512	1	1/545	15/20610	0.331106	0.641922	2.521101	ENSG0000	SLC30A3
GO:00602	1	1/545	15/20610	0.331106	0.641922	2.521101	ENSG0000	LBP
GO:00719	1	1/545	15/20610	0.331106	0.641922	2.521101	ENSG0000	CPS1
GO:19047	1	1/545	15/20610	0.331106	0.641922	2.521101	ENSG0000	BMP7
GO:00481	7	7/545	213/20610	0.333761	0.643488	1.242796	ENSG0000	CPLX2/UN
GO:00316	2	2/545	45/20610	0.334714	0.643488	1.680734	ENSG0000	NCMAP/H
GO:00436	2	2/545	45/20610	0.334714	0.643488	1.680734	ENSG0000	TP63/ARE
GO:00620	15	15/545	497/20610	0.337145	0.646937	1.141343	ENSG0000	ODC1/ELC
GO:00320	4	4/545	111/20610	0.337994	0.646937	1.362757	ENSG0000	ARRB2/NV
GO:19001	3	3/545	78/20610	0.340491	0.646937	1.454481	ENSG0000	PLK1/TAR
GO:00310	2	2/545	46/20610	0.344403	0.646937	1.644196	ENSG0000	WNT5A/R
GO:00341	2	2/545	46/20610	0.344403	0.646937	1.644196	ENSG0000	PPARGC1
GO:00506	6	6/545	181/20610	0.345891	0.646937	1.253586	ENSG0000	BRCA2/SC
GO:00301	3	3/545	79/20610	0.347749	0.646937	1.43607	ENSG0000	PRKG1/HP
GO:00422	3	3/545	79/20610	0.347749	0.646937	1.43607	ENSG0000	CCNB1/SC
GO:00082	1	1/545	16/20610	0.348807	0.646937	2.363532	ENSG0000	LBP
GO:00216	1	1/545	16/20610	0.348807	0.646937	2.363532	ENSG0000	FAIM2
GO:00219	1	1/545	16/20610	0.348807	0.646937	2.363532	ENSG0000	SOX2
GO:00220	1	1/545	16/20610	0.348807	0.646937	2.363532	ENSG0000	EPHB3
GO:00424	1	1/545	16/20610	0.348807	0.646937	2.363532	ENSG0000	DIO2
GO:00906	1	1/545	16/20610	0.348807	0.646937	2.363532	ENSG0000	DKK1
GO:00197	5	5/545	148/20610	0.353975	0.653269	1.277585	ENSG0000	HIST1H2B
GO:00423	14	14/545	467/20610	0.353978	0.653269	1.133686	ENSG0000	ARRB2/GA
GO:00507	3	3/545	80/20610	0.354998	0.653269	1.418119	ENSG0000	NPY2R/N
GO:19000	3	3/545	80/20610	0.354998	0.653269	1.418119	ENSG0000	PRKG1/HP
GO:00717	7	7/545	218/20610	0.355213	0.653269	1.214292	ENSG0000	ARRB2/AN
GO:00421	6	6/545	184/20610	0.360013	0.659839	1.233147	ENSG0000	DDC/PHG
GO:00015	14	14/545	470/20610	0.362775	0.659839	1.126449	ENSG0000	UNC13A/
GO:00336	1	1/545	17/20610	0.36604	0.659839	2.224501	ENSG0000	BCL2
GO:00427	1	1/545	17/20610	0.36604	0.659839	2.224501	ENSG0000	NPY2R
GO:00448	1	1/545	17/20610	0.36604	0.659839	2.224501	ENSG0000	PCNA
GO:00485	4	4/545	116/20610	0.368091	0.662988	1.304018	ENSG0000	PPIH/TOP
GO:00069	2	2/545	49/20610	0.373176	0.667467	1.543531	ENSG0000	XBP1/AGR
GO:00510	6	6/545	188/20610	0.378892	0.670505	1.20691	ENSG0000	HSPA1B/
GO:00486	4	4/545	118/20610	0.380116	0.670505	1.281916	ENSG0000	G6PD/FST
GO:00032	2	2/545	50/20610	0.382655	0.670505	1.512661	ENSG0000	GCLM/BC
GO:00147	2	2/545	50/20610	0.382655	0.670505	1.512661	ENSG0000	PARP2/NR
GO:00603	2	2/545	50/20610	0.382655	0.670505	1.512661	ENSG0000	DKK1/WN
GO:00613	2	2/545	50/20610	0.382655	0.670505	1.512661	ENSG0000	PPARGC1
GO:00075	1	1/545	18/20610	0.382818	0.670505	2.100917	ENSG0000	APOH
GO:00519	1	1/545	18/20610	0.382818	0.670505	2.100917	ENSG0000	CHRNB4

GO:00901[illegible]	1	1/545	18/20610	0.382818	0.670505	2.100917	ENSG0000	BMP7
GO:00508[illegible]	3	3/545	84/20610	0.383871	0.67066	1.35059	ENSG0000	PRKG1/HP
GO:00720[illegible]	3	3/545	84/20610	0.383871	0.67066	1.35059	ENSG0000	SALL1/BCL
GO:00082[illegible]	6	6/545	190/2061C	0.38834	0.677344	1.194206	ENSG0000	NCALD/N(
GO:00022[illegible]	12	12/545	406/2061C	0.388472	0.677344	1.117729	ENSG0000	ARRB2/PA
GO:00971[illegible]	7	7/545	226/2061C	0.389771	0.679337	1.171308	ENSG0000	ODC1/DD(
GO:00459[illegible]	9	9/545	298/2061C	0.390342	0.679789	1.142109	ENSG0000	UNC13A/H
GO:00704[illegible]	13	13/545	443/2061C	0.390572	0.679899	1.10974	ENSG0000	ALDH3A1/
GO:00107[illegible]	2	2/545	51/20610	0.392072	0.679899	1.483001	ENSG0000	TIAM1/BM
GO:00018[illegible]	1	1/545	19/20610	0.399152	0.680476	1.990343	ENSG0000	BRCA2
GO:00105[illegible]	1	1/545	19/20610	0.399152	0.680476	1.990343	ENSG0000	BCL2
GO:00442[illegible]	1	1/545	19/20610	0.399152	0.680476	1.990343	ENSG0000	ARX
GO:00600[illegible]	1	1/545	19/20610	0.399152	0.680476	1.990343	ENSG0000	NR4A3
GO:00725[illegible]	1	1/545	19/20610	0.399152	0.680476	1.990343	ENSG0000	XBP1
GO:00351[illegible]	5	5/545	157/2061C	0.401239	0.682218	1.204348	ENSG0000	HIF1A/GRI
GO:00096[illegible]	11	11/545	374/2061C	0.402827	0.683804	1.11225	ENSG0000	ODC1/POI
GO:00513[illegible]	4	4/545	122/2061C	0.404085	0.685406	1.239886	ENSG0000	HSP90AA1
GO:00302[illegible]	4	4/545	123/2061C	0.410052	0.691126	1.229805	ENSG0000	ENPP1/HIF
GO:00301[illegible]	2	2/545	53/20610	0.410707	0.691126	1.427038	ENSG0000	PRKG1/AP
GO:00509[illegible]	5	5/545	159/2061C	0.411711	0.691126	1.189199	ENSG0000	TIAM1/SU
GO:00508[illegible]	1	1/545	20/20610	0.415056	0.691126	1.890826	ENSG0000	NPY2R
GO:00508[illegible]	13	13/545	451/2061C	0.415318	0.691299	1.090055	ENSG0000	DKK1/UN(
GO:00097[illegible]	3	3/545	89/20610	0.419518	0.694721	1.274714	ENSG0000	FGFR2/NR
GO:19000[illegible]	2	2/545	54/20610	0.419919	0.694721	1.400612	ENSG0000	PRKG1/AP
GO:19035[illegible]	2	2/545	54/20610	0.419919	0.694721	1.400612	ENSG0000	XBP1/CREI
GO:00030[illegible]	4	4/545	125/2061C	0.421949	0.697814	1.210128	ENSG0000	SUCNR1/B
GO:00030[illegible]	14	14/545	492/2061C	0.428057	0.701131	1.07608	ENSG0000	G6PD/PAR
GO:00024[illegible]	1	1/545	21/20610	0.430539	0.701131	1.800786	ENSG0000	CD274
GO:00427[illegible]	1	1/545	21/20610	0.430539	0.701131	1.800786	ENSG0000	NPY2R
GO:00467[illegible]	1	1/545	21/20610	0.430539	0.701131	1.800786	ENSG0000	HIF1A
GO:00517[illegible]	3	3/545	91/20610	0.43359	0.704786	1.246698	ENSG0000	CDC6/FGF
GO:00309[illegible]	4	4/545	128/2061C	0.439682	0.711772	1.181766	ENSG0000	DNAJB11/
GO:00702[illegible]	4	4/545	128/2061C	0.439682	0.711772	1.181766	ENSG0000	KRT81/KR
GO:19038[illegible]	4	4/545	128/2061C	0.439682	0.711772	1.181766	ENSG0000	ANGPT1/N
GO:00017[illegible]	1	1/545	22/20610	0.445612	0.711871	1.718932	ENSG0000	DKK1
GO:00020[illegible]	1	1/545	22/20610	0.445612	0.711871	1.718932	ENSG0000	NECTIN1
GO:00025[illegible]	1	1/545	22/20610	0.445612	0.711871	1.718932	ENSG0000	FOXP3
GO:00323[illegible]	1	1/545	22/20610	0.445612	0.711871	1.718932	ENSG0000	HIF1A
GO:19001[illegible]	1	1/545	22/20610	0.445612	0.711871	1.718932	ENSG0000	MTRNR2L[illegible]
GO:19038[illegible]	10	10/545	350/2061C	0.446881	0.712684	1.080472	ENSG0000	TP63/PLK1
GO:00312[illegible]	2	2/545	57/20610	0.447101	0.712684	1.326895	ENSG0000	PAK3/CD2
GO:00508[illegible]	2	2/545	57/20610	0.447101	0.712684	1.326895	ENSG0000	PRKG1/AP
GO:00027[illegible]	5	5/545	166/2061C	0.448108	0.713508	1.139052	ENSG0000	XBP1/ATA
GO:00309[illegible]	5	5/545	167/2061C	0.453264	0.718234	1.132231	ENSG0000	HOXB2/FA
GO:19015[illegible]	6	6/545	204/2061C	0.454142	0.718234	1.11225	ENSG0000	PLA2G4A/
GO:00322[illegible]	3	3/545	94/20610	0.454455	0.718234	1.20691	ENSG0000	HNRNPA2
GO:00439[illegible]	8	8/545	278/2061C	0.4545	0.718234	1.088245	ENSG0000	OAS1/HM
GO:00215[illegible]	1	1/545	23/20610	0.460288	0.718234	1.644196	ENSG0000	BTG2
GO:00216[illegible]	1	1/545	23/20610	0.460288	0.718234	1.644196	ENSG0000	FAIM2
GO:00424[illegible]	1	1/545	23/20610	0.460288	0.718234	1.644196	ENSG0000	DIO2
GO:00714[illegible]	1	1/545	23/20610	0.460288	0.718234	1.644196	ENSG0000	WNT5A
GO:20007[illegible]	1	1/545	23/20610	0.460288	0.718234	1.644196	ENSG0000	HMGA2
GO:00343[illegible]	9	9/545	320/2061C	0.472978	0.724564	1.063589	ENSG0000	NECTIN1/(
GO:00032[illegible]	1	1/545	24/20610	0.474576	0.724564	1.575688	ENSG0000	BMP7
GO:00435[illegible]	1	1/545	24/20610	0.474576	0.724564	1.575688	ENSG0000	NR4A2
GO:19000[illegible]	1	1/545	24/20610	0.474576	0.724564	1.575688	ENSG0000	SORBS1
GO:19054[illegible]	4	4/545	134/2061C	0.474634	0.724564	1.128851	ENSG0000	TP63/SOR
GO:00439[illegible]	6	6/545	210/2061C	0.481894	0.732271	1.080472	ENSG0000	AURKA/PE

GO:00108	12	12/545	436/20610	0.485142	0.733401	1.040821	ENSG0000	PLA2G4A/
GO:00712	8	8/545	286/20610	0.486198	0.733401	1.057805	ENSG0000	CXCL2/XB
GO:00094	1	1/545	25/20610	0.488486	0.733401	1.512661	ENSG0000	DDC
GO:00105	1	1/545	25/20610	0.488486	0.733401	1.512661	ENSG0000	APOBEC3E
GO:00105	1	1/545	25/20610	0.488486	0.733401	1.512661	ENSG0000	APOBEC3E
GO:00148	1	1/545	25/20610	0.488486	0.733401	1.512661	ENSG0000	HIF1A
GO:00456	1	1/545	25/20610	0.488486	0.733401	1.512661	ENSG0000	TP63
GO:00069	9	9/545	327/20610	0.498881	0.736017	1.040821	ENSG0000	EXO1/HIST
GO:00018	10	10/545	365/20610	0.499686	0.736017	1.036069	ENSG0000	ARRB2/AN
GO:00215	1	1/545	26/20610	0.502029	0.736017	1.454481	ENSG0000	DLX1
GO:00346	1	1/545	26/20610	0.502029	0.736017	1.454481	ENSG0000	MZT1
GO:00458	1	1/545	26/20610	0.502029	0.736017	1.454481	ENSG0000	ATAD5
GO:00487	1	1/545	26/20610	0.502029	0.736017	1.454481	ENSG0000	BCL2
GO:00518	1	1/545	26/20610	0.502029	0.736017	1.454481	ENSG0000	RAB27B
GO:00603	1	1/545	26/20610	0.502029	0.736017	1.454481	ENSG0000	PPARGC1E
GO:00607	1	1/545	26/20610	0.502029	0.736017	1.454481	ENSG0000	E2F8
GO:00977	1	1/545	26/20610	0.502029	0.736017	1.454481	ENSG0000	HIF1A
GO:00995	1	1/545	26/20610	0.502029	0.736017	1.454481	ENSG0000	SNAP25
GO:19004	1	1/545	26/20610	0.502029	0.736017	1.454481	ENSG0000	NOX1
GO:20000	1	1/545	26/20610	0.502029	0.736017	1.454481	ENSG0000	MAP1A
GO:00486	2	2/545	64/20610	0.507603	0.742546	1.181766	ENSG0000	DKK1/G6P
GO:00096	6	6/545	216/20610	0.509204	0.742546	1.050459	ENSG0000	CCNB1/FA
GO:19037	5	5/545	179/20610	0.513933	0.744349	1.056327	ENSG0000	HIST1H4A
GO:00023	1	1/545	27/20610	0.515213	0.744349	1.400612	ENSG0000	CD274
GO:00216	1	1/545	27/20610	0.515213	0.744349	1.400612	ENSG0000	FAIM2
GO:00316	1	1/545	27/20610	0.515213	0.744349	1.400612	ENSG0000	NPY2R
GO:00605	1	1/545	27/20610	0.515213	0.744349	1.400612	ENSG0000	BMP7
GO:00723	1	1/545	27/20610	0.515213	0.744349	1.400612	ENSG0000	APOH
GO:00801	1	1/545	27/20610	0.515213	0.744349	1.400612	ENSG0000	APOBEC3E
GO:00996	1	1/545	27/20610	0.515213	0.744349	1.400612	ENSG0000	SNAP25
GO:00512	6	6/545	218/20610	0.51819	0.747166	1.040821	ENSG0000	RRS1/HNR
GO:00095	4	4/545	142/20610	0.519878	0.748857	1.065254	ENSG0000	ARRB2/TIN
GO:00510	8	8/545	295/20610	0.521251	0.749641	1.025533	ENSG0000	ANGPT1/N
GO:00064	7	7/545	257/20610	0.52185	0.749967	1.030022	ENSG0000	RRS1/HNR
GO:00991	5	5/545	181/20610	0.523783	0.751936	1.044655	ENSG0000	PAK3/EPH
GO:19018	2	2/545	66/20610	0.524082	0.751936	1.145955	ENSG0000	DKK1/G6P
GO:19037	2	2/545	66/20610	0.524082	0.751936	1.145955	ENSG0000	TP63/BCL2
GO:00971	1	1/545	28/20610	0.52805	0.751946	1.35059	ENSG0000	NECTIN1
GO:00991	13	13/545	488/20610	0.528875	0.752875	1.007407	ENSG0000	DKK1/CPL
GO:00323	2	2/545	68/20610	0.540185	0.763191	1.11225	ENSG0000	ANGPT1/A
GO:19048	2	2/545	68/20610	0.540185	0.763191	1.11225	ENSG0000	TP63/FGFF
GO:00600	1	1/545	29/20610	0.540547	0.763191	1.304018	ENSG0000	CDC20
GO:20010	1	1/545	29/20610	0.540547	0.763191	1.304018	ENSG0000	NPY2R
GO:00706	5	5/545	185/20610	0.543216	0.766047	1.022068	ENSG0000	ATAD5/NF
GO:00305	7	7/545	263/20610	0.546335	0.769498	1.006523	ENSG0000	SLC12A2/
GO:00326	2	2/545	69/20610	0.548094	0.769498	1.096131	ENSG0000	CD83/FOX
GO:00068	1	1/545	30/20610	0.552713	0.769498	1.26055	ENSG0000	SLC12A2
GO:00159	8	8/545	304/20610	0.555441	0.772222	0.995171	ENSG0000	CCNB1/EN
GO:19035	7	7/545	266/20610	0.558379	0.775162	0.995171	ENSG0000	ANGPT1/II
GO:00329	9	9/545	344/20610	0.560108	0.775358	0.989386	ENSG0000	CCND3/AT
GO:00426	2	2/545	71/20610	0.563625	0.775358	1.065254	ENSG0000	PCNA/ARF
GO:00504	2	2/545	71/20610	0.563625	0.775358	1.065254	ENSG0000	NPY2R/AD
GO:00603	2	2/545	71/20610	0.563625	0.775358	1.065254	ENSG0000	TOB1/BMF
GO:00359	1	1/545	31/20610	0.564558	0.775358	1.219888	ENSG0000	TFF1
GO:00506	1	1/545	31/20610	0.564558	0.775358	1.219888	ENSG0000	RNF26
GO:00321	11	11/545	423/20610	0.565367	0.776224	0.983408	ENSG0000	PBK/RNF2
GO:00315	10	10/545	385/20610	0.567769	0.778787	0.982247	ENSG0000	CEACAM6
GO:19043	2	2/545	72/20610	0.571245	0.77991	1.050459	ENSG0000	SORBS1/A

GO:00326:	4	4/545	152/2061C	0.573695	0.77991	0.995171	ENSG0000	ARRB2/SU
GO:00352(	4	4/545	152/2061C	0.573695	0.77991	0.995171	ENSG0000	XRCC2/RM
GO:00420(	4	4/545	152/2061C	0.573695	0.77991	0.995171	ENSG0000	TLR1/LBP/
GO:00082:	1	1/545	32/20610	0.57609	0.77991	1.181766	ENSG0000	HSD11B2
GO:00445!	1	1/545	32/20610	0.57609	0.77991	1.181766	ENSG0000	WNT5A
GO:00519!	1	1/545	32/20610	0.57609	0.77991	1.181766	ENSG0000	ADRA2A
GO:00602!	1	1/545	32/20610	0.57609	0.77991	1.181766	ENSG0000	MYB
GO:00610:	1	1/545	32/20610	0.57609	0.77991	1.181766	ENSG0000	WNT5A
GO:00906!	1	1/545	32/20610	0.57609	0.77991	1.181766	ENSG0000	ARRB2
GO:00098!	9	9/545	349/2061C	0.577533	0.780552	0.975211	ENSG0000	TOB1/PBK
GO:00902(	8	8/545	310/2061C	0.577656	0.780552	0.97591	ENSG0000	DKK1/TOB
GO:00034(	2	2/545	73/20610	0.578768	0.780552	1.036069	ENSG0000	TSPAN12/
GO:00468(	2	2/545	73/20610	0.578768	0.780552	1.036069	ENSG0000	ENHO/AD
GO:00326(	4	4/545	154/2061C	0.584046	0.784407	0.982247	ENSG0000	IL33/CD27
GO:00108:	1	1/545	33/20610	0.587317	0.784407	1.145955	ENSG0000	TSPAN12
GO:00216!	1	1/545	33/20610	0.587317	0.784407	1.145955	ENSG0000	FAIM2
GO:00400:	1	1/545	33/20610	0.587317	0.784407	1.145955	ENSG0000	BCL2
GO:00606	1	1/545	33/20610	0.587317	0.784407	1.145955	ENSG0000	VASH2
GO:00313:	8	8/545	313/2061C	0.588571	0.785841	0.966556	ENSG0000	HSP90AA1
GO:00458:	4	4/545	155/2061C	0.589166	0.785843	0.97591	ENSG0000	DGAT2/SC
GO:00026!	9	9/545	354/2061C	0.594646	0.789824	0.961437	ENSG0000	XBP1/ATA
GO:00516(	7	7/545	276/2061C	0.597451	0.789824	0.959114	ENSG0000	POLR3G/C
GO:00508!	1	1/545	34/20610	0.598248	0.789824	1.11225	ENSG0000	CD22
GO:00603:	1	1/545	34/20610	0.598248	0.789824	1.11225	ENSG0000	DKK1
GO:00509:	2	2/545	77/20610	0.607888	0.794504	0.982247	ENSG0000	SEMA3F/V
GO:00519(	2	2/545	77/20610	0.607888	0.794504	0.982247	ENSG0000	EPHB3/CB
GO:00507	6	6/545	239/2061C	0.608094	0.794504	0.949369	ENSG0000	AMBP/ARI
GO:00326:	1	1/545	35/20610	0.608889	0.794504	1.080472	ENSG0000	IL33
GO:00427!	1	1/545	35/20610	0.608889	0.794504	1.080472	ENSG0000	RMI1
GO:00601·	1	1/545	35/20610	0.608889	0.794504	1.080472	ENSG0000	NFATC2
GO:00199:	2	2/545	78/20610	0.614925	0.798799	0.969654	ENSG0000	ENPP1/DG
GO:00435(	3	3/545	120/2061C	0.618509	0.80013	0.945413	ENSG0000	G6PD/PAR
GO:00600·	4	4/545	161/2061C	0.619099	0.80013	0.939541	ENSG0000	HIF1A/NE(
GO:00164(	1	1/545	36/20610	0.619249	0.80013	1.050459	ENSG0000	ENPEP
GO:00420!	1	1/545	36/20610	0.619249	0.80013	1.050459	ENSG0000	IL33
GO:00519!	1	1/545	36/20610	0.619249	0.80013	1.050459	ENSG0000	NPY2R
GO:00024:	2	2/545	80/20610	0.628708	0.805949	0.945413	ENSG0000	HSP90AA1
GO:00083·	2	2/545	80/20610	0.628708	0.805949	0.945413	ENSG0000	ARRB2/NR
GO:00108:	3	3/545	123/2061C	0.63515	0.810988	0.922354	ENSG0000	CEACAM6
GO:00220:	3	3/545	123/2061C	0.63515	0.810988	0.922354	ENSG0000	FAIM2/BCI
GO:00988(	3	3/545	123/2061C	0.63515	0.810988	0.922354	ENSG0000	DHFR/GST
GO:00016(	10	10/545	407/2061C	0.637829	0.81286	0.929153	ENSG0000	ALDH3A1/
GO:00508(	5	5/545	206/2061C	0.638431	0.81286	0.917877	ENSG0000	UNC13A/A
GO:00975:	6	6/545	249/2061C	0.647507	0.815046	0.911241	ENSG0000	CXCL2/CX
GO:00020(	2	2/545	83/20610	0.648657	0.815046	0.911241	ENSG0000	NECTIN1/\
GO:00015:	1	1/545	39/20610	0.648714	0.815046	0.969654	ENSG0000	WDR63
GO:00326:	1	1/545	39/20610	0.648714	0.815046	0.969654	ENSG0000	FOXP3
GO:00515!	1	1/545	39/20610	0.648714	0.815046	0.969654	ENSG0000	UNC13A
GO:00509:	6	6/545	250/2061C	0.651314	0.817842	0.907596	ENSG0000	TIAM1/SU
GO:00347(	5	5/545	210/2061C	0.655114	0.820599	0.900393	ENSG0000	G6PD/KCN
GO:00901:	9	9/545	373/2061C	0.65645	0.820599	0.912463	ENSG0000	NR4A1/AN
GO:00147·	1	1/545	40/20610	0.658021	0.820599	0.945413	ENSG0000	G6PD
GO:19027·	1	1/545	40/20610	0.658021	0.820599	0.945413	ENSG0000	BMP7
GO:00442(	7	7/545	293/2061C	0.659541	0.821791	0.903466	ENSG0000	ENPP1/PD
GO:00512:	12	12/545	495/2061C	0.661208	0.823265	0.916764	ENSG0000	ANP32B/X
GO:00215(	1	1/545	41/20610	0.667081	0.825298	0.922354	ENSG0000	FAIM2
GO:00603:	1	1/545	41/20610	0.667081	0.825298	0.922354	ENSG0000	DKK1
GO:00901(	1	1/545	41/20610	0.667081	0.825298	0.922354	ENSG0000	SERPINB7

GO:190211	2	2/545	86/20610	0.667747	0.82542	0.879454	ENSG0000	SPAG5/MN
GO:005196	8	8/545	338/20610	0.673766	0.828697	0.895065	ENSG0000	DKK1/ID4/
GO:000191	2	2/545	87/20610	0.673921	0.828697	0.869345	ENSG0000	ARRB2/IL7
GO:004662	1	1/545	42/20610	0.675902	0.828697	0.900393	ENSG0000	G6PD
GO:004828	1	1/545	42/20610	0.675902	0.828697	0.900393	ENSG0000	FGFR2
GO:190138	1	1/545	42/20610	0.675902	0.828697	0.900393	ENSG0000	KCNJ2
GO:006096	1	1/545	43/20610	0.68449	0.83384	0.879454	ENSG0000	HIST1H1B
GO:003134	8	8/545	342/20610	0.686352	0.835176	0.884597	ENSG0000	PBK/ARRB
GO:000979	3	3/545	134/20610	0.69177	0.839574	0.846638	ENSG0000	TBX3/HIF1
GO:001063	4	4/545	177/20610	0.691947	0.839574	0.85461	ENSG0000	ANGPT1/H
GO:003016	4	4/545	178/20610	0.696154	0.8424	0.849809	ENSG0000	ARRB2/PR
GO:003134	2	2/545	91/20610	0.697691	0.8424	0.831132	ENSG0000	ARRB2/IL7
GO:009772	2	2/545	91/20610	0.697691	0.8424	0.831132	ENSG0000	SORD/DRG
GO:000236	4	4/545	179/20610	0.700319	0.842655	0.845062	ENSG0000	ANGPT1/N
GO:003235	1	1/545	45/20610	0.70099	0.842655	0.840367	ENSG0000	HIF1A
GO:003438	1	1/545	45/20610	0.70099	0.842655	0.840367	ENSG0000	DGAT2
GO:007267	3	3/545	137/20610	0.706012	0.846362	0.828099	ENSG0000	SLC12A2/V
GO:002157	1	1/545	46/20610	0.708914	0.846818	0.822098	ENSG0000	FAIM2
GO:002260	1	1/545	46/20610	0.708914	0.846818	0.822098	ENSG0000	ARRB2
GO:009886	1	1/545	46/20610	0.708914	0.846818	0.822098	ENSG0000	FGFR2
GO:003210	9	9/545	391/20610	0.709704	0.847529	0.870457	ENSG0000	TIAM1/SU
GO:007142	3	3/545	138/20610	0.710646	0.848423	0.822098	ENSG0000	RRS1/HNR
GO:000836	3	3/545	139/20610	0.715224	0.851143	0.816184	ENSG0000	ID4/NCMA
GO:000958	3	3/545	139/20610	0.715224	0.851143	0.816184	ENSG0000	ARRB2/LXI
GO:007155	6	6/545	268/20610	0.715425	0.851143	0.846638	ENSG0000	ARRB2/PD
GO:005170	2	2/545	95/20610	0.720008	0.853763	0.796137	ENSG0000	HMGA2/TA
GO:003253	9	9/545	395/20610	0.720786	0.854221	0.861642	ENSG0000	PAK3/SLC1
GO:004851	1	1/545	48/20610	0.724139	0.856336	0.787844	ENSG0000	NPY2R
GO:006025	1	1/545	48/20610	0.724139	0.856336	0.787844	ENSG0000	MYB
GO:190137	2	2/545	96/20610	0.725366	0.856626	0.787844	ENSG0000	KCNJ2/KCI
GO:190303	2	2/545	97/20610	0.730637	0.860322	0.779722	ENSG0000	PRKG1/AP
GO:001081	5	5/545	230/20610	0.730915	0.860322	0.822098	ENSG0000	CEACAM6
GO:000803	1	1/545	49/20610	0.731451	0.860322	0.771766	ENSG0000	EPHB3
GO:004591	1	1/545	49/20610	0.731451	0.860322	0.771766	ENSG0000	ENPP1
GO:000940	1	1/545	50/20610	0.738569	0.864966	0.75633	ENSG0000	HSP90AA1
GO:002169	1	1/545	50/20610	0.738569	0.864966	0.75633	ENSG0000	FAIM2
GO:007016	1	1/545	50/20610	0.738569	0.864966	0.75633	ENSG0000	BMP7
GO:009055	2	2/545	99/20610	0.740921	0.865867	0.76397	ENSG0000	TP63/BCL2
GO:004593	1	1/545	51/20610	0.745499	0.866621	0.7415	ENSG0000	NPY2R
GO:004852	3	3/545	146/20610	0.74571	0.866621	0.777052	ENSG0000	OAS1/HM
GO:000728	6	6/545	278/20610	0.747336	0.867784	0.816184	ENSG0000	AURKA/PD
GO:004390	11	11/545	490/20610	0.751153	0.870476	0.848942	ENSG0000	AURKA/PD
GO:007116	3	3/545	148/20610	0.753928	0.872191	0.766551	ENSG0000	RRS1/HNR
GO:200010	2	2/545	102/20610	0.755718	0.872461	0.7415	ENSG0000	HSP90AA1
GO:000268	5	5/545	238/20610	0.757628	0.872526	0.794465	ENSG0000	IL33/PTGE
GO:003030	4	4/545	194/20610	0.757882	0.872526	0.779722	ENSG0000	UNC13A/H
GO:003582	4	4/545	194/20610	0.757882	0.872526	0.779722	ENSG0000	TYMS/HM
GO:003057	1	1/545	53/20610	0.758814	0.872526	0.713519	ENSG0000	CTSL
GO:200017	1	1/545	53/20610	0.758814	0.872526	0.713519	ENSG0000	HIF1A
GO:005109	7	7/545	325/20610	0.759445	0.873022	0.81451	ENSG0000	PPARGC1E
GO:001052	2	2/545	104/20610	0.765173	0.874352	0.727241	ENSG0000	P2RX5/BCI
GO:001017	1	1/545	54/20610	0.765208	0.874352	0.700306	ENSG0000	DKK1
GO:001052	1	1/545	54/20610	0.765208	0.874352	0.700306	ENSG0000	P2RX5
GO:003426	1	1/545	54/20610	0.765208	0.874352	0.700306	ENSG0000	ARRB2
GO:005160	1	1/545	54/20610	0.765208	0.874352	0.700306	ENSG0000	BTG2
GO:004343	4	4/545	197/20610	0.768306	0.876744	0.767848	ENSG0000	ARRB2/NV
GO:000854	1	1/545	55/20610	0.771433	0.878705	0.687573	ENSG0000	HIF1A
GO:003412	1	1/545	55/20610	0.771433	0.878705	0.687573	ENSG0000	ARRB2

GO:00326(	3	3/545	153/2061C	0.773537	0.880143	0.7415	ENSG0000	POLR3G/P
GO:00706(	2	2/545	106/2061C	0.774308	0.880143	0.713519	ENSG0000	CD274/FO
GO:00028:	1	1/545	56/20610	0.777494	0.882385	0.675295	ENSG0000	CD274
GO:19013²	4	4/545	200/2061C	0.778376	0.882698	0.75633	ENSG0000	NFATC2/B
GO:00989(	1	1/545	57/20610	0.783393	0.885857	0.663448	ENSG0000	KCNJ2
GO:00229(	4	4/545	203/2061C	0.788097	0.889128	0.745153	ENSG0000	PHGDH/C(
GO:00071(	7	7/545	336/2061C	0.788427	0.889128	0.787844	ENSG0000	ANGPT1/D
GO:00971:	1	1/545	58/20610	0.789137	0.889128	0.652009	ENSG0000	SNAP25
GO:00706(	6	6/545	293/2061C	0.790225	0.890124	0.7744	ENSG0000	ATAD5/NF
GO:00434:	7	7/545	337/2061C	0.790927	0.890685	0.785506	ENSG0000	HIST1H1B/
GO:00432	6	6/545	295/2061C	0.7955	0.89376	0.769149	ENSG0000	ARRB2/G6
GO:00434:	6	6/545	296/2061C	0.798099	0.895553	0.766551	ENSG0000	SDF2L1/ST
GO:00903²	2	2/545	112/2061C	0.799874	0.895553	0.675295	ENSG0000	FOXM1/HI
GO:00198{	7	7/545	341/2061C	0.800705	0.89592	0.776292	ENSG0000	KIF11/CEN
GO:00215²	2	2/545	113/2061C	0.803877	0.898317	0.669319	ENSG0000	FAIM2/GB
GO:00350:	1	1/545	62/20610	0.810631	0.902861	0.609944	ENSG0000	KCNU1
GO:00600²	1	1/545	62/20610	0.810631	0.902861	0.609944	ENSG0000	TSPAN12
GO:00076(	1	1/545	63/20610	0.815654	0.904992	0.600262	ENSG0000	GUCA1B
GO:00323	1	1/545	63/20610	0.815654	0.904992	0.600262	ENSG0000	MYB
GO:00329:	1	1/545	63/20610	0.815654	0.904992	0.600262	ENSG0000	TOP1
GO:00480(	7	7/545	348/2061C	0.816974	0.906227	0.760677	ENSG0000	KIF11/CEN
GO:00025:	1	1/545	64/20610	0.820544	0.90788	0.590883	ENSG0000	APOD
GO:00028:	1	1/545	64/20610	0.820544	0.90788	0.590883	ENSG0000	RNF26
GO:00083(	3	3/545	168/2061C	0.824784	0.911069	0.675295	ENSG0000	RHOV/MY
GO:00106:	6	6/545	307/2061C	0.82503	0.911069	0.739085	ENSG0000	ANGPT1/H
GO:00075{	1	1/545	65/20610	0.825304	0.911069	0.581793	ENSG0000	CHRNB4
GO:00400:	1	1/545	68/20610	0.838842	0.919273	0.556125	ENSG0000	BCL2
GO:20007	1	1/545	69/20610	0.843117	0.92197	0.548065	ENSG0000	HMGA2
GO:00702(	1	1/545	70/20610	0.84728	0.924344	0.540236	ENSG0000	FAS
GO:00990	1	1/545	70/20610	0.84728	0.924344	0.540236	ENSG0000	SNAP25
GO:00329	8	8/545	410/2061C	0.852413	0.927395	0.737883	ENSG0000	PAK3/RHC
GO:00903²	1	1/545	72/20610	0.855277	0.929121	0.525229	ENSG0000	HMGA2
GO:00022(	1	1/545	73/20610	0.859117	0.932134	0.518034	ENSG0000	IL33
GO:00458(	1	1/545	75/20610	0.866496	0.935027	0.50422	ENSG0000	PACSIN1
GO:19035:	1	1/545	75/20610	0.866496	0.935027	0.50422	ENSG0000	PTGER2
GO:20002!	1	1/545	75/20610	0.866496	0.935027	0.50422	ENSG0000	CFHR1
GO:00076:	2	2/545	132/2061C	0.867473	0.935389	0.572977	ENSG0000	MAP1A/N(
GO:00512	8	8/545	419/2061C	0.867896	0.935613	0.722034	ENSG0000	PRKG1/SEI
GO:00439(	4	4/545	236/2061C	0.873873	0.938812	0.640958	ENSG0000	OAS1/HM
GO:00343{	1	1/545	78/20610	0.876846	0.939515	0.484827	ENSG0000	DGAT2
GO:00481!	1	1/545	78/20610	0.876846	0.939515	0.484827	ENSG0000	AREG
GO:00604!	3	3/545	188/2061C	0.877453	0.939883	0.603455	ENSG0000	TBC1D30/
GO:00104(	3	3/545	191/2061C	0.884011	0.943205	0.593977	ENSG0000	DKK1/MTF
GO:00458(	3	3/545	191/2061C	0.884011	0.943205	0.593977	ENSG0000	ARRB2/AN
GO:00451:	1	1/545	81/20610	0.886395	0.945056	0.466871	ENSG0000	PTGER4
GO:00099{	1	1/545	83/20610	0.892347	0.947453	0.455621	ENSG0000	KCNU1
GO:00619(	1	1/545	83/20610	0.892347	0.947453	0.455621	ENSG0000	IL33
GO:00326:	1	1/545	84/20610	0.895206	0.948197	0.450197	ENSG0000	ARRB2
GO:00347(	2	2/545	144/2061C	0.897255	0.949532	0.525229	ENSG0000	ENPP1/AD
GO:19059!	1	1/545	85/20610	0.897988	0.949532	0.4449	ENSG0000	MYB
GO:00508(	1	1/545	86/20610	0.900697	0.951397	0.439727	ENSG0000	NPY2R
GO:00300²	2	2/545	146/2061C	0.901569	0.951934	0.518034	ENSG0000	KCNJ2/MY
GO:00075(	3	3/545	206/2061C	0.912324	0.959346	0.550726	ENSG0000	AMBP/BCI
GO:00400:	8	8/545	451/2061C	0.912565	0.959369	0.670803	ENSG0000	PRKG1/SEI
GO:00613:	2	2/545	152/2061C	0.913516	0.959675	0.497586	ENSG0000	KCNJ2/CA
GO:00506{	1	1/545	93/20610	0.917751	0.962503	0.406629	ENSG0000	RNF26
GO:00069!	1	1/545	94/20610	0.919935	0.964099	0.402303	ENSG0000	CFHR1
GO:00516(	2	2/545	156/2061C	0.92071	0.964449	0.484827	ENSG0000	SNAP25/P

GO:00324:	1	1/545	97/20610	0.926148	0.966666	0.389861	ENSG0000	ADRA2A
GO:00026{	2	2/545	160/2061C	0.927339	0.967678	0.472706	ENSG0000	LBP/WNT5
GO:00903:	2	2/545	162/2061C	0.930455	0.968382	0.466871	ENSG0000	ANP32B/T
GO:00073:	2	2/545	163/2061C	0.931964	0.969492	0.464006	ENSG0000	CDK1/KCN
GO:00102!	8	8/545	474/2061C	0.936092	0.971933	0.638253	ENSG0000	PLK1/CCN
GO:00430(	8	8/545	474/2061C	0.936092	0.971933	0.638253	ENSG0000	SH3PXD2A
GO:00322:	1	1/545	104/2061C	0.938837	0.973625	0.36362	ENSG0000	PTGER4
GO:20001‹	6	6/545	380/2061C	0.938883	0.973625	0.597103	ENSG0000	PRKG1/IL3
GO:00020:	1	1/545	106/2061C	0.942045	0.975562	0.35676	ENSG0000	KCNJ2
GO:00323{	3	3/545	229/2061C	0.943718	0.9766	0.495413	ENSG0000	SPAG5/AN
GO:00028:	2	2/545	172/2061C	0.944225	0.976661	0.439727	ENSG0000	RNF26/CD
GO:19059!	2	2/545	172/2061C	0.944225	0.976661	0.439727	ENSG0000	NUS1/MYI
GO:01200:	1	1/545	108/2061C	0.945085	0.976857	0.350153	ENSG0000	MNS1
GO:19018{	1	1/545	109/2061C	0.946545	0.978009	0.34694	ENSG0000	APOD
GO:00019:	1	1/545	111/2061C	0.94935	0.978947	0.340689	ENSG0000	IL7R
GO:00970(	1	1/545	112/2061C	0.950696	0.979872	0.337647	ENSG0000	DGAT2
GO:00432:	2	2/545	180/2061C	0.953332	0.980737	0.420183	ENSG0000	BCL2/ADR
GO:00324(	4	4/545	295/2061C	0.954689	0.981208	0.512766	ENSG0000	KCNG1/CA
GO:00109:	1	1/545	118/2061C	0.958058	0.982957	0.320479	ENSG0000	NCMAP
GO:00508(	3	3/545	249/2061C	0.962185	0.983981	0.455621	ENSG0000	PRKG1/CD
GO:00106:	1	1/545	124/2061C	0.964322	0.984216	0.304972	ENSG0000	APOH
GO:00095(	2	2/545	198/2061C	0.968913	0.986735	0.381985	ENSG0000	CDK1/KCN
GO:00019:	2	2/545	203/2061C	0.972263	0.989026	0.372576	ENSG0000	ARRB2/IL7
GO:19034(	1	1/545	142/2061C	0.978045	0.992476	0.266313	ENSG0000	HSP90AA1
GO:00434{	2	2/545	214/2061C	0.978451	0.992483	0.353425	ENSG0000	TARDBP/Z
GO:00313‹	2	2/545	223/2061C	0.9825	0.994518	0.339162	ENSG0000	ARRB2/IL7
GO:00026!	2	2/545	224/2061C	0.982901	0.994518	0.337647	ENSG0000	CD274/FO
GO:00485:	1	1/545	167/2061C	0.988823	0.997099	0.226446	ENSG0000	TRIP13
GO:00422(	1	1/545	197/2061C	0.995034	0.998911	0.191962	ENSG0000	ARRB2
GO:00512:	3	3/545	355/2061C	0.99601	0.998911	0.319576	ENSG0000	ENPP1/DG
GO:00064:	1	1/545	222/2061C	0.997476	0.998911	0.170345	ENSG0000	EIF5B
GO:00007{	26	26/569	109/21737	5.00E-18	4.08E-16	9.11243	ENSG0000	HIST1H2BI
GO:00007:	26	26/569	114/21737	1.66E-17	1.01E-15	8.712762	ENSG0000	PLK1/ZWIL
GO:00300:	10	10/569	15/21737	3.73E-13	1.66E-11	25.46807	ENSG0000	CNOT3/NA
GO:00300:	11	11/569	26/21737	1.94E-11	7.31E-10	16.16243	ENSG0000	TOB1/CNC
GO:00306{	10	10/569	84/21737	6.77E-05	0.001227	4.54787	ENSG0000	RRS1/NOC
GO:00057:	5	5/569	29/21737	0.000852	0.011308	6.586571	ENSG0000	NOLC1/NC
GO:00304!	13	13/569	178/21737	0.000856	0.011308	2.790042	ENSG0000	PLK1/AUR
GO:00003(	5	5/569	43/21737	0.005116	0.059569	4.442106	ENSG0000	PCNA/CCI
GO:00308!	4	4/569	28/21737	0.005775	0.065678	5.457444	ENSG0000	PCNA/POI
GO:00058:	6	6/569	63/21737	0.006025	0.066964	3.638296	ENSG0000	KIF11/CEN
GO:00357:	14	14/569	260/21737	0.008914	0.092745	2.057037	ENSG0000	NCL/HNRI
GO:00308{	8	8/569	133/21737	0.023897	0.192405	2.297871	ENSG0000	POLA2/NA
GO:00009:	2	2/569	10/21737	0.02678	0.204616	7.640422	ENSG0000	BRCA1/MZ
GO:00002:	2	2/569	11/21737	0.032172	0.234807	6.945838	ENSG0000	KIF18A/KII
GO:00328(	3	3/569	27/21737	0.032751	0.235519	4.244679	ENSG0000	GABRA5/S
GO:00332(	17	17/569	397/21737	0.033249	0.235635	1.635859	ENSG0000	CPLX2/UN
GO:00442!	3	3/569	28/21737	0.035988	0.251403	4.093083	ENSG0000	GABRA5/S
GO:00171(	2	2/569	12/21737	0.037948	0.257732	6.367018	ENSG0000	YARS/MAI
GO:00725(	8	8/569	150/21737	0.044009	0.27997	2.037446	ENSG0000	AMBP/SER
GO:00432(	4	4/569	52/21737	0.04689	0.293961	2.938624	ENSG0000	HSP90AA1
GO:00425:	2	2/569	16/21737	0.064437	0.366393	4.775264	ENSG0000	POLE2/DN
GO:00162:	5	5/569	101/21737	0.125759	0.614962	1.891194	ENSG0000	HSPA1B/N
GO:19024!	13	13/569	354/21737	0.139797	0.645318	1.402902	ENSG0000	GABRA5/S
GO:00306:	5	5/569	107/21737	0.14949	0.670647	1.785145	ENSG0000	UNC13A/F
GO:00442!	4	4/569	79/21737	0.152684	0.678751	1.934284	ENSG0000	NECTIN1/I
GO:00988{	4	4/569	84/21737	0.177985	0.756825	1.819148	ENSG0000	GABRA5/N
GO:00163:	9	9/569	244/21737	0.191619	0.787408	1.409094	ENSG0000	MAP7/ARI

GO:012011	4	4/569	88/21737	0.199165	0.811598	1.736459	ENSG0000	KIF4A/RAE
GO:009868	2	2/569	34/21737	0.223261	0.8841	2.247183	ENSG0000	NECTIN1/S
GO:009889	1	1/569	10/21737	0.233031	0.8841	3.820211	ENSG0000	TIAM1
GO:000591	5	5/569	129/21737	0.249598	0.8841	1.480702	ENSG0000	NECTIN1/C
GO:000211	1	1/569	11/21737	0.253116	0.8841	3.472919	ENSG0000	PLXNC1
GO:199012	1	1/569	11/21737	0.253116	0.8841	3.472919	ENSG0000	HNRNPA3
GO:000802	7	7/569	199/21737	0.266803	0.902296	1.343793	ENSG0000	DDC/UNC
GO:004461	1	1/569	12/21737	0.272677	0.902296	3.183509	ENSG0000	MAD2L1
GO:004262	1	1/569	13/21737	0.291727	0.902296	2.938624	ENSG0000	APOH
GO:004526	1	1/569	13/21737	0.291727	0.902296	2.938624	ENSG0000	ATP5G1
GO:006170	1	1/569	13/21737	0.291727	0.902296	2.938624	ENSG0000	SESN3
GO:009899	1	1/569	13/21737	0.291727	0.902296	2.938624	ENSG0000	RAB27B
GO:004273	6	6/569	175/21737	0.309658	0.902296	1.309787	ENSG0000	UNC13A/C
GO:003204	1	1/569	14/21737	0.310278	0.902296	2.728722	ENSG0000	ELMO1
GO:004322	1	1/569	14/21737	0.310278	0.902296	2.728722	ENSG0000	NCMAP
GO:004429	1	1/569	14/21737	0.310278	0.902296	2.728722	ENSG0000	HSP90AA1
GO:009924	1	1/569	14/21737	0.310278	0.902296	2.728722	ENSG0000	TIAM1
GO:001632	11	11/569	354/21737	0.324068	0.902296	1.187071	ENSG0000	SLC12A2/S
GO:000595	1	1/569	15/21737	0.328344	0.902296	2.546807	ENSG0000	PRKG1
GO:003108	1	1/569	15/21737	0.328344	0.902296	2.546807	ENSG0000	SNAP25
GO:003122	1	1/569	15/21737	0.328344	0.902296	2.546807	ENSG0000	P2RX5
GO:003820	1	1/569	15/21737	0.328344	0.902296	2.546807	ENSG0000	SESN3
GO:004302	1	1/569	16/21737	0.345938	0.904618	2.387632	ENSG0000	NOX1
GO:004445	1	1/569	16/21737	0.345938	0.904618	2.387632	ENSG0000	P2RX5
GO:009883	1	1/569	16/21737	0.345938	0.904618	2.387632	ENSG0000	UNC13A
GO:004878	3	3/569	80/21737	0.34914	0.905614	1.432579	ENSG0000	UNC13A/N
GO:003120	2	2/569	48/21737	0.358981	0.915165	1.591755	ENSG0000	CPLX2/SNA
GO:009856	2	2/569	48/21737	0.358981	0.915165	1.591755	ENSG0000	RAB27B/SI
GO:003131	1	1/569	17/21737	0.363071	0.915165	2.247183	ENSG0000	COQ3
GO:004321	1	1/569	17/21737	0.363071	0.915165	2.247183	ENSG0000	NCMAP
GO:004329	5	5/569	152/21737	0.366928	0.920143	1.256648	ENSG0000	NECTIN1/S
GO:000191	2	2/569	50/21737	0.377859	0.923887	1.528084	ENSG0000	ASRGL1/G
GO:000014	1	1/569	19/21737	0.396006	0.941085	2.010637	ENSG0000	SH3BP1
GO:004319	2	2/569	53/21737	0.405725	0.941085	1.441589	ENSG0000	CPLX2/UN
GO:004367	4	4/569	124/21737	0.408539	0.941085	1.232326	ENSG0000	CPLX2/UN
GO:003471	1	1/569	20/21737	0.411831	0.941085	1.910105	ENSG0000	SNRPF
GO:190271	1	1/569	20/21737	0.411831	0.941085	1.910105	ENSG0000	GABRA5
GO:003053	4	4/569	125/21737	0.414435	0.941085	1.222467	ENSG0000	SNRPF/PP
GO:000989	5	5/569	163/21737	0.42399	0.941085	1.171844	ENSG0000	G6PD/SNA
GO:009880	6	6/569	200/21737	0.425963	0.941085	1.146063	ENSG0000	CHRNA5/T
GO:000569	1	1/569	21/21737	0.427241	0.941085	1.819148	ENSG0000	GAR1
GO:004525	1	1/569	22/21737	0.442249	0.950665	1.736459	ENSG0000	ATP5G1
GO:003123	3	3/569	94/21737	0.447863	0.950665	1.219216	ENSG0000	SNAP25/T
GO:001646	1	1/569	23/21737	0.456863	0.950665	1.660961	ENSG0000	MYH14
GO:003317	1	1/569	23/21737	0.456863	0.950665	1.660961	ENSG0000	ATP5G1
GO:004693	1	1/569	23/21737	0.456863	0.950665	1.660961	ENSG0000	BCL2
GO:009706	13	13/569	470/21737	0.459444	0.951984	1.056654	ENSG0000	UNC13A/A
GO:003258	2	2/569	60/21737	0.468238	0.958148	1.273404	ENSG0000	GABRA5/S
GO:007101	3	3/569	97/21737	0.468297	0.958148	1.181509	ENSG0000	HNRNPA2
GO:003298	1	1/569	24/21737	0.471096	0.959858	1.591755	ENSG0000	MYH14
GO:001989	5	5/569	174/21737	0.480043	0.972062	1.097762	ENSG0000	SNAP25/C
GO:003011	3	3/569	100/21737	0.488387	0.972062	1.146063	ENSG0000	NCALD/BA
GO:003438	1	1/569	26/21737	0.498454	0.972062	1.469312	ENSG0000	APOH
GO:009738	1	1/569	26/21737	0.498454	0.972062	1.469312	ENSG0000	GUCA1B
GO:004517	2	2/569	64/21737	0.502159	0.972062	1.193816	ENSG0000	KIF4A/CDC
GO:003042	5	5/569	180/21737	0.509845	0.972062	1.06117	ENSG0000	HSP90AA1
GO:004319	5	5/569	181/21737	0.514746	0.972062	1.055307	ENSG0000	ARRB2/FU
GO:004430	4	4/569	143/21737	0.517374	0.972062	1.06859	ENSG0000	CPLX2/UN

GO:00996	3	3/569	105/21737	0.521021	0.972062	1.091489	ENSG0000	GABRA5/T
GO:00001	8	8/569	298/21737	0.521171	0.972062	1.02556	ENSG0000	CDC20/AS
GO:00059	1	1/569	28/21737	0.524399	0.972062	1.364361	ENSG0000	PIK3R3
GO:00905	1	1/569	28/21737	0.524399	0.972062	1.364361	ENSG0000	CHAMP1
GO:00312	5	5/569	183/21737	0.524486	0.972062	1.043773	ENSG0000	GABRA5/T
GO:00304	5	5/569	186/21737	0.538934	0.975333	1.026938	ENSG0000	HSP90AA1
GO:00306	8	8/569	306/21737	0.551295	0.975333	0.998748	ENSG0000	AREG/UN
GO:00059	3	3/569	110/21737	0.552489	0.975333	1.041876	ENSG0000	ENPEP/MY
GO:00444	3	3/569	110/21737	0.552489	0.975333	1.041876	ENSG0000	ACOX2/H
GO:00451	11	11/569	426/21737	0.561163	0.982457	0.986439	ENSG0000	SLC12A2/
GO:00985	5	5/569	191/21737	0.562552	0.982457	1.000055	ENSG0000	G6PD/SN
GO:00487	1	1/569	32/21737	0.572338	0.98547	1.193816	ENSG0000	NECTIN1
GO:00343	1	1/569	33/21737	0.583549	0.998891	1.15764	ENSG0000	APOH
GO:00198	8	8/569	315/21737	0.584218	0.998891	0.970212	ENSG0000	SNAP25/C
GO:00452	9	9/569	357/21737	0.592513	0.99974	0.963078	ENSG0000	ARRB2/GA
GO:00988	2	2/569	76/21737	0.595158	0.99974	1.005319	ENSG0000	TIAM1/GR
GO:00056	10	10/569	400/21737	0.603322	0.99974	0.955053	ENSG0000	E2F8/NR4
GO:00302	1	1/569	35/21737	0.605099	0.99974	1.091489	ENSG0000	SLC30A3
GO:00059	8	8/569	325/21737	0.619408	0.99974	0.94036	ENSG0000	UNC13A/
GO:00996	4	4/569	164/21737	0.625546	0.99974	0.931759	ENSG0000	GABRA5/
GO:00018	1	1/569	39/21737	0.644917	0.99974	0.979541	ENSG0000	SH3BP1
GO:00431	1	1/569	40/21737	0.654228	0.99974	0.955053	ENSG0000	MAP1A
GO:00099	1	1/569	41/21737	0.663296	0.99974	0.931759	ENSG0000	MYO1A
GO:00059	3	3/569	130/21737	0.665219	0.99974	0.881587	ENSG0000	WDR63/M
GO:00970	3	3/569	131/21737	0.670275	0.99974	0.874857	ENSG0000	WDR63/M
GO:19049	3	3/569	131/21737	0.670275	0.99974	0.874857	ENSG0000	ANP32E/A
GO:00444	1	1/569	42/21737	0.672127	0.99974	0.909574	ENSG0000	WDR63
GO:00056	5	5/569	218/21737	0.678324	0.99974	0.876195	ENSG0000	HNRNPA2
GO:00992	4	4/569	176/21737	0.679971	0.99974	0.86823	ENSG0000	GABRA5/
GO:00343	1	1/569	44/21737	0.6891	0.99974	0.86823	ENSG0000	APOH
GO:19907	1	1/569	44/21737	0.6891	0.99974	0.86823	ENSG0000	APOH
GO:00447	5	5/569	226/21737	0.708398	0.99974	0.845179	ENSG0000	E2F8/HIF1
GO:00620	10	10/569	438/21737	0.713508	0.99974	0.872194	ENSG0000	AMBP/CT
GO:00988	8	8/569	355/21737	0.714638	0.99974	0.860893	ENSG0000	FAS/KIF18
GO:00017	4	4/569	187/21737	0.724793	0.99974	0.817157	ENSG0000	KIF18A/TI
GO:00315	4	4/569	191/21737	0.739887	0.99974	0.800044	ENSG0000	SORD/HIF
GO:00319	2	2/569	100/21737	0.740801	0.99974	0.764042	ENSG0000	AIFM1/TI
GO:00313	1	1/569	52/21737	0.748662	0.99974	0.734656	ENSG0000	COQ3
GO:00985	8	8/569	368/21737	0.750625	0.99974	0.830481	ENSG0000	FAS/KIF18
GO:00432	3	3/569	149/21737	0.751793	0.99974	0.76917	ENSG0000	FUS/RBFO
GO:00302	1	1/569	54/21737	0.761679	0.99974	0.707446	ENSG0000	WDR63
GO:00303	1	1/569	54/21737	0.761679	0.99974	0.707446	ENSG0000	KCNJ2
GO:00164	1	1/569	57/21737	0.779954	0.99974	0.670212	ENSG0000	ATP5G1
GO:00321	1	1/569	57/21737	0.779954	0.99974	0.670212	ENSG0000	PLEKHG6
GO:00451	1	1/569	58/21737	0.785729	0.99974	0.658657	ENSG0000	MYO1A
GO:00003	1	1/569	59/21737	0.791353	0.99974	0.647493	ENSG0000	MPV17L2
GO:00057	1	1/569	59/21737	0.791353	0.99974	0.647493	ENSG0000	MPV17L2
GO:00163	1	1/569	61/21737	0.802162	0.99974	0.626264	ENSG0000	MYO1A
GO:00988	3	3/569	163/21737	0.803346	0.99974	0.703106	ENSG0000	ENPEP/MY
GO:00306	7	7/569	353/21737	0.820049	0.99974	0.757549	ENSG0000	ANPEP/SN
GO:00319	1	1/569	67/21737	0.831351	0.99974	0.570181	ENSG0000	ACSL6
GO:00321	1	1/569	68/21737	0.835779	0.99974	0.561796	ENSG0000	PLEKHG6
GO:00448	2	2/569	125/21737	0.842491	0.99974	0.611234	ENSG0000	KIF18A/M
GO:00015	1	1/569	72/21737	0.852361	0.99974	0.530585	ENSG0000	CSTA
GO:00321	1	1/569	73/21737	0.856239	0.99974	0.523317	ENSG0000	PLEKHG6
GO:00347	2	2/569	130/21737	0.857883	0.99974	0.587725	ENSG0000	SNRPF/W
GO:19902	2	2/569	130/21737	0.857883	0.99974	0.587725	ENSG0000	NOX1/RR
GO:00058	1	1/569	74/21737	0.860015	0.99974	0.516245	ENSG0000	FUS

GO:00995	7	7/569	375/21737	0.863478	0.99974	0.713106	ENSG0000	ARRB2/PA
GO:00436	1	1/569	75/21737	0.863692	0.99974	0.509361	ENSG0000	TOP2A
GO:00989	2	2/569	134/21737	0.869193	0.99974	0.570181	ENSG0000	GABRA5/C
GO:00990	1	1/569	77/21737	0.870759	0.99974	0.496131	ENSG0000	GABRA5
GO:00319	6	6/569	334/21737	0.873083	0.99974	0.686265	ENSG0000	NR4A1/DT
GO:00701	2	2/569	137/21737	0.877128	0.99974	0.557695	ENSG0000	SH3BP1/C
GO:00985	1	1/569	79/21737	0.87746	0.99974	0.483571	ENSG0000	L2HGDH
GO:00312	8	8/569	430/21737	0.878339	0.99974	0.710737	ENSG0000	GABRA5/K
GO:00059	1	1/569	80/21737	0.880679	0.99974	0.477526	ENSG0000	ARRB2
GO:00989	1	1/569	80/21737	0.880679	0.99974	0.477526	ENSG0000	GABRA5
GO:00312	7	7/569	386/21737	0.881754	0.99974	0.692784	ENSG0000	GABRA5/N
GO:00313	4	4/569	244/21737	0.884778	0.99974	0.626264	ENSG0000	L2HGDH/F
GO:00442	1	1/569	83/21737	0.889839	0.99974	0.460266	ENSG0000	TOP2A
GO:00319	7	7/569	393/21737	0.892289	0.99974	0.680445	ENSG0000	HSP90AA1
GO:00140	6	6/569	349/21737	0.897292	0.99974	0.65677	ENSG0000	ARRB2/PA
GO:00322	6	6/569	353/21737	0.903041	0.99974	0.649328	ENSG0000	ARRB2/PA
GO:00444	4	4/569	255/21737	0.903996	0.99974	0.599249	ENSG0000	ATP5G1/T
GO:00319	3	3/569	211/21737	0.916894	0.99974	0.543158	ENSG0000	MSTO1/BC
GO:00017	1	1/569	95/21737	0.919977	0.99974	0.402127	ENSG0000	GUCA1B
GO:00988	2	2/569	158/21737	0.921347	0.99974	0.483571	ENSG0000	ATP5G1/T
GO:00977	2	2/569	159/21737	0.923025	0.99974	0.48053	ENSG0000	NPY2R/GU
GO:00003	1	1/569	97/21737	0.924129	0.99974	0.393836	ENSG0000	MPV17L2
GO:00057	1	1/569	97/21737	0.924129	0.99974	0.393836	ENSG0000	MPV17L2
GO:00361	1	1/569	100/21737	0.929958	0.99974	0.382021	ENSG0000	MNS1
GO:00313	3	3/569	221/21737	0.931246	0.99974	0.518581	ENSG0000	L2HGDH/F
GO:00306	4	4/569	275/21737	0.931788	0.99974	0.555667	ENSG0000	AREG/NCA
GO:00601	1	1/569	101/21737	0.9318	0.99974	0.378239	ENSG0000	GUCA1B
GO:00016	1	1/569	113/21737	0.950472	0.99974	0.338072	ENSG0000	NUDT1
GO:00312	2	2/569	189/21737	0.960159	0.99974	0.404255	ENSG0000	RAB27B/C
GO:00059	7	7/569	467/21737	0.962912	0.99974	0.572623	ENSG0000	SORBS1/H
GO:00972	2	2/569	201/21737	0.969555	0.99974	0.38012	ENSG0000	NUDT1/M
GO:00098	6	6/569	429/21737	0.970096	0.99974	0.534295	ENSG0000	ANPEP/EN
GO:00306	2	2/569	207/21737	0.973413	0.99974	0.369103	ENSG0000	AREG/NCA
GO:00360	1	1/569	139/21737	0.97525	0.99974	0.274835	ENSG0000	TBC1D30
GO:00300	2	2/569	211/21737	0.975719	0.99974	0.362105	ENSG0000	SORBS2/SI
GO:00300	1	1/569	141/21737	0.976537	0.99974	0.270937	ENSG0000	SORBS2
GO:00312	3	3/569	279/21737	0.978278	0.99974	0.410775	ENSG0000	ELOVL6/XI
GO:00423	1	1/569	147/21737	0.98001	0.99974	0.259878	ENSG0000	KCNJ2
GO:00058	3	3/569	286/21737	0.981195	0.99974	0.400721	ENSG0000	MPV17L2/
GO:00316	1	1/569	152/21737	0.98251	0.99974	0.25133	ENSG0000	SORBS2
GO:00444	6	6/569	495/21737	0.990314	0.99974	0.463056	ENSG0000	TBC1D30/
GO:00306	2	2/569	283/21737	0.995447	0.99974	0.26998	ENSG0000	TLR1/WNT
GO:00300	1	1/569	216/21737	0.996844	0.99974	0.176862	ENSG0000	SORBS2
GO:00443	1	1/569	216/21737	0.996844	0.99974	0.176862	ENSG0000	MPV17L2
GO:00444	1	1/569	234/21737	0.998052	0.99974	0.163257	ENSG0000	SORBS2
GO:00036	14	14/539	83/19728	5.12E-08	6.33E-06	6.173682	ENSG0000	BLM/RFC3
GO:00314	10	10/539	92/19728	0.000206	0.008183	3.978382	ENSG0000	HIST1H1B/
GO:00441	6	6/539	35/19728	0.000334	0.012594	6.274477	ENSG0000	PFDN2/HS
GO:00314	11	11/539	123/19728	0.000559	0.020053	3.27327	ENSG0000	HIST1H1B/
GO:00166	12	12/539	149/19728	0.000819	0.022924	2.947741	ENSG0000	ALDH3A1/
GO:00506	19	19/539	310/19728	0.00094	0.024401	2.243294	ENSG0000	DDC/PHGI
GO:00517	6	6/539	45/19728	0.001333	0.033457	4.880148	ENSG0000	SDF2L1/HS
GO:00510	11	11/539	153/19728	0.003265	0.064698	2.631453	ENSG0000	CHAF1B/H
GO:00166	4	4/539	29/19728	0.007611	0.12194	5.048429	ENSG0000	DHFR/MTI
GO:00167	6	6/539	65/19728	0.008559	0.123761	3.378564	ENSG0000	SRM/HMB
GO:00228	23	23/539	496/19728	0.009963	0.133967	1.697229	ENSG0000	STEAP1/GA
GO:00977	5	5/539	56/19728	0.018189	0.185864	3.267957	ENSG0000	POLR3G/P
GO:00198	9	9/539	145/19728	0.018266	0.185864	2.271793	ENSG0000	DDC/KYNU

GO:007027	5	5/539	58/19728	0.020881	0.199027	3.155268	ENSG0000	DDC/KYNU
GO:001676	3	3/539	24/19728	0.026805	0.240287	4.575139	ENSG0000	PSAT1/BCA
GO:007172	2	2/539	10/19728	0.028995	0.245316	7.320223	ENSG0000	TLR1/LBP
GO:004317	11	11/539	217/19728	0.036692	0.281929	1.855356	ENSG0000	DDC/ACO
GO:000554	2	2/539	12/19728	0.041026	0.297043	6.100186	ENSG0000	TYMS/DHF
GO:000390	2	2/539	13/19728	0.047626	0.323081	5.63094	ENSG0000	HMGA2/N
GO:001672	2	2/539	13/19728	0.047626	0.323081	5.63094	ENSG0000	ACOX2/RF
GO:002280	17	17/539	401/19728	0.050016	0.333293	1.551668	ENSG0000	SLC9A2/SL
GO:000802	10	10/539	202/19728	0.051327	0.333684	1.811936	ENSG0000	NCL/CDC2
GO:003107	8	8/539	149/19728	0.052249	0.333684	1.96516	ENSG0000	CDK1/HIF1
GO:001684	2	2/539	14/19728	0.05458	0.333684	5.22873	ENSG0000	SDSL/CHA
GO:001910	2	2/539	14/19728	0.05458	0.333684	5.22873	ENSG0000	PCNA/NEI
GO:000534	8	8/539	156/19728	0.064822	0.35478	1.87698	ENSG0000	SLC26A9/S
GO:003406	3	3/539	34/19728	0.064992	0.35478	3.22951	ENSG0000	POLA2/NA
GO:004239	10	10/539	215/19728	0.071648	0.37728	1.702377	ENSG0000	CHAF1B/M
GO:003024	3	3/539	36/19728	0.07456	0.378908	3.050093	ENSG0000	ENPP1/SU
GO:000090	2	2/539	17/19728	0.077339	0.378908	4.306013	ENSG0000	TYMS/DHF
GO:006113	11	11/539	247/19728	0.077492	0.378908	1.630009	ENSG0000	AMBP/BIR
GO:001624	3	3/539	38/19728	0.084711	0.404833	2.889562	ENSG0000	AMBP/KCN
GO:000453	4	4/539	61/19728	0.085245	0.404833	2.400073	ENSG0000	EXO1/RAD
GO:004339	3	3/539	39/19728	0.089996	0.420912	2.81547	ENSG0000	CTSL/FST/
GO:004216	2	2/539	21/19728	0.111301	0.481666	3.48582	ENSG0000	CHRNA5/C
GO:001690	3	3/539	44/19728	0.118345	0.507829	2.49553	ENSG0000	ALDH3A1/
GO:001593	3	3/539	45/19728	0.124371	0.517874	2.440074	ENSG0000	HNRNPA3
GO:000515	2	2/539	23/19728	0.129488	0.532811	3.182705	ENSG0000	ENPP1/SO
GO:003054	3	3/539	47/19728	0.136743	0.556583	2.336241	ENSG0000	DKK1/FST/
GO:004748	6	6/539	129/19728	0.142229	0.572719	1.702377	ENSG0000	ZWINT/HS
GO:002288	2	2/539	25/19728	0.148298	0.584651	2.928089	ENSG0000	HNRNPA3
GO:009853	3	3/539	52/19728	0.169345	0.64079	2.111603	ENSG0000	NR4A1/NF
GO:005108	5	5/539	112/19728	0.192188	0.690982	1.633978	ENSG0000	SDF2L1/BI
GO:000469	2	2/539	30/19728	0.197292	0.690982	2.440074	ENSG0000	CCND3/CD
GO:000801	4	4/539	87/19728	0.214356	0.691726	1.68281	ENSG0000	DACT2/SA
GO:001687	3	3/539	59/19728	0.218154	0.691726	1.861074	ENSG0000	FARSB/YA
GO:004317	4	4/539	88/19728	0.220127	0.691726	1.663687	ENSG0000	OSBPL6/R
GO:001923	2	2/539	33/19728	0.22754	0.691726	2.218249	ENSG0000	APOBEC3E
GO:009910	5	5/539	120/19728	0.231259	0.691726	1.525046	ENSG0000	TSPAN13/
GO:000370	3	3/539	61/19728	0.232603	0.691726	1.800055	ENSG0000	NR4A1/NF
GO:000216	1	1/539	10/19728	0.242007	0.691726	3.660111	ENSG0000	AGR2
GO:000552	1	1/539	10/19728	0.242007	0.691726	3.660111	ENSG0000	FKBP5
GO:001085	1	1/539	10/19728	0.242007	0.691726	3.660111	ENSG0000	GUCA1B
GO:003537	1	1/539	10/19728	0.242007	0.691726	3.660111	ENSG0000	ADGRE2
GO:004529	1	1/539	10/19728	0.242007	0.691726	3.660111	ENSG0000	CDH26
GO:000550	2	2/539	35/19728	0.247881	0.691726	2.091492	ENSG0000	ACOX2/CY
GO:000469	1	1/539	11/19728	0.262727	0.691726	3.327374	ENSG0000	PRKG1
GO:003240	1	1/539	11/19728	0.262727	0.691726	3.327374	ENSG0000	PCNA
GO:004329	1	1/539	11/19728	0.262727	0.691726	3.327374	ENSG0000	LANCL1
GO:001984	2	2/539	38/19728	0.278475	0.698394	1.926374	ENSG0000	ADH4/UGT
GO:000165	6	6/539	162/19728	0.282706	0.698394	1.355597	ENSG0000	NPY2R/ED
GO:003369	1	1/539	12/19728	0.282882	0.698394	3.050093	ENSG0000	CD22
GO:000549	4	4/539	101/19728	0.298147	0.707368	1.449549	ENSG0000	OSBPL6/A
GO:001986	2	2/539	40/19728	0.298834	0.707368	1.830056	ENSG0000	AMBP/CD2
GO:000109	1	1/539	13/19728	0.302487	0.707368	2.81547	ENSG0000	NOP58
GO:001999	1	1/539	13/19728	0.302487	0.707368	2.81547	ENSG0000	UNC13A
GO:001514	2	2/539	42/19728	0.319099	0.720631	1.74291	ENSG0000	SLC45A4/S
GO:000531	1	1/539	14/19728	0.321557	0.720631	2.614365	ENSG0000	SLC1A2
GO:003970	1	1/539	14/19728	0.321557	0.720631	2.614365	ENSG0000	DKK1
GO:004291	1	1/539	14/19728	0.321557	0.720631	2.614365	ENSG0000	ABCG2
GO:004550	1	1/539	14/19728	0.321557	0.720631	2.614365	ENSG0000	WDR63

GO:00168	5	5/539	138/19728	0.325591	0.727507	1.326127	ENSG0000	MTHFD2/A
GO:00037	2	2/539	43/19728	0.32918	0.729128	1.702377	ENSG0000	PPIH/FKBP
GO:00481	1	1/539	15/19728	0.340106	0.729128	2.440074	ENSG0000	FST
GO:19904	1	1/539	16/19728	0.358149	0.729128	2.28757	ENSG0000	PARP2
GO:00430	2	2/539	46/19728	0.359154	0.729128	1.591353	ENSG0000	BIRC5/NLF
GO:00020	3	3/539	79/19728	0.366763	0.729128	1.389916	ENSG0000	TP63/BLM
GO:00168	2	2/539	47/19728	0.369039	0.729128	1.557494	ENSG0000	PPIH/FKBP
GO:00168	2	2/539	47/19728	0.369039	0.729128	1.557494	ENSG0000	GCLM/CPS
GO:00300	2	2/539	47/19728	0.369039	0.729128	1.557494	ENSG0000	COL9A3/C
GO:19015	2	2/539	47/19728	0.369039	0.729128	1.557494	ENSG0000	SLC28A2/S
GO:00055	1	1/539	17/19728	0.3757	0.729128	2.153007	ENSG0000	TMPO
GO:00355	3	3/539	82/19728	0.38909	0.739392	1.339065	ENSG0000	TOB1/SOR
GO:00151	1	1/539	18/19728	0.392771	0.739392	2.033395	ENSG0000	SLCO2B1
GO:00433	1	1/539	18/19728	0.392771	0.739392	2.033395	ENSG0000	FST
GO:00463	3	3/539	83/19728	0.396491	0.741011	1.322932	ENSG0000	TOB1/HM
GO:00306	6	6/539	187/19728	0.403505	0.741011	1.174367	ENSG0000	TOB1/MAI
GO:00168	2	2/539	51/19728	0.407932	0.741011	1.435338	ENSG0000	RPIA/GSTA
GO:00512	3	3/539	85/19728	0.411219	0.742561	1.291804	ENSG0000	TOX3/PIK3
GO:00046	1	1/539	20/19728	0.425529	0.752707	1.830056	ENSG0000	GALNTL6
GO:00167	9	9/539	300/19728	0.43541	0.755123	1.098033	ENSG0000	ELOVL6/ES
GO:00055	13	13/539	443/19728	0.436119	0.755123	1.074073	ENSG0000	PLA2G4A/
GO:00167	1	1/539	21/19728	0.44124	0.755123	1.74291	ENSG0000	TGM3
GO:00170	2	2/539	55/19728	0.445625	0.759175	1.33095	ENSG0000	CALCRL/M
GO:00168	2	2/539	56/19728	0.454838	0.761706	1.307183	ENSG0000	ODC1/DD
GO:00000	1	1/539	22/19728	0.456523	0.761706	1.663687	ENSG0000	ACADL
GO:00160	1	1/539	22/19728	0.456523	0.761706	1.663687	ENSG0000	PPIH
GO:00551	1	1/539	22/19728	0.456523	0.761706	1.663687	ENSG0000	CDC20
GO:00166	4	4/539	127/19728	0.458566	0.761706	1.152791	ENSG0000	AIFM1/NC
GO:00305	4	4/539	129/19728	0.470557	0.761706	1.134918	ENSG0000	GABRA5/C
GO:00168	1	1/539	23/19728	0.471388	0.761706	1.591353	ENSG0000	GUCY1B3
GO:00300	1	1/539	23/19728	0.471388	0.761706	1.591353	ENSG0000	OGN
GO:19013	1	1/539	23/19728	0.471388	0.761706	1.591353	ENSG0000	ADRA2A
GO:19908	2	2/539	58/19728	0.472997	0.76267	1.262107	ENSG0000	POLR2A/N
GO:00046	1	1/539	24/19728	0.485847	0.773453	1.525046	ENSG0000	GSTA1
GO:01200	1	1/539	24/19728	0.485847	0.773453	1.525046	ENSG0000	TTPA
GO:00166	2	2/539	60/19728	0.490787	0.779668	1.220037	ENSG0000	ACOX2/A
GO:00166	2	2/539	61/19728	0.499539	0.780983	1.200036	ENSG0000	SESN3/GS
GO:00166	2	2/539	62/19728	0.508194	0.781014	1.180681	ENSG0000	IFI30/PCY
GO:00048	12	12/539	429/19728	0.508216	0.781014	1.023807	ENSG0000	AMBP/BIR
GO:00152	3	3/539	99/19728	0.510372	0.781014	1.109125	ENSG0000	ABCG2/SL
GO:00455	1	1/539	26/19728	0.513593	0.781014	1.407735	ENSG0000	WDR63
GO:00971	2	2/539	63/19728	0.516751	0.781014	1.16194	ENSG0000	HSP90AA1
GO:00483	2	2/539	64/19728	0.525208	0.781014	1.143785	ENSG0000	CPLX2/SN
GO:00515	2	2/539	64/19728	0.525208	0.781014	1.143785	ENSG0000	DNA2/RTE
GO:00019	1	1/539	27/19728	0.526899	0.781014	1.355597	ENSG0000	CTSL
GO:00053	1	1/539	27/19728	0.526899	0.781014	1.355597	ENSG0000	SLC1A2
GO:01401	1	1/539	28/19728	0.539843	0.782195	1.307183	ENSG0000	B3GNT6
GO:00706	1	1/539	29/19728	0.552433	0.786356	1.262107	ENSG0000	USP13
GO:00037	7	7/539	257/19728	0.556597	0.790788	0.996917	ENSG0000	TOB1/E2F
GO:00167	8	8/539	299/19728	0.573812	0.793735	0.979294	ENSG0000	SDF2L1/ST
GO:00054	1	1/539	31/19728	0.576591	0.793735	1.180681	ENSG0000	SNAP25
GO:00152	1	1/539	31/19728	0.576591	0.793735	1.180681	ENSG0000	OSBPL6
GO:00167	1	1/539	31/19728	0.576591	0.793735	1.180681	ENSG0000	HPDL
GO:00303	1	1/539	31/19728	0.576591	0.793735	1.180681	ENSG0000	CDK1
GO:00435	1	1/539	31/19728	0.576591	0.793735	1.180681	ENSG0000	FAM83A
GO:00050	4	4/539	151/19728	0.594427	0.799292	0.969566	ENSG0000	PLEKHG6/
GO:00168	2	2/539	75/19728	0.611483	0.806386	0.97603	ENSG0000	HMGA2/N
GO:00718	1	1/539	35/19728	0.621072	0.813334	1.045746	ENSG0000	ARRB2

GO:00480:	2	2/539	77/19728	0.625821	0.813892	0.950678	ENSG0000	G6PD/RPI/
GO:014010	4	4/539	158/19728	0.630066	0.815569	0.92661	ENSG0000	FARSB/YAI
GO:00167-	6	6/539	240/19728	0.643675	0.825626	0.915028	ENSG0000	TYMS/PRM
GO:00430:	4	4/539	161/19728	0.644712	0.825626	0.909344	ENSG0000	NOLC1/PR
GO:00001-	3	3/539	121/19728	0.646182	0.826104	0.907466	ENSG0000	CPLX2/UN
GO:00015:	1	1/539	38/19728	0.651344	0.82952	0.963187	ENSG0000	LBP
GO:00167!	3	3/539	127/19728	0.678234	0.842567	0.864593	ENSG0000	PCNA/NEI
GO:00168	1	1/539	41/19728	0.679201	0.842567	0.89271	ENSG0000	ACSL6
GO:00422	10	10/539	409/19728	0.683685	0.845344	0.894893	ENSG0000	ANPEP/NC
GO:00162:	1	1/539	42/19728	0.687984	0.845624	0.871455	ENSG0000	HSD11B2
GO:00055:	6	6/539	252/19728	0.689408	0.845624	0.871455	ENSG0000	HMMR/LX
GO:00055:	5	5/539	212/19728	0.691096	0.845624	0.863234	ENSG0000	UNC13A/F
GO:19016{	1	1/539	44/19728	0.704837	0.849187	0.831843	ENSG0000	SLC26A9
GO:00082	2	2/539	95/19728	0.736763	0.872299	0.77055	ENSG0000	PRMT3/FA
GO:00150:	1	1/539	49/19728	0.743099	0.872938	0.746961	ENSG0000	NECTIN1
GO:00192(	5	5/539	227/19728	0.746461	0.875522	0.806192	ENSG0000	DBF4/CCN
GO:00510:	1	1/539	53/19728	0.770111	0.883984	0.690587	ENSG0000	DACT2
GO:00082(	4	4/539	193/19728	0.776563	0.884647	0.758572	ENSG0000	LXN/FGFR:
GO:00200:	3	3/539	154/19728	0.7959	0.897174	0.713009	ENSG0000	AMBP/CYF
GO:00321{	2	2/539	109/19728	0.802505	0.90192	0.67158	ENSG0000	TOP2A/US
GO:00302	1	1/539	60/19728	0.810739	0.905767	0.610019	ENSG0000	NCALD
GO:00469(	3	3/539	164/19728	0.829297	0.911778	0.669533	ENSG0000	AMBP/CYF
GO:00090!	2	2/539	116/19728	0.829575	0.911778	0.631054	ENSG0000	PHGDH/N
GO:00428{	1	1/539	68/19728	0.84847	0.92863	0.538252	ENSG0000	SLC14A1
GO:00081	1	1/539	69/19728	0.852624	0.929413	0.530451	ENSG0000	WDR4
GO:00230:	1	1/539	70/19728	0.856665	0.930835	0.522873	ENSG0000	HSP90AA1
GO:00167{	1	1/539	72/19728	0.864417	0.937905	0.508349	ENSG0000	CTU2
GO:00010!	1	1/539	75/19728	0.875269	0.942886	0.488015	ENSG0000	NOP58
GO:00055:	1	1/539	84/19728	0.902891	0.961637	0.435728	ENSG0000	CTSL
GO:00047:	3	3/539	199/19728	0.911781	0.969733	0.551776	ENSG0000	DLGAP5/C
GO:00198:	2	2/539	149/19728	0.917195	0.970011	0.49129	ENSG0000	FSTL4/FGF
GO:00512:	1	1/539	95/19728	0.928499	0.977671	0.385275	ENSG0000	HPDL
GO:00510:	3	3/539	215/19728	0.935748	0.980297	0.510713	ENSG0000	MYH14/A(
GO:00018{	7	7/539	415/19728	0.938769	0.981196	0.617368	ENSG0000	EIF5B/NOI
GO:00605{	6	6/539	390/19728	0.957539	0.990414	0.563094	ENSG0000	DEPDC1/N
GO:00044!	1	1/539	114/19728	0.957879	0.990414	0.321062	ENSG0000	CYP4F11
GO:00192(	1	1/539	116/19728	0.960162	0.990414	0.315527	ENSG0000	ANP32E
GO:010100	1	1/539	120/19728	0.964365	0.992031	0.305009	ENSG0000	USP13
GO:00045-	1	1/539	127/19728	0.970682	0.995809	0.288198	ENSG0000	EXO1
GO:00199!	1	1/539	143/19728	0.981237	0.997696	0.255952	ENSG0000	IL20RA
GO:00051	1	1/539	144/19728	0.981754	0.997696	0.254174	ENSG0000	CDH26
GO:00197{	6	6/539	449/19728	0.984737	0.997696	0.489102	ENSG0000	ASB4/BRC/
GO:01400:	1	1/539	165/19728	0.989849	0.997696	0.221825	ENSG0000	TAF1L
GO:00167(	1	1/539	176/19728	0.992535	0.997696	0.207961	ENSG0000	CYP4F11
GO:00037:	3	3/539	352/19728	0.996721	0.997696	0.311941	ENSG0000	BRCA1/FU
GO:00171	1	1/539	212/19728	0.997274	0.997696	0.172647	ENSG0000	HGF

Category
GO_BP
GO_BP
GO_BP
GO_BP
GO_BP
GO_BP
GO_BP
GO_BP
GO_BP
GO_BP
GO_BP
GO_BP
GO_BP
GO_BP
GO_BP
GO_BP
GO_BP
GO_BP
GO_BP
GO_BP
GO_BP
GO_BP
GO_BP
GO_BP
GO_BP
GO_BP
GO_BP
GO_BP
GO_BP
GO_BP
GO_BP
GO_BP
GO_BP
GO_BP
GO_BP
GO_BP
GO_BP
GO_BP
GO_BP
GO_BP
GO_BP
GO_BP
GO_BP
GO_BP
GO_BP
GO_BP
GO_BP
GO_BP
GO_BP
GO_BP
GO_BP
GO_BP
GO_BP
GO_BP
GO_BP
GO_BP
GO_BP

GO_BP
GO_BP
GO_BP
GO_BP
GO_BP
GO_BP
GO_BP
GO_BP
GO_BP
GO_BP
GO_BP
GO_BP
GO_BP
GO_BP
GO_BP
GO_BP
GO_BP
GO_BP
GO_BP
GO_BP
GO_BP
GO_BP
GO_BP
GO_BP
GO_BP
GO_BP
GO_BP
GO_BP
GO_BP
GO_BP
GO_BP
GO_BP
GO_BP
GO_BP
GO_BP
GO_BP
GO_BP
GO_BP
GO_BP
GO_BP
GO_BP
GO_BP
GO_BP
GO_BP
GO_BP
GO_BP
GO_BP
GO_BP
GO_BP
GO_BP
GO_BP
GO_BP
GO_BP
GO_BP
GO_BP
GO_BP
GO_BP
GO_BP

GO_BP
GO_BP
GO_BP
GO_BP
GO_BP
GO_BP
GO_BP
GO_BP
GO_BP
GO_BP
GO_BP
GO_BP
GO_BP
GO_BP
GO_BP
GO_BP
GO_BP
GO_BP
GO_BP
GO_BP
GO_BP
GO_BP
GO_BP
GO_BP
GO_BP
GO_BP
GO_BP
GO_BP
GO_BP
GO_BP
GO_BP
GO_BP
GO_BP
GO_BP
GO_BP
GO_BP
GO_BP
GO_BP
GO_BP
GO_BP
GO_BP
GO_BP
GO_BP
GO_BP
GO_BP
GO_BP
GO_BP
GO_BP
GO_BP
GO_BP
GO_BP
GO_BP
GO_BP
GO_BP
GO_BP
GO_BP
GO_BP
GO_BP

GO_BP
GO_BP
GO_BP
GO_BP
GO_BP
GO_BP
GO_BP
GO_BP
GO_BP
GO_BP
GO_BP
GO_BP
GO_BP
GO_BP
GO_BP
GO_BP
GO_BP
GO_BP
GO_BP
GO_BP
GO_BP
GO_BP
GO_BP
GO_BP
GO_BP
GO_BP
GO_BP
GO_BP
GO_BP
GO_BP
GO_BP
GO_BP
GO_BP
GO_BP
GO_BP
GO_BP
GO_BP
GO_BP
GO_BP
GO_BP
GO_BP
GO_BP
GO_BP
GO_BP
GO_BP
GO_BP
GO_BP
GO_BP
GO_BP
GO_BP
GO_BP
GO_BP
GO_BP
GO_BP
GO_BP
GO_BP
GO_BP
GO_BP

GO_BP
GO_BP
GO_BP
GO_BP
GO_BP
GO_BP
GO_BP
GO_BP
GO_BP
GO_BP
GO_BP
GO_BP
GO_BP
GO_BP
GO_BP
GO_BP
GO_BP
GO_BP
GO_BP
GO_BP
GO_BP
GO_BP
GO_BP
GO_BP
GO_BP
GO_BP
GO_BP
GO_BP
GO_BP
GO_BP
GO_BP
GO_BP
GO_BP
GO_BP
GO_BP
GO_BP
GO_BP
GO_BP
GO_BP
GO_BP
GO_BP
GO_BP
GO_BP
GO_BP
GO_BP
GO_BP
GO_BP
GO_BP
GO_BP
GO_BP
GO_BP
GO_BP
GO_BP
GO_BP
GO_BP
GO_BP
GO_BP
GO_BP

GO_BP
GO_BP
GO_BP
GO_BP
GO_BP
GO_BP
GO_BP
GO_BP
GO_BP
GO_BP
GO_BP
GO_BP
GO_BP
GO_BP
GO_BP
GO_BP
GO_BP
GO_BP
GO_BP
GO_BP
GO_BP
GO_BP
GO_BP
GO_BP
GO_BP
GO_BP
GO_BP
GO_BP
GO_BP
GO_BP
GO_BP
GO_BP
GO_BP
GO_BP
GO_BP
GO_BP
GO_BP
GO_BP
GO_BP
GO_BP
GO_BP
GO_BP
GO_BP
GO_BP
GO_BP
GO_BP
GO_BP
GO_BP
GO_BP
GO_BP
GO_BP
GO_BP
GO_BP
GO_BP
GO_BP
GO_BP
GO_BP
GO_BP

GO_BP
GO_BP
GO_BP
GO_BP
GO_BP
GO_BP
GO_BP
GO_BP
GO_BP
GO_BP
GO_BP
GO_BP
GO_BP
GO_BP
GO_BP
GO_BP
GO_BP
GO_BP
GO_BP
GO_BP
GO_BP
GO_BP
GO_BP
GO_BP
GO_BP
GO_BP
GO_BP
GO_BP
GO_BP
GO_BP
GO_BP
GO_BP
GO_BP
GO_BP
GO_BP
GO_BP
GO_BP
GO_BP
GO_BP
GO_BP
GO_BP
GO_BP
GO_BP
GO_BP
GO_BP
GO_BP
GO_BP
GO_BP
GO_BP
GO_BP
GO_BP
GO_BP
GO_BP
GO_BP
GO_BP
GO_BP
GO_BP
GO_BP

GO_BP
GO_BP
GO_BP
GO_BP
GO_BP
GO_BP
GO_BP
GO_BP
GO_BP
GO_BP
GO_BP
GO_BP
GO_BP
GO_BP
GO_BP
GO_BP
GO_BP
GO_BP
GO_BP
GO_BP
GO_BP
GO_BP
GO_BP
GO_BP
GO_BP
GO_BP
GO_BP
GO_BP
GO_BP
GO_BP
GO_BP
GO_BP
GO_BP
GO_BP
GO_BP
GO_BP
GO_BP
GO_BP
GO_BP
GO_BP
GO_BP
GO_BP
GO_BP
GO_BP
GO_BP
GO_BP
GO_BP
GO_BP
GO_BP
GO_BP
GO_BP
GO_BP
GO_BP
GO_BP
GO_BP
GO_BP
GO_BP
GO_BP

GO_BP
GO_BP
GO_BP
GO_BP
GO_BP
GO_BP
GO_BP
GO_BP
GO_BP
GO_BP
GO_BP
GO_BP
GO_BP
GO_BP
GO_BP
GO_BP
GO_BP
GO_BP
GO_BP
GO_BP
GO_BP
GO_BP
GO_BP
GO_BP
GO_BP
GO_BP
GO_BP
GO_BP
GO_BP
GO_BP
GO_BP
GO_BP
GO_BP
GO_BP
GO_BP
GO_BP
GO_BP
GO_BP
GO_BP
GO_BP
GO_BP
GO_BP
GO_BP
GO_BP
GO_BP
GO_BP
GO_BP
GO_BP
GO_BP
GO_BP
GO_BP
GO_BP
GO_BP
GO_BP
GO_BP
GO_BP
GO_BP
GO_BP

GO_BP
GO_BP
GO_BP
GO_BP
GO_BP
GO_BP
GO_BP
GO_BP
GO_BP
GO_BP
GO_BP
GO_BP
GO_BP
GO_BP
GO_BP
GO_BP
GO_BP
GO_BP
GO_BP
GO_BP
GO_BP
GO_BP
GO_BP
GO_BP
GO_BP
GO_BP
GO_BP
GO_BP
GO_BP
GO_BP
GO_BP
GO_BP
GO_BP
GO_BP
GO_BP
GO_BP
GO_BP
GO_BP
GO_BP
GO_BP
GO_BP
GO_BP
GO_BP
GO_BP
GO_BP
GO_BP
GO_BP
GO_BP
GO_BP
GO_BP
GO_BP
GO_BP
GO_BP
GO_BP
GO_BP
GO_BP
GO_BP
GO_BP

GO_BP
GO_BP
GO_BP
GO_BP
GO_BP
GO_BP
GO_BP
GO_BP
GO_BP
GO_BP
GO_BP
GO_BP
GO_BP
GO_BP
GO_BP
GO_BP
GO_BP
GO_BP
GO_BP
GO_BP
GO_BP
GO_BP
GO_BP
GO_BP
GO_BP
GO_BP
GO_BP
GO_BP
GO_BP
GO_BP
GO_BP
GO_BP
GO_BP
GO_BP
GO_BP
GO_BP
GO_BP
GO_BP
GO_BP
GO_BP
GO_BP
GO_BP
GO_BP
GO_BP
GO_BP
GO_BP
GO_BP
GO_BP
GO_BP
GO_BP
GO_BP
GO_BP
GO_BP
GO_BP
GO_BP
GO_BP
GO_BP
GO_BP

GO_BP
GO_BP
GO_BP
GO_BP
GO_BP
GO_BP
GO_BP
GO_BP
GO_BP
GO_BP
GO_BP
GO_BP
GO_BP
GO_BP
GO_BP
GO_BP
GO_BP
GO_BP
GO_BP
GO_BP
GO_BP
GO_BP
GO_BP
GO_BP
GO_BP
GO_BP
GO_BP
GO_BP
GO_BP
GO_BP
GO_BP
GO_BP
GO_BP
GO_BP
GO_BP
GO_BP
GO_BP
GO_BP
GO_BP
GO_BP
GO_BP
GO_BP
GO_BP
GO_BP
GO_BP
GO_BP
GO_BP
GO_BP
GO_BP
GO_BP
GO_BP
GO_BP
GO_BP
GO_BP
GO_BP
GO_BP
GO_BP
GO_BP

GO_BP
GO_BP
GO_BP
GO_BP
GO_BP
GO_BP
GO_BP
GO_BP
GO_BP
GO_BP
GO_BP
GO_BP
GO_BP
GO_BP
GO_BP
GO_BP
GO_BP
GO_BP
GO_BP
GO_BP
GO_BP
GO_BP
GO_BP
GO_BP
GO_BP
GO_BP
GO_BP
GO_BP
GO_BP
GO_BP
GO_BP
GO_BP
GO_BP
GO_BP
GO_BP
GO_BP
GO_BP
GO_BP
GO_BP
GO_BP
GO_BP
GO_BP
GO_BP
GO_BP
GO_BP
GO_BP
GO_BP
GO_BP
GO_BP
GO_BP
GO_BP
GO_BP
GO_BP
GO_BP
GO_BP
GO_BP
GO_BP
GO_BP

GO_BP
GO_BP
GO_BP
GO_BP
GO_BP
GO_BP
GO_BP
GO_BP
GO_BP
GO_BP
GO_BP
GO_BP
GO_BP
GO_BP
GO_BP
GO_BP
GO_BP
GO_BP
GO_BP
GO_BP
GO_BP
GO_BP
GO_BP
GO_BP
GO_BP
GO_BP
GO_BP
GO_BP
GO_BP
GO_BP
GO_BP
GO_CC
GO_CC
GO_CC
GO_CC
GO_CC
GO_CC
GO_CC
GO_CC
GO_CC
GO_CC
GO_CC
GO_CC
GO_CC
GO_CC
GO_CC
GO_CC
GO_CC
GO_CC
GO_CC
GO_CC
GO_CC
GO_CC
GO_CC
GO_CC
GO_CC
GO_CC
GO_CC

GO_CC
GO_CC
GO_CC
GO_CC
GO_CC
GO_CC
GO_CC
GO_CC
GO_CC
GO_CC
GO_CC
GO_CC
GO_CC
GO_CC
GO_CC
GO_CC
GO_CC
GO_CC
GO_CC
GO_CC
GO_CC
GO_CC
GO_CC
GO_CC
GO_CC
GO_CC
GO_CC
GO_CC
GO_CC
GO_CC
GO_CC
GO_CC
GO_CC
GO_CC
GO_CC
GO_CC
GO_CC
GO_CC
GO_CC
GO_CC
GO_CC
GO_CC
GO_CC
GO_CC
GO_CC
GO_CC
GO_CC
GO_CC
GO_CC
GO_CC
GO_CC
GO_CC
GO_CC
GO_CC
GO_CC
GO_CC
GO_CC
GO_CC

GO_CC
GO_CC
GO_CC
GO_CC
GO_CC
GO_CC
GO_CC
GO_CC
GO_CC
GO_CC
GO_CC
GO_CC
GO_CC
GO_CC
GO_CC
GO_CC
GO_CC
GO_CC
GO_CC
GO_CC
GO_CC
GO_CC
GO_CC
GO_CC
GO_CC
GO_CC
GO_CC
GO_CC
GO_CC
GO_CC
GO_CC
GO_CC
GO_CC
GO_CC
GO_CC
GO_CC
GO_CC
GO_CC
GO_CC
GO_CC
GO_CC
GO_CC
GO_CC
GO_CC
GO_CC
GO_CC
GO_CC
GO_CC
GO_CC
GO_CC
GO_CC
GO_CC
GO_CC
GO_CC
GO_CC
GO_CC
GO_CC
GO_CC

GO_CC
GO_CC
GO_CC
GO_CC
GO_CC
GO_CC
GO_CC
GO_CC
GO_CC
GO_CC
GO_CC
GO_CC
GO_CC
GO_CC
GO_CC
GO_CC
GO_CC
GO_CC
GO_CC
GO_CC
GO_CC
GO_CC
GO_CC
GO_CC
GO_CC
GO_CC
GO_CC
GO_CC
GO_CC
GO_CC
GO_CC
GO_CC
GO_CC
GO_CC
GO_CC
GO_CC
GO_CC
GO_CC
GO_CC
GO_CC
GO_CC
GO_CC
GO_CC
GO_CC
GO_CC
GO_MF
GO_MF
GO_MF
GO_MF
GO_MF
GO_MF
GO_MF
GO_MF
GO_MF
GO_MF
GO_MF
GO_MF
GO_MF

GO_MF
GO_MF
GO_MF
GO_MF
GO_MF
GO_MF
GO_MF
GO_MF
GO_MF
GO_MF
GO_MF
GO_MF
GO_MF
GO_MF
GO_MF
GO_MF
GO_MF
GO_MF
GO_MF
GO_MF
GO_MF
GO_MF
GO_MF
GO_MF
GO_MF
GO_MF
GO_MF
GO_MF
GO_MF
GO_MF
GO_MF
GO_MF
GO_MF
GO_MF
GO_MF
GO_MF
GO_MF
GO_MF
GO_MF
GO_MF
GO_MF
GO_MF
GO_MF
GO_MF
GO_MF
GO_MF
GO_MF
GO_MF
GO_MF
GO_MF
GO_MF
GO_MF
GO_MF
GO_MF
GO_MF
GO_MF
GO_MF
GO_MF

GO_MF
GO_MF
GO_MF
GO_MF
GO_MF
GO_MF
GO_MF
GO_MF
GO_MF
GO_MF
GO_MF
GO_MF
GO_MF
GO_MF
GO_MF
GO_MF
GO_MF
GO_MF
GO_MF
GO_MF
GO_MF
GO_MF
GO_MF
GO_MF
GO_MF
GO_MF
GO_MF
GO_MF
GO_MF
GO_MF
GO_MF
GO_MF
GO_MF
GO_MF
GO_MF
GO_MF
GO_MF
GO_MF
GO_MF
GO_MF
GO_MF
GO_MF
GO_MF
GO_MF
GO_MF
GO_MF
GO_MF
GO_MF
GO_MF
GO_MF
GO_MF
GO_MF
GO_MF
GO_MF
GO_MF
GO_MF
GO_MF
GO_MF

GO_MF
GO_MF
GO_MF
GO_MF
GO_MF
GO_MF
GO_MF
GO_MF
GO_MF
GO_MF
GO_MF
GO_MF
GO_MF
GO_MF
GO_MF
GO_MF
GO_MF
GO_MF
GO_MF
GO_MF
GO_MF
GO_MF
GO_MF
GO_MF
GO_MF
GO_MF
GO_MF
GO_MF
GO_MF
GO_MF
GO_MF
GO_MF
GO_MF
GO_MF
GO_MF
GO_MF
GO_MF
GO_MF
GO_MF
GO_MF
GO_MF
GO_MF
GO_MF
GO_MF
GO_MF
GO_MF

Supplementary Table S6

Terms	Counts	GeneRatio	BgRatio	pValue	FDR	foldEnrichı	geneID	geneSymb
hsa05322~	25	25/288	136/8031	9.76E-12	2.74E-09	5.125996		
hsa05034~	28	28/288	187/8031	8.90E-11	1.25E-08	4.175357		
hsa04110~	21	21/288	124/8031	2.28E-09	2.14E-07	4.72253		
hsa03030~	11	11/288	36/8031	2.82E-08	1.98E-06	8.520544		
hsa03440~	8	8/288	41/8031	8.45E-05	0.004748	5.441057		
hsa03460~	9	9/288	54/8031	0.000111	0.00521	4.647569		
hsa05203~	18	18/288	204/8031	0.000368	0.014782	2.460478		
hsa04217~	15	15/288	159/8031	0.000561	0.019716	2.6307		
hsa04914~	11	11/288	99/8031	0.000792	0.024741	3.09838		
hsa00480~	8	8/288	57/8031	0.000889	0.024972	3.913743		
hsa00350~	6	6/288	36/8031	0.001586	0.039135	4.647569		
hsa00270~	7	7/288	49/8031	0.001671	0.039135	3.983631		
hsa04115~	8	8/288	73/8031	0.004412	0.095365	3.055936		
hsa00670~	4	4/288	20/8031	0.00498	0.099961	5.577083		
hsa00980~	8	8/288	77/8031	0.006108	0.114418	2.897186		
hsa03430~	4	4/288	23/8031	0.008366	0.146921	4.849638		
hsa00982~	7	7/288	72/8031	0.01427	0.235876	2.711082		
hsa01524~	7	7/288	73/8031	0.015316	0.239094	2.673944		
hsa01230~	7	7/288	75/8031	0.017568	0.259826	2.602639		
hsa00071~	5	5/288	44/8031	0.019828	0.27859	3.168797		
hsa00360~	3	3/288	17/8031	0.021372	0.285972	4.920956		
hsa05204~	7	7/288	83/8031	0.028917	0.357362	2.351782		
hsa03410~	4	4/288	33/8031	0.02925	0.357362	3.380051		
hsa04924~	6	6/288	69/8031	0.036669	0.429333	2.424819		
hsa00260~	4	4/288	40/8031	0.053867	0.605464	2.788542		
hsa00130~	2	2/288	11/8031	0.056931	0.61529	5.070076		
hsa04915~	9	9/288	138/8031	0.059649	0.620787	1.818614		
hsa05202~	11	11/288	192/8031	0.084213	0.830902	1.597602		
hsa03420~	4	4/288	47/8031	0.086863	0.830902	2.373227		
hsa04114~	8	8/288	128/8031	0.088712	0.830902	1.742839		
hsa00830~	5	5/288	67/8031	0.091665	0.830902	2.081001		
hsa01523~	3	3/288	31/8031	0.098234	0.836481	2.698589		
hsa03020~	3	3/288	31/8031	0.098234	0.836481	2.698589		
hsa00330~	4	4/288	50/8031	0.103412	0.854667	2.230833		
hsa04725~	7	7/288	113/8031	0.110874	0.871466	1.727415		
hsa04270~	8	8/288	135/8031	0.111647	0.871466	1.652469		
hsa04218~	9	9/288	160/8031	0.120836	0.89586	1.568555		
hsa00040~	3	3/288	34/8031	0.121148	0.89586	2.460478		
hsa01200~	7	7/288	117/8031	0.126721	0.913043	1.668358		
hsa03320~	5	5/288	77/8031	0.141898	0.961446	1.810741		
hsa04022~	9	9/288	167/8031	0.145579	0.961446	1.502807		
hsa00240~	4	4/288	57/8031	0.146995	0.961446	1.956871		
hsa00770~	2	2/288	19/8031	0.147125	0.961446	2.935307		
hsa04370~	4	4/288	59/8031	0.16058	0.999948	1.890537		
hsa04080~	16	16/288	340/8031	0.161255	0.999948	1.312255		
hsa00900~	2	2/288	22/8031	0.18567	0.999948	2.535038		
hsa00860~	3	3/288	42/8031	0.190023	0.999948	1.991815		
hsa04614~	2	2/288	23/8031	0.198823	0.999948	2.424819		
hsa04360~	9	9/288	181/8031	0.201918	0.999948	1.386568		
hsa04210~	7	7/288	136/8031	0.215684	0.999948	1.435279		
hsa02010~	3	3/288	45/8031	0.218006	0.999948	1.859028		
hsa05161~	8	8/288	162/8031	0.225616	0.999948	1.377058		
hsa00010~	4	4/288	68/8031	0.226618	0.999948	1.640319		
hsa05211~	4	4/288	69/8031	0.234357	0.999948	1.616546		
hsa05230~	4	4/288	69/8031	0.234357	0.999948	1.616546		
hsa00053~	2	2/288	27/8031	0.252262	0.999948	2.065586		
hsa05030~	3	3/288	49/8031	0.256487	0.999948	1.70727		

hsa00650~	2	2/288	28/8031	0.265719	0.999948	1.991815
hsa05215~	5	5/288	97/8031	0.268023	0.999948	1.437393
hsa00030~	2	2/288	30/8031	0.292614	0.999948	1.859028
hsa00072~	1	1/288	10/8031	0.306086	0.999948	2.788542
hsa04612~	4	4/288	78/8031	0.306307	0.999948	1.430021
hsa04721~	4	4/288	78/8031	0.306307	0.999948	1.430021
hsa00983~	4	4/288	79/8031	0.314475	0.999948	1.41192
hsa01521~	4	4/288	79/8031	0.314475	0.999948	1.41192
hsa00512~	2	2/288	32/8031	0.319377	0.999948	1.742839
hsa04215~	2	2/288	32/8031	0.319377	0.999948	1.742839
hsa01212~	3	3/288	57/8031	0.33545	0.999948	1.467654
hsa05014~	3	3/288	57/8031	0.33545	0.999948	1.467654
hsa05134~	3	3/288	57/8031	0.33545	0.999948	1.467654
hsa04146~	4	4/288	83/8031	0.347301	0.999948	1.343876
hsa04730~	3	3/288	60/8031	0.365141	0.999948	1.394271
hsa04024~	9	9/288	216/8031	0.370806	0.999948	1.161892
hsa05210~	4	4/288	86/8031	0.371971	0.999948	1.296996
hsa00250~	2	2/288	36/8031	0.372062	0.999948	1.54919
hsa04668~	5	5/288	112/8031	0.374381	0.999948	1.244885
hsa05162~	6	6/288	138/8031	0.374736	0.999948	1.212409
hsa00140~	3	3/288	61/8031	0.374996	0.999948	1.371414
hsa05418~	6	6/288	139/8031	0.381198	0.999948	1.203687
hsa04960~	2	2/288	37/8031	0.384992	0.999948	1.50732
hsa04540~	4	4/288	88/8031	0.388387	0.999948	1.267519
hsa04141~	7	7/288	167/8031	0.391759	0.999948	1.16885
hsa04151~	14	14/288	354/8031	0.39193	0.999948	1.102813
hsa04623~	3	3/288	63/8031	0.394611	0.999948	1.327877
hsa05235~	4	4/288	89/8031	0.396576	0.999948	1.253277
hsa04929~	3	3/288	64/8031	0.404359	0.999948	1.307129
hsa04550~	6	6/288	143/8031	0.407048	0.999948	1.170017
hsa04970~	4	4/288	91/8031	0.412897	0.999948	1.225733
hsa05032~	4	4/288	91/8031	0.412897	0.999948	1.225733
hsa05033~	2	2/288	40/8031	0.423049	0.999948	1.394271
hsa00970~	3	3/288	66/8031	0.423716	0.999948	1.267519
hsa05323~	4	4/288	93/8031	0.429125	0.999948	1.199373
hsa04216~	2	2/288	41/8031	0.435467	0.999948	1.360264
hsa05226~	6	6/288	149/8031	0.445634	0.999948	1.122903
hsa00380~	2	2/288	42/8031	0.447739	0.999948	1.327877
hsa05031~	3	3/288	69/8031	0.452331	0.999948	1.212409
hsa05205~	8	8/288	205/8031	0.455064	0.999948	1.088211
hsa04713~	4	4/288	97/8031	0.461204	0.999948	1.149914
hsa00120~	1	1/288	17/8031	0.462845	0.999948	1.640319
hsa00450~	1	1/288	17/8031	0.462845	0.999948	1.640319
hsa00910~	1	1/288	17/8031	0.462845	0.999948	1.640319
hsa05231~	4	4/288	98/8031	0.469131	0.999948	1.13818
hsa04520~	3	3/288	71/8031	0.471083	0.999948	1.178257
hsa04621~	7	7/288	181/8031	0.474274	0.999948	1.078442
hsa04921~	6	6/288	154/8031	0.4774	0.999948	1.086445
hsa00061~	1	1/288	18/8031	0.482149	0.999948	1.54919
hsa01210~	1	1/288	18/8031	0.482149	0.999948	1.54919
hsa04750~	4	4/288	100/8031	0.484853	0.999948	1.115417
hsa05170~	8	8/288	212/8031	0.493048	0.999948	1.05228
hsa04390~	6	6/288	157/8031	0.49621	0.999948	1.065685
hsa00531~	1	1/288	19/8031	0.500761	0.999948	1.467654
hsa00514~	2	2/288	47/8031	0.506747	0.999948	1.186613
hsa04064~	4	4/288	104/8031	0.51572	0.999948	1.072516
hsa04971~	3	3/288	76/8031	0.516625	0.999948	1.10074
hsa05212~	3	3/288	76/8031	0.516625	0.999948	1.10074

hsa00280~	2	2/288	48/8031	0.51805	0.999948	1.161892
hsa00532~	1	1/288	20/8031	0.518707	0.999948	1.394271
hsa05167~	7	7/288	189/8031	0.520236	0.999948	1.032793
hsa05166~	8	8/288	219/8031	0.530273	0.999948	1.018645
hsa04928~	4	4/288	106/8031	0.530831	0.999948	1.05228
hsa00220~	1	1/288	21/8031	0.536009	0.999948	1.327877
hsa05130~	7	7/288	192/8031	0.537103	0.999948	1.016656
hsa04659~	4	4/288	107/8031	0.5383	0.999948	1.042445
hsa04340~	2	2/288	50/8031	0.540136	0.999948	1.115417
hsa03018~	3	3/288	79/8031	0.542924	0.999948	1.05894
hsa04931~	4	4/288	108/8031	0.545709	0.999948	1.032793
hsa04066~	4	4/288	109/8031	0.553056	0.999948	1.023318
hsa04662~	3	3/288	82/8031	0.56838	0.999948	1.020198
hsa00340~	1	1/288	23/8031	0.568777	0.999948	1.212409
hsa04012~	3	3/288	85/8031	0.592951	0.999948	0.984191
hsa00592~	1	1/288	25/8031	0.599238	0.999948	1.115417
hsa04911~	3	3/288	86/8031	0.600938	0.999948	0.972747
hsa04923~	2	2/288	56/8031	0.60211	0.999948	0.995908
hsa00790~	1	1/288	26/8031	0.613655	0.999948	1.072516
hsa04950~	1	1/288	26/8031	0.613655	0.999948	1.072516
hsa04010~	10	10/288	294/8031	0.614593	0.999948	0.948484
hsa04978~	2	2/288	58/8031	0.621323	0.999948	0.961566
hsa04211~	3	3/288	89/8031	0.624278	0.999948	0.939958
hsa00062~	1	1/288	27/8031	0.627555	0.999948	1.032793
hsa01040~	1	1/288	27/8031	0.627555	0.999948	1.032793
hsa04976~	3	3/288	90/8031	0.631848	0.999948	0.929514
hsa04744~	1	1/288	28/8031	0.640956	0.999948	0.995908
hsa00561~	2	2/288	61/8031	0.648791	0.999948	0.914276
hsa00591~	1	1/288	29/8031	0.653877	0.999948	0.961566
hsa04611~	4	4/288	124/8031	0.655099	0.999948	0.89953
hsa04213~	2	2/288	62/8031	0.65759	0.999948	0.89953
hsa04350~	3	3/288	94/8031	0.661066	0.999948	0.88996
hsa04657~	3	3/288	94/8031	0.661066	0.999948	0.88996
hsa00590~	2	2/288	63/8031	0.666212	0.999948	0.885251
hsa00630~	1	1/288	30/8031	0.666334	0.999948	0.929514
hsa04974~	3	3/288	95/8031	0.668104	0.999948	0.880592
hsa04062~	6	6/288	189/8031	0.677282	0.999948	0.885251
hsa00410~	1	1/288	31/8031	0.678345	0.999948	0.89953
hsa04927~	2	2/288	65/8031	0.682932	0.999948	0.858013
hsa00564~	3	3/288	98/8031	0.688577	0.999948	0.853635
hsa04925~	3	3/288	98/8031	0.688577	0.999948	0.853635
hsa04530~	5	5/288	161/8031	0.690735	0.999948	0.866007
hsa00230~	4	4/288	130/8031	0.691274	0.999948	0.858013
hsa04640~	3	3/288	99/8031	0.695189	0.999948	0.845013
hsa04630~	5	5/288	162/8031	0.695949	0.999948	0.860661
hsa04068~	4	4/288	131/8031	0.697035	0.999948	0.851463
hsa05221~	2	2/288	67/8031	0.698962	0.999948	0.8324
hsa00051~	1	1/288	33/8031	0.701088	0.999948	0.845013
hsa04061~	3	3/288	100/8031	0.701694	0.999948	0.836563
hsa04933~	3	3/288	100/8031	0.701694	0.999948	0.836563
hsa04728~	4	4/288	132/8031	0.702719	0.999948	0.845013
hsa04664~	2	2/288	68/8031	0.706723	0.999948	0.820159
hsa05223~	2	2/288	68/8031	0.706723	0.999948	0.820159
hsa05146~	3	3/288	102/8031	0.714387	0.999948	0.820159
hsa05120~	2	2/288	70/8031	0.721746	0.999948	0.796726
hsa04620~	3	3/288	104/8031	0.726662	0.999948	0.804387
hsa04660~	3	3/288	104/8031	0.726662	0.999948	0.804387
hsa04140~	4	4/288	137/8031	0.729986	0.999948	0.814173

hsa00500~	1	1/288	36/8031	0.732234	0.999948	0.774595
hsa04014~	7	7/288	232/8031	0.73266	0.999948	0.84137
hsa04510~	6	6/288	201/8031	0.733259	0.999948	0.8324
hsa05169~	6	6/288	201/8031	0.733259	0.999948	0.8324
hsa05164~	5	5/288	170/8031	0.735481	0.999948	0.820159
hsa05218~	2	2/288	72/8031	0.736116	0.999948	0.774595
hsa00760~	1	1/288	37/8031	0.741879	0.999948	0.75366
hsa05143~	1	1/288	37/8031	0.741879	0.999948	0.75366
hsa05100~	2	2/288	73/8031	0.743062	0.999948	0.763984
hsa04120~	4	4/288	140/8031	0.74543	0.999948	0.796726
hsa05330~	1	1/288	38/8031	0.751179	0.999948	0.733827
hsa05340~	1	1/288	38/8031	0.751179	0.999948	0.733827
hsa04918~	2	2/288	75/8031	0.756486	0.999948	0.743611
hsa04015~	6	6/288	210/8031	0.770519	0.999948	0.796726
hsa05131~	7	7/288	242/8031	0.771385	0.999948	0.806603
hsa03008~	3	3/288	112/8031	0.771665	0.999948	0.746931
hsa05332~	1	1/288	41/8031	0.777121	0.999948	0.680132
hsa05224~	4	4/288	147/8031	0.778842	0.999948	0.758787
hsa04724~	3	3/288	114/8031	0.781925	0.999948	0.733827
hsa04514~	4	4/288	148/8031	0.783321	0.999948	0.75366
hsa04810~	6	6/288	214/8031	0.785789	0.999948	0.781834
hsa04726~	3	3/288	115/8031	0.786911	0.999948	0.727446
hsa03040~	4	4/288	149/8031	0.787728	0.999948	0.748602
hsa04261~	4	4/288	149/8031	0.787728	0.999948	0.748602
hsa04932~	4	4/288	149/8031	0.787728	0.999948	0.748602
hsa04940~	1	1/288	43/8031	0.792899	0.999948	0.648498
hsa04975~	1	1/288	43/8031	0.792899	0.999948	0.648498
hsa04962~	1	1/288	44/8031	0.800366	0.999948	0.633759
hsa04742~	2	2/288	83/8031	0.804288	0.999948	0.671938
hsa04935~	3	3/288	119/8031	0.805922	0.999948	0.702994
hsa03022~	1	1/288	45/8031	0.807565	0.999948	0.619676
hsa04152~	3	3/288	120/8031	0.810446	0.999948	0.697135
hsa05135~	3	3/288	120/8031	0.810446	0.999948	0.697135
hsa05160~	4	4/288	155/8031	0.812689	0.999948	0.719624
hsa04930~	1	1/288	46/8031	0.814505	0.999948	0.606205
hsa04610~	2	2/288	85/8031	0.814862	0.999948	0.656127
hsa04919~	3	3/288	121/8031	0.814881	0.999948	0.691374
hsa00565~	1	1/288	47/8031	0.821195	0.999948	0.593307
hsa04973~	1	1/288	47/8031	0.821195	0.999948	0.593307
hsa05163~	6	6/288	225/8031	0.823809	0.999948	0.743611
hsa04512~	2	2/288	88/8031	0.829767	0.999948	0.633759
hsa04310~	4	4/288	160/8031	0.831618	0.999948	0.697135
hsa04672~	1	1/288	49/8031	0.833863	0.999948	0.56909
hsa04727~	2	2/288	89/8031	0.834491	0.999948	0.626639
hsa00510~	1	1/288	50/8031	0.839857	0.999948	0.557708
hsa04979~	1	1/288	50/8031	0.839857	0.999948	0.557708
hsa05110~	1	1/288	50/8031	0.839857	0.999948	0.557708
hsa05144~	1	1/288	50/8031	0.839857	0.999948	0.557708
hsa04380~	3	3/288	128/8031	0.843531	0.999948	0.653564
hsa04913~	1	1/288	51/8031	0.845636	0.999948	0.546773
hsa04926~	3	3/288	129/8031	0.847295	0.999948	0.648498
hsa05222~	2	2/288	92/8031	0.847962	0.999948	0.606205
hsa04666~	2	2/288	93/8031	0.852227	0.999948	0.599686
hsa04650~	3	3/288	131/8031	0.854589	0.999948	0.638597
hsa05320~	1	1/288	53/8031	0.856578	0.999948	0.52614
hsa05225~	4	4/288	168/8031	0.858586	0.999948	0.663938
hsa05017~	3	3/288	133/8031	0.861577	0.999948	0.628994
hsa01522~	2	2/288	98/8031	0.871961	0.999948	0.56909

hsa05206~	8	8/288	310/8031	0.873734	0.999948	0.719624
hsa05213~	1	1/288	58/8031	0.880668	0.999948	0.480783
hsa04916~	2	2/288	101/8031	0.882611	0.999948	0.552186
hsa04972~	2	2/288	102/8031	0.885976	0.999948	0.546773
hsa05142~	2	2/288	102/8031	0.885976	0.999948	0.546773
hsa04625~	2	2/288	104/8031	0.892439	0.999948	0.536258
hsa00310~	1	1/288	61/8031	0.893139	0.999948	0.457138
hsa05217~	1	1/288	63/8031	0.900723	0.999948	0.442626
hsa05321~	1	1/288	65/8031	0.907771	0.999948	0.429006
hsa04060~	7	7/288	294/8031	0.908825	0.999948	0.663938
hsa05165~	8	8/288	330/8031	0.911678	0.999948	0.67601
hsa04720~	1	1/288	67/8031	0.914319	0.999948	0.4162
hsa05145~	2	2/288	112/8031	0.915039	0.999948	0.497954
hsa04137~	1	1/288	68/8031	0.917418	0.999948	0.41008
hsa04670~	2	2/288	113/8031	0.917529	0.999948	0.493547
hsa05010~	9	9/288	369/8031	0.919322	0.999948	0.680132
hsa04020~	4	4/288	193/8031	0.920404	0.999948	0.577936
hsa04920~	1	1/288	69/8031	0.920405	0.999948	0.404136
hsa04934~	3	3/288	155/8031	0.920989	0.999948	0.539718
hsa04917~	1	1/288	70/8031	0.923284	0.999948	0.398363
hsa04071~	2	2/288	119/8031	0.931088	0.999948	0.468662
hsa04722~	2	2/288	119/8031	0.931088	0.999948	0.468662
hsa05214~	1	1/288	75/8031	0.936195	0.999948	0.371806
hsa05220~	1	1/288	76/8031	0.938505	0.999948	0.366913
hsa05412~	1	1/288	77/8031	0.940731	0.999948	0.362148
hsa05132~	4	4/288	206/8031	0.941878	0.999948	0.541464
hsa04260~	1	1/288	87/8031	0.95902	0.999948	0.320522
hsa05152~	3	3/288	180/8031	0.959698	0.999948	0.464757
hsa04910~	2	2/288	139/8031	0.962612	0.999948	0.401229
hsa05410~	1	1/288	90/8031	0.963318	0.999948	0.309838
hsa04658~	1	1/288	92/8031	0.965931	0.999948	0.303102
hsa04912~	1	1/288	93/8031	0.967167	0.999948	0.299843
hsa04714~	4	4/288	231/8031	0.969055	0.999948	0.482864
hsa05150~	1	1/288	96/8031	0.970613	0.999948	0.290473
hsa05414~	1	1/288	96/8031	0.970613	0.999948	0.290473
hsa04072~	2	2/288	148/8031	0.971764	0.999948	0.37683
hsa04723~	2	2/288	148/8031	0.971764	0.999948	0.37683
hsa04070~	1	1/288	99/8031	0.973698	0.999948	0.281671
hsa04145~	2	2/288	152/8031	0.975101	0.999948	0.366913
hsa04150~	2	2/288	155/8031	0.977351	0.999948	0.359812
hsa04922~	1	1/288	106/8031	0.979699	0.999948	0.26307
hsa05016~	5	5/288	306/8031	0.987176	0.999948	0.455644
hsa04142~	1	1/288	128/8031	0.991019	0.999948	0.217855
hsa00190~	1	1/288	133/8031	0.99254	0.999948	0.209665
hsa04144~	3	3/288	248/8031	0.994395	0.999948	0.337324
hsa05012~	3	3/288	249/8031	0.994562	0.999948	0.335969
hsa03010~	1	1/288	158/8031	0.997057	0.999948	0.17649
hsa03013~	1	1/288	180/8031	0.998705	0.999948	0.154919
hsa05168~	7	7/288	491/8031	0.999093	0.999948	0.397552
hsa04740~	4	4/288	448/8031	0.999948	0.999948	0.248977

ol
